# Expanding the Inositol Pyrophosphate Toolbox: Stereoselective Synthesis and Application of PP‐InsP_4_ Isomers in Plant Signaling

**DOI:** 10.1002/anie.202507058

**Published:** 2025-07-31

**Authors:** Kevin Ritter, Anne‐Sophie C. Braun, Guizhen Liu, Mengsi Lu, Verena Gaugler, Gabriel Schaaf, Henning Jacob Jessen

**Affiliations:** ^1^ Faculty of Chemistry and Pharmacy, Institute of Organic Chemistry, and CIBSS‐Centre for Integrative Biological Signaling Studies Albert‐Ludwigs University Freiburg Freiburg 79104 Germany; ^2^ Department of Plant Nutrition Institute of Crop Science and Resource Conservation Rheinische Friedrich‐Wilhelms‐Universität Bonn Bonn 53115 Germany

**Keywords:** Capillary electrophoresis mass spectrometry, Inositol phosphates, Inositol pyrophosphates, Phosphate homeostasis, Phosphorylation

## Abstract

Inositol pyrophosphates (PP‐InsPs) are highly phosphorylated signaling molecules that regulate diverse cellular processes, including phosphate homeostasis and energy metabolism across species. Despite extensive research on well‐characterized exhaustively phosphorylated PP‐InsPs, such as 5‐PP‐InsP_5_ (5‐InsP_7_) and 1,5‐(PP)_2_‐InsP_4_ (1,5‐InsP_8_), the functional relevance of less abundant not fully phosphorylated isomers, remains largely unknown. In this study, we synthesized all unsymmetric 5‐PP‐InsP_4_ isomers in enantiopure form and assigned their structures using ^31^P‐NMR analysis in combination with a chiral solvating agent. Additionally, we developed ^18^O‐labeled PP‐InsP_4_ standards for mass spectrometry in combination with capillary electrophoresis (CE‐MS), enabling the assignment of PP‐InsP_4_ in *Arabidopsis thaliana* under phosphate starvation. Our findings show that the previously detected, phosphate starvation‐induced root‐specific PP‐InsP_4_ isomer does not match any 5‐PP‐InsP_4_ isomer, contrary to previous suggestions, thus indicating an alternative phosphorylation pattern. Enzyme assays further demonstrate that *Arabidopsis* ITPK1 selectively phosphorylates [6‐OH]‐InsP_5_ and [3‐OH]‐InsP_5_ at the 5‐position, while other InsP_5_ isomers remain unchanged. This suggests that an unidentified enzymatic activity is involved in the formation of the elusive root PP‐InsP_4_ species. Our study provides a comprehensive framework for the synthesis, analysis, and functional investigation of PP‐InsP_4_, providing an entry point for future studies on their biochemical activity and their physiological roles.

## Graphical abstract

Stereochemically defined inositol pyrophosphate isomers (PP‐InsP_4_) were synthesized and assigned by NMR spectroscopy using a chiral solvating agent. In the form of isotopically labeled standards, they were applied in CE‐MS‐based profiling of plant extracts and kinase assays, enabling structural reassignment and functional insight into enzymatic inositol phosphate turnover.

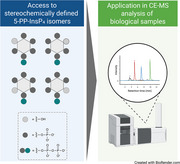

## Introduction

**Figure 1:**
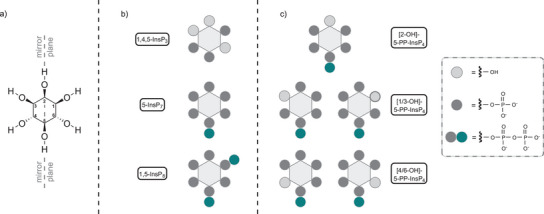
a) Structure of myo‐inositol showing the conventional position numbering and its mirror plane. b) Selected structures of biologically relevant inositol polyphosphates. c) Comprehensive overview of all possible 5‐PP‐InsP_4_ isomers.

Inositol poly‐ and pyrophosphates (InsPs and PP‐InsPs) constitute a family of highly charged signaling molecules pivotal for energy metabolism and phosphate homeostasis.^[^ ^1^, ^2^, ^3^, ^4^, ^5^ ^]^ These compounds are derived from *myo*‐inositol, a naturally occurring *meso* compound with a cyclohexane hexa‐ol scaffold and a plane of symmetry dissecting positions 2 and 5. The introduction of phosphate groups outside the mirror plane generates enantiomeric pairs. The numbering of the positions is based on a convention derived from phosphatidylinositols, where the glycerol backbone is attached to position 1. The remaining positions are numbered consecutively in a counterclockwise direction (see Figure 1a).

The first inositol phosphate that rose to prominence, the calcium release factor 1,4,5‐InsP_3_, belongs to the lower phosphorylated inositols (see Figure 1).^[^ ^6^ ^]^ Over the decades, many important new signals have been identified as part of a large network. In recent years the densely phosphorylated inositol pyrophosphates have entered center stage. In plants and mammals, the most prominent inositol pyrophosphates (PP‐InsPs) are 5‐PP‐InsP_5_ (5‐InsP_7_), 1‐PP‐InsP_5_ (1‐InsP_7_), and 1,5‐(PP)_2_‐InsP_4_ (1,5‐InsP_8_) (shown in Figure 1).^[^ ^1^, ^2^, ^3^, ^4^, ^5^ ^]^ However, recent advances in analytical methods have revealed numerous previously unnoticed isomers. For instance, 4/6‐PP‐InsP_5_, once thought to be exclusive to *Dictyostelium discoideum*, has now been identified in various other organisms. In *Arabidopsis thaliana*, for example, its abundance is comparable to or even exceeds that of 5‐PP‐InsP_5_.^[^ ^7^ ^]^ In patient peripheral blood mononuclear cell (PBMC) samples, 4/6‐PP‐InsP_5_ has been discovered as sole PP‐InsP.^[^ ^8^ ^]^ It is conceivable that new analytical technologies will uncover more previously unknown family members, such as the ones that contain triphosphates, which were only reported in vitro so far.^[^ ^9^, ^10^ ^]^

Here, we develop synthetic and analytical approaches targeting a subset of the PP‐InsP_4_ compounds, characterized by the presence of both a pyrophosphate group and one unmodified hydroxyl group. The first representative of this class was discovered in 1993 alongside PP‐InsP_5_ (InsP_7_).^[^ ^11^ ^]^ In 2008, structural elucidation by NMR identified this molecule as [2‐OH]‐5‐PP‐InsP_4_, featuring a pyrophosphate group at the 5‐position of the inositol scaffold and a free hydroxyl group at position 2 (shown in Figure 1).^[^ ^9^ ^]^ In vitro synthesis of this isomer was observed with enzymes from various organisms.^[^ ^12^, ^13^, ^14^, ^15^ ^]^ In yeast, [2‐OH]‐5‐PP‐InsP_4_ is synthesized by the kinase KCS1 and was found to be involved in the regulation of telomere length.^[^ ^16^, ^17^ ^]^ A recent study showed *Zea mays* ITPK1 to phosphorylate [6‐OH]‐InsP_5_, yielding [6‐OH]‐3‐PP‐InsP_4_ as a defined product in vitro.^[^ ^18^ ^]^ This reaction demonstrates that selected InsP_5_ isomers can be converted into PP‐InsP_4_ species by plant ITPKs under suitable conditions. In root samples of *A. thaliana* and rice grown under phosphate starvation conditions, a novel PP‐InsP_4_ isomer was detected using CE‐MS measurements. Notably, this member of the inositol pyrophosphate family defies the common trend by increasing under phosphate starvation, whereas others, such as InsP_8_, decline – sometimes even falling below the detection limit. The unknown isomer did not comigrate with isobaric InsP_6_ or a [2‐OH]‐5‐PP‐InsP_4_ standard, indicating a distinct structure.^[^ ^7^ ^]^ In a separate experiment, Whitfield et al. demonstrated that ITPK1 from *A. thaliana*, a known 5‐kinase, selectively phosphorylated [6‐OH]‐InsP_5_, while other InsP_5_ isomers remained unreactive. As ITPK1 is specific for the 5‐position in InsP_6_, the resulting product was proposed to be [6‐OH]‐5‐PP‐InsP_4_.^[^ ^19^ ^]^ However, due to the lack of reference compounds, the precise structure of this isomer remains unconfirmed both in vitro and in vivo. Although 30 different PP‐InsP_4_ isomers are theoretically possible—five of which are 5‐PP‐InsP_4_ derivatives—synthetic reference compounds are unavailable so far except for one: the only reported synthesis of a PP‐InsP_4_ compound was published by Potter et al. in 2014, who successfully prepared the symmetric [2‐OH]‐5‐PP‐InsP_4_.^[^ ^20^ ^]^

To overcome this significant shortcoming, our work focuses on the synthesis of the remaining unsymmetric 5‐PP‐InsP_4_ isomers (see Figure 1), which are of particular interest due to the predicted selective phosphorylation of the 5‐position by ITPK1. It is a candidate enzyme that may also be responsible for the generation of the inositol pyrophosphate in plant roots that defies the trend under phosphate starvation. The aim is to identify and characterize this particular isomer in plants and scrutinize the specificity of *Arabidopsis thaliana* ITPK1.

To achieve this goal, we developed a synthesis strategy targeting both racemates and enantiopure isomers containing a pyrophosphate at the 5‐position while systematically moving the free OH group around the ring. Using a chiral semipreparative HPLC column to separate synthetic intermediates, all isomers were obtained in enantiopure form. Enantiomer assignment of PP‐InsP_4_ compounds remains challenging due to the difficulty of obtaining crystal structures of the final compounds and optical rotation measurements are usually uninformative due to their inherently low values and the lack of reference data.^[^ ^21^, ^22^ ^]^ To address this particular problem, we extended a recently published method for inositol phosphate enantiomer assignment using ^31^P‐NMR spectroscopy with a chiral solvating agent.^[^ ^23^, ^24^ ^]^ The synthesis was modified such that also enantiopure InsP_5_ were obtained, which were then assigned by comparison to commercially available enantiopure InsP_5_ standards using NMR. Additionally, ^18^O isotopically labeled PP‐InsP_4_ isomers were synthesized as standards for CE‐MS analyses, enabling the assignment of unknown PP‐InsP_4_ peaks in biological samples.^[^ ^8^, ^25^, ^26^, ^27^ ^]^ The availability of both enantiopure and isotopically labeled PP‐InsP_4_ derivatives provides a valuable toolbox for future studies, including enzyme assays and investigations into their biological functions.

## Results and Discussion

### Enantioselective Synthesis of [1‐OH]‐ and [3‐OH]‐5‐PP‐InsP_4_

**Figure 2:**
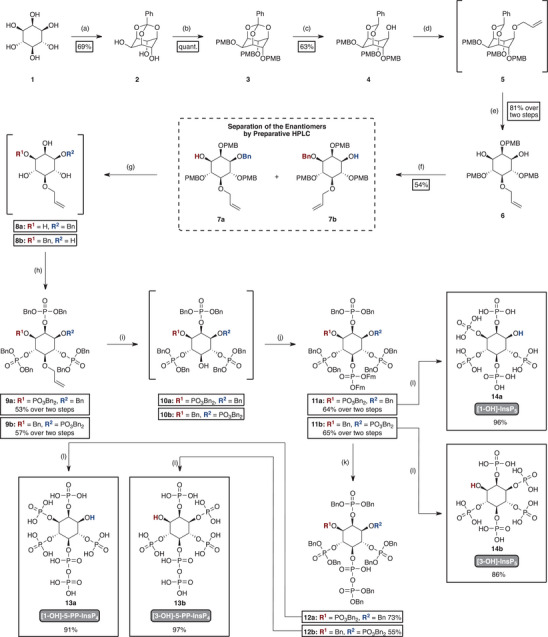
Schematic overview of the synthesis of enantiopure [1‐OH]‐ and [3‐OH]‐PP‐InsP_4_ (**13a** and **13b**), as well as [1‐OH]‐ and 3‐[OH]‐InsP_5_ (**14a** and **14b**). Reagents and conditions: a) trimethyl orthobenzoate (1.0 eq), camphorsulfonic acid (2 mol%), DMSO, 80 °C, 3 h. b) PMB‐Cl (3.3 eq), NaH (3.5 eq), TBAI, DMF, 0 °C, overnight. c) DIBAL‐H (3.0 eq), DCM, ‐78 °C, 3 h. d) allyl bromide (2.0 eq), NaH (2.5 eq), DMF, 0 °C, overnight. e) pTsOH (0.9 eq), MeOH/CH_2_Cl_2_, 0 °C, 2 h. f) benzyl bromide (1.0 eq), NaH (1.0 eq), DMF, 0 °C, overnight. g) TFA (5%) in CHCl_3_, rt, 1 h. h) Bn‐PA (6.0 eq), ETT (6.0 eq), DMF, rt, 30 min; then mCPBA (6.0 eq), 0 °C, 10 min. i) PdCl_2_ (2.0 eq), MeOH, rt, 2 h. j) Fm‐PA (2.0 eq), ETT (2.0 eq), CH_2_Cl_2_, rt, 30 min; then mCPBA (6.0 eq), 0 °C, 10 min. k) DBU (4.0 eq), BSTFA (4.0 eq), MeCN, rt, 10 min; then TFA (4.0 eq) in MeOH, rt, 5 min; then Bn‐PA (2.0 eq), ETT (2.0 eq), MeCN, 15 min, rt; then mCPBA (2.0 eq), 0 °C, 10 min. l) H_2_ (30 bar), Pd/C (3.0 eq), NaHCO_3_, tBuOH/H_2_O (4:1), rt, 5 h. Bn‐PA, bis‐benzyl‐N,N‐diisopropylamino phosphoramidite; BSTFA, N,O‐bis(trimethylsilyl) trifluoroacetamide; DBU, 1,8‐Diazabicyclo[5.4.0]undec‐7‐ene; ETT, 5‐ethylthio‐1H‐tetrazole; Fm‐PA, Bis(9H‐fluoren‐9‐ylmethyl)‐N,N‐diisopropylamino phosphoramidite.

The starting point of the synthesis of [1‐OH]‐ and [3‐OH]‐5‐PP‐InsP_4_ (**13a** and **13b**) was a previously described route, designed to selectively access the 5‐position of the inositol scaffold, as well as the 1‐ and 3‐positions.^[^ ^21^, ^22^ ^]^ *myo*‐Inositol (**1**) was first protected as an orthobenzoate (**2**), followed by the introduction of *para*‐methoxybenzyl‐groups (PMB) as protecting groups, allowing for selective modifications in subsequent steps (Figure 2).^[^ ^21^, ^22^ ^]^ Selective access to the 5‐position was achieved by opening the orthoester with diisobutylaluminium hydride (DIBAL‐H) under controlled temperature, yielding the 1,3‐acetal‐protected inositol **4** with a free hydroxy group at position 5. The regioselectivity likely results from coordination to the axial oxygen at position 5, followed by reduction from the less hindered face.^[^ ^22^, ^28^, ^29^ ^]^ The outcome strongly depends on the choice of reducing agent: while DIBAL‐H gives the 1,3‐acetal, use of trimethylaluminium results in predominant formation of the 1,5‐regioisomer.^[^ ^28^, ^29^ ^]^ Next, the free hydroxyl group was alkylated with allyl bromide using NaH as a base. The resulting intermediate **5** was not isolated but was directly subjected to mild acidic conditions to selectively hydrolyze the acetal without affecting the PMB‐groups. This step produced inositol derivative **6**, bearing two free hydroxyl groups at positions 1 and 3. One of these was subsequently benzylated using benzyl bromide under basic conditions, affording a mixture of mono‐ and dibenzylated products. The desired mono‐benzylated compound was isolated by chromatography in 54% yield. No further optimization was attempted, as this yield was sufficient for the following steps. As *myo*‐inositol contains a mirror plane that passes through positions 2 and 5, substitution at position 1 or 3 disrupts this symmetry and results in a pair of enantiomers, as is the case in this step. The obtained enantiomers (**7a** and **7b**) were efficiently separated using a chiral polysaccharide‐based preparative HPLC column (Chiralpak AD‐H, Daicel), achieving an enantiomeric ratio (*er*) > 99:1, with a difference in retention times of approximately 15 min. From this stage onward, all reactions were performed both on the racemate and separately also for each enantiomer.

The PMB‐groups were cleaved next using trifluoroacetic acid (TFA), yielding the crude products **8a** and **8b** with four unprotected hydroxyl groups. These were fully phosphorylated in the subsequent step using benzyl phosphoramidite **S1** (Bn‐PA) as the phosphitylating agent followed by oxidation with *meta*‐chloroperoxybenzoic acid (*m*CPBA) to give tetraphosphates **9a** and **9b**. The benzyl groups on the phosphates are installed as orthogonal protecting groups to withstand the next reaction conditions. The allyl group at the 5‐position was selectively cleaved with PdCl_2_, generating a free hydroxyl group that was phosphorylated without isolation using the phosphoramidite approach described above with Fm‐phosphoramidite **S2** (Fm‐PA). This reaction provided access to pentakisphosphates **11a** and **11b**. The newly introduced phosphate in 5‐position is protected by an Fm‐group (fluorenylmethyl), which is orthogonal to the benzyl‐protected phosphates. As a result, the Fm‐protected phosphate at the 5‐position can be selectively addressed under mild basic conditions (e.g., with diazabicycloundecene, DBU).

Installation of the diphosphate was performed in a one‐flask reaction sequence, combining the removal of the Fm‐group with the introduction of a second phosphate. Although one Fm‐group was readily removed with DBU, the resulting negative charge slows down the cleavage of the second Fm. Following previously described procedures,^[^ ^22^ ^]^ the phosphate was temporarily masked with a TMS group using *N*,*O*‐bis(trimethylsilyl)trifluoroacetamide (BSTFA), facilitating complete deprotection under basic conditions. The resulting bis‐TMS‐phosphate was then treated with TFA in MeOH, furnishing a free phosphate group, which was subsequently phosphorylated by the phosphoramidite approach using Bn‐phosphoramidite **S1** (Bn‐PA). This one‐flask procedure afforded pentakisphosphates **12a** and **12b** in high purity.

Finally, benzyl‐protected intermediates **12a** and **12b** were hydrogenated, removing all protecting groups to yield [1‐OH]‐ (**13b**) and [3‐OH]‐5‐PP‐InsP_4_ (**13a**). In addition, enantiopure [1‐OH]‐ (**14b**) and [3‐OH]‐InsP_5_ (**14a**) were obtained by hydrogenating intermediates **11a** and **11b** prior to diphosphorylation. These compounds served as references for enantiomer assignment by NMR with a chiral solvating agent.

Next, an ^18^O‐labeled version of the compounds was synthesized for use as an internal heavy isotope standard in mass spectrometry in combination with capillary electrophoresis (CE‐MS) measurements.^[^ ^26^, ^27^ ^]^ Because enantiomers are not resolved by CE‐MS, it was sufficient to generate the racemate. To introduce the stable isotope label, an ^18^O‐labeled variant (compound **S3**)^[^ ^30^ ^]^ instead of the ^16^O‐Bn‐phosphoramidite was used in the diphosphorylation step, yielding the ^18^O_2_‐labeled [1/3‐OH]‐5‐PP‐InsP_4_ (±**13**) in 85% yield with > 99% isotopic purity. Such compounds are of high value in quantitative MS analysis and isomer assignment as shown below.

### Enantioselective Synthesis of [4‐OH]‐ and [6‐OH]‐5‐PP‐InsP_4_

**Figure 3:**
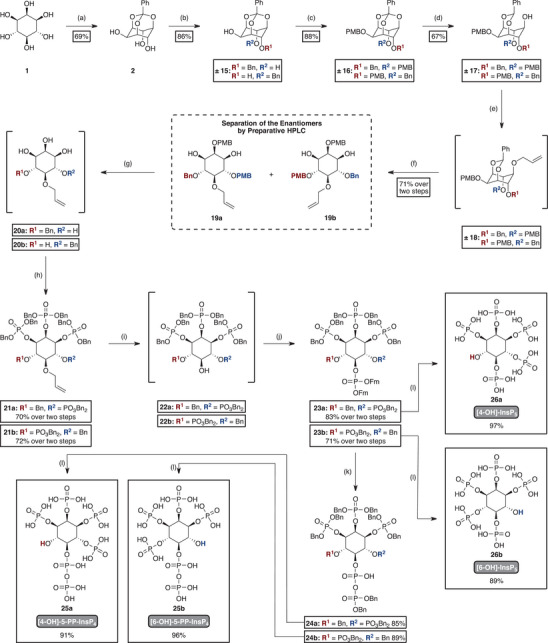
Schematic overview of the synthesis of enantiopure [4‐OH]‐ and [6‐OH]‐PP‐InsP_4_ (**25a** and **25b**), as well as [4‐OH]‐ and [6‐OH]‐InsP_5_ (**26a** and **26b**). Reagents and conditions: a) trimethyl orthobenzoate (1.0 eq), camphorsulfonic acid (2 mol%), DMSO, 80 °C, 3 h. b) benzyl bromide (1.0 eq), NaH (0.95 eq), DMF, 0 °C, 60 min. c) PMB‐Cl (2.2 eq), NaH (2.5 eq), TBAI, DMF, rt, 2 h. d) DIBAL‐H (3.0 eq), DCM, ‐78 °C, 3 h. e) allyl bromide (2.0 eq), NaH (2.5 eq), DMF, 0 °C, overnight. f) pTsOH (0.9 eq), MeOH/CH_2_Cl_2_, 0 °C, 2 h. g) TFA (5%) in CHCl_3_, rt, 1 h. h) Bn‐PA (6.0 eq), ETT (6.0 eq), DMF, rt, 30 min; then mCPBA (6.0 eq), 0 °C, 10 min. i) PdCl_2_ (2.0 eq), MeOH, rt, 2 h. j) Fm‐PA (2.0 eq), ETT (2.0 eq), CH_2_Cl_2_, rt, 30 min; then mCPBA (6.0 eq), 0 °C, 10 min. k) DBU (4.0 eq), BSTFA (4.0 eq), MeCN, rt, 10 min; then TFA (4.0 eq) in MeOH, rt, 5 min; then Bn‐PA (2.0 eq), ETT (2.0 eq), MeCN, 15 min, rt; then mCPBA (2.0 eq), 0 °C, 10 min. l) H_2_ (30 bar), Pd/C (3.0 eq), NaHCO_3_, tBuOH/H_2_O (4:1), rt, 5 h. Bn‐PA, Bis‐benzyl‐N,N‐diisopropylamino phosphoramidite; BSTFA, N,O‐bis(trimethylsilyl) trifluoroacetamide; DBU, 1,8‐Diazabicyclo[5.4.0]undec‐7‐ene; ETT, 5‐Ethylthio‐1H‐tetrazole; Fm‐PA, Bis(9H‐fluoren‐9‐ylmethyl)‐N,N‐diisopropylamino phosphoramidite.

The synthesis of [4‐OH]‐ and [6‐OH]‐5‐PP‐InsP_4_ (**25a** and **25b**) is derived from the previously described method for [1‐OH]‐ and [3‐OH]‐5‐PP‐InsP_4_ (**13a** and **13b**). First, inositol (**1**) was protected as an orthobenzoate (compound **2**). Unlike the earlier route, desymmetrization occurred at the second step (Figure 3). Here, only one axial hydroxyl group (4‐ or 6‐position) was selectively benzylated with NaH and benzyl bromide, as previously reported in the literature, while the equatorial 2‐OH remained untouched.^[^ ^31^ ^]^ The remaining two hydroxyl groups were subsequently protected with PMB‐groups. This approach generated the racemic mixture, which was further used in the synthesis until resolution at a later stage.

After orthoester cleavage by DIBAL‐H, the resulting racemic acetal **± 17** was subjected to allylation with allyl bromide. Without isolation of intermediate **± 18**, the acetal was cleaved under slightly acidic conditions to furnish racemic diol **± 19**. At this stage, enantiomer separation was achieved using a chiral polysaccharide‐based preparative HPLC column (Chiralpak AD‐H, Daicel). The separation provided both enantiomers **19a** and **19b** in sufficient quantities (∼1 g of each enantiomer), each with an enantiomeric ratio (*e.r*.) > 99:1. From this stage onward, all transformations were performed separately for each enantiomer.

The PMB‐groups were cleaved with TFA without isolation of the intermediates (**20a** and **20b**). The resulting compounds were then phosphorylated with Bn‐phosphoramidite to yield the tetrakisphosphates (**21a** and **21b**). After selective removal of the allyl group by PdCl_2_, the reaction mixture was immediately subjected to a second phosphorylation with Fm‐phosphoramidite, affording the pentakisphosphates (**23a** and **23b**). Pyrophosphorylation under the previously described DBU/BSTFA conditions (vide supra) generated the diphosphates **24a** and **24b**, and a final hydrogenation step removed all protecting groups to yield [4‐OH]‐ and [6‐OH]‐5‐PP‐InsP_4_ (**25a** and **25b**) in enantiomerically pure form.

[4‐OH]‐ and [6‐OH]‐InsP_5_ (**26a** and **26b**) were obtained via hydrogenation of the InsP_5_ intermediates **23a** and **23b** prior to diphosphorylation. These compounds served as references for enantiomer assignment via NMR with a chiral solvating agent and are themselves valuable products. Additionally, an ^18^O‐labeled racemic version of [4/6‐OH]‐5‐PP‐InsP_4_ (compound **S7**) was synthesized as an analytical standard for CE‐MS measurements.^[^ ^30^ ^]^

### Assignment of the Enantiomeric Identity of PP‐InsP_4_ Compounds via ^31^P‐NMR Spectroscopy Using a Chiral Solvating Agent

To assign the enantiomeric identity of the newly synthesized PP‐InsP_4_ compounds, we employed a ^31^P‐NMR method using the chiral solvating agent l‐arginine amide hydrochloride (l‐Arg‐NH_2_).^[^ ^23^, ^24^ ^]^ This approach circumvents the limitations of conventional techniques like optical rotation and X‐ray crystallography, which are often inconclusive for these compounds due to their weak optical activity and poor crystallinity. The absence of commercial PP‐InsP_4_ standards further complicates direct assignments. Therefore, we used commercially available enantiopure InsP_5_ standards as references, enabling indirect assignment via comparison of synthetic InsP_5_ intermediates.

Unlike optical methods, the NMR‐based approach is robust against variations in pH, concentration, and counterions, as analyte and reference are measured under identical conditions. Although NMR alone cannot distinguish enantiomers, excess l‐Arg‐NH_2_ generates diastereomeric ion pairs, causing distinct chemical shifts and enabling enantiomer resolution. Reliable assignment requires addition of the reference compound to the same sample, as chemical shifts in ^31^P‐NMR are highly pH‐dependent and not directly comparable across samples.

Here, we focus on the assignment of [1‐OH]‐ and [3‐OH]‐InsP_5_ (**14a** and **14b**), from which the configuration of [1‐OH]‐ and [3‐OH]‐PP‐InsP_4_ (**13a** and **13b**) was inferred. Assignments of [4‐OH]‐ and [6‐OH]‐InsP_5_ (**26a** and **26b**) are described in the Supporting Information. Each synthetic enantiomer and the commercial [1‐OH]‐InsP_5_ standard were analyzed with l‐Arg‐NH_2_. All samples contained EDTA to enhance resolution. For highly phosphorylated compounds, pH adjustment was not required.

**Figure 4:**
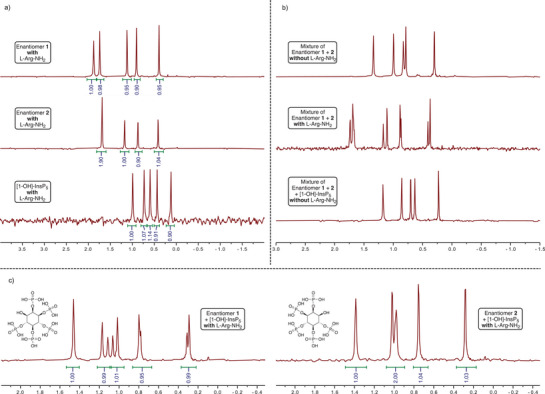
a) ^31^P{^1^H}‐NMR spectra of enantiomers 1 and 2, as well as the commercial [1‐OH]‐InsP_5_, measured in the presence of l‐Arg‐NH_2_. The observation of a single signal set of five phosphates (integrating as 1:1:1:1:1) confirms the enantiomeric purity of the compounds. b) ^31^P{^1^H}‐NMR spectra of a mixture of enantiomers 1 and 2, with and without l‐Arg‐NH_2_. In the absence of the chiral solvating agent, a single signal set is observed as expected for an achiral environment. In the presence of l‐Arg‐NH_2_, two distinct signal sets appear, confirming the mirror image relationship of the parent inositol phosphates. Additionally, the spectrum of a mixture of enantiomers 1 and 2 with the [1‐OH]‐InsP_5_ standard in the absence of l‐Arg‐NH_2_ also shows a single signal set, confirming that the synthetic enantiomeric pair corresponds to [1‐OH]‐ and [3‐OH]‐InsP_5_. C) ^31^P{^1^H}‐NMR spectra of enantiomer 1 spiked with [1‐OH]‐InsP_5_ in the presence of l‐Arg‐NH_2_, showing two distinct signal sets and identifying the enantiomer as [3‐OH]‐InsP_5_ (**14b**). The spectrum of enantiomer 2 spiked with [1‐OH]‐InsP_5_ under the same conditions shows a single signal set, confirming its identity as [1‐OH]‐InsP_5_ (**14a**).

A single signal set in the ^31^P‐NMR spectra confirmed enantiomeric purity. Mixtures of both enantiomers without l‐Arg‐NH_2_ gave one signal set, consistent with their indistinguishability in achiral environments. Addition of the [1‐OH]‐InsP_5_ standard also yielded a single set, confirming the synthetic compounds correspond to [1‐OH]‐ and [3‐OH]‐InsP_5_ (see Figure 4b).

In the presence of l‐Arg‐NH_2_, signal splitting indicated enantiomer differentiation. Spiking each synthetic enantiomer with the standard confirmed identities: the sample showing two signal sets was [3‐OH]‐InsP_5_ (**14a**); the sample with a single set was [1‐OH]‐InsP_5_ (**14b**) (see Figure 4c).

### Application of Novel 5‐PP‐InsP_4_ Compounds as Standards for CE‐MS Analysis of Biological Samples

**Figure 5:**
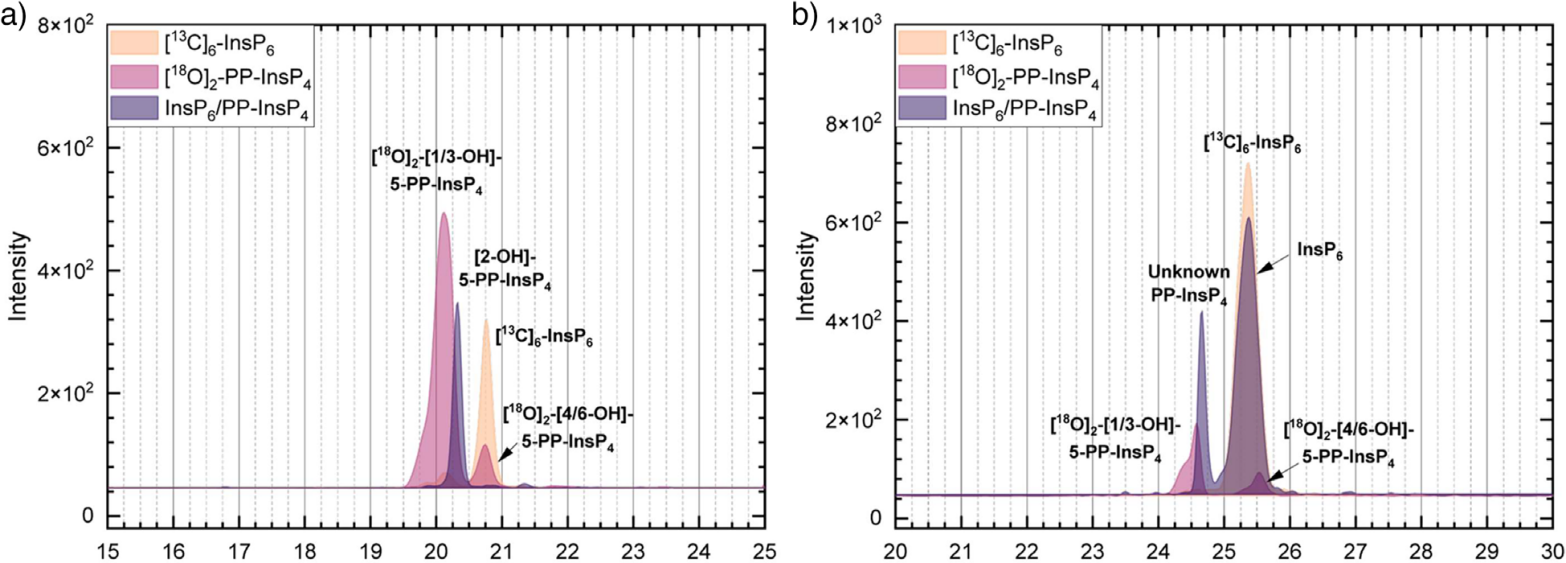
a) CE‐MS analysis of a reference mixture containing the newly synthesized 5‐PP‐InsP_4_ isomers ([1/3‐OH]‐5‐PP‐InsP_4_ and [4/6‐OH]‐5‐PP‐InsP_4_) as [^18^O]_2_‐labeled compounds, along with [^1^ ^3^C]₆‐labeled InsP₆ and unlabeled [2‐OH]‐5‐PP‐InsP_4_. b) CE‐MS analysis of wild‐type Arabidopsis thaliana root extracts under phosphate starvation conditions, spiked with [^18^O]_2_‐labeled [1/3‐OH]‐5‐PP‐InsP_4_ and [4/6‐OH]‐5‐PP‐InsP_4_ as well with [^13^C]_6_‐labeled InsP_6_ as internal standards. The unknown PP‐InsP_4_ peak does not co‐migrate with either isomer, indicating that it is not a 5‐PP‐InsP_4_ isomer. The following m/z values (z = 2) are monitored: PP‐InsP_4_ / InsP_6_ = 328.9; [^13^C]_6_‐InsP_6_ = 331.9; [^18^O]_2_‐PP‐InsP_4_ = 330.0.

To evaluate the suitability of the newly synthesized 5‐PP‐InsP_4_ isomers as standards for CE‐MS, a reference mixture containing all synthesized 5‐PP‐InsP_4_ isomers along with InsP_6_ was prepared and analyzed (see Figure 5a). The reference mixture contained two novel ^18^O labeled references as heavy internal standards (M + 4), unlabeled [2‐OH]‐5‐PP‐InsP_4_ and ^13^C labeled InsP_6_ (M + 6).^[^ ^20^, ^32^, ^33^ ^]^ While most compounds were well separated under the applied conditions (see Supporting Information) that we have usually employed for the analysis of diverse biological samples,^[^ ^8^, ^26^, ^34^ ^]^ no baseline separation for InsP_6_ and [4/6‐OH]‐5‐PP‐InsP_4_ was achieved; they could however be differentiated in the MS experiment due to their different m/z. In biological samples without isotopic labeling, the co‐migration of these peaks with one another under the applied conditions will complicate identification as PP‐InsP_4_ are isobaric with InsP_6_. However, minor variations in migration times between different runs were observed, and in some cases, a small but noticeable shift between InsP_6_ and [4/6‐OH]‐PP‐InsP_4_ was detected, which can be used for further optimization if required.

Riemer et al. previously used CE‐MS to analyze wild‐type *A. thaliana* and rice root extracts under phosphate starvation conditions, detecting an unknown PP‐InsP_4_ peak that behaved contrary to all known inositol pyrophosphates. It increases its concentration under phosphate starvation while being almost undetectable under phosphate sufficiency.^[^ ^7^ ^]^ At the time, only [2‐OH]‐5‐PP‐InsP_4_ was available as a reference, and it was shown that the unknown compound did not correspond to this isomer, both in *Arabidopsis* and rice. However, no standards existed for the other possible 5‐PP‐InsP_4_ isomers. Since [1/3‐OH]‐InsP_5_ is the most abundant InsP_5_ isomer in *Arabidopsis* extracts, we considered it to be a likely precursor for the pyrophosphate.^[^ ^7^ ^]^ Additionally, Whitfield et al. demonstrated that ITPK1 – a kinase known to phosphorylate the 5‐position of InsP_6_ in *Arabidopsis* – produces a product when provided with [6‐OH]‐InsP_5_ as a substrate, suggesting that [6‐OH]‐5‐PP‐InsP_4_ could be the candidate isomer.^[^ ^19^ ^]^ Since it was demonstrated that the uncharacterized PP‐InsP_4_ was absent under phosphate starvation in the *itpk1*‐KO background in *Arabidopsis*, ITPK1's involvement in its synthesis seemed highly likely. These findings provided the rationale to synthesize all 5‐PP‐InsP_4_ isomers as described above to enable the structural assignment of the outlier inositol pyrophosphate.

To investigate whether the unknown PP‐InsP_4_ peak corresponds to one of the synthesized isomers, CE‐MS measurements were performed on samples extracted from *Arabidopsis thaliana* roots prepared under the same phosphate starvation conditions and spiked with [^18^O]_2_‐labeled [1/3‐OH]‐5‐PP‐InsP_4_ (**±13**) and [4/6‐OH]‐5‐PP‐InsP_4_ (**±25**) as internal standards. Strikingly, both isomers (racemic) migrated differently from the unknown PP‐InsP_4_ peak in the sample (see Figure 5b). The close migration of the analyte with [1/3‐OH]‐5‐PP‐InsP_4_ (**±13**) highlights the necessity to generate stable isotope labeled references as otherwise the spiking with an isobaric compound may have led to a misassignment. Since [2‐OH]‐5‐PP‐InsP_4_ had already been excluded in the earlier study, this result demonstrates that the pyrophosphate cannot be located at position 5.

**Figure 6:**
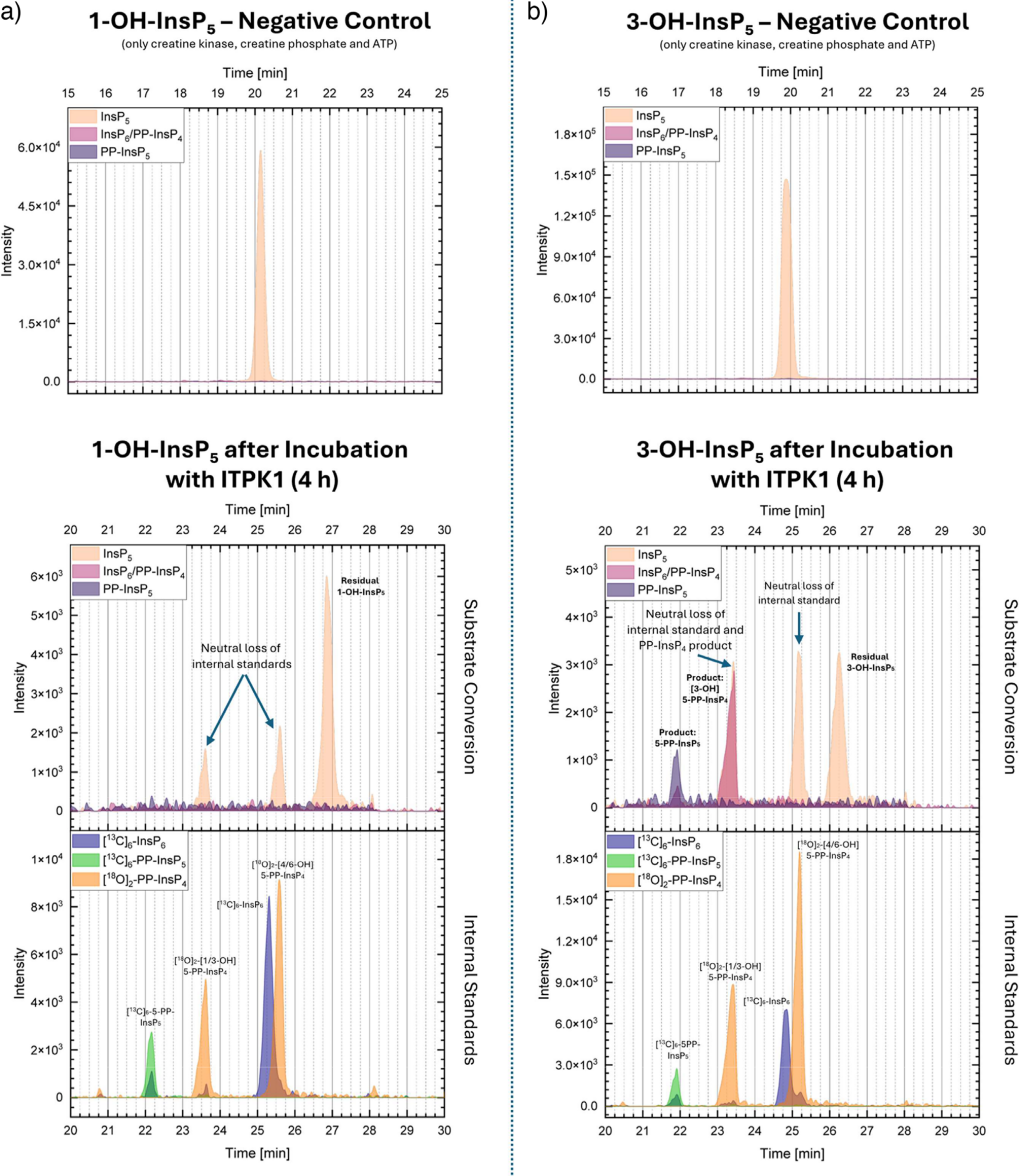
CE‐MS electropherograms illustrating the substrate specificity of Arabidopsis ITPK1 towards different InsP_5_ isomers. For each substrate, two measurements are shown: the upper panel displays the negative control containing InsP_5_, ATP, creatine kinase, and phosphocreatine, but no ITPK1; the lower panel shows the reaction after 4 h incubation with ITPK1. The electropherograms of the reaction control are presented as stacked traces: unlabeled analytes are shown in the upper subpanel, and [^13^C]_6_‐labeled internal standards as well as [^18^O]_2_‐labeled reference compounds in the lower subpanel. The following m/z values (z = 2) are monitored: InsP_5_ = 289.0; PP‐InsP_4_ / InsP_6_ = 328.9; PP‐InsP_5_ = 368.9; [^13^C]_6_‐InsP_6_ = 331.9; [^18^O]_2_‐PP‐InsP_4_ = 330.0; [^13^C]_6_‐PP‐InsP_5_ = 371.9. Apparent signals in specific m/z traces may not exclusively reflect the presence of the corresponding compounds but can result from in‐source fragmentation of PP‐InsP_4_ or PP‐InsP_5_ species via neutral loss of phosphate (∼80 Da), yielding fragment ions that coincide with the m/z of other inositol phosphates such as InsP_5_ or InsP_6_. a) Incubation with [1‐OH]‐InsP_5_ did not result in detectable product formation. b) Incubation with [3‐OH]‐InsP_5_ led to the formation of [3‐OH]‐5‐PP‐InsP_4_ and 5‐PP‐InsP_5_.

**Figure 7:**
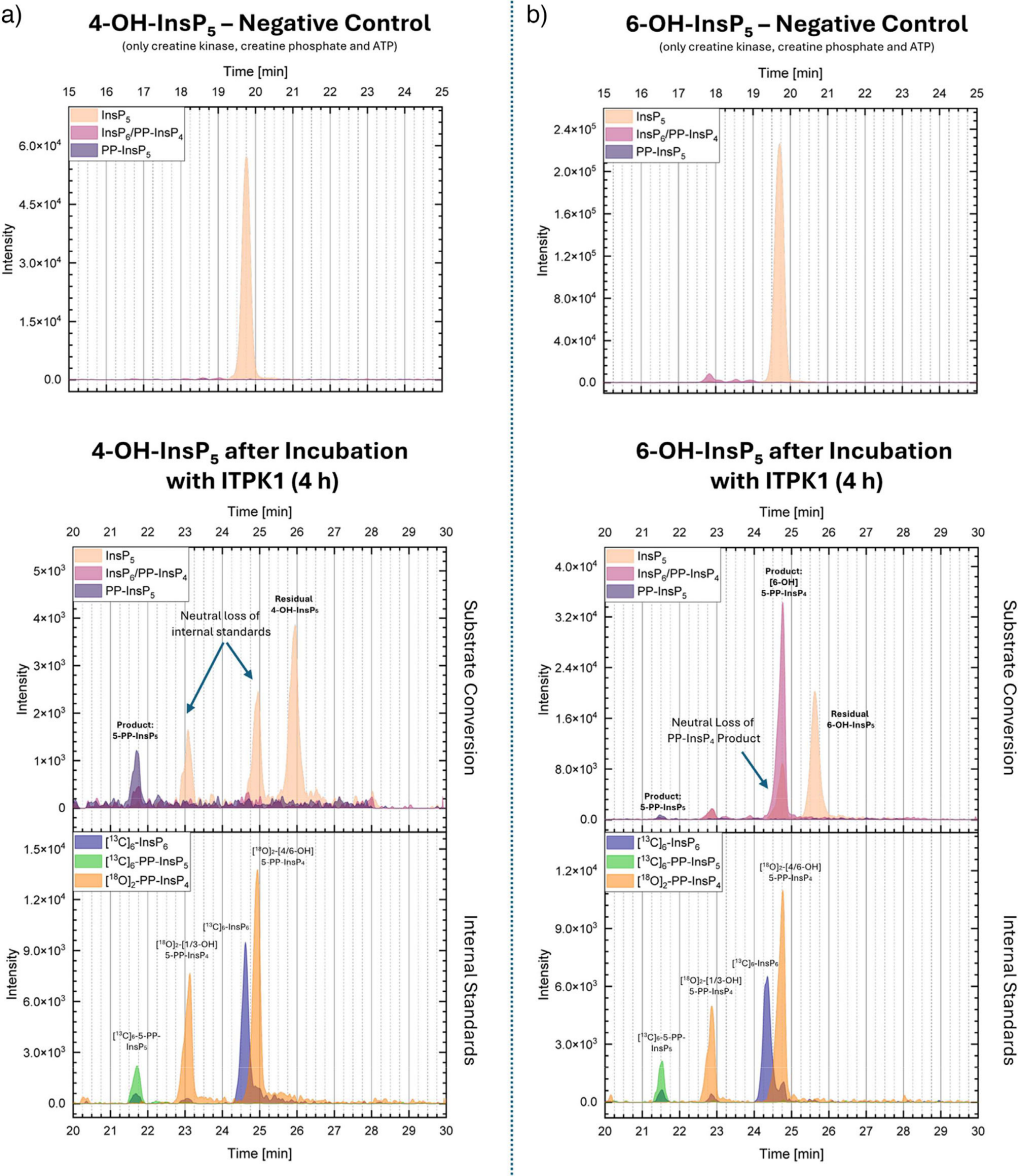
CE‐MS electropherograms illustrating the substrate specificity of Arabidopsis ITPK1 toward different InsP_5_ isomers. For each substrate, two measurements are shown: the upper panel displays the negative control containing InsP_5_, ATP, creatine kinase, and phosphocreatine, but no ITPK1; the lower panel shows the reaction after 4 h incubation with ITPK1. The electropherograms of the reaction control are presented as stacked traces: unlabeled analytes are shown in the upper subpanel, and [^13^C]_6_‐labeled internal standards as well as [^18^O]_2_‐labeled reference compounds in the lower subpanel. The following m/z values (z = 2) are monitored: InsP_5_ = 289.0; PP‐InsP_4_ / InsP_6_ = 328.9; PP‐InsP_5_ = 368.9; [^13^C]_6_‐InsP_6_ = 331.9; [^18^O]_2_‐PP‐InsP_4_ = 330.0; [^13^C]_6_‐PP‐InsP_5_ = 371.9. Apparent signals in specific m/z traces may not exclusively reflect the presence of the corresponding compounds but can result from in‐source fragmentation of PP‐InsP_4_ or PP‐InsP_5_ species via neutral loss of phosphate (∼80 Da), yielding fragment ions that coincide with the m/z of other inositol phosphates such as InsP_5_ or InsP_6_. a) Incubation with [4‐OH]‐InsP_5_ resulted in the formation of 5‐PP‐InsP_5_. b) Incubation with [6‐OH]‐InsP_5_ led to the formation of [6‐OH]‐5‐PP‐InsP_4_ and 5‐PP‐InsP_5_.

To further investigate ITPK1 specificity, we repeated the enzymatic assays previously described by Whitfield et al. under slightly modified conditions and using a differently tagged version of the protein (HIS‐MBP instead of HIS‐tag only, see Supporting Information).^[^ ^19^ ^]^ Our results were largely consistent with their findings: recombinant *Arabidopsis* ITPK1 efficiently phosphorylated [6‐OH]‐InsP_5_ (**26b**) to [6‐OH]‐5‐PP‐InsP_4_ (**25b**) (Figure 7b), while no PP‐InsP_4_ formation was observed for [1‐OH]‐InsP_5_ (**14a**) (see Figure 6a) or [4‐OH]‐InsP_5_ (**26a**) (see Figure 7a).^[^ ^19^ ^]^ However, we observed a significantly higher conversion of [6‐OH]‐InsP_5_ (**26b**) compared to the original study. Notably, and in contrast to their results, we also detected phosphorylation of [3‐OH]‐InsP_5_ (**14b**), yielding [3‐OH]‐5‐PP‐InsP_4_ (**13b**) (see Figure 6b).

Notably, we also observed the formation of 5‐PP‐InsP_5_ in several reactions. This product was most prominent in incubations with [4‐OH]‐InsP_5_ (**26a**), despite the absence of any detectable PP‐InsP_4_ intermediate (Figure 7a), and also appeared in reactions with [3‐OH]‐InsP_5_ (**14b**) (Figure 6b). A small amount of 5‐PP‐InsP_5_ was also observed from [6‐OH]‐InsP_5_ (**26b**) (Figure 7b). Compound identity was confirmed via CE‐MS by co‐migration with a [^13^C]_6_‐labeled 5‐PP‐InsP_5_ reference standard.^[^ ^26^, ^32^ ^]^

To clarify the origin of this product, we tested whether 5‐PP‐InsP_5_ might result from monophosphorylation of PP‐InsP_4_ intermediates. However, incubation of synthetic PP‐InsP_4_ standards with ITPK1 did not lead to detectable formation of 5‐PP‐InsP_5_, ruling out this pathway (see Supporting Information). Additional control experiments with ATP alone or with creatine kinase and phosphocreatine also failed to produce either InsP_6_ or 5‐PP‐InsP_5_, indicating that the reaction requires enzymatic catalysis by ITPK1 (see Figures 6 and 7 and Supporting Information). These results strongly suggest that the observed 5‐PP‐InsP_5_ formation proceeds via an initial phosphorylation of certain InsP_5_ isomers to yield InsP_6_, followed by pyrophosphorylation at the 5‐position. This previously unrecognized activity of ITPK1 was observed specifically with [3‐OH]‐ and [4‐OH]‐InsP_5_ (**14b** and **26a**) while [1‐OH]‐InsP_5_ (**14a**) remained unreactive and [6‐OH]‐InsP_5_ (**26b**) gave only trace amounts of 5‐PP‐InsP_5_.

This implies that ITPK1 may be able to phosphorylate specific InsP_5_ isomers to InsP_6_ in vitro, representing a potential IPK1‐independent route for InsP_6_ biosynthesis. This observation was made under defined in vitro conditions, and its relevance in a cellular context remains to be established. However, since none of the synthesized 5‐PP‐InsP_4_ standards matched the unknown peak detected in Arabidopsis root extracts, ITPK1 is unlikely to be responsible for its generation under phosphate starvation. Consequently, the identity of the enzyme catalyzing this modification has yet to be determined.

## Conclusion

In this study, we have developed a synthetic strategy that enables the enantioselective preparation of all chiral 5‐PP‐InsP_4_ isomers. By employing chiral HPLC separation and a ^31^P‐NMR‐based enantiomer assignment using a chiral solvating agent, we unambiguously determined the absolute configuration of these stereoisomers. The availability of isotopically labeled reference compounds allowed us to reassess the structural identity of a previously detected PP‐InsP_4_ species that accumulates in roots of rice and *A. thaliana* under phosphate starvation. Our CE‐MS analyses show that this unknown compound does not match any of the synthesized 5‐PP‐InsP_4_ isomers, demonstrating that its pyrophosphate group cannot be located at the 5‐position. This suggests that the structural diversity of partially phosphorylated inositol pyrophosphates is significantly greater than previously believed.

Enzymatic assays revealed that *Arabidopsis* ITPK1 selectively phosphorylates [6‐OH]‐InsP_5_ (**26b**) and [3‐OH]‐InsP_5_ (**14b**) to form the corresponding 5‐PP‐InsP_4_ isomers, while [1‐OH]‐InsP_5_ (**14a**) remained unreactive. Unexpectedly, [3‐OH]‐ and [4‐OH]‐InsP_5_ (**14b** and **26a**) gave rise to 5‐PP‐InsP_5_ suggesting that ITPK1 can catalyze both monophosphorylation and pyrophosphorylation steps. A minor amount of 5‐PP‐InsP_5_ was also observed from [6‐OH]‐InsP_5_ (**26b**). These findings refine the current understanding of ITPK1 substrate specificity and reveal a previously unrecognized route for InsP_6_ and PP‐InsP_5_ formation under defined in vitro conditions. While IPK1 has long been considered the sole enzyme catalyzing the conversion of InsP_5_ to InsP_6_ in plants,^[^ ^35^ ^]^ our results demonstrate that ITPK1 can fulfill this function for specific InsP_5_ isomers in vitro. While currently limited to in vitro conditions, this activity may reflect an alternative, IPK1‐independent route to InsP_6_ formation in plants. Moreover, the ability of ITPK1 to catalyze both monophosphorylation and pyrophosphorylation reactions is consistent with its known activity on InsP_4_ and InsP_6_ substrates and highlights its intrinsic substrate promiscuity.^[^ ^19^ ^]^

The findings reported in this study refine our understanding of ITPK1 substrate specificity and suggest that an alternative enzyme is responsible for the formation of the unidentified PP‐InsP_4_ isomer. The enantiopure PP‐InsP_4_ standards developed in this study will serve as valuable tools for future biochemical and enzymatic assays, enabling a deeper study of this underexplored class of inositol pyrophosphates and uncovering their significance in cellular signaling.

## Supporting Information

The authors have cited additional references within the Supporting Information.^[^ ^8^, ^22^, ^26^, ^30^, ^36^, ^37^, ^38^, ^39^, ^40^, ^41^ ^]^

## Conflict of Interests

The authors declare no conflict of interest.

## Supporting information



Supporting Information

## Data Availability

The data that support the findings of this study are available in the Supporting Information of this article.

## References

[1] T. Bittner , C. Wittwer , S. Hauke , D. Wohlwend , S. Mundinger , A. K. Dutta , D. Bezold , T. Dürr , T. Friedrich , C. Schultz , H. J. Jessen , J. Am. Chem. Soc. 2020, 142, 10606–10611. 32459478 10.1021/jacs.0c01697

[2] L. Nagpal , S. He , F. Rao , S. H. Snyder , Annu. Rev. Biochem. 2024, 93, 317–338. 39094034 10.1146/annurev-biochem-030222-121901

[3] S. G. Thota , R. Bhandari , J. Biosci. 2015, 40, 593–605. 26333405 10.1007/s12038-015-9549-x

[4] A. Chakraborty , Biol. Rev. 2018, 93, 1203–1227. 29282838 10.1111/brv.12392 PMC6383672

[5] M. K. Ried , R. Wild , J. Zhu , J. Pipercevic , K. Sturm , L. Broger , R. K. Harmel , L. A. Abriata , L. A. Hothorn , D. Fiedler , S. Hiller , M. Hothorn , Nat. Commun. 2021, 12, 384. 33452263 10.1038/s41467-020-20681-4 PMC7810988

[6] D.‐O. D. Mak , J. K. Foskett , Cell Calcium 2015, 58, 67–78. 25555684 10.1016/j.ceca.2014.12.008 PMC4458407

[7] E. Riemer , D. Qiu , D. Laha , R. K. Harmel , P. Gaugler , V. Gaugler , M. Frei , M. R. Hajirezaei , N. P. Laha , L. Krusenbaum , R. Schneider , A. Saiardi , D. Fiedler , H. J. Jessen , G. Schaaf , R. F. H. Giehl , Mol. Plant 2021, 14, 1864–1880. 34274522 10.1016/j.molp.2021.07.011 PMC8573591

[8] D. Qiu , C. Gu , G. Liu , K. Ritter , V. B. Eisenbeis , T. Bittner , A. Gruzdev , L. Seidel , B. Bengsch , S. B. Shears , H. J. Jessen , Chem. Sci. 2022, 14, 658–667. 36741535 10.1039/d2sc05147h PMC9847636

[9] P. Draškovič , A. Saiardi , R. Bhandari , A. Burton , G. Ilc , M. Kovačevič , S. H. Snyder , M. Podobnik , Chem. Biol. 2008, 15, 274–286. 18355727 10.1016/j.chembiol.2008.01.011

[10] R. Gerasimaite , I. Pavlovic , S. Capolicchio , A. Hofer , A. Schmidt , H. J. Jessen , A. Mayer , ACS Chem. Biol. 2017, 12, 648–653. 28186404 10.1021/acschembio.7b00026

[11] F. S. Menniti , R. N. Miller , J. W. Putney , S. B. Shears , J. Biol. Chem. 1993, 268, 3850–3856. 8382679

[12] M. P. Thomas , B. V. L. Potter , FEBS J. 2014, 281, 14–33. 24152294 10.1111/febs.12575 PMC4063336

[13] H. Wang , C. Gu , R. J. Rolfes , H. J. Jessen , S. B. Shears , J. Biol. Chem. 2018, 293, 6905–6914. 29540476 10.1074/jbc.RA117.001670 PMC5936820

[14] F. Pisani , T. Livermore , G. Rose , J. R. Chubb , M. Gaspari , A. Saiardi , PLoS One 2014, 9, e85533. 24416420 10.1371/journal.pone.0085533 PMC3887064

[15] S. B. Shears , Biochem. J. 2004, 377, 265–280. 14567754 10.1042/BJ20031428 PMC1223885

[16] S. J. York , B. N. Armbruster , P. Greenwell , T. D. Petes , J. D. York , J. Biol. Chem. 2005, 280, 4264–4269. 15561716 10.1074/jbc.M412070200

[17] A. M. Seeds , J. D. York , Biochem. Soc. Symp. 2007, 74, 183–197. 10.1042/BSS0740183 17233590

[18] G. Zong , S. B. Shears , H. Wang , FASEB J. 2022, 36, e22380. 35635723 10.1096/fj.202200393R PMC9202514

[19] H. Whitfield , G. White , C. Sprigg , A. M. Riley , B. V. L. Potter , A. M. Hemmings , C. A. Brearley , Biochem. J. 2020, 477, 2621–2638. 32706850 10.1042/BCJ20200423 PMC7115839

[20] H. Wang , H. Y. Godage , A. M. Riley , J. D. Weaver , S. B. Shears , B. V. L. Potter , Chem. Biol. 2014, 21, 689–699. 24768307 10.1016/j.chembiol.2014.03.009 PMC4085797

[21] S. Capolicchio , H. Wang , D. T. Thakor , S. B. Shears , H. J. Jessen , Angew. Chem. Int. Ed. 2014, 53, 9508–9511. 10.1002/anie.201404398 PMC4153399 25044992

[22] S. Capolicchio , D. T. Thakor , A. Linden , H. J. Jessen , Angew. Chem. Int. Ed. 2013, 52, 6912–6916. 10.1002/anie.201301092 23712702

[23] K. Ritter , N. Jork , A.‐S. Unmüßig , M. Köhn , H. J. Jessen , Biomolecules 2023, 13, 1150. 37509185 10.3390/biom13071150 PMC10377360

[24] D. Blüher , D. Laha , S. Thieme , A. Hofer , L. Eschen‐Lippold , A. Masch , G. Balcke , I. Pavlovic , O. Nagel , A. Schonsky , R. Hinkelmann , J. Wörner , N. Parvin , R. Greiner , S. Weber , A. Tissier , M. Schutkowski , J. Lee , H. Jessen , G. Schaaf , U. Bonas , Nat. Commun. 2017, 8, 2159. 29255246 10.1038/s41467-017-02195-8 PMC5735085

[25] D. Qiu , V. B. Eisenbeis , A. Saiardi , H. J. Jessen , JoVE 2021, e62847. 10.3791/62847 34459823

[26] D. Qiu , M. S. Wilson , V. B. Eisenbeis , R. K. Harmel , E. Riemer , T. M. Haas , C. Wittwer , N. Jork , C. Gu , S. B. Shears , G. Schaaf , B. Kammerer , D. Fiedler , A. Saiardi , H. J. Jessen , Nat. Commun. 2020, 11, 6035. 33247133 10.1038/s41467-020-19928-x PMC7695695

[27] T. M. Haas , S. Mundinger , D. Qiu , N. Jork , K. Ritter , T. Dürr‐Mayer , A. Ripp , A. Saiardi , G. Schaaf , H. J. Jessen , Angew. Chem. Int. Ed. 2022, 61, e202112457. 10.1002/anie.202112457 PMC9298905 34734451

[28] I. H. Gilbert , A. B. Holmes , M. J. Pestchanker , R. C. Young , Carbohydr. Res. 1992, 234, 117–130.

[29] S. J. Conway , J. Gardiner , S. J. A. Grove , M. K. Johns , Z.‐Y. Lim , G. F. Painter , D. E. J. E. Robinson , C. Schieber , J. W. Thuring , L. S.‐M. Wong , M.‐X. Yin , A. W. Burgess , B. Catimel , P. T. Hawkins , N. T. Ktistakis , L. R. Stephens , A. B. Holmes , Org. Biomol. Chem. 2009, 8, 66–76. 20024134 10.1039/b913399b

[30] A. Hofer , G. S. Cremosnik , A. C. Müller , R. Giambruno , C. Trefzer , G. Superti‐Furga , K. L. Bennett , H. J. Jessen , Chem. ‐ Eur. J. 2015, 21, 10116–10122. 26033174 10.1002/chem.201500838

[31] R. Sardessai , S. Krishnaswamy , M. S. Shashidhar , CrystEngComm 2012, 14, 8010.

[32] R. K. Harmel , R. Puschmann , M. Nguyen Trung , A. Saiardi , P. Schmieder , D. Fiedler , Chem. Sci. 2019, 10, 5267–5274. 31191882 10.1039/c9sc00151d PMC6540952

[33] R. Puschmann , R. K. Harmel , D. Fiedler , Biochemistry 2019, 58, 3927–3932. 31461621 10.1021/acs.biochem.9b00587

[34] G. Liu , E. Riemer , R. Schneider , D. Cabuzu , O. Bonny , C. A. Wagner , D. Qiu , A. Saiardi , A. Strauss , T. Lahaye , G. Schaaf , T. Knoll , J. P. Jessen , H. J. Jessen , RSC Chem. Biol. 2023, 4, 300–309. 37034402 10.1039/d2cb00235c PMC10074554

[35] H. Kuo , Y. Hsu , W. Lin , K. Chen , T. Munnik , C. A. Brearley , T. Chiou , Plant J. 2018, 95, 613–630. 10.1111/tpj.13974 29779236

[36] K. Pahnke , C. Meier , ChemBioChem 2017, 18, 1616–1626. 28589630 10.1002/cbic.201700232

[37] H. Y. Godage , A. M. Riley , T. J. Woodman , M. P. Thomas , M. F. Mahon , B. V. L. Potter , J. Org. Chem. 2013, 78, 2275–2288. 23438216 10.1021/jo3027774 PMC3601604

[38] A. Hager , M. Wu , H. Wang , N. W. Brown Jr. , S. B. Shears , N. Veiga , D. Fiedler , Chem. ‐ Eur. J. 2016, 22, 12406–12414. 27460418 10.1002/chem.201601754 PMC5076471

[39] C. Murali , M. S. Shashidhar , C. S. Gopinath , Tetrahedron 2007, 63, 4149–4155.

[40] D. Laha , N. Parvin , A. Hofer , R. F. H. Giehl , N. Fernandez‐Rebollo , N. Von Wirén , A. Saiardi , H. J. Jessen , G. Schaaf , ACS Chem. Biol. 2019, 14, 2127–2133. 31525024 10.1021/acschembio.9b00423

[41] M. A. L. Podeschwa , O. Plettenburg , H.‐J. Altenbach , Eur. J. Org. Chem. 2005, 2005, 3116–3127.

