# Supplementary material for: Expanding the Inositol Pyrophosphate Toolbox: Stereoselective Synthesis and Application of PP‐InsP4 Isomers in Plant Signaling
Source: Angew Chem Int Ed Engl. 2025 Jul 31;64(36):e202507058. doi: 10.1002/anie.202507058 (PMC12402868; doi:10.1002/anie.202507058)

Supporting Information

Expanding the Inositol Pyrophosphate Toolbox: Stereoselective Synthesis and Application of PP-InsP_4 Isomers in Plant Signaling

Kevin Ritter, Anne-Sophie Braun, Guizhen Liu, Mengsi Lu, Verena Gaugler, Gabriel Schaaf, Henning Jacob Jessen*

Inhaltsverzeichnis

1 General Remarks **2**

2 Synthesis of Phosphoramidites **4**

3 Synthesis of enantiopure and ^{18}O-labeled [1-OH]- and [3-OH]-5-PP-InsP$_4$ **5**

4 Synthesis of enantiopure and ^{18}O-labeled [4-OH]- and [6-OH]-5-PP-InsP$_4$ **18**

5 Synthesis of InsP$_5$ Isomers **30**

6 Assignment of the Enantiomeric Identity of PP-InsP$_4$ Compounds via ^{31}P-NMR Spectroscopy using a Chiral Solvating Agent – 4/6-OH-5-PP-InsP$_4$ **33**

7 CE-Measurements & ITPK1 Assay **34**

8 Literature **42**

9 NMR – Spectra **43**

10 Mass Spectra, CE Electropherograms & Chiral HPLC **86**

1 General Remarks

Reactions were carried out using oven-dried glassware under an atmosphere of dry Argon and magnetically stirred, unless noted otherwise. Air- and moisture-sensitive liquids and solutions were transferred via syringe or stainless steel canula.

Reagents were purchased from commercial suppliers (Acros, Sigma-Aldrich, Fluka, TCI, BLDpharm, ChemPur, Alfa Aesar, VWR) and used without further purification, unless noted otherwise.

Solvents were obtained in analytical grade and used as received for extractions, precipitation and solid washings.

Dry solvents for reactions were purchased in a dry form from commercial suppliers (Sigma-Aldrich, Acros, Thermo Scientific) and stored over molecular sieves as well as under the atmosphere of dry Argon.

Deuterated solvents for NMR and reactions were obtained from commercial suppliers (Eurisotope and Deutero) in the indicated purity grade and used as received for NMR spectroscopy.

Thin layer chromatography (TLC) was performed with Merck silica gel 60 F_{254} . Compounds were visually analysed by UV light (λ = 254 and 365 nm) or stained. Staining solutions: $KMnO_4$ stain (1.5 g $KMnO_4$, 10 g Na_2CO_3, 1.25 mL 10% aq. NaOH, 200 mL H_2O), phosphomolybdic acid stain (PMA, 3-4 g $H_3PMo_{12}O_{40}$ in 200 mL EtOH).

Silica column chromatography was carried out using silica gel 60 (0.04 – 0.063 mm, 230 – 400 mesh) from Macherery-Nagel as stationary phase and with non-dry solvents.

Preparative RP-MPLC was performed using a PuriFlash 5.125 by Advion-Interchim. The stationary phase consists of C_{18} reversed-phase (15 µm or 30 µm) silica, prepacked in different column sizes and supplied by Advion-Interchim.

Strong ion-exchange chromatography was performed using an automated Äkta-system. Q-Sepharose was purchased from Sigma-Aldrich. Buffer solutions were produced manually using milliQ H_2O.

Lyophilizations were done with Christ Freeze Dryer Alpha 1-4 LDplus and Christ Freeze Dryer Alpha 1-2 LDplus.

^{1}H-NMR spectra were recorded on a Bruker 400 MHz (with Prodigy CryoProbe) spectrometer in the indicated deuterated solvent. Data are reported as follows: chemical shift (δ, ppm), multiplicity (s, singlet; d, doublet; t, triplet; q, quartet; m, multiplet; br. s, broad singlet), coupling constant(s) (J, Hz), integration. All signals

were referenced to the internal solvent signal as standard (D_2O: δ = 4.79 ppm; MeCN-d_3: δ = 1.94 ppm, $CDCl_3$: δ = 7.26 ppm).

$^{13}C\{^1H\}$-NMR spectra were recorded with ^1H-broadband decoupling on a Bruker 101 MHz (with Prodigy CryoProbe) spectrometers at 298K in the indicated deuterated solvent. All signals were referenced to an internal standard.

$^{31}P\{^1H\}$-NMR spectra and **^{31}P-NMR** spectra were recorded with ^1H-broadband decoupling or ^1H-coupling on a 162 MHz spectrometer (with Prodigy CryoProbe) in the indicated deuterated solvent. All signals were referenced to an internal standard (PPP).

Mass spectra were recorded by C. Warth (Mass spectrometry service of the University of Freiburg) on a Thermo LCQ Advantage [spray voltage: 2.5 – 4.0 kV, spray current: 5 μA, ion transfer tube: 250 (150) °C, evaporation temperature: 50 – 400°C].

Enantiopure $InsP_5$ standards were purchased from SiChem.

2 Synthesis of Phosphoramidites

2.1 Synthesis of Bn-Phosphoramidite **S1**

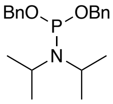

S1

$C_{20}H_{28}NO_2P$
MW: 345,42

The compound was synthesized according to Hofer et al. Analytical data were in accordance with literature.[1]

2.2 Synthesis of Fm-Phosphoramidite **S2**

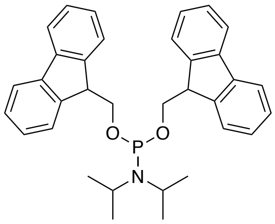

S2

$C_{34}H_{36}NO_2P$
MW: 521,64

The compound was synthesized according to Pahnke et al. Analytical data were in accordance with literature.[2]

2.3 Synthesis of ^{18}O-Labled Bn-Phosphoramidite **S3**

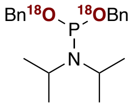

S3

$C_{20}H_{28}N^{18}O_2P$
MW: 349,42

The compound was synthesized according to Hofer et al. Analytical data were in accordance with literature.[1]

3 Synthesis of enantiopure and ^{18}O-labeled [1-OH]- and [3-OH]-5-PP-InsP$_4$

3.1 Synthesis of *myo*-Inositol 1,3,5-Orthobenzoate (2)

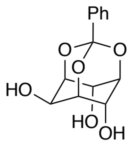

2
$C_{13}H_{14}O_6$
MW: 266,25

The compound was synthesized according to Godage et al. Analytical data were in accordance with literature.[3]

3.2 Synthesis of PMB-Protected Orthobenzoate 3

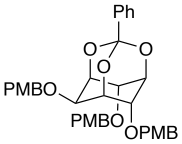

3
$C_{37}H_{38}O_9$
MW: 626,70

The compound was synthesized according to Capolicchio et al. Analytical data were in accordance with literature.[4]

3.3 Synthesis of PMB-Protected Acetal 4

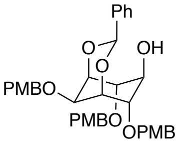

4
$C_{37}H_{40}O_9$
MW: 628,72

PMB-protected orthobenzoate **3** (10.0 g, 16.0 mmol) was dissolved in CH_2Cl_2 (200 mL) and the solution was cooled to -78°C. A solution of DIBAL-H in toluene (1.2 M, 40 mL, 47.9 mmol, 3.0 eq) was added dropwise over 2h. Reaction control was done by TLC (CH/EtOAc 2:1). The solution was warmed to -30°C and the reaction was stopped by careful addition of MeOH (30 mL). The solution was diluted with CH_2Cl_2 (200 mL) and a saturated aqueous solution of Rochelle salt (200 ml) was added. The mixture was stirred for 30 min and the phases were separated. The aqueous phase was extracted with CH_2Cl_2 (2 × 200 mL) and the combined organic phases were washed with water (2 × 250 mL) and brine (300 mL) and dried over $MgSO_4$. The solvent was removed under reduced pressure and crude product was purified by silica column

chromatography (gradient: cyclohexane/EtOAc 3:1 to 2:1). The product **4** (6.36 g, 10.1 mmol, 63%) was obtained as a colorless solid.

Analytical data were consistent with the values reported in literature.[4]

^{1}H-NMR (400 MHz, $CDCl_3$): δ = 7.57 – 7.50 (m, 2H), 7.42 – 7.32 (m, 5H), 7.29 – 7.24 (m, 4H), 6.91 – 6.85 (m, 6H), 5.70 (s, 1H), 4.71 – 4.64 (m, 4H), 4.54 (d, *J* = 11.4 Hz, 2H), 4.37 (d, *J* = 2.4 Hz, 2H), 3.95 (d, *J* = 8.6 Hz, 2H), 3.81 (s, 3H), 3.80 (s, 6H), 3.74 (ddd, *J* = 9.0, 8.2, 2.8 Hz, 1H), 3.58 (t, *J* = 2.4 Hz, 1H), 2.39 (d, *J* = 2.8 Hz, 1H ppm.

3.4 Synthesis of PMB-Protected Diol **6**

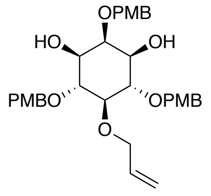

6

$C_{33}H_{40}O_9$
MW: 580,67

PMB-protected acetal **4** (6.36 g, 10.1 mmol) was dissolved in DMF (60 mL) and the solution was cooled to 0°C. Sodium hydride (60% dispersion in oil, 1.01 g, 25.3 mmol, 2.5 eq) was added and the solution was stirred for 30 min. Allyl bromide (1.75 ml, 2.45 g, 20.2 mmol, 2.0 eq) was added and the reaction was stirred overnight and allowed to warm to room temperature. An aqueous solution of LiCl (10%, 150 mL) was added at 0°C and the mixture was extracted with EtOAc (2 × 200 mL). The combined organic phases were washed with water (2 × 200 mL) and brine (200 mL) and dried over $MgSO_4$. The solvent was removed under reduced pressure and the crude product was dissolved in a mixture of MeOH (84 mL) and CH_2Cl_2 (57 mL). The mixture was cooled to 0°C and pTsOH (1.73 g, 9.10 mmol, 0.9 eq) was added. The mixture was stirred for 2h at 0°C and a saturated aqueous solution of $NaHCO_3$ (200 mL) was added. The phases were separated and the aqueous phase was extracted with EtOAc (200 mL). The combined organic phases were washed with water (200 mL) and brine (200 mL) and dried over $MgSO_4$. The solvent was removed under reduced pressure and the crude product was purified by silica column chromatography (cyclohexane/EtOAc 2:1 to 1:1). The product (4.76 g, 8.19 mmol, 81%) was obtained as a colorless oil.

Analytical data were consistent with the values reported in literature.[5]

^{1}H-NMR (400 MHz, $CDCl_3$): δ = 7.33 – 7.28 (m, 4H), 7.25 – 7.21 (m, 2H), 6.92 – 6.86 (m, 6H), 6.01 (ddt, *J* = 17.2, 10.5, 5.5 Hz, 1H), 5.33 (dq, *J* = 17.2, 1.7 Hz, 1H), 5.19 (dq, *J* = 10.4, 1.4 Hz, 1H), 4.83 (d, *J* = 10.7 Hz, 2H), 4.74 – 4.65 (m, 4H), 4.36 (dt, *J* = 5.6, 1.5 Hz, 2H), 3.96 (t, *J* = 2.8 Hz, 1H), 3.83 (s, 3H), 3.81 (s, 6H), 3.70 (t, *J* = 9.5 Hz, 2H), 3.47 (ddd, *J* = 9.7, 5.6, 2.8 Hz, 2H), 3.29 (t, *J* = 9.3 Hz, 1H), 2.22 (dt, *J* = 5.5, 1.4 Hz, 2H) ppm.

3.5 Synthesis of Enantiopure PMB-Protected Alcohol **7a** and **7b**

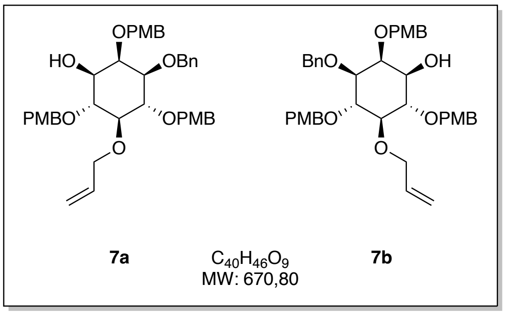

PMB-protected Diol **6** (1.50 g, 2.58 mmol) was dissolved in DMF (15 ml) and cooled to 0°C. Sodium hydride (60% dispersion in oil, 103 mg, 2.58 mmol, 1.0 eq) was added in small portions and the mixture was stirred for 30 min. A solution of benzyl bromide (307 µL, 442 mg, 2.58 mmol, 1.0 eq) in DMF (10 mL) was added dropwise. The reaction was stirred overnight and allowed to warm to room temperature. An aqueous solution of LiCl (10% w/v, 100 mL) was added carefully at 0°C. The mixture was extracted with EtOAc (2 × 150 mL) and the combined organic phases were washed with water (100 mL) and brine (100 mL) and dried over $MgSO_4$. The solvent was removed under reduced pressure and the crude product was purified by silica column chromatography (gradient: cyclohexane/EtOAc 3:1 to 1:1). The title compound **±7** (932 mg, 1.39 mmol, 54%) was obtained as racemic mixture in form of a colorless oil. Separation of the enantiomers was performed on a CHIRALPAK AD-H (250 x 20 mm, 5 µm) semi-preparative HPLC column (isocratic: heptane/ethanol 85:15).

Note: *Higher base equivalents tended to reduce the yield of the desired mono-benzylated product in related substrates.*

Retention times of the enantiomers on a analytical Daicel® CHIRALPAK-AD3 column:
T_R [Enantiomer 1 (**7b**)]: 18.5 min
T_R [Enantiomer 2 (**7a**)]: 32.4 min

Enantiomer assignment was done by generating $InsP_5$ compounds in a later step comparing with commercial standards.

Analytical data for the enantiomeric compounds **7a** and **7b** was identical.
^{1}H-NMR (400 MHz, $CDCl_3$): δ = 7.35 – 7.19 (m, 11H), 6.92 – 6.78 (m, 6H), 6.07 – 5.93 (m, 1H), 5.31 (dq, *J* = 17.2, 1.7 Hz, 1H), 5.18 (dt, *J* = 10.6, 1.6 Hz, 1H), 4.94 – 4.59 (m, 8H), 4.44 – 4.27 (m, 2H), 4.01 – 3.90 (m, 2H), 3.81 (s, 3H), 3.80 (s, 3H), 3.80 (s, 3H), 3.70 (t, *J* = 9.5 Hz, 1H), 3.43 – 3.33 (m, 2H), 3.28 (t, *J* = 9.3 Hz, 1H), 2.14 (d, *J* = 6.2 Hz, 1H) ppm.
^{13}C-NMR (101 MHz, $CDCl_3$): δ = 159.35, 159.20, 138.43, 135.30, 131.02, 130.93, 130.86, 129.82, 129.80, 129.46, 128.41, 127.63, 127.53, 116.52, 113.93, 113.77, 113.74, 83.35, 81.72, 81.59, 81.06, 75.60, 75.16, 74.41, 74.36, 72.97, 72.26, 55.29 ppm.

** One inositol signal is located below the $CDCl_3$ peak and could not be clearly identified in the ^{13}C NMR spectrum. Additionally, the methoxy carbon signals of the PMB groups and one aromatic signal are overlapping. However, these signals were identified using HSQC.*

HRMS (ESI) $[M+NH_4]^+$ calculated for $C_{40}H_{46}O_9$: 688.3480, found 688.3496.

Enantiomer 1(**7b**): **$[\alpha]_D^{20}$** = – 2.4° (C = 1.0 g/100 ml, $CHCl_3$)

Enantiomer 2 (**7a**) **$[\alpha]_D^{20}$** = + 2.3° (C = 1.0 g/100 ml, $CHCl_3$)

3.6 Synthesis of Allyl-Protected $InsP_4$ Derivative **9**

3.6.1 General procedure

PMB-Protected Alcohol **7** was dissolved in $CHCl_3$ and TFA (5% v/v) was added. The reaction mixture was stirred for 1h. Reaction control was done via TLC (DCM/MeOH 95:5). After the reaction was completed cyclohexane (30 ml) and were added. The solvent was removed under reduced pressure. Bn-phosphoramidite **S1** (6.0 eq) was added and the mixture was coevaporated twice with MeCN (3 ml). The mixture was dissolved in DMF and ETT (6.0 eq) was added. The mixture was stirred for 60 min and reaction control was done via ^{31}P-NMR. After the phosphitylation was completed oxidation was done by mCPBA (6.0 eq) at 0°C. The mixture was stirred for 10 min at rt. EtOAc (60 ml) and a solution of LiCl (10%, 50 ml). The phases were separated and the organic phase was washed with water (50 ml) and brine (50 ml) and dried over $MgSO_4$. Celite was added and the solvent was removed under reduced pressure to prepare the crude product as dry load to purify the product by automated reversed phase MPLC (Interchim C_{18}-HP-Column, H_2O/MeCN, gradient: 20 - 100% MeCN). The product was obtained as a colorless oil.

3.6.2 Synthesis of Racemic Allyl-Protected $InsP_4$ Derivative **±9**

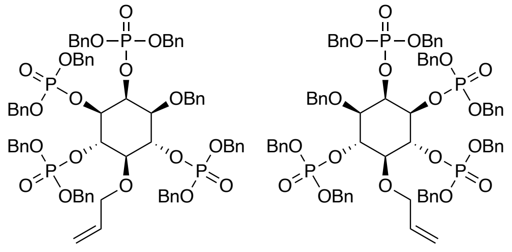

±9

$C_{72}H_{74}O_{18}P_4$
MW: 1351,26

Compound **±9** was synthesized according to general procedure 3.6.1. Inositol derivative **±7** (103 mg, 0.187 mmol) was dissolved in $CHCl_3$ (3.8 ml) and TFA (0.2 ml) was added. Phosphorylation was done in DMF (6 ml) with Bn-phosphoramidite **S1** (388 mg, 1.12 mmol, 6.0 eq) using ETT (146 mg, 1.12 mmol, 6.0 eq) as activator and mCPBA (70%, 277 mg, 1.12 mmol, 6.0 eq) for oxidation. The compound **±9** (144 mg, 0.107 mmol, 57%) was obtained as a colorless oil.

¹H-NMR (400 MHz, $CDCl_3$): δ = 7.48 – 7.41 (m, 2H), 7.41 – 7.12 (m, 43H), 5.90 (ddt, *J* = 17.3, 10.4, 5.7 Hz, 1H), 5.53 (dt, *J* = 9.4, 2.4 Hz, 1H), 5.24 – 4.78 (m, 21H), 4.47 (d, *J* = 10.8 Hz, 1H), 4.41 – 4.29 (m, 3H), 3.55 – 3.46 (m, 2H) ppm.

³¹P {¹H}-NMR (162 MHz, $CDCl_3$) δ = -0.88 (s, 1P), -1.10 (s, 1P), -1.20 (s, 1P), -2.54 (s, 1P) ppm.

¹³C{¹H}-NMR (101 MHz, $CDCl_3$): δ = 136.53, 136.05 – 135.61 (m)*, 134.52, 128.84 – 127.64 (m)*, 117.32, 78.67 – 78.44 (m), 78.25 (d, *J* = 6.8 Hz), 76.93 – 76.78 (m), 75.96 (t, *J* = 2.6 Hz), 74.46 – 74.26 (m), 73.94, 73.74 (d, *J* = 5.9 Hz), 72.16, 70.08 – 69.12 (m)* ppm.

**Due to significant signal overlap, not all expected carbon resonances could be identified. Overlapping signals are listed as multiplets (m) and marked with an asterisk. Missing inositol signals are located below the $CDCl_3$ signal and were observed in HSQC.*

HRMS (ESI) $[M+NH_4]^+$ calculated for $C_{72}H_{74}O_{18}P_4$: 1368.4164, found 1368.4183.

3.6.3 Synthesis of Enantiopure Allyl-protected $InsP_4$ Derivative **9a**

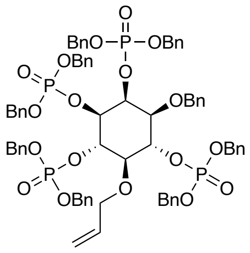

9a

$C_{72}H_{74}O_{18}P_4$
MW: 1351,26

Compound **9a** was synthesized according to general procedure 3.6.1. Inositol derivative **7a** (214 mg, 0.389 mmol) was dissolved in $CHCl_3$ (8.0 ml) and TFA (0.4 ml) was added. Phosphorylation was done in DMF (6 ml) with Bn-phosphoramidite **S1** (805 mg, 2.33 mmol, 6.0 eq) using ETT (304 mg, 2.33 mmol, 6.0 eq) as activator and mCPBA (70%, 575 mg, 2.33 mmol, 6.0 eq) for oxidation. The compound **9a** (341 mg, 0.252 mmol, 65%) was obtained as a colorless oil.

Analytical data was in accordance with racemic $InsP_5$ derivative **±9**.

$[\alpha]_D^{20}$ = + 1.8° (C = 1.0 g/100 ml, $CHCl_3$)

3.6.4 Synthesis of Enantiopure Allyl-protected $InsP_4$ Derivative **9b**

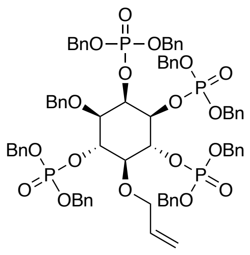

9b

$C_{72}H_{74}O_{18}P_4$
MW: 1351,26

Compound **9b** was synthesized according to general procedure 3.6.1. Inositol derivative **7b** (240 mg, 0.436 mmol) was dissolved in $CHCl_3$ (8.7 ml) and TFA (0.44 ml) was added. Phosphorylation was done in DMF (6 ml) with Bn-phosphoramidite **S1** (903 mg, 2.62 mmol, 6.0 eq) using ETT (340 mg, 2.62 mmol, 6.0 eq) as activator and mCPBA (70%, 645 mg, 2.62 mmol, 6.0 eq) for oxidation. The compound **9b** (311 mg, 0.230 mmol, 53%) was obtained as a colorless oil.

Analytical data was in accordance with racemic $InsP_5$ derivative **±9**.
$[\alpha]_D^{20}$ = – 1.1° (C = 1.0 g/100 ml, $CHCl_3$)

3.7 Synthesis of protected $InsP_5$ Derivative 11

3.7.1 General Procedure

$InsP_4$-derivative **9** was dissolved in MeOH and $PdCl_2$ (2.0 eq) was added. The reaction was stirred for 2h at room temperature. The reaction progress was followed by ^{31}P-NMR. After completion of the reaction the mixture was diluted with EtOAc (50 mL) and washed with a saturated aqueous $NaHCO_3$-solution (50 mL) and brine (50 mL). The organic phase was dried over $MgSO_4$ and the solvent was removed under reduced pressure. The reaction mixture was dissolved in CH_2Cl_2 (50 mL) and Fm-phosphoramidite **S2** (2.0 eq) added. The mixture was coevaporated twice with a mixture of CH_2Cl_2 and MeCN (1:1, 5 mL) and then dissolved in CH_2Cl_2. A solution of ETT in MeCN (2.0 eq) was added and the reaction was stirred for 30 min at room temperature. The reaction progress was followed by ^{31}P-NMR. The mixture was cooled to 0°C and *m*CPBA (2.0 eq) was added. The reaction was stirred for 10 min at room temperature. Celite was added and the solvent was removed under reduced pressure to prepare the crude product as dry load to purify the product by automated reversed phase MPLC MPLC (Interchim C_{18}-HP-Column, H_2O/MeCN, gradient: 20-100% MeCN). The product was obtained as a colorless oil.

3.7.2 Synthesis of Racemic protected $InsP_5$ Derivative ±**11**

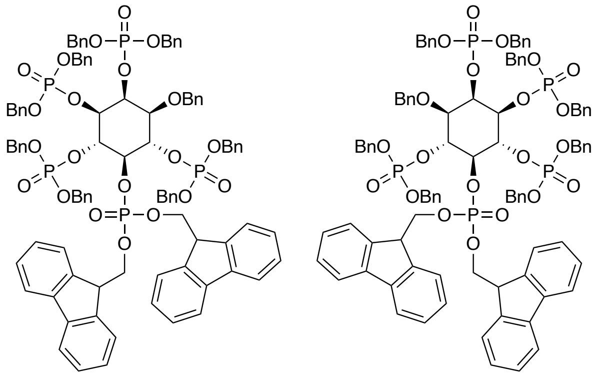

±11

$C_{97}H_{91}O_{21}P_5$
MW: 1747,64

Compound **±11** was synthesized according to general procedure 3.7.1. Inositol derivative **±9** (62.0 mg, 45.9 µmol) dissolved in MeOH (3 mL) and $PdCl_2$ (18.9 mg, 91.8 µmol, 2.0 eq) was added. Phosphorylation was performed in CH_2Cl_2 (3 mL) with Fm-phosphoramidite **S2** (48.0 mg, 92.0 µmol, 2.0 eq) using an ETT-solution (0.5 M, 184 µL, 12.0 mg, 92.0 µmol, 2.0 eq) as activator and mCPBA (70%, 23.0 mg, 92.0 µmol, 2.0 eq) for oxidation. The compound **±11** (47.0 mg, 26.9 µmol, 59%) was obtained as a colorless oil.

^{1}H-NMR (400 MHz, $CDCl_3$): δ = 7.63 – 7.53 (m, 4H), 7.46 – 7.37 (m, 4H), 7.34 – 6.96 (m, 53H), 5.54 (dt, *J* = 9.4, 2.4 Hz, 1H), 5.20 (dd, *J* = 11.8, 6.0 Hz, 1H), 5.15 – 4.69 (m, 18H), 4.55 – 4.43 (m, 2H), 4.39 – 4.30 (m, 1H), 4.26 (ddd, *J* = 9.8, 6.8, 5.4 Hz, 1H), 4.20 – 4.10 (m, 3H), 3.92 (td, *J* = 6.9, 3.3 Hz, 2H), 3.57 (dt, *J* = 9.8, 2.1 Hz, 1H) ppm.

^{31}P {^{1}H}-NMR (162 MHz, $CDCl_3$) δ = -0.93 (s, 1P), -1.43 (s, 1P), -1.76 (s, 1P), -1.80 (s, 1P), -2.20 (s, 1P) ppm.

^{13}C{^{1}H}-NMR (101 MHz, $CDCl_3$): δ = 143.32, 143.28, 143.25, 143.24, 136.43, 136.08 – 135.58 (m)*, 128.98 – 127.37 (m)*, 126.94, 126.93, 126.81, 125.48, 125.45, 125.40, 125.34, 119.70, 119.67, 75.29, 74.99 – 74.74 (m), 74.06 – 73.84 (m), 73.43 (d, *J* = 6.0 Hz), 72.07, 70.12 – 69.14 (m)*, 49.61 – 44.09 (m)* ppm.

** Due to significant signal overlap, not all expected carbon resonances could be identified. Overlapping signals are listed as multiplets (m) and marked with an asterisk. Missing inositol signals are located below the $CDCl_3$ signal and were observed in HSQC.*

HRMS (ESI) $[M+NH_4]^+$ calculated for $C_{97}H_{91}O_{21}P_5$: 1765.5113, found 1765.5142.

3.7.3 Synthesis of Enantiopure protected $InsP_5$ Derivative **11a**

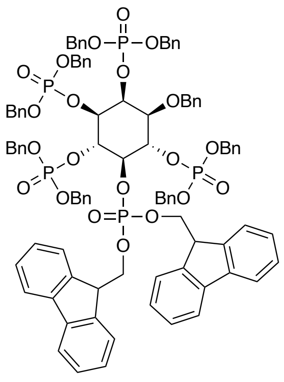

11a

$C_{97}H_{91}O_{21}P_5$
MW: 1747,64

Compound **11a** was synthesized according to general procedure 3.7.1. Inositol derivative **9a** (200 mg, 0.148 mmol) dissolved in MeOH (6 mL) and $PdCl_2$ (58.3 mg, 0.296 mmol, 2.0 eq) was added. Phosphorylation was performed in CH_2Cl_2 (10 mL) with Fm-phosphoramidite **S2** (154 mg, 0.296 mmol, 2.0 eq) using an ETT-solution (0.5M, 592 µL, 39.0 mg, 0.296 mmol, 2.0 eq) as activator and mCPBA (70%, 73.0 mg, 0.296 mmol, 2.0 eq) for oxidation. The compound **11a** (165 mg, 0.944 µmol, 64%) was obtained as a colorless oil.

Analytical data was in accordance with racemic $InsP_5$ derivative **±11**.
$[\alpha]_D^{20}$ = + 3.9° (C = 1.0 g/100 ml, $CHCl_3$)

3.7.4 Synthesis of Enantiopure protected $InsP_5$ Derivative **11b**

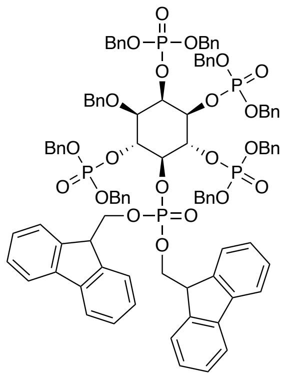

11b

$C_{97}H_{91}O_{21}P_5$
MW: 1747,64

Compound **11b** was synthesized according to general procedure 3.7.1. Inositol derivative **9b** (162 mg, 0.120 mmol) dissolved in MeOH (6 mL) and $PdCl_2$ (47.0 mg, 0.240 mmol, 2.0 eq) was added. Phosphorylation was performed in CH_2Cl_2 (10 mL) with Fm-phosphoramidite **S2** (125 mg, 0.240 mmol, 2.0 eq) using an ETT-solution (0.5M, 480 µL, 31.0 mg, 0.240 mmol, 2.0 eq) as activator and mCPBA (70%, 59.0 mg, 0.240 mmol, 2.0 eq) for oxidation. The compound **11b** (136 mg, 0.778 µmol, 65%) was obtained as a colorless oil.

Analytical data was in accordance with racemic $InsP_5$ derivative **±11**.

$[\alpha]_D^{20}$ = – 4.2° (C = 1.0 g/100 ml, $CHCl_3$)

3.8 Synthesis of PP-$InsP_4$ Derivative **12**

3.8.1 General Procedure

$InsP_5$-derivative **11** was coevaporated with MeCN (2 × 2 mL) and dissolved in MeCN. DBU (4.0 eq) and BSTFA (4.0 eq) were added. The reaction was stirred for 4 min at room temperature. Reaction control was done by ^{31}P-NMR. After completion of the reaction, a solution of TFA (4.0 eq) in MeOH was added and the mixture was stirred for 2 min. The solvent was removed under reduced pressure and the residue was dissolved in MeCN (2 mL). A solution of Bn-phosphoramidite **S1** (2.0 eq) in MeCN and a solution of ETT in MeCN (2.0 eq) were added and the reaction was stirred for 30 min at room temperature. The reaction progress was followed by ^{31}P-NMR. The mixture was cooled to 0°C and *m*CPBA (2.0 eq) was added. The reaction was stirred for 10 min at room temperature. The reaction mixture was directly subjected to an automated reversed phase MPLC (Interchim C_{18}-AQ-Column, H_2O/MeCN with 5% triethylammonium acetate buffer, gradient: 20 - 100% MeCN) to obtain the title compound as a colorless oil.

3.8.2 Synthesis of Racemic ^{18}O-labeled PP-$InsP_4$ Derivative **±12**

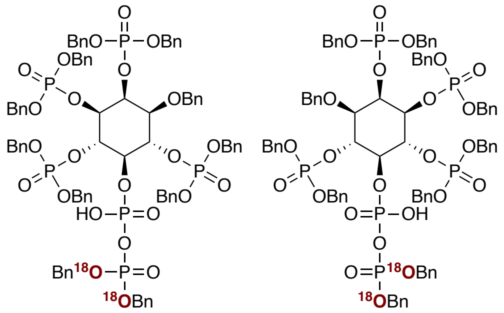

±12

$C_{83}H_{84}O_{22}{}^{18}O_2P_6$
MW: 1655,4039

Compound **±12** was synthesized according to general procedure 3.8.1. Inositol derivative **±11** (50.0 mg, 28.6 µmol) dissolved in MeCN (3 mL) and DBU (17 µL, 17.0 mg, 114 µmol, 4.0 eq) and BSTFA (31 µL, 29.0 mg, 114 µmol, 4.0 eq) were added. A solution of TFA (25%, 9 µL, 13.0 mg, 114 µmol, 4.0 eq) in MeOH (26 µL) was added. Phosphorylation was performed in MeCN (2 mL) with a solution of ^{18}O-labeled Bn-phosphoramidite **S3** (20.0 mg, 60.7 µmol, 2.0 eq) in MeCN (1 mL) using an ETT-solution (1 M, 57 µl, 7.00 mg, 57.2 µmol, 2.0 eq) as activator and mCPBA (70%, 14 mg, 57.2µmol, 2.0 eq) for oxidation. The compound **±12** (46.0 mg, 26.0 µmol, 92 %) was obtained as a colorless oil.

^{1}H-NMR (400 MHz, $CDCl_3$): δ = 7.43 – 7.02 (m, 55H), 5.46 (d, *J* = 9.3 Hz, 1H), 5.30 – 4.79 (m, 22H), 4.77 – 4.52 (m, 2H), 4.51 – 4.25 (m, 2H), 3.44 (d, *J* = 9.7 Hz, 1H) ppm.

^{31}**P {^{1}H}-NMR** (162 MHz, $CDCl_3$) δ = -1.07 (s, 1P), -1.53 (s, 1P), -1.64 (s, 1P), -2.53 (s, 1P), -11.12 (d, *J* = 13.2 Hz, 1P), -13.09 (d, *J* = 13.2 Hz, 1P) ppm.

^{13}**C-NMR** (101 MHz, $CDCl_3$): δ = 137.15 – 135.43 (m)*, 128.88 – 127.29 (m)*, 75.85, 75.77 – 75.40 (m), 74.87 – 74.53 (m), 74.54 – 74.10 (m), 73.79 – 73.16 (m), 72.00, 70.15 – 68.68 (m)* ppm.

** Due to significant signal overlap, not all expected carbon resonances could be identified. Overlapping signals are listed as multiplets (m) and marked with an asterisk. Missing inositol signals are located below the $CDCl_3$ signal and were observed in HSQC.*

HRMS (ESI) $[M-H]^-$ calculated for $C_{83}H_{84}O_{22}{}^{18}O_2P_6$: 1653.3779, found 1653.3761.

3.8.3 Synthesis of Enantiopure PP-InsP_4 Derivative **12a**

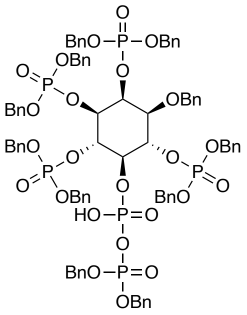

12a

$C_{83}H_{84}O_{24}P_6$
MW: 1651,40

Compound **12a** was synthesized according to general procedure 3.8.1. Inositol derivative **11a** (53.0 mg, 30.3 µmol) dissolved in MeCN (3 mL) and DBU (18 µL, 18.0 mg, 121 µmol, 4.0 eq) and BSTFA (33 µL, 31.0 mg, 121 µmol, 4.0 eq) were added. A solution of TFA (25%, 9 µL, 14.0 mg, 121 µmol, 4.0 eq) in MeOH (28 µL) was added. Phosphorylation was performed in MeCN (2 mL) with a solution of Bn-phosphoramidite **S2** (21.0 mg, 60.7 µmol, 2.0 eq) in MeCN (1 mL) using an ETT-solution (0.5M, 121 µl, 8.00 mg, 60.7 µmol, 2.0 eq) as activator and mCPBA (70%, 15 mg, 60.7 µmol, 2.0 eq) for oxidation. The compound **12a** (40.0 mg, 22.0 µmol, 73 %) was obtained as a colorless oil.

NMR data was in accordance with racemic InsP_5 derivative **±12**.

HRMS (ESI) $[M+NH_4]^+$ calculated for $C_{83}H_{84}O_{24}P_6$: 1669.4150, found 1669.4184.

$[\alpha]_D^{20}$ = + 1.2° (C = 1.0 g/100 ml, $CHCl_3$)

3.8.4 Synthesis of Enantiopure PP-InsP_4 Derivative **12b**

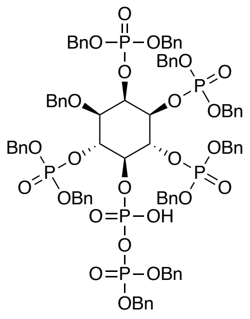

Compound **12b** was synthesized according to general procedure 3.8.1. Inositol derivative **11b** (53.0 mg, 30.3 µmol) dissolved in MeCN (3 mL) and DBU (18 µL, 18.0 mg, 121 µmol, 4.0 eq) and BSTFA (33 µL, 31.0 mg, 121 µmol, 4.0 eq) were added. A solution of TFA (25%, 9 µL, 14.0 mg, 121 µmol, 4.0 eq) in MeOH (28 µL) was added. Phosphorylation was performed in MeCN (2 mL) with a solution of Bn-phosphoramidite **S2** (21.0 mg, 60.7 µmol, 2.0 eq) in MeCN (1 mL) using an ETT-solution (0.5M, 121 µl, 8.00 mg, 60.7 µmol, 2.0 eq) as activator and mCPBA (70%, 15 mg, 60.7 µmol, 2.0 eq) for oxidation. The compound **12b** (43.0 mg, 25.0 µmol, 81 %) was obtained as a colorless oil.

Analytical data was in accordance with racemic InsP_5 derivative **±12** and enantiomer **12a**.
$[\alpha]_D^{20}$ = – 1.0° (C = 1.0 g/100 ml, $CHCl_3$)

3.9 Synthesis of [1-OH]- and [3-OH]-5-PP-InsP_4

3.9.1 General Procedure

PP-InsP_4-derivative **12** was dissolved in a mixture of *t*BuOH and H_2O (3 mL, 4:1). $NaHCO_3$ (11.0 eq) and Pd/C (10% on activated charcoal, 3 eq) were added and the reaction was hydrogenated for 3h at 35 bar (autoclave). The reaction was interrupted and water (2.8 mL) was added to the mixture to ensure dissolution. The reaction was further hydrogenated for an additional 3h at 35 bar (autoclave). The catalyst was removed by filtration through a syringe filter. The filter was rinsed with water (2 mL). The reaction mixture was aliquoted into Eppendorf tubes in equal portions and the solvent was removed by lyophilization. The product **13** was obtained as a colorless solid.

3.9.2 Synthesis of racemic ^{18}O-labeled [1-OH]- and [3-OH]-5-PP-InsP$_4$ (**±13**)

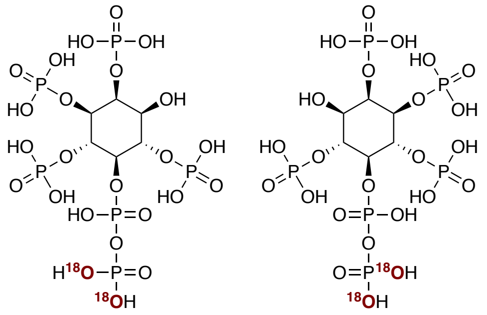

Compound ±**13** was synthesized according to general procedure 3.9.1. Inositol derivative ±**12** (16.0 mg, 9.10 µmol) dissolved in a mixture of *t*BuOH and H_2O and $NaHCO_3$ (8.40 mg, 100 µmol, 11.0 eq) and Pd/C (10% on activated charcoal, 29.1 mg, 27.3 µmol, 3 eq) were added. The compound ±**13** (7.00 mg, 7.73 µmol, 85%) was obtained as a colorless solid.

^{1}H-NMR (400 MHz, D_2O): δ = 4.46 (q, *J* = 9.5 Hz, 1H), 4.34 (q, *J* = 9.3 Hz, 1H), 4.19 (q, *J* = 9.4 Hz, 1H), 4.13 – 4.06 (m, 1H), 3.71 – 3.66 (m, 1H) ppm.

One inositol signal is below the H_2O peak and was observed by HSQC.

^{31}P {^{1}H}-NMR (162 MHz, D_2O) δ = 1.31(s, 1P), 0.58 (s, 1P), 0.44 (s, 1P), 0.22 (s, 1P), -10.51 (d, *J* = 20.1 Hz), -10.94 (d, *J* = 20.1 Hz) ppm.

Resolution was increased by the addition of EDTA.

^{13}C-NMR (101 MHz, D_2O): δ = 77.90, 75.97, 75.97, 75.64, 73.71, 70.49 ppm.

Due to the low amount of compound ^{13}C measurement was not practical. Resonances were assigned by edHSQC.

HRMS (ESI) $[M-2H]^{2-}$ calculated for $C_6H_{18}O_{22}{}^{18}O_2P_6$: 330.9276, found 330.9263.

$[18O]_2$-enrichment observed by CE-MS was greater than 96%.

3.9.3 Synthesis of enantiopure [1-OH]-5-PP-InsP$_4$ (**13a**)

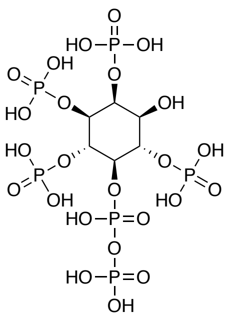

13a

$C_6H_{18}O_{24}P_6$
MW: 660,03

Compound **13a** was synthesized according to general procedure 3.9.1. Inositol derivative **12a** (18.0 mg, 10.9 µmol) dissolved in a mixture of *t*BuOH and H_2O and $NaHCO_3$ (18.1 mg, 216 µmol, 11.0 eq) and Pd/C (10% on activated charcoal, 35.0 mg, 120 µmol, 3 eq) were added. The compound **13a** (9.0 mg, 9.94 µmol, 91%) was obtained as a colorless solid.

NMR data were in accordance with racemic InsP$_5$ derivative **±13**.
HRMS (ESI) $[M-2H]^{2-}$ calculated for $C_6H_{18}O_{24}P_6$: 328.9234, found 328.9239.

3.9.4 Synthesis of enantiopure [3-OH]-5-PP-InsP$_4$ (**13b**)

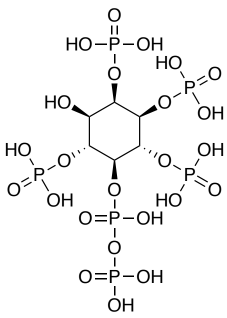

13b

$C_6H_{18}O_{24}P_6$
MW: 660,03

Compound **13b** was synthesized according to general procedure 3.9.1. Inositol derivative **12a** (15.0 mg, 9.10 µmol) dissolved in a mixture of *t*BuOH and H_2O and $NaHCO_3$ (8.40 mg, 100 µmol, 11.0 eq) and Pd/C (10% on activated charcoal, 29.0 mg, 27.3µmol, 3 eq) were added. The compound **13b** (8.0 mg, 8.83 µmol, 97%) was obtained as a colorless solid.

NMR data were in accordance with racemic InsP$_5$ derivative **±13**.
HRMS (ESI) $[M-2H]^{2-}$ calculated for $C_6H_{18}O_{24}P_6$: 328.9234, found 328.9235.

4 Synthesis of enantiopure and ^{18}O-labeled [4-OH]- and [6-OH]-5-PP-InsP$_4$

4.1 Synthesis of Racemic Benzyl-protected Orthobenzoate **±15**

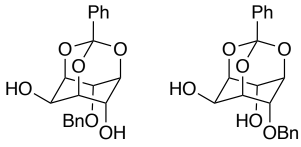

±15

$C_{20}H_{20}O_6$
MW: 356,3740

The compound was synthesized according to Murali et al. Analytical data were in accordance with literature.[6]

4.2 Synthesis of Racemic PMB-protected Orthobenzoate **±16**

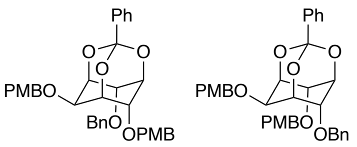

±16

$C_{36}H_{36}O_8$
MW: 596,6760

Benzyl-protected orthobenzoate **±15** (6.91 g, 19.4 mmol) was dissolved in DMF (55 ml) and NaH (60% dispersion in oil, 1.94 g, 48.5 mmol, 2.5 eq) was added at 0°C. The mixture was stirred for 30 min at 0°C. A catalytic amount of TBAI (spatula tip) was added and PMB-chloride (5.79 mL, 6.68 g, 42.7 mmol, 2.2 eq) was added at 0°C. The reaction was stirred for 2h at rt. The reaction was quenched carefully by the addition of a solution of LiCl (10%, 100 mL) and the mixture was diluted with EtOAc (250 mL). The phases were separated and the organic phase was washed with a solution of LiCl (10%, 2 × 100 mL) and dried over $MgSO_4$. The solvent was removed under reduced pressure and the crude product was purified by flash column chromatography (Gradient: cyclohexane/CH_2Cl_2 1:1 to pure CH_2Cl_2) to obtain the product **±16** (10.1 g, 17.0 mmol, 88%) as a colorless solid.

^{1}H-NMR (400 MHz, $CDCl_3$): δ = 7.69 – 7.60 (m, 2H), 7.35 – 7.27 (m, 8H), 7.24 – 7.20 (m, 2H), 7.16 – 7.10 (m, 2H), 6.86 – 6.78 (m, 4H), 4.66 – 4.40 (m, 11H), 4.08 (t, J = 1.6 Hz, 1H), 3.81 (s, 3H), 3.77 (s, 3H) ppm.
^{13}C-NMR (101 MHz, $CDCl_3$): δ = 159.33, 159.31, 137.74, 137.24, 130.15, 129.81, 129.70, 129.36, 129.28, 128.40, 127.93, 127.79, 127.63, 125.42, 113.83, 113.81, 107.88, 74.09, 73.83, 72.08, 71.95, 71.53, 71.33, 70.81, 69.16, 65.66, 55.29, 55.24 ppm.
HRMS (ESI) $[M+H]^+$ calculated for $C_{36}H_{36}O_8$: 597.2483, found 597.2488.

4.3 Synthesis of Racemic PMB-Protected Acetal ±17

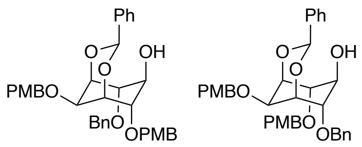

±17

$C_{36}H_{38}O_8$
MW: 598,6920

PMB-protected orthobenzoate **±16** (5.20 g, 8.71 mmol) was dissolved in CH_2Cl_2 (100 mL) and the solution was cooled to -78°C. A solution of DIBAL-H in toluene (1.2M, 21.8 mL, 26.1 mmol, 3.0 eq) was added dropwise over 2h. Reaction control was done by TLC (CH/EtOAc 2:1). The solution was warmed to -30°C and the reaction was stopped by careful addition of MeOH (10 mL). The solution was diluted with CH_2Cl_2 (100 mL) and a saturated aqueous solution of Rochelle salt (100 ml) was added. The mixture was stirred for 30 min and the phases were separated. The aqueous phase was extracted with CH_2Cl_2 (2 × 100 mL) and the combined organic phases were washed with water (2 × 100 mL) and brine (100 mL) and dried over $MgSO_4$. The solvent was removed under reduced pressure and crude product was purified by silica column chromatography (gradient: cyclohexane/EtOAc 4:1 to 3:1). The product **±17** (3.47 g, 5.80 mmol, 67%) was obtained as a colorless solid.

^{1}H-NMR (400 MHz, $CDCl_3$): δ = 7.61 – 7.51 (m, 2H), 7.45 – 7.22 (m, 12H), 6.96 – 6.84 (m, 4H), 5.73 (d, *J* = 2.6 Hz, 1H), 4.82 – 4.52 (m, 6H), 4.40 (t, *J* = 2.5 Hz, 2H), 3.99 (ddd, *J* = 8.2, 5.6, 2.4 Hz, 2H), 3.86 – 3.75 (m, 7H), 3.61 (t, *J* = 2.4 Hz, 1H), 2.66 – 2.53 (m, 1H) ppm.
^{13}C-NMR (101 MHz, $CDCl_3$): δ = 159.54, 159.37, 138.08, 137.54, 129.98, 129.73, 129.51, 129.48, 129.41, 128.59, 128.43, 128.05, 127.98, 126.63, 114.03, 113.94, 92.89, 81.59, 81.11, 73.71, 73.68, 71.79, 71.42, 70.46, 67.86, 55.31 ppm.
** Not all inositol signals could be identified due to signal overlap. Additionally, the methoxy carbon signals of the PMB groups are overlapping. However, these signals were detected in the HSQC spectrum.*
HRMS (ESI) $[M+Na]^+$ calculated for $C_{36}H_{38}O_8$: 621.2459, found 621.2455.

4.4 Synthesis of Enantiopure Allyl-Protected Diol 19a and 19b

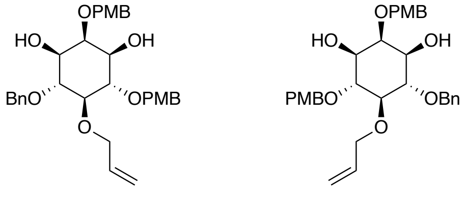

19a $C_{32}H_{38}O_8$ **19b**
MW: 550,65

PMB-protected acetal **±17** (2.95 g, 4.93 mmol) was dissolved in DMF (20 mL) and the solution was cooled to 0°C. Sodium hydride (60% dispersion in oil, 493 mg, 12.3 mmol, 2.5 eq) was added and the solution was

stirred for 30 min. Allyl bromide (0.85 ml, 1.19 g, 9.85 mmol, 2.0 eq) was added and the reaction was stirred for 45 min at rt. An aqueous solution of LiCl (10%, 70 mL) was added at 0°C and the mixture was extracted with EtOAc (2 × 100 mL). The combined organic phases were washed with water (2 × 200 mL) and brine (200 mL) and dried over $MgSO_4$. The solvent was removed under reduced pressure and the crude product was dissolved in a mixture of MeOH (41 mL) and CH_2Cl_2 (28 mL). The mixture was cooled to 0°C and pTsOH (844 mg, 4.43 mmol, 0.9 eq) was added. The mixture was stirred for 2h at 0°C and a saturated aqueous solution of $NaHCO_3$ (50 mL) was added. The phases were separated and the aqueous phase was extracted with EtOAc (3 × 200 mL). The combined organic phases were washed with water (200 mL) and brine (200 mL) and dried over $MgSO_4$. The solvent was removed under reduced pressure and the crude product was purified by silica column chromatography (cyclohexane/EtOAc 2:1 to 1:1). The title compound **±19** (1.97 g, 3.51 mmol, 71%) was obtained as racemic mixture in form of a colorless oil. Separation of the enantiomers was performed on a CHIRALPAK AD-H (250 x 20 mm, 5 µm) semi-preparative HPLC column (isocratic: heptane/ethanol 85:15).

Retention times of the enantiomers on a analytical Daicel® CHIRALPAK-AD3 column:
T_R [Enantiomer 1 (**19a**)]: 27.6 min
T_R [Enantiomer 2 (**19b**)]: 38.9 min

Enantiomer assignment was done by generating $InsP_5$ compounds in a later step comparing with commercial standards.

Analytical data for the enantiomeric compounds **19a** and **19b** were identical.
^{1}H-NMR (400 MHz, $CDCl_3$): δ = 7.41 – 7.33 (m, 4H), 7.33 – 7.29 (m, 3H), 7.26 – 7.21 (m, 2H), 6.93 – 6.85 (m, 4H), 6.00 (ddt, *J* = 17.2, 10.5, 5.6 Hz, 1H), 5.31 (dq, *J* = 17.2, 1.7 Hz, 1H), 5.18 (ddt, *J* = 10.4, 1.9, 1.3 Hz, 1H), 4.94 – 4.81 (m, 2H), 4.78 – 4.66 (m, 4H), 4.37 – 4.33 (m, 2H), 3.96 (t, *J* = 2.8 Hz, 1H), 3.83 (s, 3H), 3.81 (s, 3H), 3.71 (td, *J* = 9.5, 0.8 Hz, 2H), 3.54 – 3.43 (m, 2H), 3.31 (t, *J* = 9.2 Hz, 1H), 2.24 (dd, *J* = 9.5, 5.6 Hz, 2H) ppm.
^{13}C-NMR (101 MHz, $CDCl_3$): δ = 159.42, 159.32, 138.58, 135.07, 130.77, 130.72, 129.82, 129.55, 128.55, 128.12, 127.87, 116.71, 114.00, 113.87, 83.28, 82.29, 81.74, 78.53, 75.56, 75.18, 74.87, 74.20, 72.55, 72.52, 55.30 ppm.
** The methoxy carbon signals of the PMB groups are overlapping. However, these signals were detected in the HSQC spectrum.*
HRMS (ESI) $[M+Na]^+$ calculated for $C_{32}H_{38}O_8Na$: 573.2459, found 573.2449.
Enantiomer 1(**19a**): **$[\alpha]_D^{20}$** = – 7.0° (C = 1.0 g/100 ml, $CHCl_3$)
Enantiomer 2 (**19b**): **$[\alpha]_D^{20}$** = + 8.6° (C = 1.0 g/100 ml, $CHCl_3$)

4.5 Synthesis of Allyl-Protected $InsP_4$ Derivative **21**

4.5.1 General Procedure

PMB-Protected Alcohol **19** was dissolved in $CHCl_3$ and TFA (5% v/v) was added. The reaction mixture was stirred for 2h. Reaction control was done via TLC (DCM/MeOH 95:5). After the reaction was completed, cyclohexane (30 ml) were added. The solvent was removed under reduced pressure. Bn-phosphoramidite **S1** (6.0 eq) was added and the mixture was coevaporated twice with MeCN (3 ml). The mixture was dissolved in DMF and ETT (6.0 eq) was added. The mixture was stirred for 60 min and reaction control was done via ^{31}P-NMR. After the phosphitylation was completed oxidation was done by mCPBA (6.0 eq) at 0°C. The mixture was stirred for 10 min at rt. EtOAc (60 ml) and a solution of LiCl (10%, 50 ml). The phases were separated and the organic phase was washed with water (50 ml) and brine (50 ml) and dried over $MgSO_4$. Celite was added and the solvent was removed under reduced pressure. The crude product was dry-loaded and purified by automated reversed phase MPLC (Interchim C_{18}-HP-Column, H_2O/MeCN, gradient: 20 - 100% MeCN). The product was obtained as a colorless oil.

4.5.2 Synthesis of Racemic Allyl-Protected $InsP_4$ Derivative **±21**

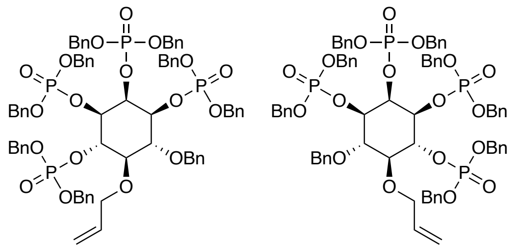

±21

$C_{72}H_{74}O_{18}P_4$
MW: 1351,26

Compound **±21** was synthesized according to general procedure 4.5.1. Inositol derivative **±19** (58.0 mg, 105 µmol) was dissolved in $CHCl_3$ (3.8 ml) and TFA (0.2 ml) was added. Phosphorylation was done in DMF (6 ml) with Bn-phosphoramidite **S1** (218 mg, 632 µmol, 6.0 eq) using ETT (82.0 mg, 632 µmol, 6.0 eq) as activator and mCPBA (70%, 156 mg, 632 µmol, 6.0 eq) for oxidation. The compound **±21** (89.0 mg, 65.0 µmol, 63%) was obtained as a colorless oil.

^{1}H-NMR (400 MHz, $CDCl_3$): δ = 7.38 – 7.11 (m, 45H), 5.84 (ddt, *J* = 17.3, 10.5, 5.6 Hz, 1H), 5.51 (dt, *J* = 8.8, 2.4 Hz, 1H), 5.27 – 4.85 (m, 19H), 4.72 (s, 2H), 4.46 – 4.20 (m, 4H), 3.91 (t, *J* = 9.6 Hz, 1H), 3.39 (t, *J* = 9.4 Hz, 1H) ppm.

^{31}P {^{1}H}-NMR (162 MHz, $CDCl_3$) δ = -0.74 (s, 1P), -1.09 (s, 1P), -1.49 (s, 1P), -2.36 (s,1P) ppm.

^{13}C-NMR (101 MHz, $CDCl_3$): δ = 137.87, 136.22 – 135.37 (m)*, 134.51, 128.76 – 127.26 (m)*, 117.08, 79.85 (d, *J* = 2.1 Hz), 79.19 (d, *J* = 5.0 Hz), 75.74 – 75.44 (m)*, 74.57, 74.00 (q, *J* = 4.4 Hz), 70.13 – 69.10 (m)*. ppm.

** Due to significant signal overlap, not all expected carbon resonances could be identified. Overlapping signals are listed as multiplets (m) and marked with an asterisk. Missing inositol signals are located below the $CDCl_3$ signal and were observed in HSQC.*

HRMS (ESI) $[M+Na]^+$ calculated for $C_{72}H_{74}O_{18}P_4$: 1373.3718, found 1373.3694.

4.5.3 Synthesis of Enantiopure Allyl-Protected $InsP_4$ Derivative **21a**

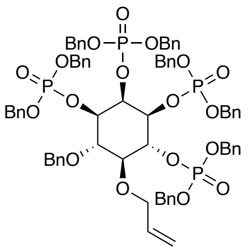

21a

$C_{72}H_{74}O_{18}P_4$
MW: 1351,26

Compound **21a** was synthesized according to general procedure 4.5.1. Inositol derivative **19a** (210 mg, 0.381 mol) was dissolved in $CHCl_3$ (13.4 ml) and TFA (0.67 ml) was added. Phosphorylation was done in DMF (6 ml) with Bn-phosphoramidite **S1** (790 mg, 2.29 mmol, 6.0 eq) using ETT (298 mg, 2.29 mmol, 6.0 eq) as activator and mCPBA (70%, 564 mg, 2.29 mmol, 6.0 eq) for oxidation. The compound **21a** (233 mg, 172 mmol, 45%) was obtained as a colorless oil.

Analytical data was in accordance with racemic $InsP_5$ derivative **±21**
$[\alpha]_D^{20}$ = – 2.2° (C = 1.0 g/100 ml, $CHCl_3$)

4.5.4 Synthesis of Enantiopure Allyl-Protected $InsP_4$ Derivative **21b**

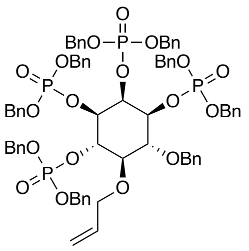

21b

$C_{72}H_{74}O_{18}P_4$
MW: 1351,26

Compound **21b** was synthesized according to general procedure 4.5.1. Inositol derivative **19b** (200 mg, 0.363 mol) was dissolved in $CHCl_3$ (12.7 ml) and TFA (0.64 ml) was added. Phosphorylation was done in DMF (6 ml) with Bn-phosphoramidite **S1** (753 mg, 2.18 mmol, 6.0 eq) using ETT (284 mg, 2.18 mmol, 6.0 eq) as activator and mCPBA (70%, 537 mg, 2.18 mmol, 6.0 eq) for oxidation. The compound **21b** (352 mg, 260 mmol, 72%) was obtained as a colorless oil.

Analytical data were in accordance with racemic $InsP_5$ derivative **±21**
$[\alpha]_D^{20}$ = + 1.6° (C = 1.0 g/100 ml, $CHCl_3$)

4.6 Synthesis of Protected $InsP_5$ Derivative **23**

4.6.1 General Procedure

$InsP_4$-derivative **21** was dissolved in MeOH and $PdCl_2$ (2.0 eq) was added. The reaction was stirred for 2h at room temperature. The reaction progress was followed by ^{31}P-NMR. After completion of the reaction the mixture was diluted with EtOAc (50 mL) and washed with a saturated aqueous $NaHCO_3$-solution (50 mL) and brine (50 mL). The organic phase was dried over $MgSO_4$ and the solvent was removed under reduced pressure. The reaction mixture was dissolved in CH_2Cl_2 (50 mL) and Fm-phosphoramidite **S2** (2.0 eq) added. The mixture was coevaporated twice with a mixture of CH_2Cl_2 and MeCN (1:1, 5 mL) and then dissolved in CH_2Cl_2. A solution of ETT in MeCN (2.0 eq) was added and the reaction was stirred for 30 min at room temperature. The reaction progress was followed by ^{31}P-NMR. The mixture was cooled to 0°C and *m*CPBA (2.0 eq) was added. The reaction was stirred for 10 min at room temperature. Celite was added and the solvent was removed under reduced pressure to prepare the crude product as dry load to purify the product by automated reversed phase MPLC MPLC (Interchim C_{18}-HP-Column, H_2O/MeCN, gradient: 20-100% MeCN). The product was obtained as a colorless oil.

4.6.2 Synthesis of Racemic Protected $InsP_5$ Derivative **±23**

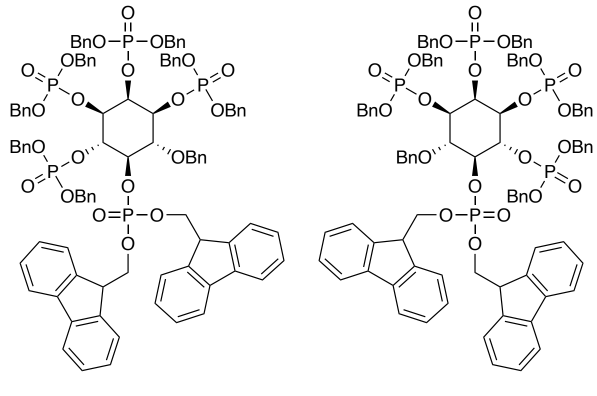

±23

$C_{97}H_{91}O_{21}P_5$
MW: 1747,64

Compound **±23** was synthesized according to general procedure 4.6.1. Inositol derivative **±21** (101 mg, 74.7 µmol) dissolved in MeOH (3 mL) and $PdCl_2$ (29.0 mg, 150 µmol, 2.0 eq) was added. Phosphorylation was performed in CH_2Cl_2 (2 mL) with Fm-phosphoramidite **S2** (77.0 mg, 148 µmol, 2.0 eq) using an ETT-solution (1 M, 148 µL, 19.0 mg, 148 µmol, 2.0 eq) as activator and mCPBA (70%, 36.0 mg, 148 µmol, 2.0 eq) for oxidation. The compound **±23** (77.0 mg, 44.1 µmol, 60%) was obtained as a colorless oil.

^{1}H-NMR (400 MHz, $CDCl_3$): δ = 7.58 (ddt, *J* = 15.5, 7.4, 0.9 Hz, 3H), 7.48 (dt, *J* = 7.5, 0.9 Hz, 1H), 7.39 – 6.93 (m, 57H), 5.56 (dt, *J* = 8.8, 2.3 Hz, 1H), 5.28 – 4.90 (m, 15H), 4.86 – 4.63 (m, 4H), 4.48 – 4.37 (m, 3H), 4.31 (t, *J* = 9.5 Hz, 1H), 4.07 (ddd, *J* = 9.7, 7.2, 5.2 Hz, 1H), 3.94 – 3.86 (m, 2H), 3.83 (t, *J* = 6.6 Hz, 1H), 3.68 (ddd, *J* = 9.6, 8.1, 5.3 Hz, 1H), 3.34 (t, *J* = 7.3 Hz, 1H) ppm.

^{31}P {^{1}H}-NMR (162 MHz, $CDCl_3$) δ = -1.08 (s, 1P), -1.10 (s, 1P), -1.71 (s, 1P), -1.92 (s, 1P), -2.05 (s, 1P) ppm.

^{13}C-NMR (101 MHz, $CDCl_3$): δ = 143.41, 143.38, 143.03, 142.65, 141.24, 141.18, 141.13, 141.05, 137.89, 136.18 – 135.27 (m)*, 128.74 – 126.57 (m)*, 125.36 – 124.90 (m)*, 120.15 – 119.11 (m)*, 78.09 – 77.62 (m), 76.53 – 76.17 (m), 75.52 – 75.23 (m), 74.38, 73.74 – 73.42 (m), 70.28 – 68.87 (m)*, 47.59 (d, J = 8.9 Hz), 47.16 (d, J = 8.9 Hz) ppm.

** Due to significant signal overlap, not all expected carbon resonances could be identified. Overlapping signals are listed as multiplets (m) and marked with an asterisk.*

Missing inositol signal are located below the $CDCl_3$ signal and were observed in HSQC.

HRMS (ESI) $[M+Na]^+$ calculated for $C_{97}H_{91}O_{21}P_5Na$: 1769.4633, found 1769.4606.

4.6.3 Synthesis of Enantiopure Protected $InsP_5$ Derivative **23a**

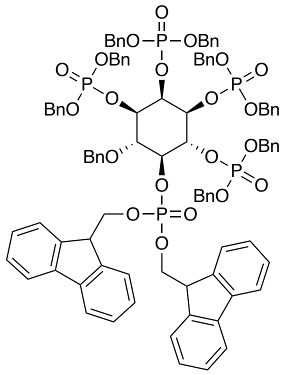

23a

$C_{97}H_{91}O_{21}P_5$
MW: 1747,64

Compound **23a** was synthesized according to general procedure 4.6.1. Inositol derivative **21a** (202 mg, 149 µmol) dissolved in MeOH (6 mL) and $PdCl_2$ (59.0 mg, 299 µmol, 2.0 eq) was added. Phosphorylation was performed in CH_2Cl_2 (10 mL) with Fm-phosphoramidite **S2** (156 mg, 299 µmol, 2.0 eq) using an ETT-solution (0.5 M, 598 µL, 39.0 mg, 299 µmol, 2.0 eq) as activator and mCPBA (70%, 74.0 mg, 299 µmol, 2.0 eq) for oxidation. The compound **23a** (216 mg, 123 µmol, 83%) was obtained as a colorless oil.

Analytical data was in accordance with racemic $InsP_5$ derivative **±23**.

$[\alpha]_D^{20}$ = + 8.0° (C = 1.0 g/100 ml, $CHCl_3$)

4.6.4 Synthesis of Enantiopure Protected $InsP_5$ Derivative **23b**

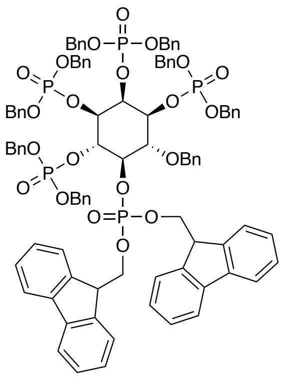

23b

$C_{97}H_{91}O_{21}P_5$
MW: 1747,64

Compound **23b** was synthesized according to general procedure 4.6.1. Inositol derivative **21b** (216 mg, 160 µmol) dissolved in MeOH (5 mL) and $PdCl_2$ (63.0 mg, 320 µmol, 2.0 eq) was added. Phosphorylation was performed in CH_2Cl_2 (10 mL) with Fm-phosphoramidite **S2** (167 mg, 320 µmol, 2.0 eq) using an ETT-solution (0.5 M, 639 µL, 42.0 mg, 320 µmol, 2.0 eq) as activator and mCPBA (70%, 79.0 mg, 320 µmol, 2.0 eq) for oxidation. The compound **23b** (198 mg, 113 µmol, 71%) was obtained as a colorless oil.

Analytical data was in accordance with racemic $InsP_5$ derivative **±23**.
$[\alpha]_D^{20}$ = – 6.0° (C = 1.0 g/100 ml, $CHCl_3$)

4.7 Synthesis of Protected PP-$InsP_4$ Derivative **24**

4.7.1 General Procedure

$InsP_5$-derivative **24** was coevaporated with MeCN (2 × 2 mL) and dissolved in MeCN. DBU (4.0 eq) and BSTFA (4.0 eq) were added. The reaction was stirred for 4 min at room temperature. Reaction control was done by ^{31}P-NMR. After completion of the reaction solution of TFA (4.0 eq) in MeOH was added and the mixture was stirred for 2 min. The solvent was removed under reduced pressure and the residue was dissolved in MeCN (2 mL). A solution of Bn-phosphoramidite **S1** (2.0 eq) in MeCN and a solution of ETT in MeCN (2.0 eq) were added and the reaction was stirred for 30 min at room temperature. The reaction progress was followed by ^{31}P-NMR. The mixture was cooled to 0°C and *m*CPBA (2.0 eq) was added. The reaction was stirred for 10 min at room temperature. The reaction mixture was directly subjected to an automated reversed phase MPLC (Interchim C_{18}-AQ-Column, H_2O/MeCN with 5% triethylammonium acetate buffer, gradient: 20 - 100% MeCN) to obtain the title compound as a colorless oil.

4.7.2 Synthesis of Racemic ^{18}O-labeled Protected PP-InsP$_4$ Derivative **±24**

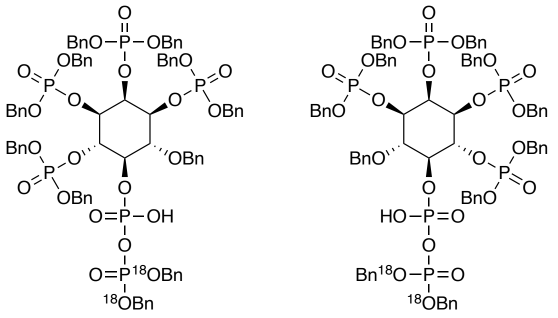

Compound ±**24** was synthesized according to general procedure 3.8.1. Inositol derivative ±**23** (50.0 mg, 28.6 µmol) dissolved in MeCN (3 mL) and DBU (17 µL, 17.0 mg, 114 µmol, 4.0 eq) and BSTFA (31 µL, 29.0 mg, 114 µmol, 4.0 eq) were added. A solution of TFA (25%, 9 µL, 13.0 mg, 114 µmol, 4.0 eq) in MeOH (26 µL) was added. Phosphorylation was performed in MeCN (2 mL) with a solution of ^{18}O-labeled Bn-phosphoramidite **S3** (20.0 mg, 57.2 µmol, 2.0 eq) in MeCN (1 mL) using an ETT-solution (0.5 м, 114 µl, 7.00 mg, 57.2 µmol, 2.0 eq) as activator and mCPBA (70%, 14 mg, 57.2 µmol, 2.0 eq) for oxidation. The compound ±**24** (47.0 mg, 26.0 µmol, 91 %) was obtained as a colorless oil.

^{1}H-NMR (400 MHz, CDCl$_3$): δ = 7.46 – 7.40 (m, 2H), 7.34 – 7.06 (m, 51H), 7.05 – 6.97 (m, 2H), 5.52 (dt, *J* = 8.8, 2.5 Hz, 1H), 5.21 (ddd, *J* = 11.0, 6.9, 3.6 Hz, 2H), 5.15 – 4.85 (m, 19H), 4.76 – 4.63 (m, 2H), 4.60 (d, *J* = 11.2 Hz, 1H), 4.57 – 4.44 (m, 2H), 3.98 (t, *J* = 9.3 Hz, 1H) ppm.

^{31}P {^{1}H}-NMR (162 MHz, CDCl$_3$) δ = -1.10 (s, 1P), -1.48 (s, 1P), -1.86 (s, 1P), -2.38 (s, 1P), -11.46 (d, *J* = 11.7 Hz, 1P), -13.30 (d, *J* = 11.4 Hz, 1P) ppm.

^{13}C-NMR (101 MHz, CDCl$_3$): δ = 138.66, 137.06 – 135.43 (m)*, 128.72 – 127.37 (m)*, 126.99, 78.58 – 78.03 (m), 76.49 – 76.30 (m), 76.30 – 76.18 (m), 76.06 – 75.68 (m), 75.31 – 75.00 (m), 74.76, 74.06 – 73.69 (m), 70.23 – 68.84 (m)* ppm.

** Due to significant signal overlap, not all expected carbon resonances could be identified. Overlapping signals are listed as multiplets (m) and marked with an asterisk.*

Missing inositol signals are located below the CDCl$_3$ signal and were observed in HSQC.

HRMS (ESI) [M-H]$^-$ calculated for $C_{83}H_{84}O_{22}{}^{18}O_2P_6$: 1653.3779, found 1653.3752.

4.7.3 Synthesis of Enantiopure Protected PP-InsP_4 Derivative **24a**

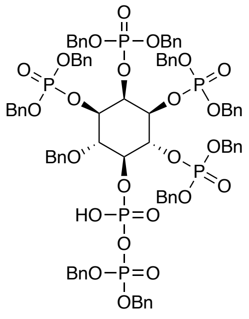

24a

$C_{83}H_{84}O_{24}P_6$
MW: 1651,40

Compound **24a** was synthesized according to general procedure 3.8.1. Inositol derivative **23a** (50.0 mg, 28.6 µmol) dissolved in MeCN (3 mL) and DBU (17 µL, 17.0 mg, 114 µmol, 4.0 eq) and BSTFA (31 µL, 29.0 mg, 114 µmol, 4.0 eq) were added. A solution of TFA (25%, 9 µL, 13.0 mg, 114 µmol, 4.0 eq) in MeOH (26 µL) was added. Phosphorylation was performed in MeCN (2 mL) with a solution of Bn-phosphoramidite **S1** (20.0 mg, 57.2 µmol, 2.0 eq) in MeCN (1 mL) using an ETT-solution (0.5 M, 114 µl, 7.00 mg, 57.2 µmol, 2.0 eq) as activator and mCPBA (70%, 14 mg, 57.2 µmol, 2.0 eq) for oxidation. The compound **24a** (44.0 mg, 24.0 µmol, 85 %) was obtained as a colorless oil.

NMR data was in accordance with racemic InsP_5 derivative **±24**.

HRMS (ESI) $[M-H]^-$ calculated for $C_{83}H_{84}O_{24}P_6$: 1649.3705, found 1649.3679.

$[\alpha]_D^{20}$ = + 1.1° (C = 1.0 g/100 ml, $CHCl_3$)

4.7.4 Synthesis of Enantiopure Protected PP-InsP_4 Derivative **24b**

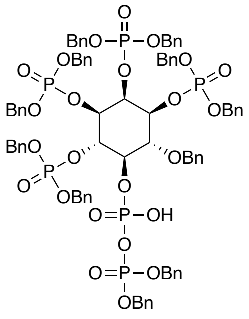

24b

$C_{83}H_{84}O_{24}P_6$
MW: 1651,40

Compound **24b** was synthesized according to general procedure 3.8.1. Inositol derivative **23b** (50.0 mg, 28.6 µmol) dissolved in MeCN (3 mL) and DBU (17 µL, 17.0 mg, 114 µmol, 4.0 eq) and BSTFA (31 µL, 29.0 mg, 114 µmol, 4.0 eq) were added. A solution of TFA (25%, 9 µL, 13.0 mg, 114 µmol, 4.0 eq) in MeOH (26 µL) was added. Phosphorylation was performed in MeCN (2 mL) with a solution of Bn-phosphoramidite **S1** (20.0 mg, 57.2 µmol, 2.0 eq) in MeCN (1 mL) using an ETT-solution (0.5 M, 114 µl, 7.00 mg, 57.2 µmol, 2.0 eq) as

activator and mCPBA (70%, 14 mg, 57.2 µmol, 2.0 eq) for oxidation. The compound **24b** (46.0 mg, 26.0 µmol, 89 %) was obtained as a colorless oil.

Analytical data was in accordance with racemic $InsP_5$ derivative **±24** and enantiomer **24a**.
$[\alpha]_D^{20}$ = – 1.2° (C = 1.0 g/100 ml, $CHCl_3$)

4.8 Synthesis of Enantiopure [4-OH]- and [6-OH]-5-PP-$InsP_4$ **(25)**

4.8.1 General Procedure

PP-$InsP_4$-derivative **24** was dissolved in a mixture of tBuOH and H_2O (3 mL, 4:1). $NaHCO_3$ (11.0 eq) and Pd/C (10% on activated charcoal, 3 eq) were added and the reaction was hydrogenated 3h at 35 bar (Autoclave). The reaction was interrupted and water (2.8 mL) was added to the mixture. The reaction was hydrogenated for additional 3h at 35 bar (Autoclave). The catalyst was removed by filtration trough a syringe filter. The filter was rinsed with water (2 ml). The reaction mixture was aliquoted into Eppendorf tubes in equal portions and the solvent was removed by lyophilization. The product **25** was obtained as a colorless solid.

4.8.2 Synthesis of Racemic ^{18}O-labeled [4-OH]- and [6-OH]-5-PP-$InsP_4$ **(±25)**

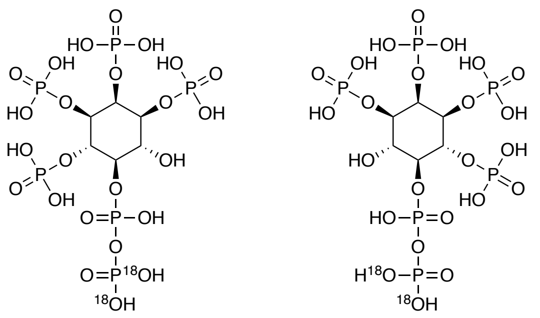

±25
$C_6H_{18}O_{22}{}^{18}O_2P_6$
MW: 664,0289

Compound ±**25** was synthesized according to general procedure 3.9.1. Inositol derivative ±**24** (13.0 mg, 7.90 µmol) dissolved in a mixture of tBuOH and H_2O and $NaHCO_3$ (7.30 mg, 86.6 µmol, 11.0 eq) and Pd/C (10% on activated charcoal, 25.1 mg, 23.6 µmol, 3 eq) were added. The compound ±**25** (7.0 mg, 7.73 µmol, 98%) was obtained as a colorless solid.

^{1}H-NMR (400 MHz, D_2O): δ = 4.89 – 4.82 (m, 1H), 4.45 (q, *J* = 9.7 Hz, 1H), 4.19 – 4.05 (m, 3H), 4.01 – 3.93 (m, 1H) ppm.

^{31}P {^{1}H}-NMR (162 MHz, D_2O) δ = 0.75 (s, 1P), 0.41 (s,1P), 0.17(s, 1P), -0.02 (s, 1P), -9.33 (d, *J* = 20.1 Hz), -10.23 (d, *J* = 20.1 Hz) ppm. *Resolution was increased by the addition of EDTA.*

^{13}C-NMR (101 MHz, D_2O): δ = 79.51, 75.64, 75.00, 73.39, 73.39, 70.17 ppm.

Due to the low amount of compound ^{13}C measurement was not feasible. Peaks were assigned by edHSQC.

HRMS (ESI) $[M-2H]^{2-}$ calculated for $C_6H_{18}O_{22}{}^{18}O_2P_6$: 330.9276, found 330.9281.

$[18O]_2$-enrichment observed by CE-MS was greater than 96%.

4.8.3 Synthesis of Enantiopure [4-OH]-5-PP-InsP$_4$ (**25a**)

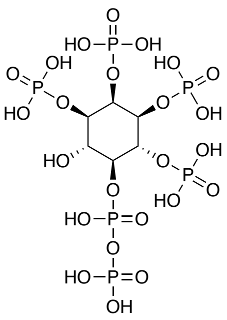

25a

$C_6H_{18}O_{24}P_6$
MW: 660,0286

Compound **25a** was synthesized according to general procedure 3.9.1. Inositol derivative **24a** (16.0 mg, 9.70 µmol) dissolved in a mixture of tBuOH and H_2O and $NaHCO_3$ (9.00 mg, 106.6 µmol, 11.0 eq) and Pd/C (10% on activated charcoal, 31.0 mg, 29.1 µmol, 3 eq) were added. The compound **25a** (8.0 mg, 8.83 µmol, 91%) was obtained as a colorless solid.

NMR data was in accordance with racemic InsP$_5$ derivative **±25**.
HRMS (ESI) $[M-2H]^{2-}$ calculated for $C_6H_{18}O_{24}P_6$: 328.9234, found 328.9234.

4.8.4 Synthesis of Enantiopure [6-OH]-5-PP-InsP$_4$ (**25b**)

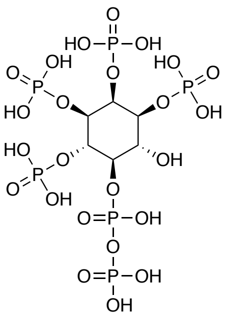

25b

$C_6H_{18}O_{24}P_6$
MW: 660,0286

Compound **25b** was synthesized according to general procedure 3.9.1. Inositol derivative **24a** (17.0 mg, 10.3 µmol) dissolved in a mixture of tBuOH and H_2O and $NaHCO_3$ (9.50 mg, 113 µmol, 11.0 eq) and Pd/C (10% on activated charcoal, 32.9 mg, 30.9 µmol, 3 eq) were added. The compound **24a** (9.0 mg, 9.94 µmol, 96%) was obtained as a colorless solid.

NMR data was in accordance with racemic InsP$_5$ derivative **±25**.
HRMS (ESI) $[M-2H]^{2-}$ calculated for $C_6H_{18}O_{24}P_6$: 328.9234, found 328.9237.

5 Synthesis of $InsP_5$ Isomers

5.1 General Procedure

$InsP_5$-derivative **11** or **23** was dissolved in a mixture of *t*BuOH and H_2O (3 mL, 4:1). $NaHCO_3$ (10.0 eq) and Pd/C (10% on activated charcoal, 3 eq) were added and the reaction was hydrogenated 3h at 35 bar (autoclave). The reaction was interrupted and water (2.8 mL) was added to the mixture to ensure solubility. The reaction was hydrogenated for additional 3h at 35 bar (autoclave). The catalyst was removed by filtration through a syringe filter. The filter was rinsed with water (2 mL). The reaction mixture was aliquoted into Eppendorf tubes in equal portions and the solvent was removed by lyophilization. The product **14** or **26** was obtained as a colorless solid.

5.2 Synthesis of Enantiopure [1-OH]-$InsP_5$ **(14a)**

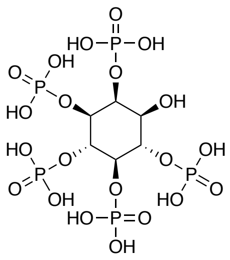

14a

$C_6H_{17}O_{21}P_5$
MW: 580,05

Compound **14a** was synthesized according to general procedure 3.9.1. Inositol derivative **11a** (28.0 mg, 16.0 µmol) dissolved in a mixture of *t*BuOH and H_2O and $NaHCO_3$ (14.8 mg, 176 µmol, 11.0 eq) and Pd/C (10% on activated charcoal, 51.1 mg, 48.1 µmol, 3 eq) were added. The compound **14a** (14.0 mg, 15.5 µmol, 96%) was obtained as a colorless solid.

Analytical data was in accordance with commercial [1-OH]-$InsP_5$ as reference compound and with literature.[7]

5.3 Synthesis of Enantiopure [3-OH]-$InsP_5$ **(14b)**

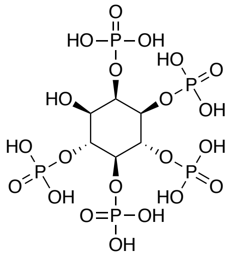

14b

$C_6H_{17}O_{21}P_5$
MW: 580,05

Compound **14b** was synthesized according to general procedure 3.9.1. Inositol derivative **11b** (39.0 mg, 22.3 µmol) dissolved in a mixture of *t*BuOH and H_2O and $NaHCO_3$ (20.6 mg, 246 µmol, 11.0 eq) and Pd/C (10% on activated charcoal, 71.2 mg, 66.9 µmol, 3 eq) were added. The compound **14b** (18.0 mg, 19.9 µmol, 89%) was obtained as a acolorless solid.

Analytical data was in accordance with commercial [1-OH]-InsP_5 as reference compound and with literature.[7]

5.4 Synthesis of Enantiopure [4-OH]-InsP_5 **(26a)**

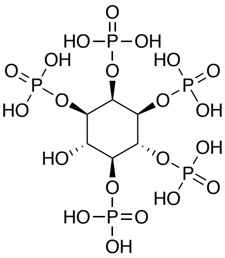

26a

$C_6H_{17}O_{21}P_5$
MW: 580,05

Compound **26a** was synthesized according to general procedure 3.9.1. Inositol derivative **23a** (32.0 mg, 18.2 µmol) dissolved in a mixture of tBuOH and H_2O and $NaHCO_3$ (16.8 mg, 200 µmol, 11.0 eq) and Pd/C (10% on activated charcoal, 58.2 mg, 54.7 µmol, 3 eq) were added. The compound **26a** (16.0 mg, 17.7 µmol, 97%) was obtained as a colorless solid.

Analytical data was in accordance with commercial [6-OH]-InsP_5 as reference compound and with literature.[7]

5.5 Synthesis of Enantiopure [6-OH]-InsP_5 **(26b)**

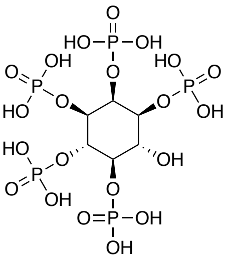

26b

$C_6H_{17}O_{21}P_5$
MW: 580,05

Compound **26b** was synthesized according to general procedure 3.9.1. Inositol derivative **23b** (37.0 mg, 21.1 µmol) dissolved in a mixture of tBuOH and H_2O and $NaHCO_3$ (19.5 mg, 232 µmol, 11.0 eq) and Pd/C

(10% on activated charcoal, 67.2 mg, 63.2µmol, 3 eq) were added. The compound **26b** (17.0 mg, 18.8 µmol, 89%) was obtained as a colorless solid.

Analytical data was in accordance with commercial [6-OH]-InsP_5 as reference compound and with literature.[7]

6 Assignment of the Enantiomeric Identity of PP-InsP$_4$ Compounds via ^{31}P-NMR Spectroscopy using a Chiral Solvating Agent – 4/6-OH-5-PP-InsP$_4$

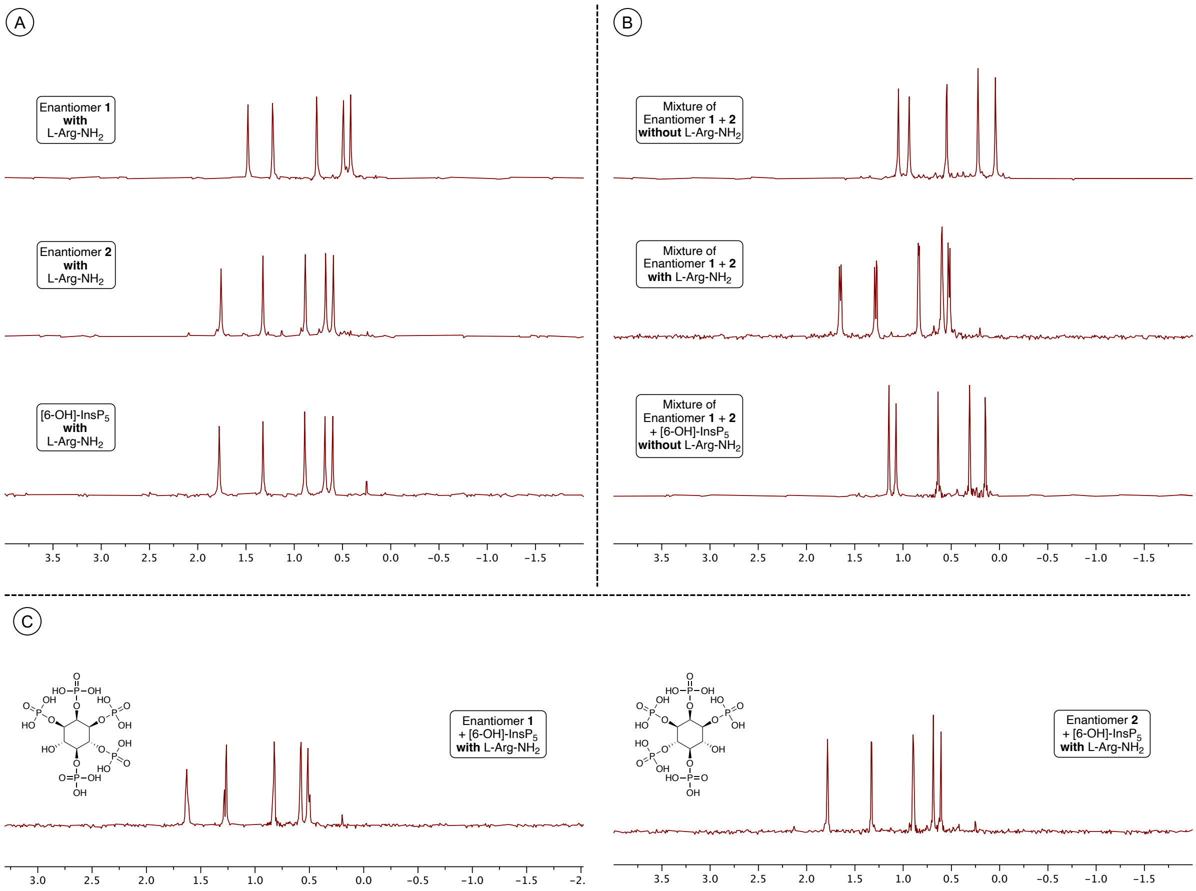

Figure S1: ^{31}P{^{1}H}-NMR spectra of Enantiomers 1 and 2, as well as the commercial [6-OH]-InsP$_5$, measured in the presence of L-Arg-NH$_2$. The observation of a single signal set for each confirms the enantiomeric purity of the compounds. **(b)** ^{31}P{^{1}H}-NMR spectra of a mixture of enantiomers 1 and 2, with and without L-Arg-NH$_2$. In the absence of the chiral solvating agent, a single signal set is observed, indicating indistinguishability in an achiral environment. In the presence of L-Arg-NH$_2$, two distinct signal sets appear, confirming their enantiomeric relationship. Additionally, the spectrum of a mixture of enantiomers 1 and 2 with the [6-OH]-InsP$_5$ standard in the absence of L-Arg-NH$_2$ also shows a single signal set, confirming that the synthesized enantiomeric pair corresponds to [4-OH]- and [6-OH]-InsP$_5$. **(c)** ^{31}P{^{1}H}-NMR spectra of enantiomer 1 spiked with [6-OH]-InsP$_5$ in the presence of L-Arg-NH$_2$, showing two distinct signal sets and identifying the enantiomer as [4-OH]-InsP$_5$ (**13a**). The spectrum of enantiomer 2 spiked with [6-OH]-InsP$_5$ under the same conditions shows a single signal set, confirming its identity as [6-OH]-InsP$_5$ (**13b**).

7 CE-Measurements & ITPK1 Assay

7.1 ITPK1 Expression & Purification

The His_8-MBP-ITPK1 fusion protein was expressed in *Escherichia coli* BL21 CodonPlus (DE3) RIL cells (Stratagene).[8] Bacterial cultures were grown overnight at 37 °C with continuous shaking at 200 rpm, then diluted 1:1000 into fresh 2YT medium containing 50 mg/L kanamycin and 25 mg/L chloramphenicol. The cells were incubated at 37 °C while shaking at 200 rpm until the optical density at 600 nm (OD600) reached approximately 0.6. Protein expression was induced by adding 500 µM IPTG (isopropyl-β-D-1-thiogalactopyranoside), and the cultures were further incubated overnight at 16 °C. Cell lysis was performed using glass beads (Ø 0.1 mm) in a lysis buffer containing 300 mM NaCl, 20 mM Na_2HPO_4, 2 mM DTT, pH 7.4 and EDTA-free complete ULTRA protease inhibitor cocktail (Roche). Protein purification was carried out in batch mode using Ni-NTA agarose resin (Macherey-Nagel), and the protein was eluted in the lysis buffer containing 250 mM imidazole. The eluate was dialyzed using Slide-A-Lyzer Dialysis Cassettes (Thermo Scientific) according to the manufacturer's instructions and a buffer containing 300 mM NaCl, 20 mM Na_2HPO_4, pH 7.4. Protein aliquots were stored in a buffer with 20 % glycerol at -80 °C. The purified protein was analyzed by SDS-PAGE with Coomassie blue staining, and its concentration was estimated by comparison to a BSA standard.

7.2 ITPK1 in Vitro Kinase Assays

Recombinant Arabidopsis thaliana ITPK1 was used in enzymatic assays to investigate the phosphorylation of inositol phosphates. The reactions were performed in 10 mM HEPES (pH 7.5), 2.5 mM $MgCl_2$, 1 mM DTT, 1 mM ATP, and an ATP regeneration system consisting of 5 mM phosphocreatine and 0.33 U creatine kinase, with 35 µM ITPK1 and 250 µM of the respective $InsP_5$ isomer as a substrate, in a total volume of 30 µL. Reactions were incubated at 25°C for 4 hours and quenched by incubation for 5 min at 70°C. Internal standards were added immediately prior to the analysis by CE-MS. Depending on signal intensity, some reaction mixtures were analyzed undiluted, while others were diluted prior to measurement.

7.3 CE-MS measurements

CE-MS measurements were performed on a CE-qTOF system for the analysis of synthetic molecules and on a CE-QQQ for the assignment of isomers in biological samples. Analysis of the ITPK1 Assays was performed on the CE-QTOF system. All experiments were performed with a bare fused silica capillary with a length of 100 cm and 50 mm internal diameter. A 40 mM ammonium acetate, titrated with ammonia solution to pH 9.0, was used as background electrolyte. Samples were injected by applying 100 mbar pressure for 10 s (20 nL). The setting of the CE-qTOF and CE-QQQ system were the same as described in the literature.[9,10]

7.4 Incubation of [1/3-OH]-5-PP-$InsP_4$ and [4/6-OH]-PP-$InsP_4$ with ITPK1

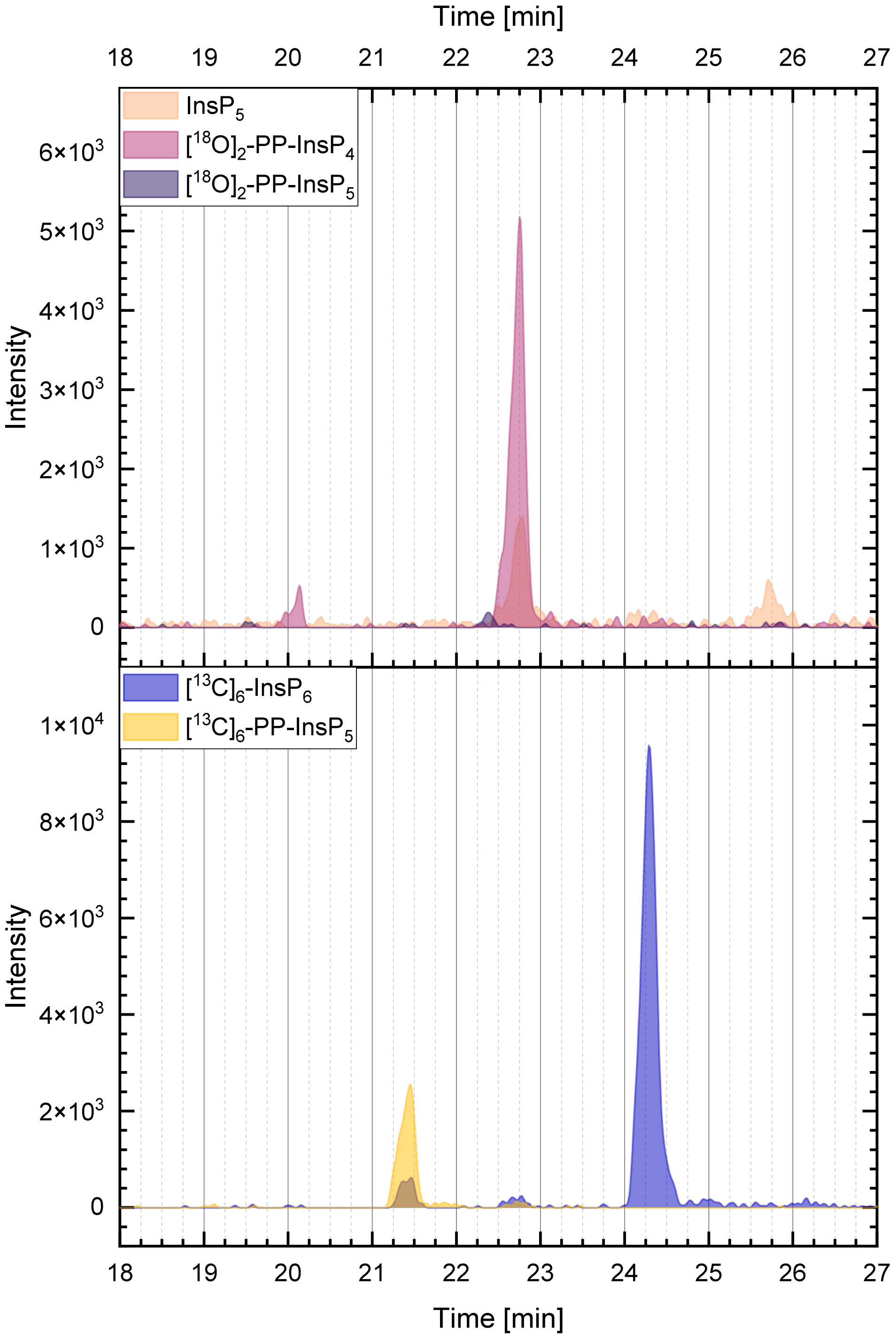

Figure S2: CE-MS electropherograms of $[^{18}O_2]$-labeled [1/3-OH]-5-PP-$InsP_4$ after 4 h incubation with recombinant Arabidopsis ITPK1 in the presence of ATP, creatine kinase and phosphocreatine. The upper panel shows extracted ion traces for the substrate $[^{18}O_2]$-PP-$InsP_4$ (m/z 330.0), the expected product $[^{18}O_2]$-PP-$InsP_5$ (m/z 368.9), and $InsP_5$ (m/z 289.0), which may result from spontaneous hydrolysis. The lower panel shows $[^{13}C_6]$-labeled internal standards: $[^{13}C_6]$-PP-$InsP_5$ (m/z 371.9) and $[^{13}C_6]$-$InsP_6$ (m/z 331.9). No conversion of $[^{18}O_2]$-[1/3-OH]-5-PP-$InsP_4$ to $[^{18}O_2]$-5-PP-$InsP_5$ was observed, indicating that [1/3-OH]-5-PP-$InsP_4$ is not accepted as a substrate under these conditions.

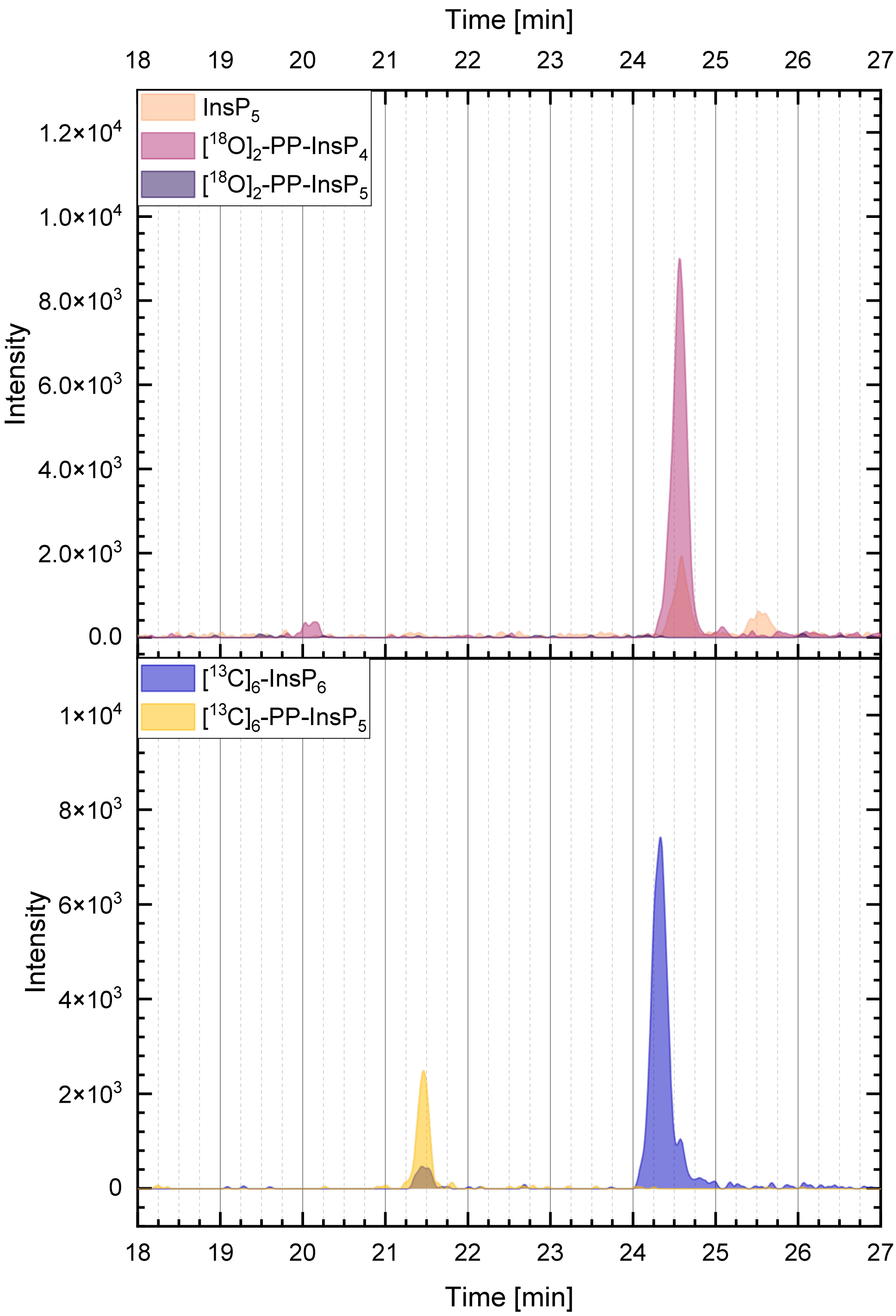

Figure S3: CE-MS electropherograms of [$^{18}O_2$]-labeled [4/6-OH]-5-PP-InsP$_4$ after 4 h incubation with recombinant Arabidopsis ITPK1 in the presence of ATP, creatine kinase and phosphocreatine. The upper panel shows extracted ion traces for the substrate [$^{18}O_2$]-PP-InsP$_4$ (m/z 330.0), the expected product [$^{18}O_2$]-PP-InsP$_5$ (m/z 368.9), and InsP$_5$ (m/z 289.0), which may result from spontaneous hydrolysis. The lower panel shows [$^{13}C_6$]-labeled internal standards: [$^{13}C_6$]-PP-InsP$_5$ (m/z 371.9) and [$^{13}C_6$]-InsP$_6$ (m/z 331.9). No conversion of [$^{18}O_2$]-[4/6-OH]-5-PP-InsP$_4$ to [$^{18}O_2$]-5-PP-InsP$_5$ was observed, indicating that [4/6-OH]-5-PP-InsP$_4$ is not accepted as a substrate under these conditions.

7.5 Negative Controls (ATP Only, No Enzyme)

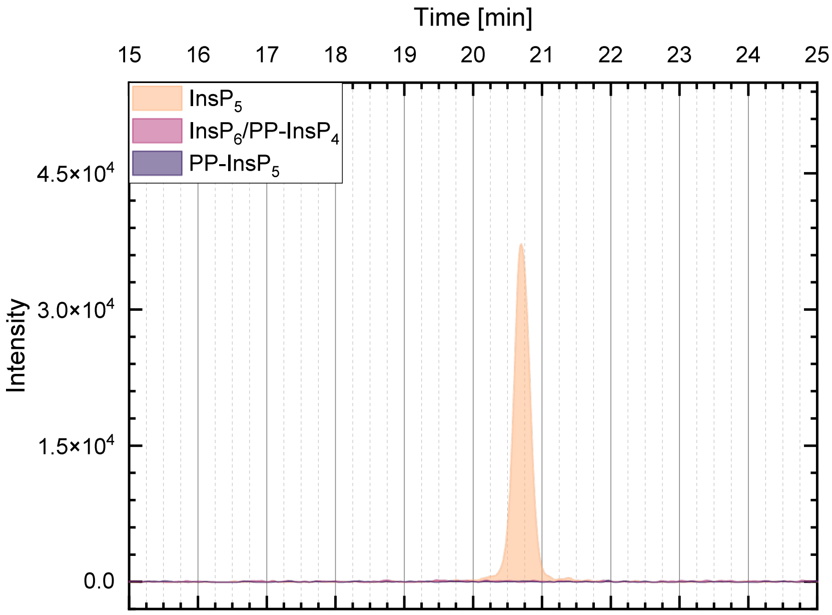

Figure S4: CE-MS electropherogram of [1-OH]-$InsP_5$ after incubation in the presence of ATP only (1 mM), without ITPK1 or an ATP regeneration system. Mass traces are shown for $InsP_5$ (m/z 289.0), $InsP_6$/PP-$InsP_4$ (m/z 328.9), and PP-$InsP_5$ (m/z 368.9). No formation of phosphorylated products was detected under these conditions.

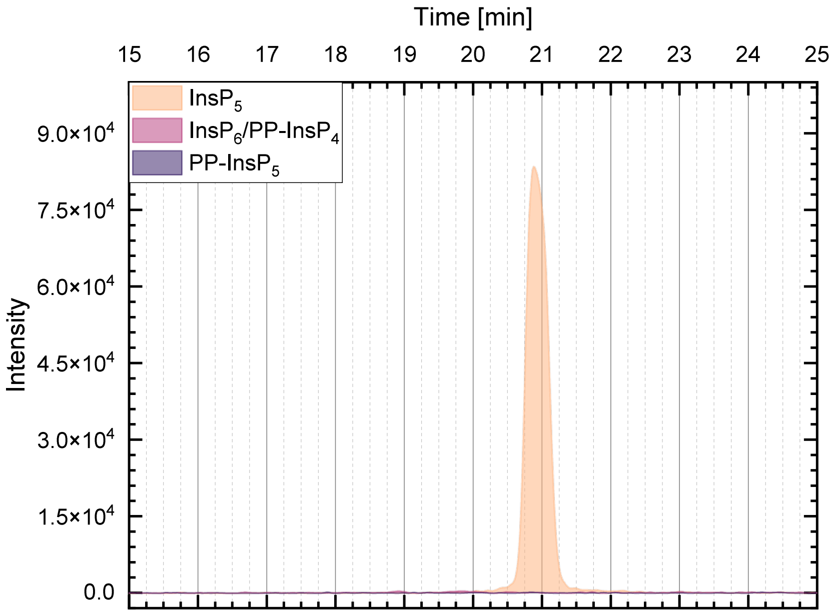

Figure S5: CE-MS electropherogram of [3-OH]-$InsP_5$ after incubation in the presence of ATP only (1 mM), without ITPK1 or an ATP regeneration system. Mass traces are shown for $InsP_5$ (m/z 289.0), $InsP_6$/PP-$InsP_4$ (m/z 328.9), and PP-$InsP_5$ (m/z 368.9). No formation of phosphorylated products was detected under these conditions.

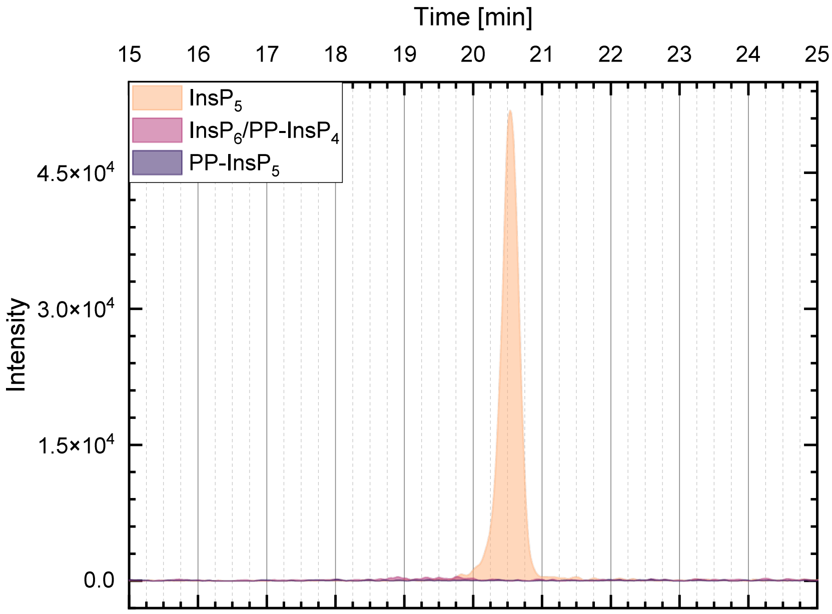

Figure S6: CE-MS electropherogram of **[4-OH]-InsP$_5$** after incubation in the presence of ATP only (1 mM), without ITPK1 or an ATP regeneration system. Mass traces are shown for InsP$_5$ (m/z 289.0), InsP$_6$/PP-InsP$_4$ (m/z 328.9), and PP-InsP$_5$ (m/z 368.9). No formation of phosphorylated products was detected under these conditions.

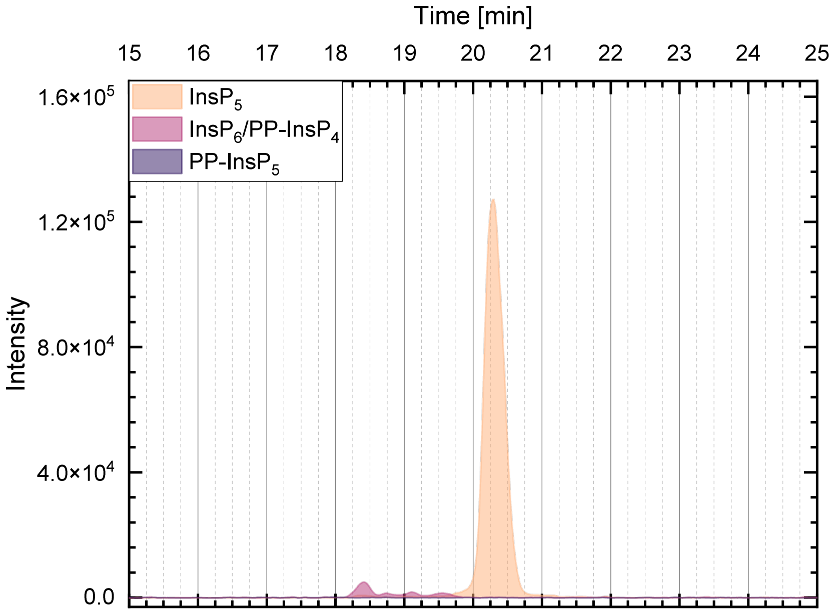

Figure S7: CE-MS electropherogram of **[6-OH]-InsP$_5$** after incubation in the presence of ATP only (1 mM), without ITPK1 or an ATP regeneration system. Mass traces are shown for InsP$_5$ (m/z 289.0), InsP$_6$/PP-InsP$_4$ (m/z 328.9), and PP-InsP$_5$ (m/z 368.9). No formation of phosphorylated products was detected under these conditions.

7.6 Time-Resolved CE-MS Analysis of ITPK1-Catalyzed Conversion of $InsP_5$ Isomers

Table S1: Normalized CE-MS peak areas of [3-OH]-$InsP_5$ and its phosphorylation products over time.

Time [in h]	[3-OH]-$InsP_5$ Normalized Peak Area [%]	[3-OH]-5-PP-$InsP_4$ Normalized Peak Area [%]	5-PP-$InsP_5$ Normalized Peak Area [%]
0	100	0	0
0.5	76	18	5
1	78	17	5
2	62	29	10
3	57	28	15
4	51	37	12

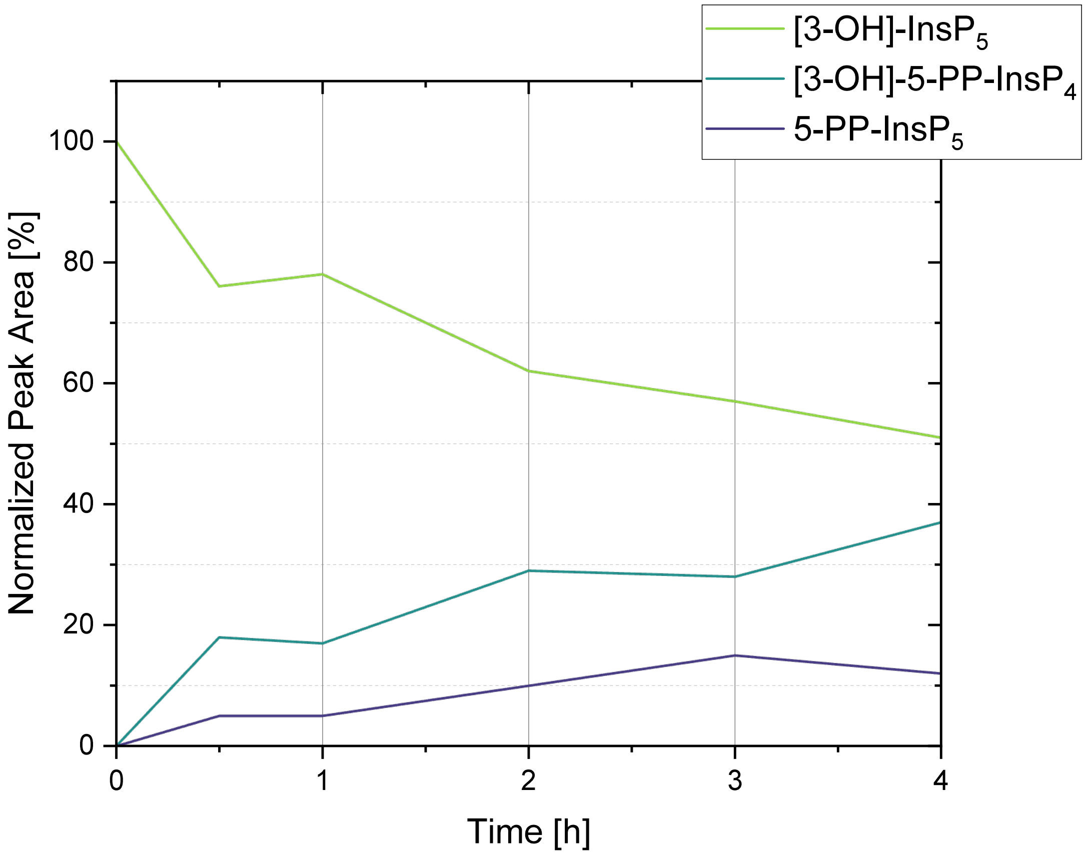

Figure S8: Time-dependent conversion of [3-OH]-$InsP_5$ by Arabidopsis ITPK1, monitored by CE-MS. Peak areas of [3-OH]-$InsP_5$ (green), [3-OH]-5-PP-$InsP_4$ (blue), and 5-PP-$InsP_5$ (purple) were normalized to the total signal at each time point and plotted as percentage values.

Table S2: Normalized CE-MS peak areas of [4-OH]-$InsP_5$ and its phosphorylation products over time.

Time [in h]	[4-OH]-$InsP_5$ Normalized Peak Area [%]	[4-OH]-5-PP-$InsP_4$ Normalized Peak Area [%]	5-PP-$InsP_5$ Normalized Peak Area [%]
0	100	0	0
0.5	94	0	6
1	93	0	7
2	87	0	13
3	87	0	13
4	80	0	20

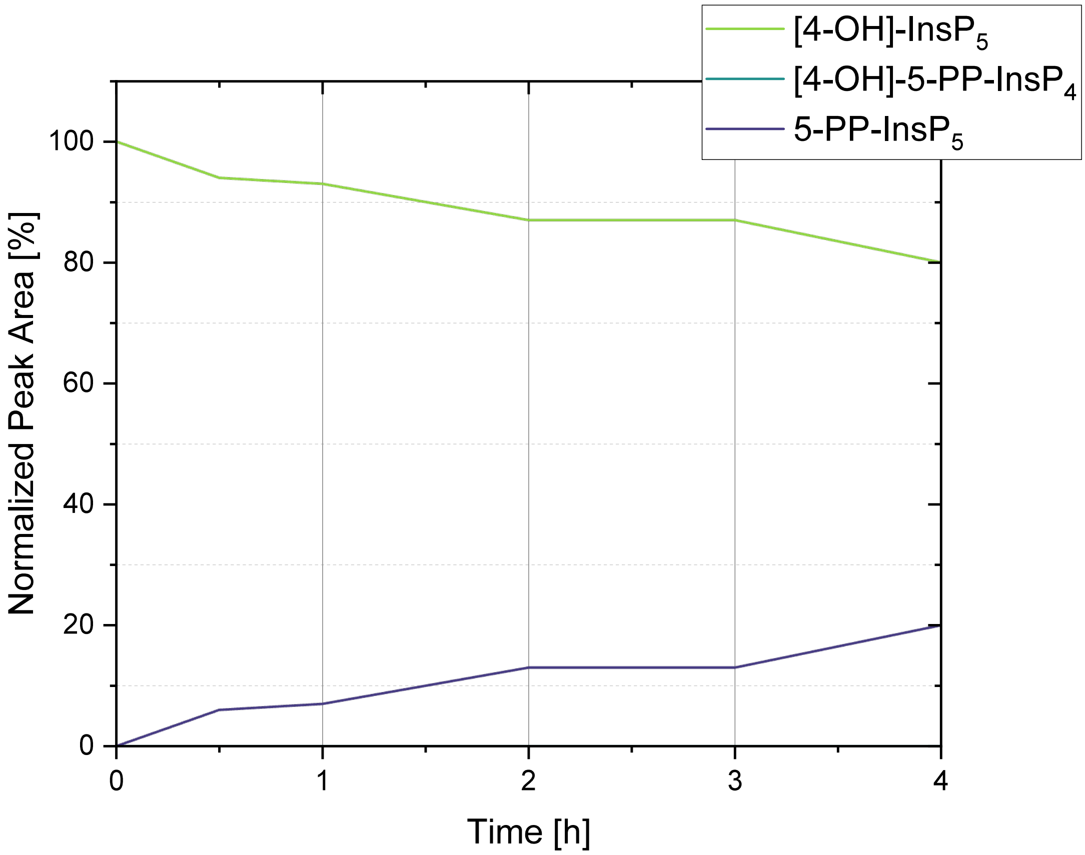

Figure S9: Time-dependent conversion of [4-OH]-$InsP_5$ by Arabidopsis ITPK1, monitored by CE-MS. Peak areas of [4-OH]-$InsP_5$ (green), [4-OH]-5-PP-$InsP_4$ (blue), and 5-PP-$InsP_5$ (purple) were normalized to the total signal at each time point and plotted as percentage values.

Table S3: Normalized CE-MS peak areas of [6-OH]-InsP$_5$ and its phosphorylation products over time.

Time [in h]	[6-OH]-InsP$_5$ Normalized Peak Area [%]	[6-OH]-5-PP-InsP$_4$ Normalized Peak Area [%]	5-PP-InsP$_5$ Normalized Peak Area [%]
0	100	0	0
0.5	85	15	0
1	76	23	1
2	63	36	1
3	53	46	1
4	43	56	1

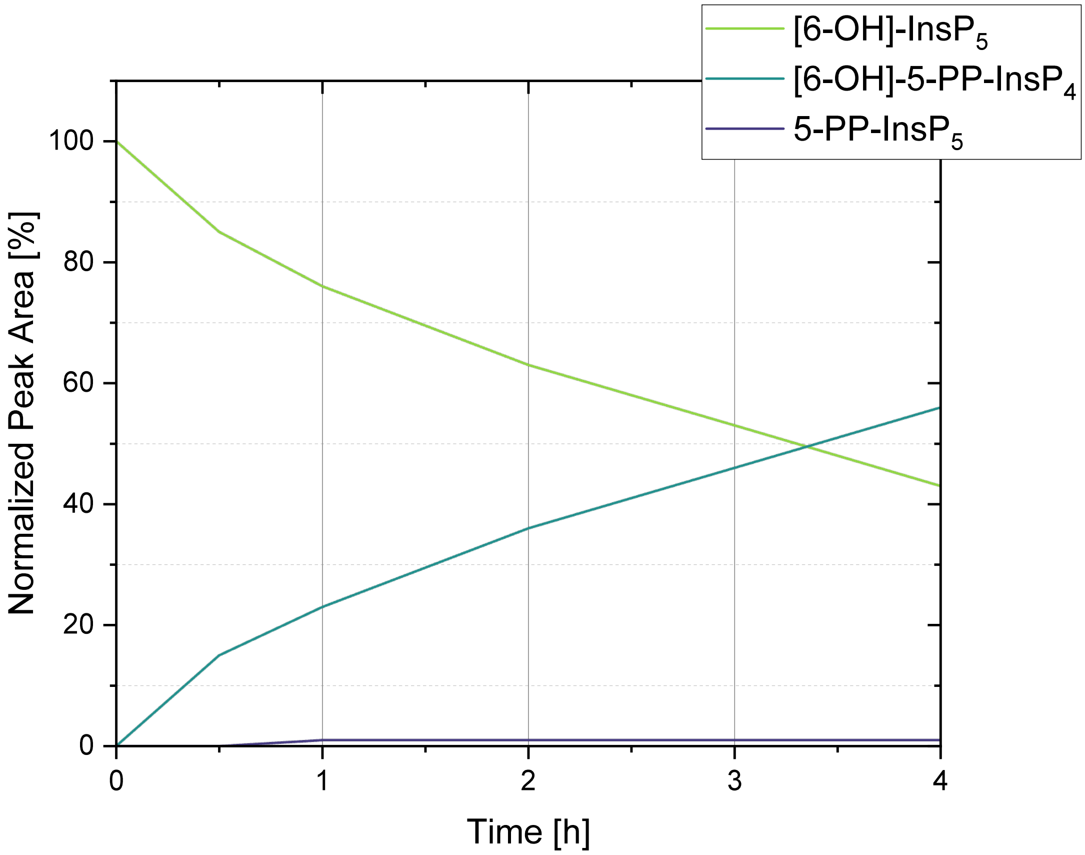

Figure S10: Time-dependent conversion of [6-OH]-InsP$_5$ by Arabidopsis ITPK1, monitored by CE-MS. Peak areas of [6-OH]-InsP$_5$ (green), [6-OH]-5-PP-InsP$_4$ (blue), and 5-PP-InsP$_5$ (purple) were normalized to the total signal at each time point and plotted as percentage values.

8 Literature

[1] A. Hofer, G. S. Cremosnik, A. C. Müller, R. Giambruno, C. Trefzer, G. Superti-Furga, K. L. Bennett, H. J. Jessen, *Chem. Eur. J.* **2015**, *21*, 10116–10122.

[2] K. Pahnke, C. Meier, *ChemBioChem* **2017**, *18*, 1616–1626.

[3] H. Y. Godage, A. M. Riley, T. J. Woodman, M. P. Thomas, M. F. Mahon, B. V. L. Potter, *J. Org. Chem.* **2013**, *78*, 2275–2288.

[4] S. Capolicchio, D. T. Thakor, A. Linden, H. J. Jessen, *Angew. Chem. Int. Ed.* **2013**, *52*, 6912–6916.

[5] A. Hager, M. Wu, H. Wang, N. W. Brown Jr., S. B. Shears, N. Veiga, D. Fiedler, *Chem. Eur. J.* **2016**, *22*, 12406–12414.

[6] C. Murali, M. S. Shashidhar, C. S. Gopinath, *Tetrahedron* **2007**, *63*, 4149–4155.

[7] M. A. L. Podeschwa, O. Plettenburg, H.-J. Altenbach, *Eur. J. Org. Chem.* **2005**, *2005*, 3116–3127.

[8] D. Laha, N. Parvin, A. Hofer, R. F. H. Giehl, N. Fernandez-Rebollo, N. Von Wirén, A. Saiardi, H. J. Jessen, G. Schaaf, *ACS Chem. Biol.* **2019**, *14*, 2127–2133.

[9] D. Qiu, M. S. Wilson, V. B. Eisenbeis, R. K. Harmel, E. Riemer, T. M. Haas, C. Wittwer, N. Jork, C. Gu, S. B. Shears, G. Schaaf, B. Kammerer, D. Fiedler, A. Saiardi, H. J. Jessen, *Nat. Commun.* **2020**, *11*, 6035.

[10] D. Qiu, C. Gu, G. Liu, K. Ritter, V. B. Eisenbeis, T. Bittner, A. Gruzdev, L. Seidel, B. Bengsch, S. B. Shears, H. J. Jessen, *Chem. Sci.* **2022**, *14*, 658–667.

9 NMR – Spectra

(Sorted according to molecule numbering)

Compound 4: ^1H – NMR ($CDCl_3$, 400 MHz)

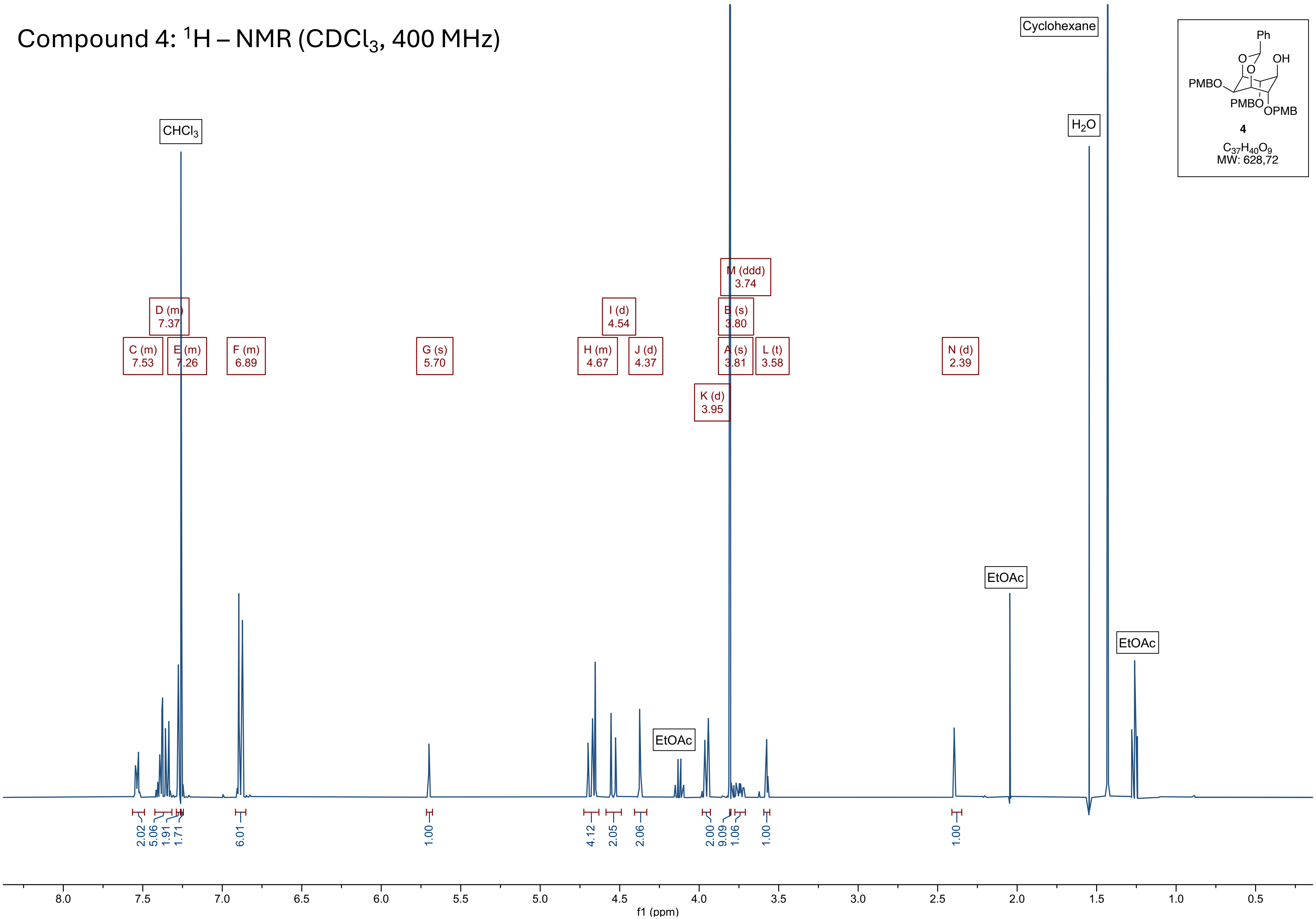

Compound 6: ^{1}H – NMR ($CDCl_3$, 400 MHz)

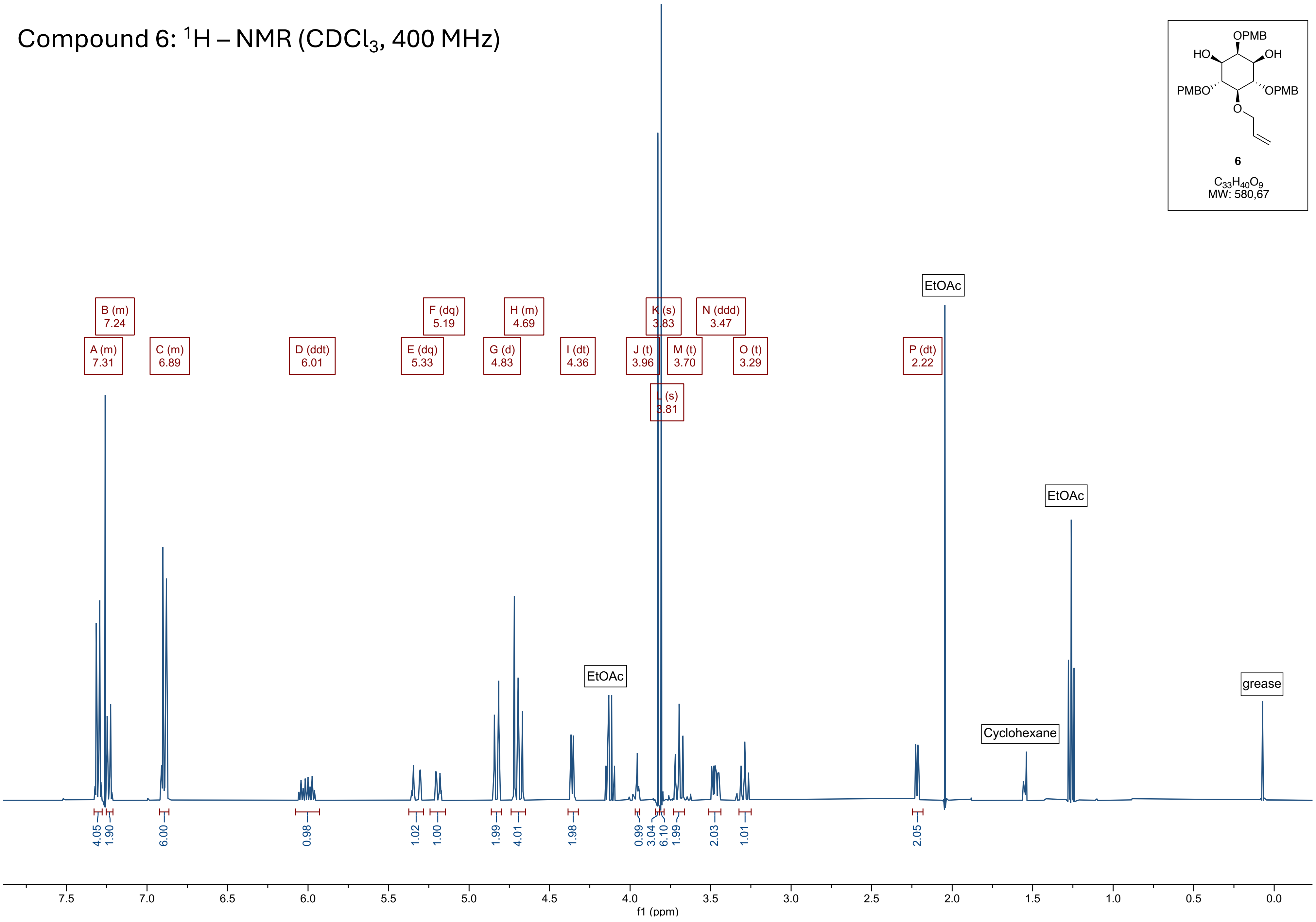

Compound ±7: ^{1}H – NMR ($CDCl_3$, 400 MHz)

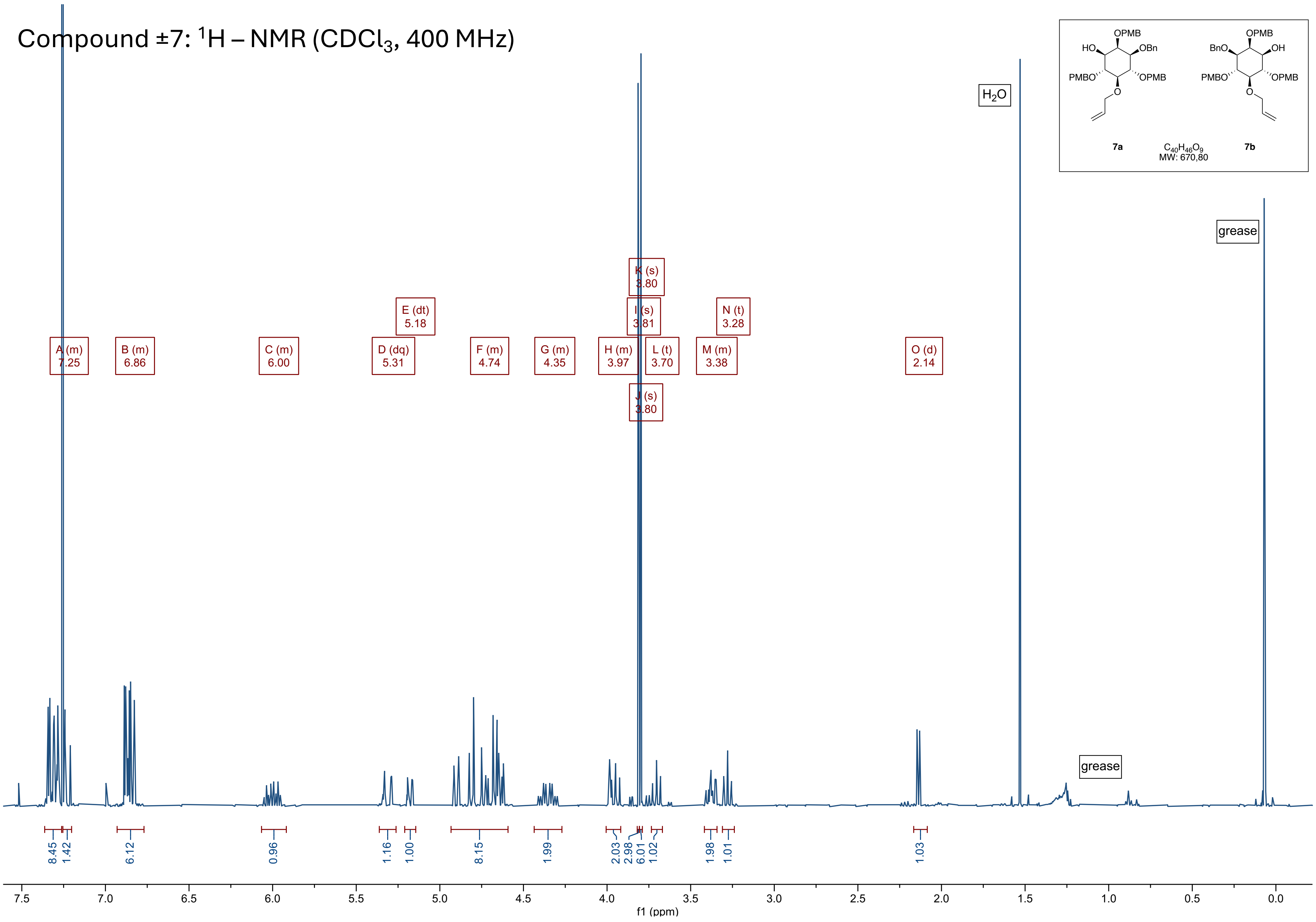

Compound ±7: $^{13}C\{^{1}H\}$ – NMR ($CDCl_3$, 101 MHz)

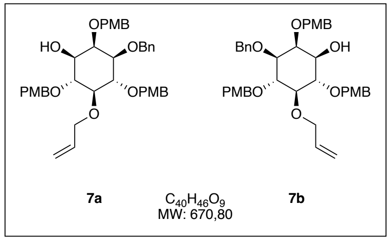

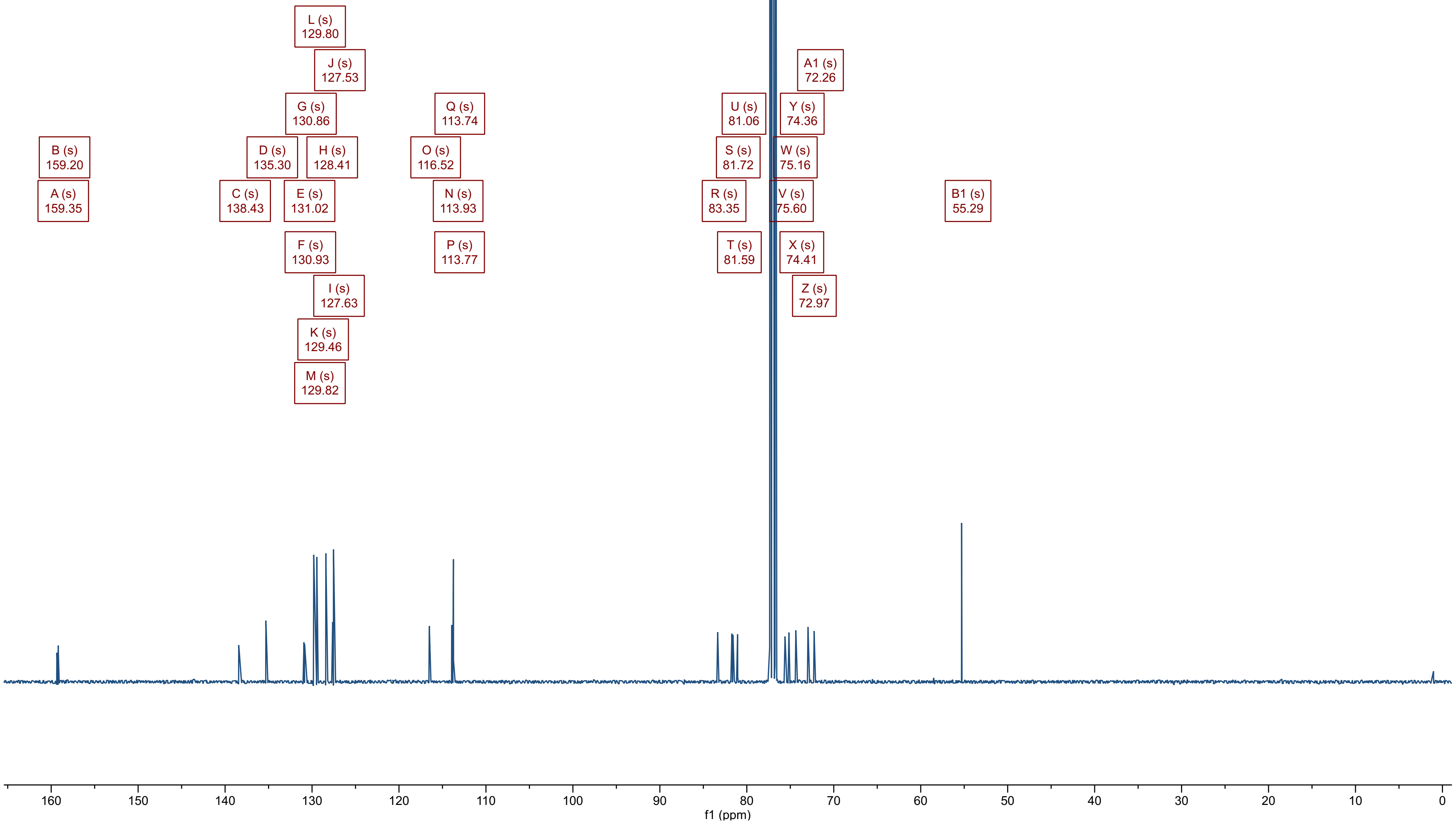

Compound ±9: ¹H – NMR ($CDCl_3$, 400 MHz)

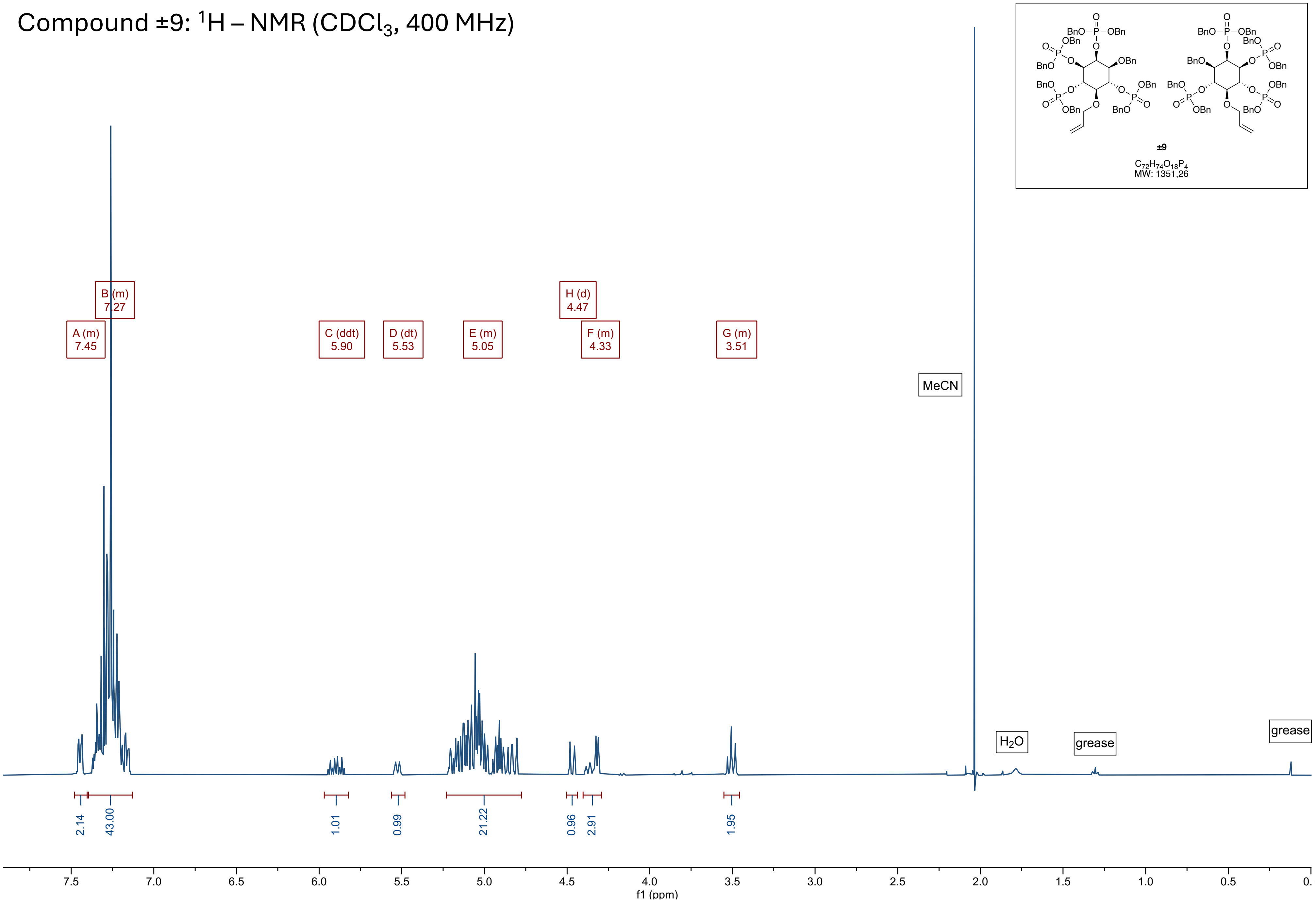

Compound ±9: $^{31}P\{^1H\}$ – NMR ($CDCl_3$, 162 MHz)

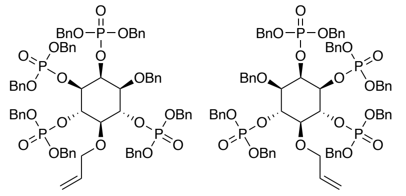

±9

$C_{72}H_{74}O_{18}P_4$
MW: 1351,26

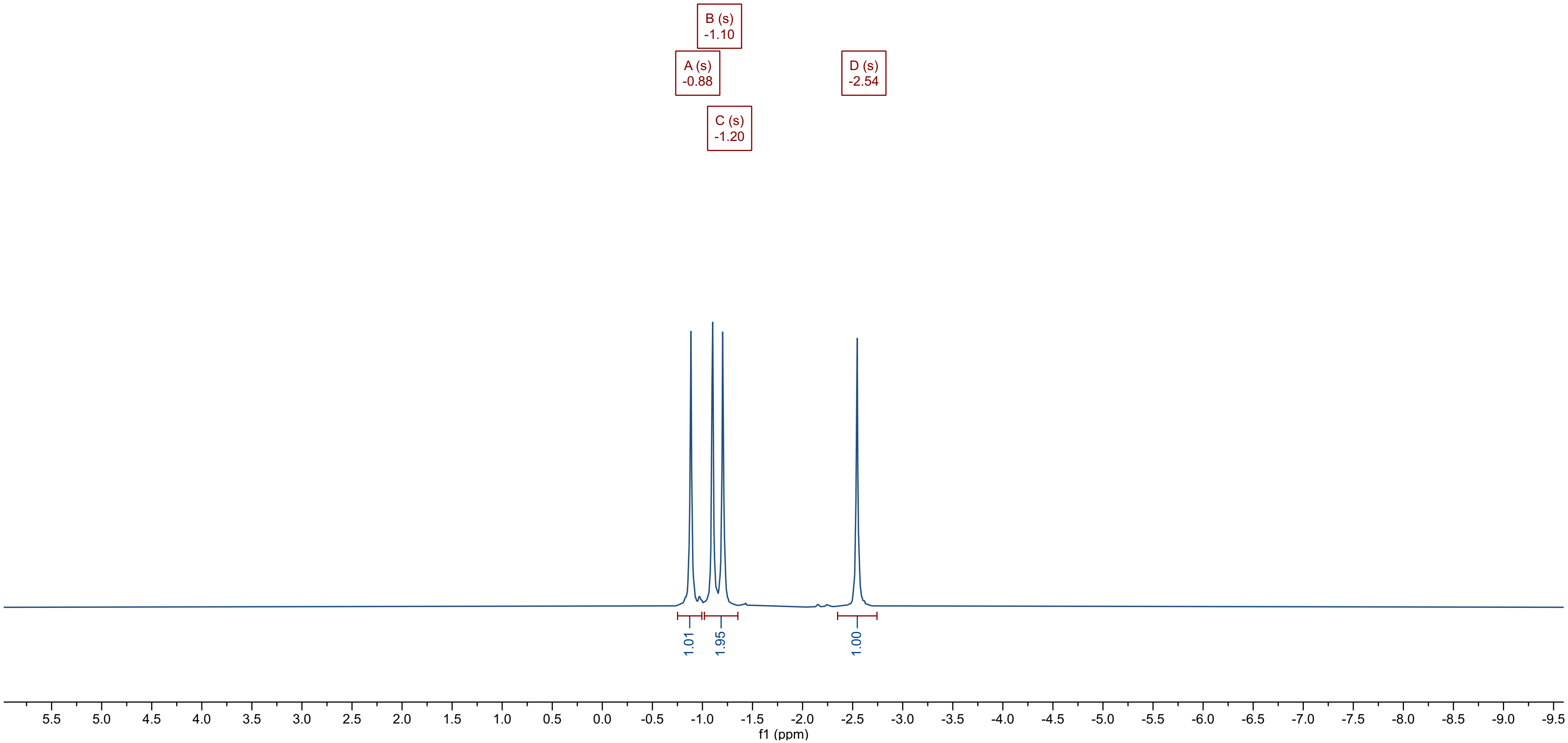

Compound ±9: $^{13}C\{^1H\}$ – NMR ($CDCl_3$, 101 MHz)

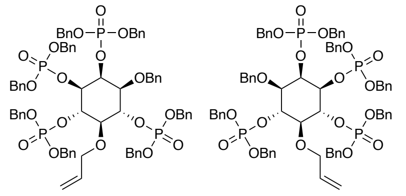

±9

$C_{72}H_{74}O_{18}P_4$
MW: 1351,26

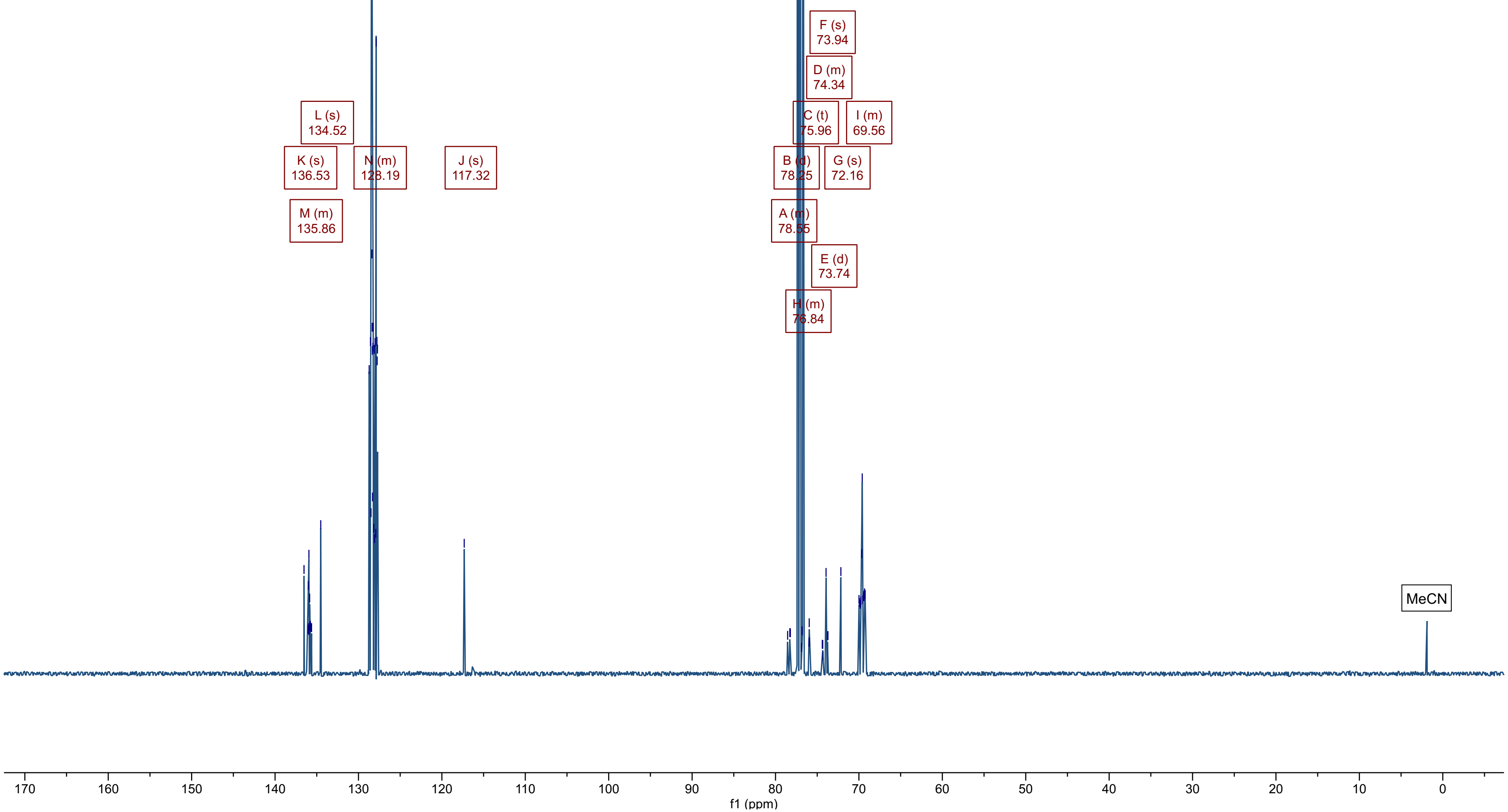

Compound ±11: ^1H – NMR ($CDCl_3$, 400 MHz)

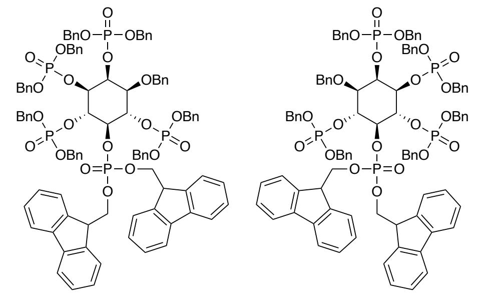

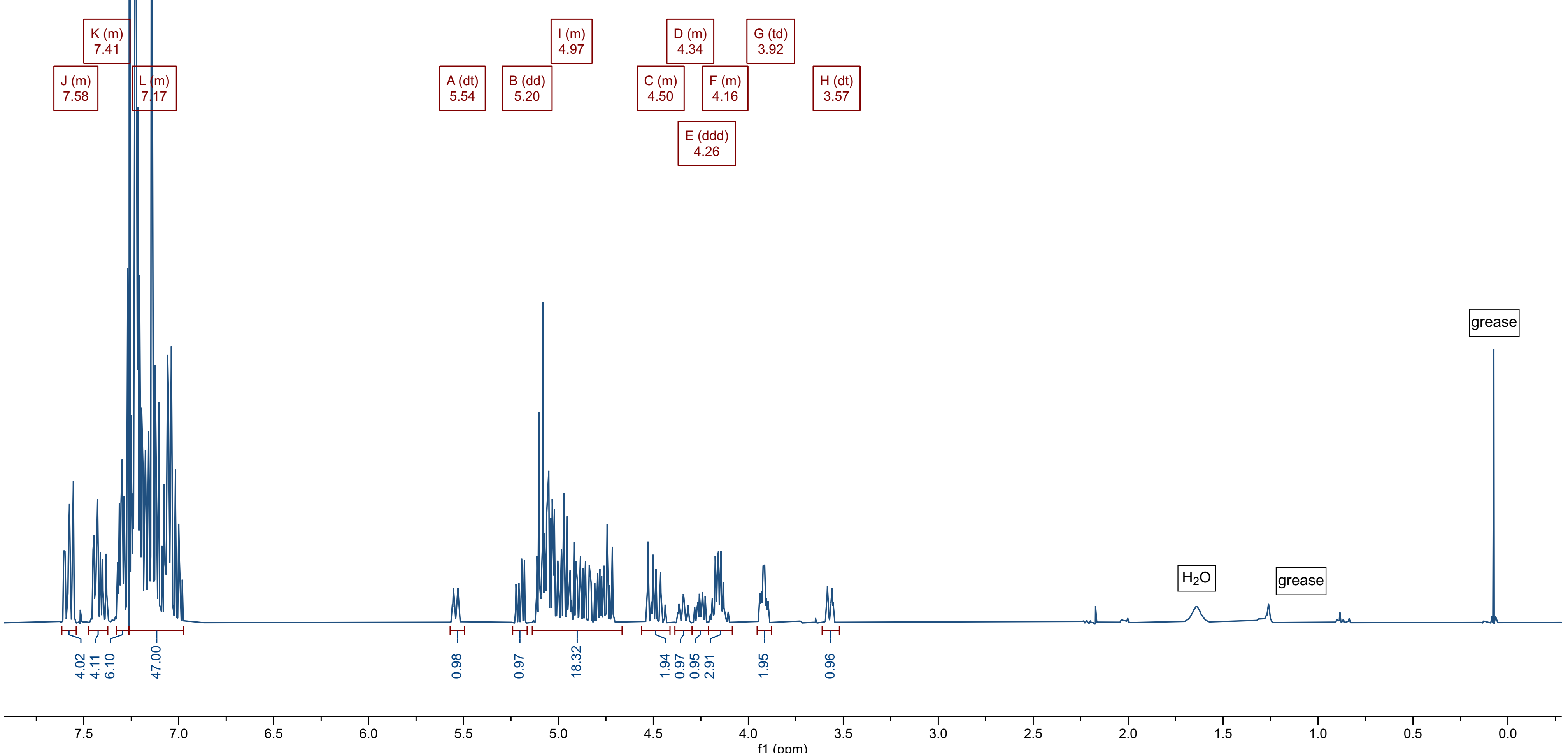

Compound ±11: $^{31}P\{^1H\}$ – NMR ($CDCl_3$, 162 MHz)

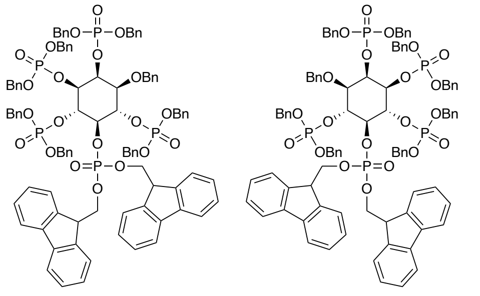

±11

$C_{97}H_{91}O_{21}P_5$
MW: 1747,64

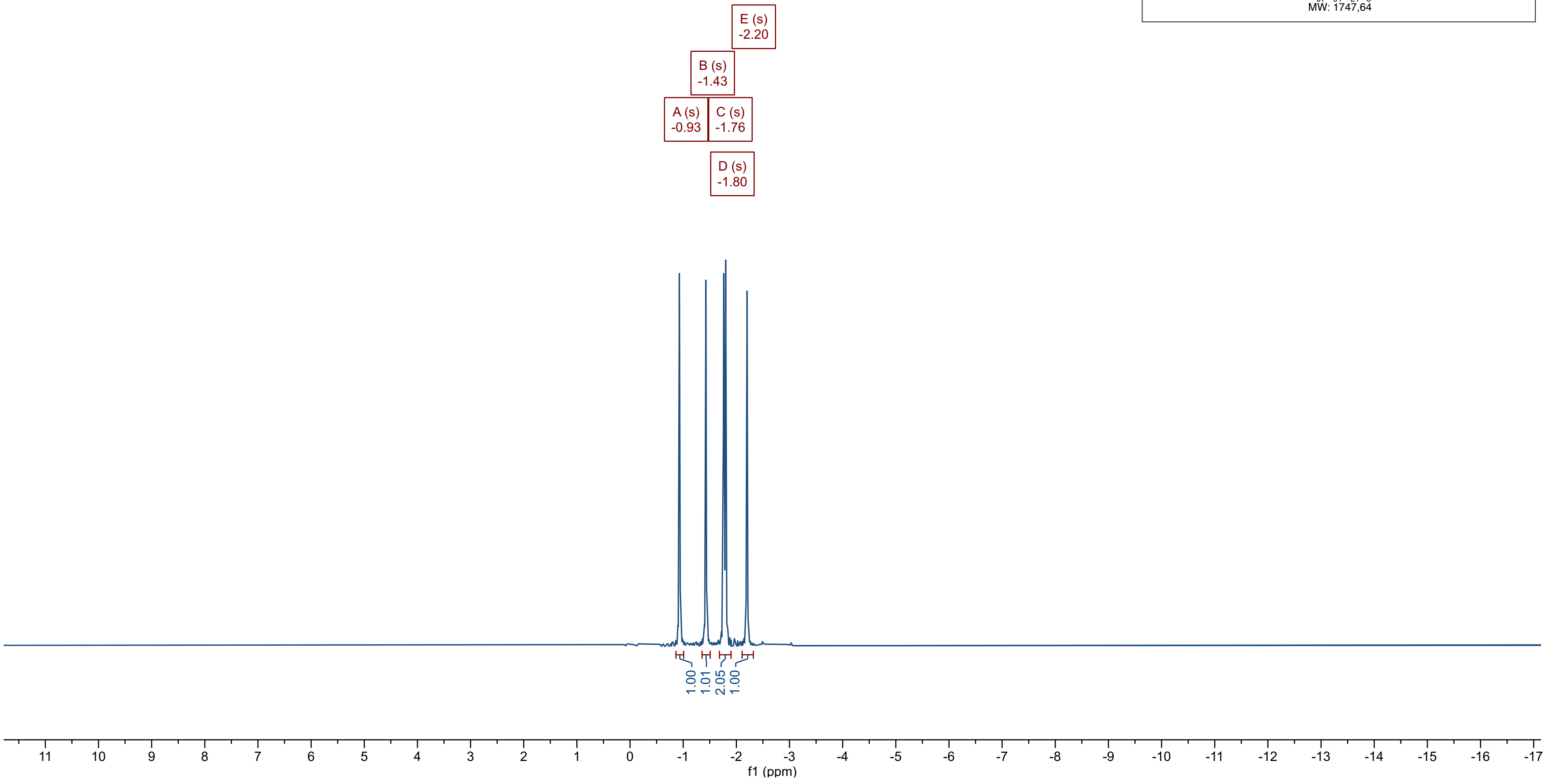

Compound ±11: $^{13}C\{^1H\}$ – NMR ($CDCl_3$, 101 MHz)

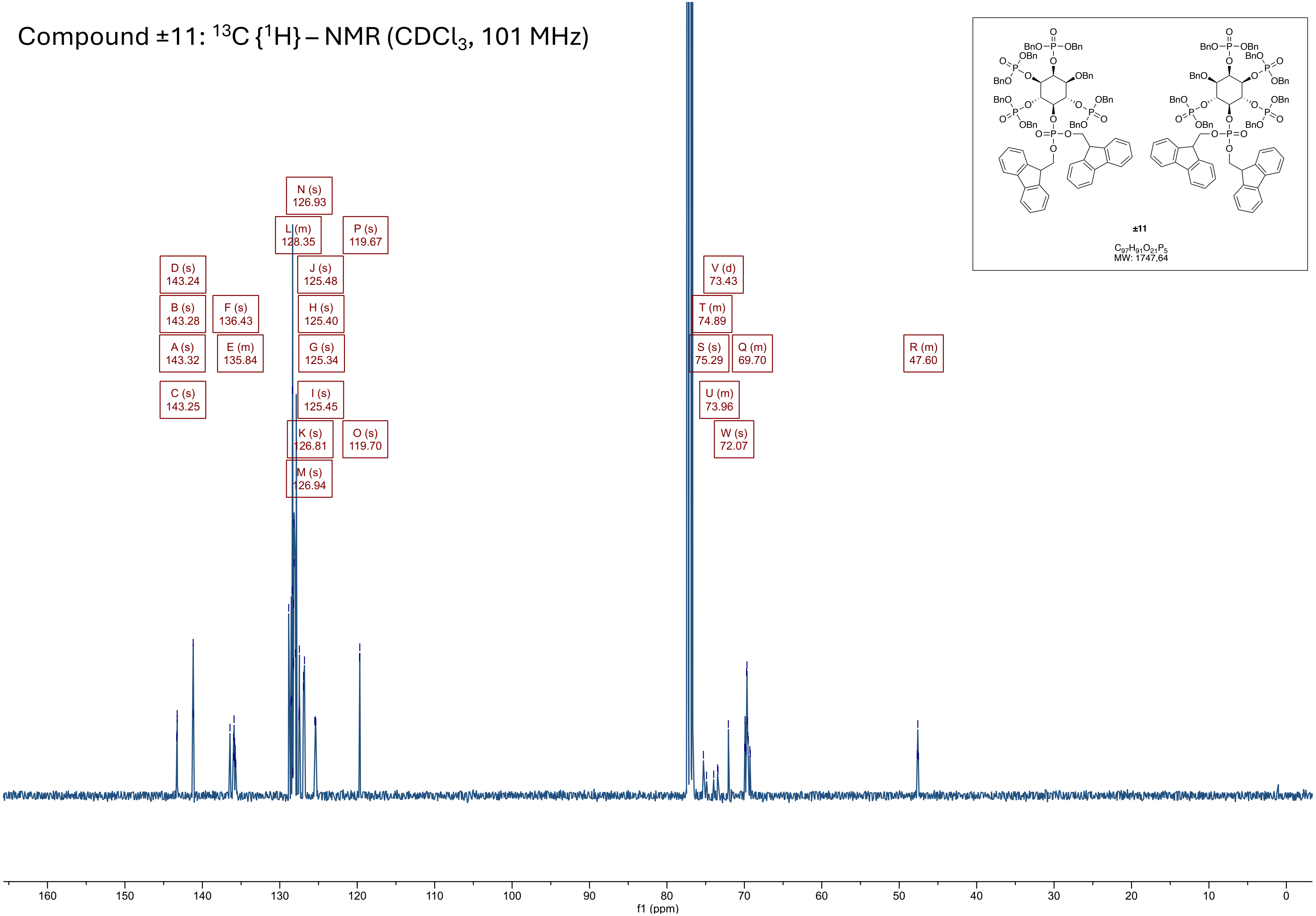

Compound ±12: ^{1}H – NMR ($CDCl_3$, 400 MHz)
TEA-Salt

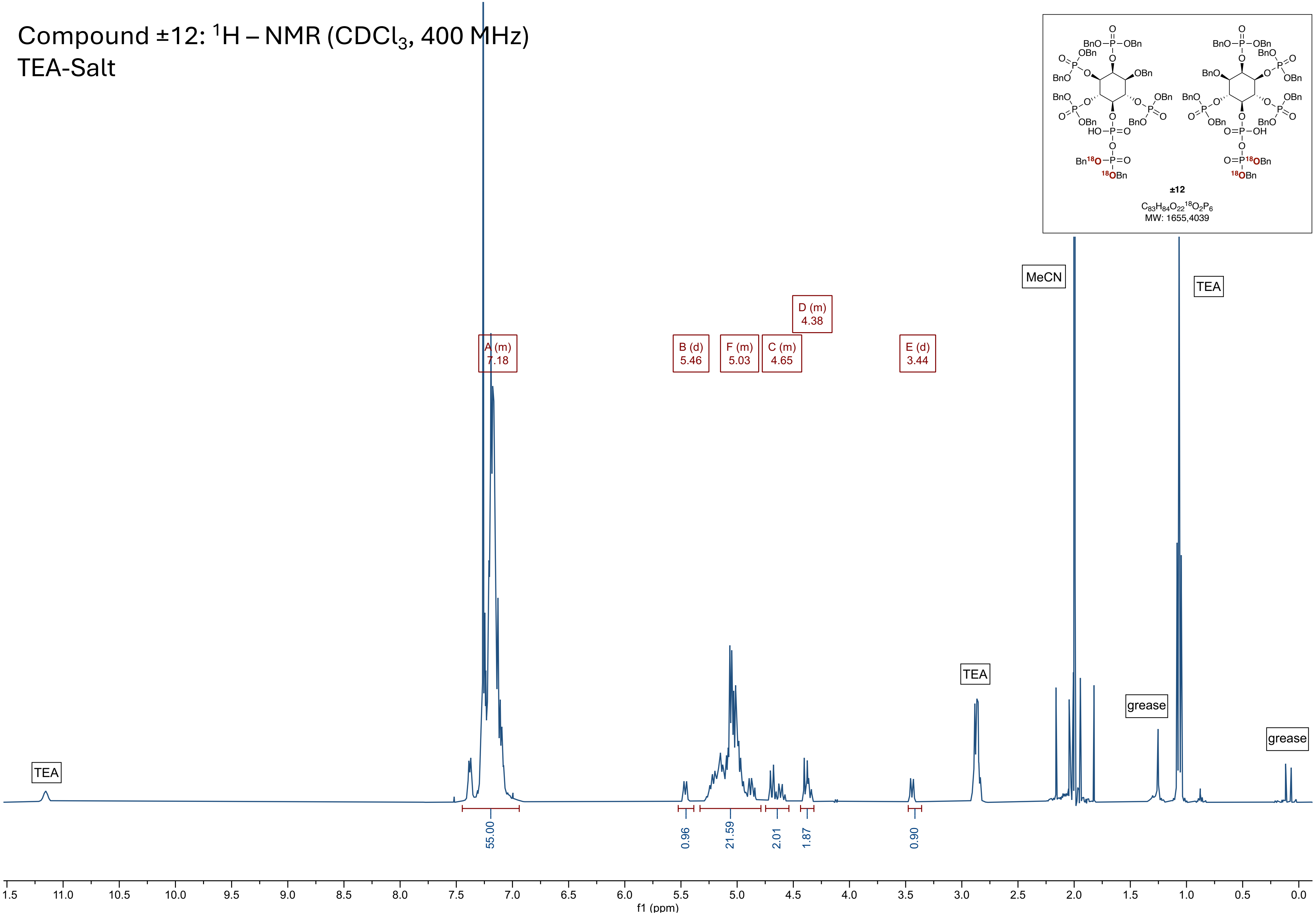

Compound ±12: $^{31}P\{^{1}H\}$ – NMR ($CDCl_3$, 162 MHz)

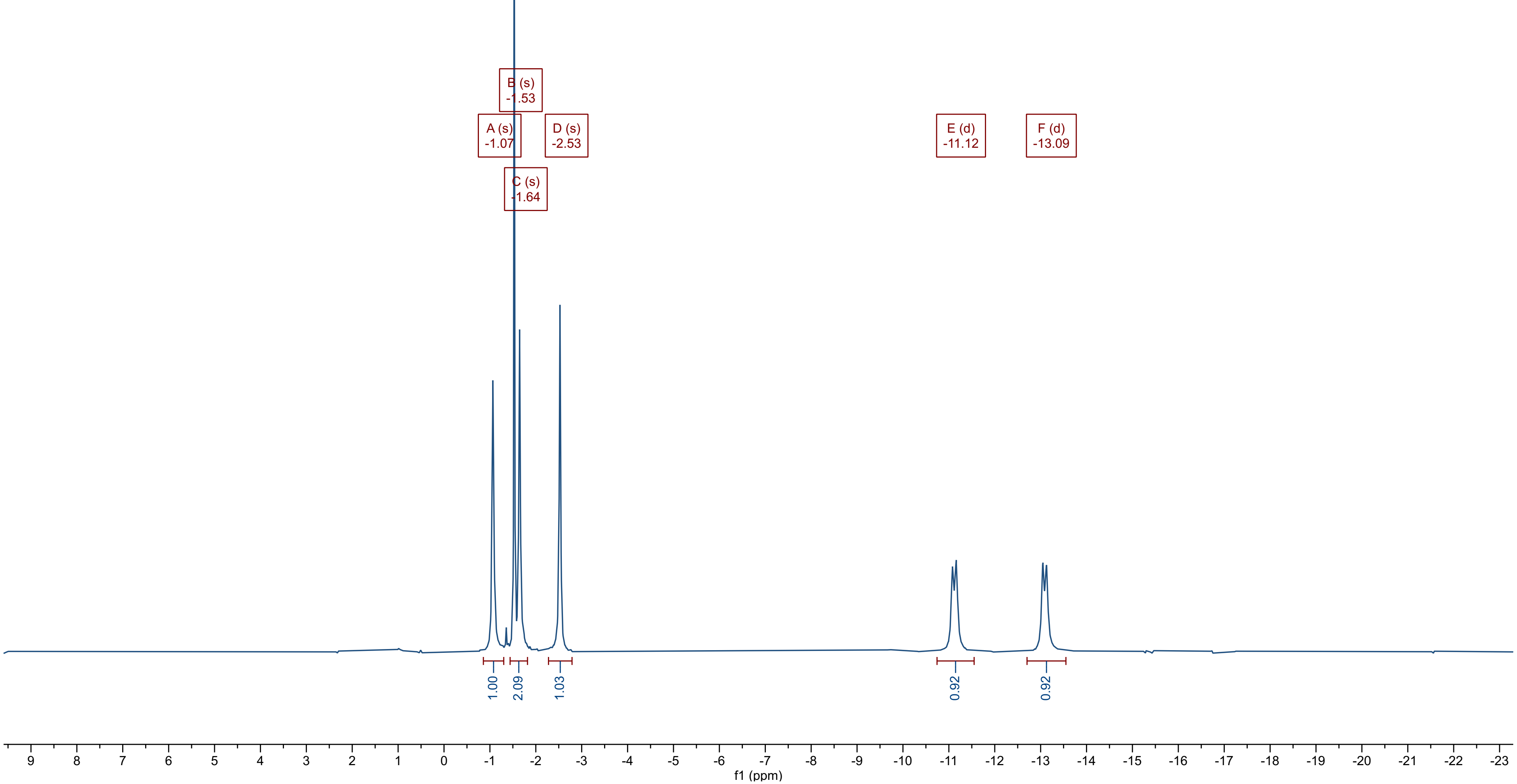

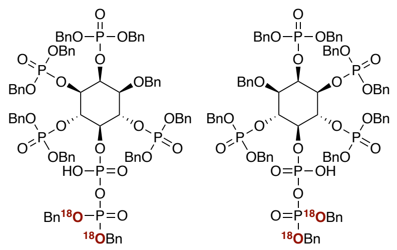

±12

$C_{83}H_{84}O_{22}{}^{18}O_2P_6$

MW: 1655,4039

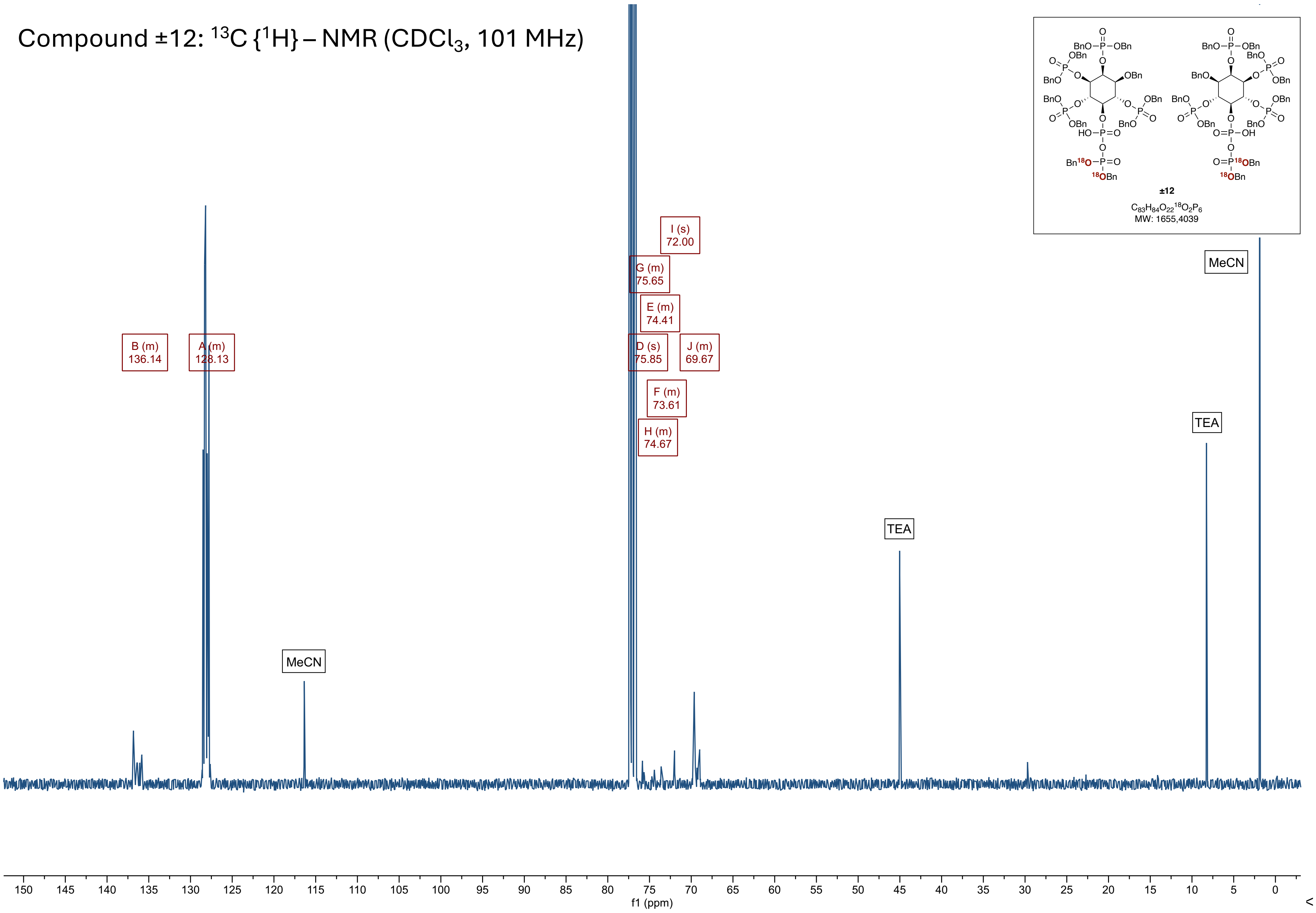

Compound ±12: 13C {1H} – NMR (CDCl3, 101 MHz)
B (m)
136.14
A (m)
128.13
MeCN
I (s)
72.00
G (m)
75.65
E (m)
74.41
D (s)
75.85
J (m)
69.67
F (m)
73.61
H (m)
74.67
TEA
TEA
MeCN
±12
C83H84O22 18O2P6
MW: 1655,4039
f1 (ppm)

Compound ±13: ^{1}H – NMR (D_2O, 400 MHz)

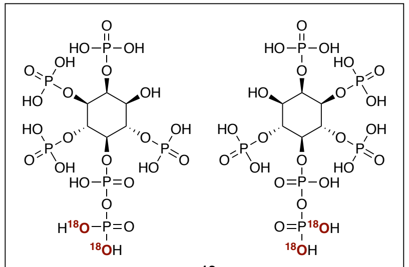

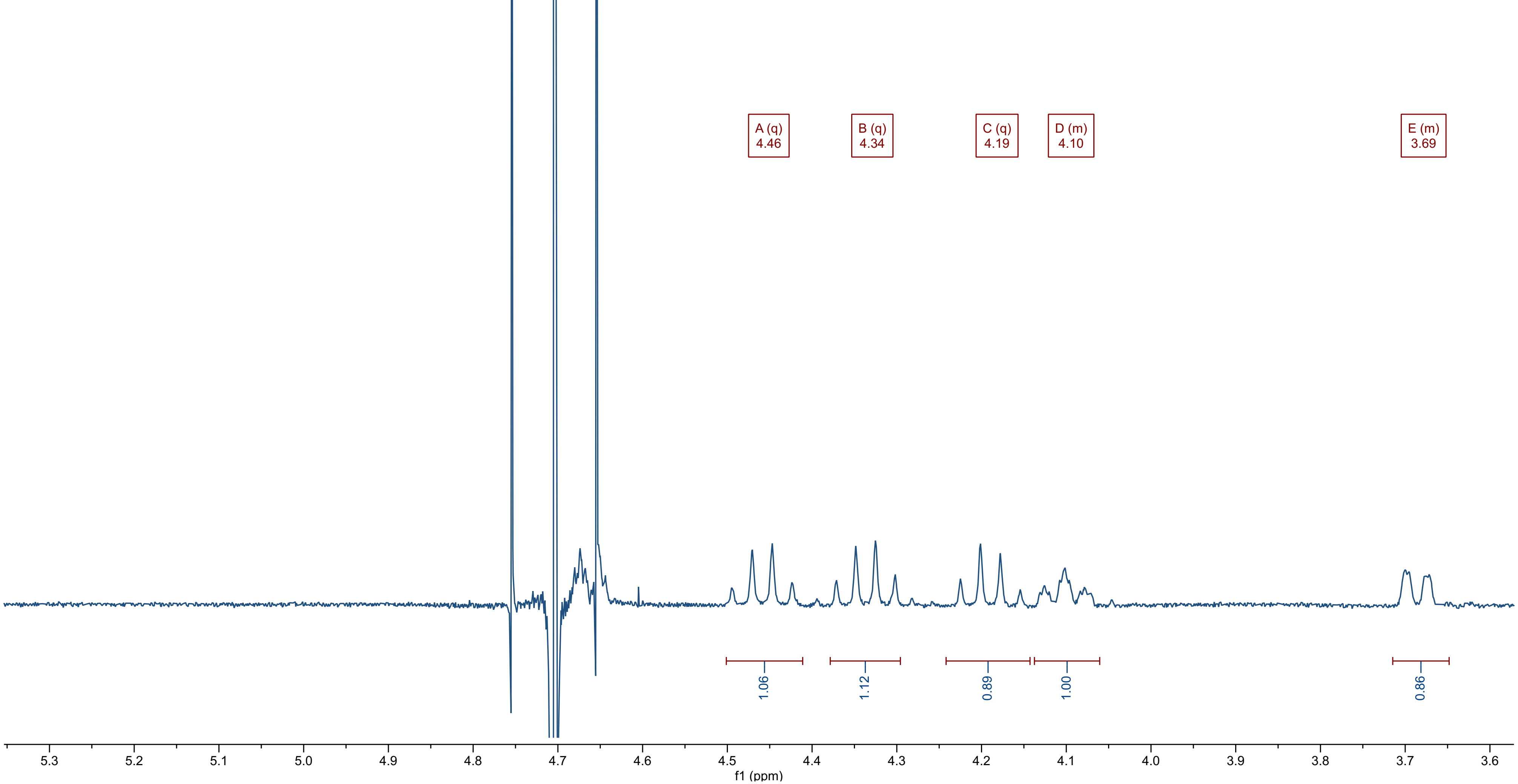

Compound ±13: $^{31}P\{^1H\}$ – NMR (D_2O, 162 MHz)

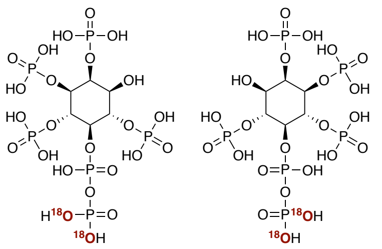

±13

$C_6H_{18}O_{22}{}^{18}O_2P_6$
MW: 664,0289

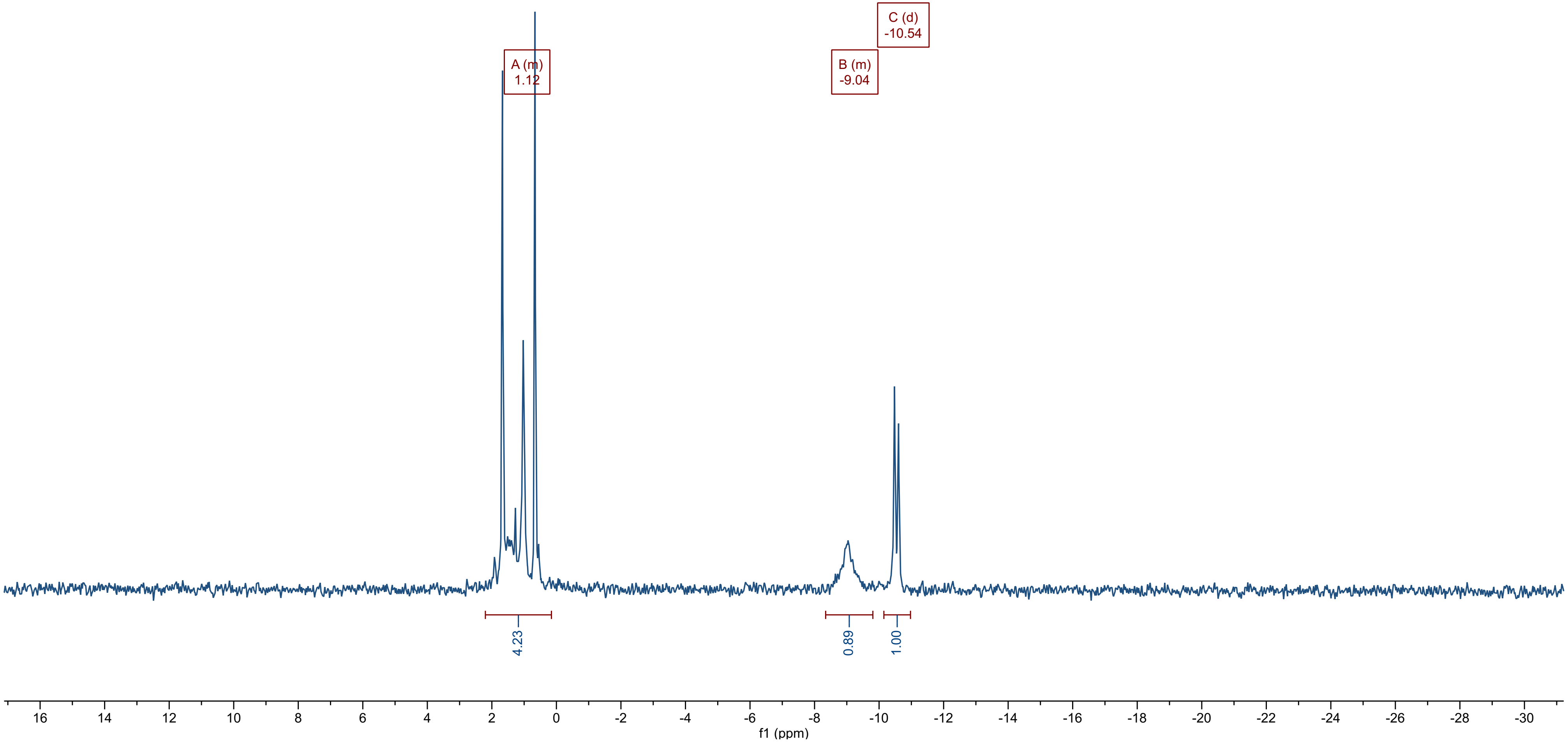

Compound ±13: edHSQC to identify ^{13}C peaks

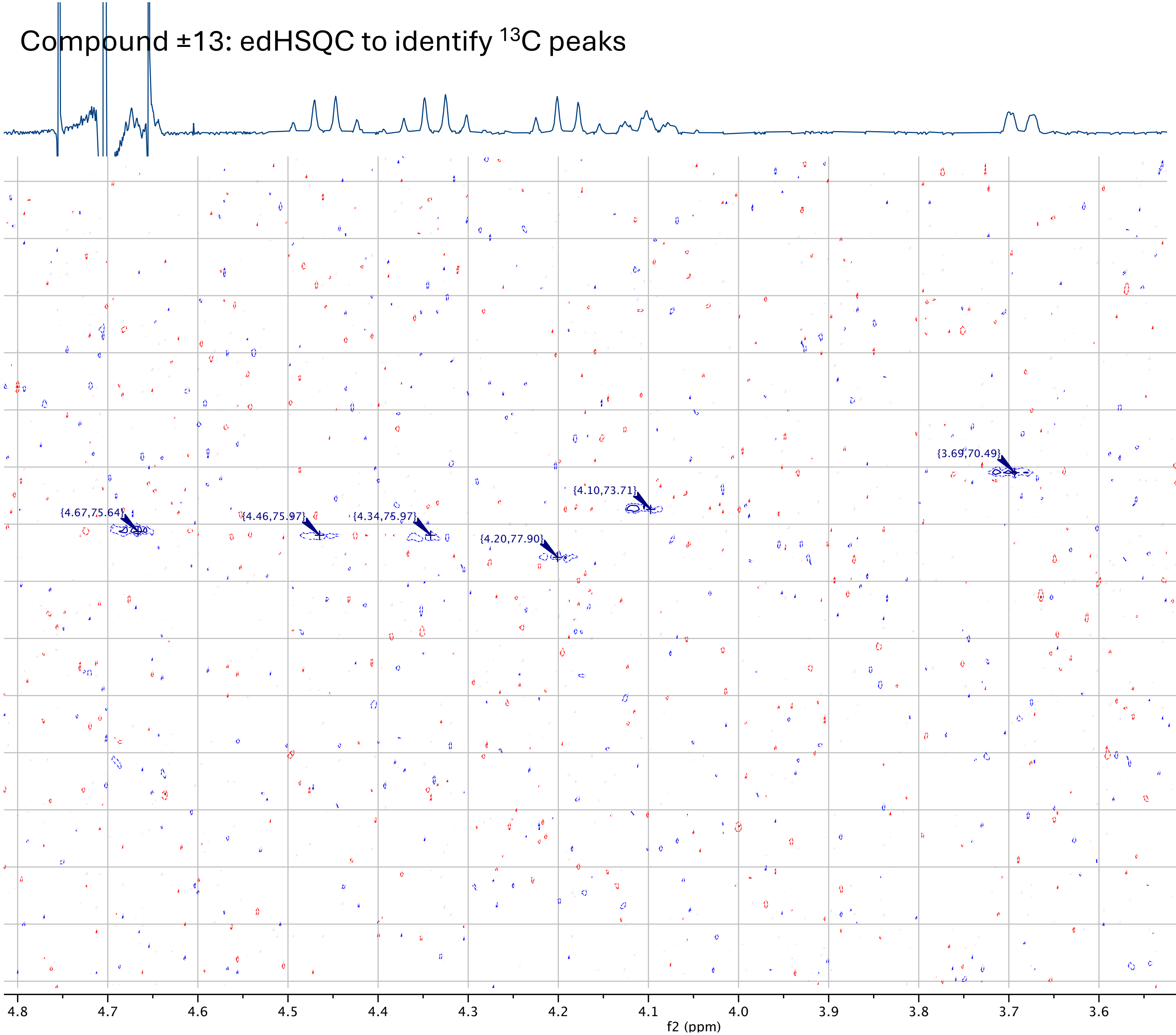

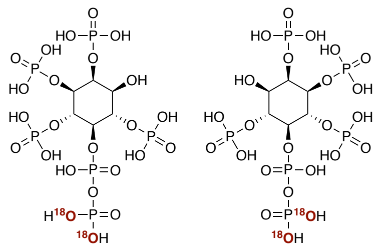

±13

$C_6H_{18}O_{22}{}^{18}O_2P_6$

MW: 664,0289

Compound ±13: ^{1}H – NMR (D_2O, 400 MHz)
Supplemented with EDTA

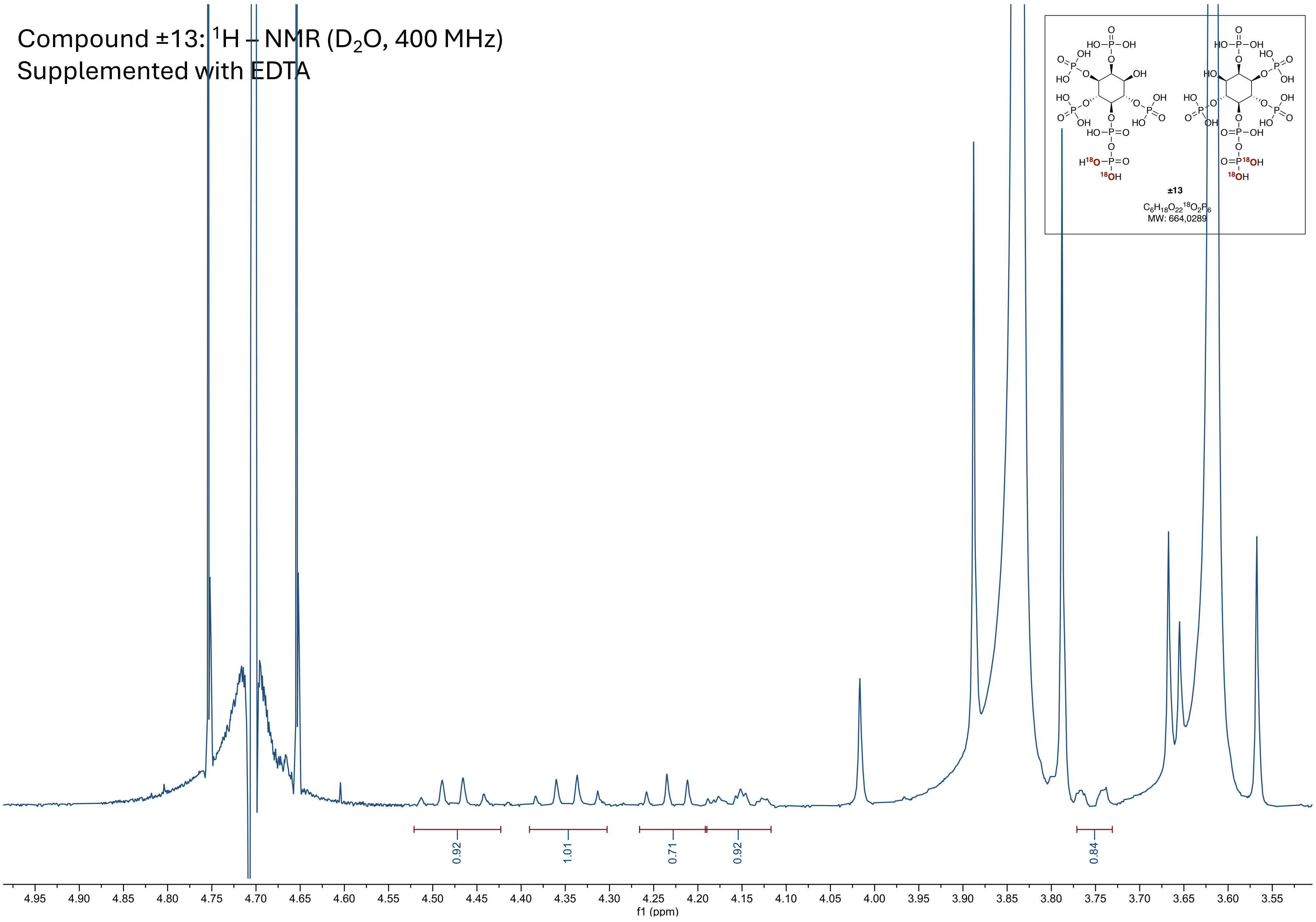

Compound ±13: $^{31}P\{^{1}H\}$ – NMR (D_2O, 162 MHz) Supplemented with ETDA

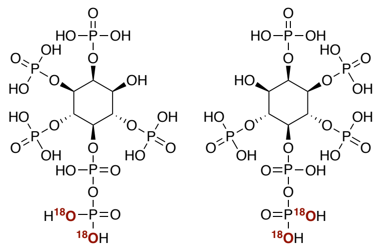

±13

$C_6H_{18}O_{22}{}^{18}O_2P_6$

MW: 664,0289

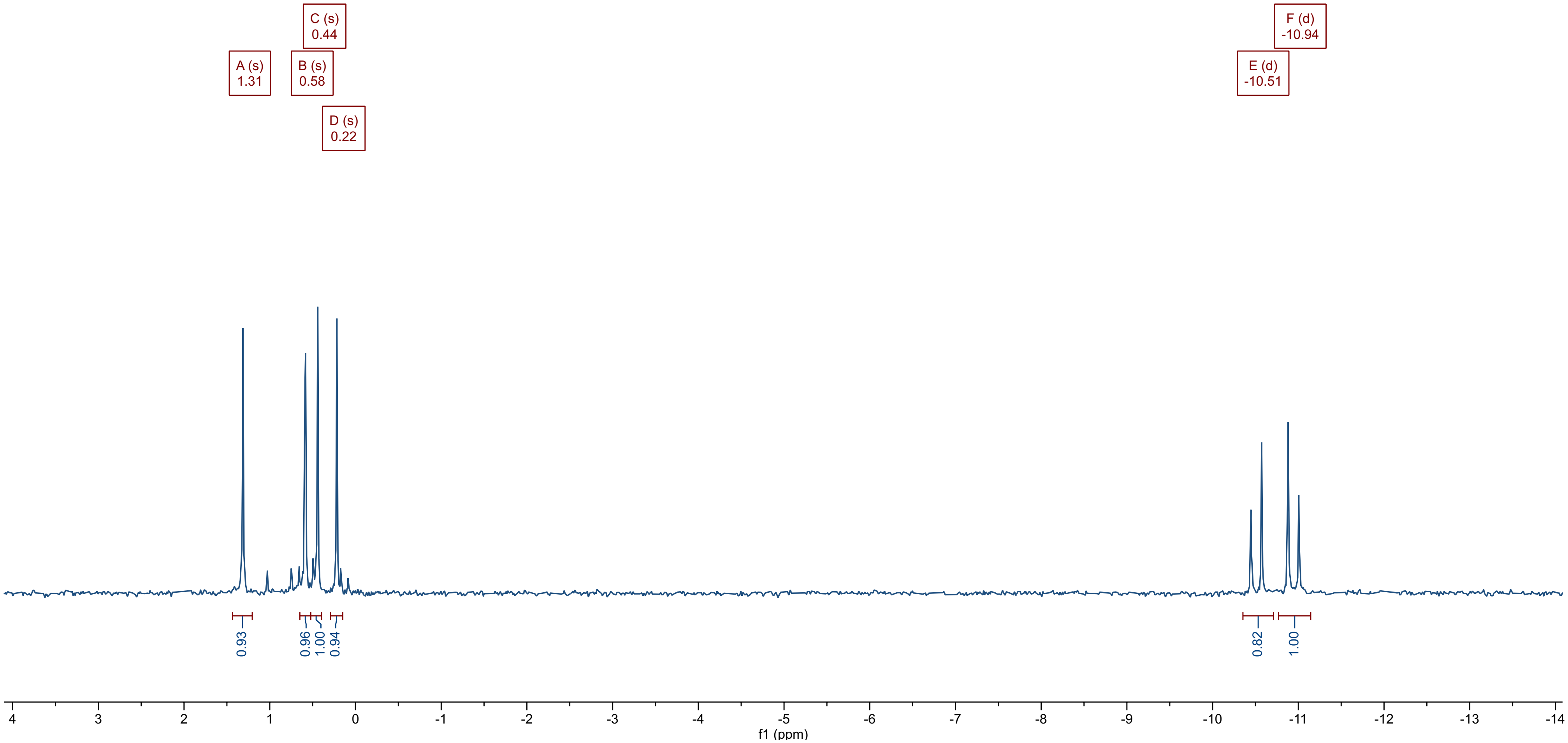

Compound 14a: ^{1}H – NMR (D_2O, 400 MHz)
Supplemented with TFA

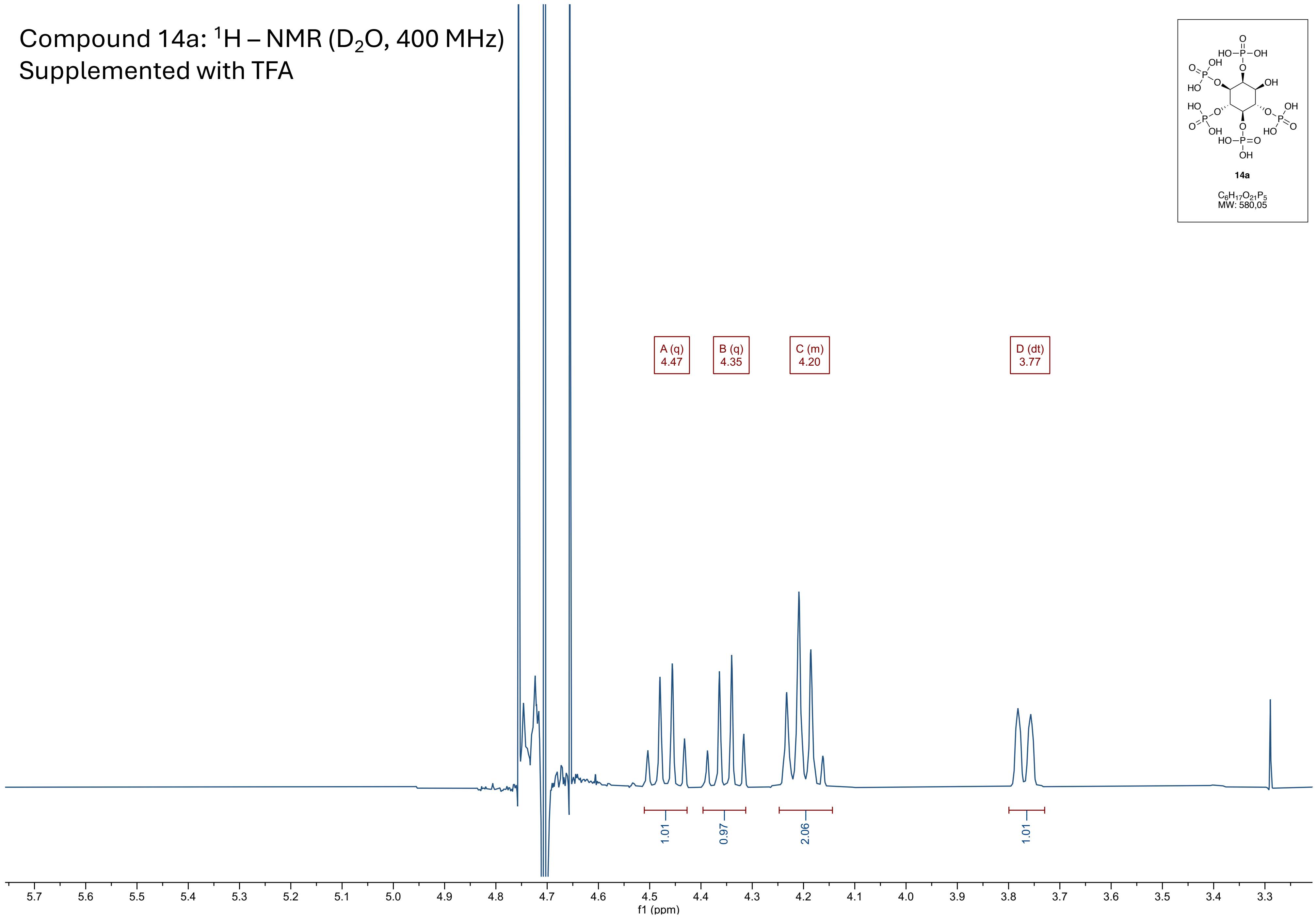

Compound 14a: $^{31}P\{^{1}H\}$ – NMR (D_2O, 162 MHz) Supplemented with TFA

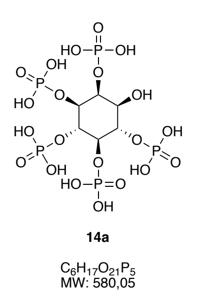

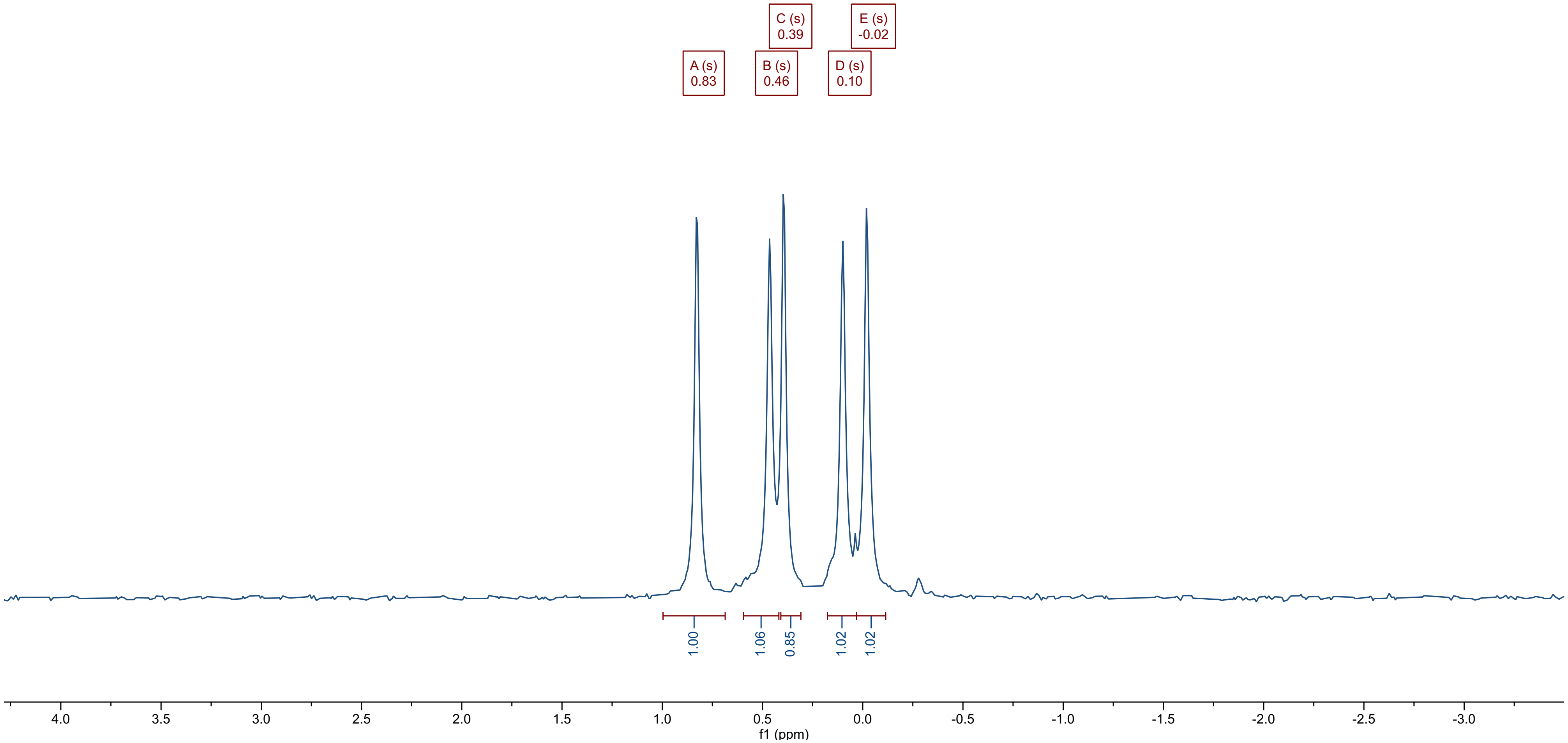

Compound ±16: ^{1}H – NMR ($CDCl_3$, 400 MHz)

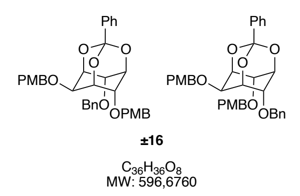

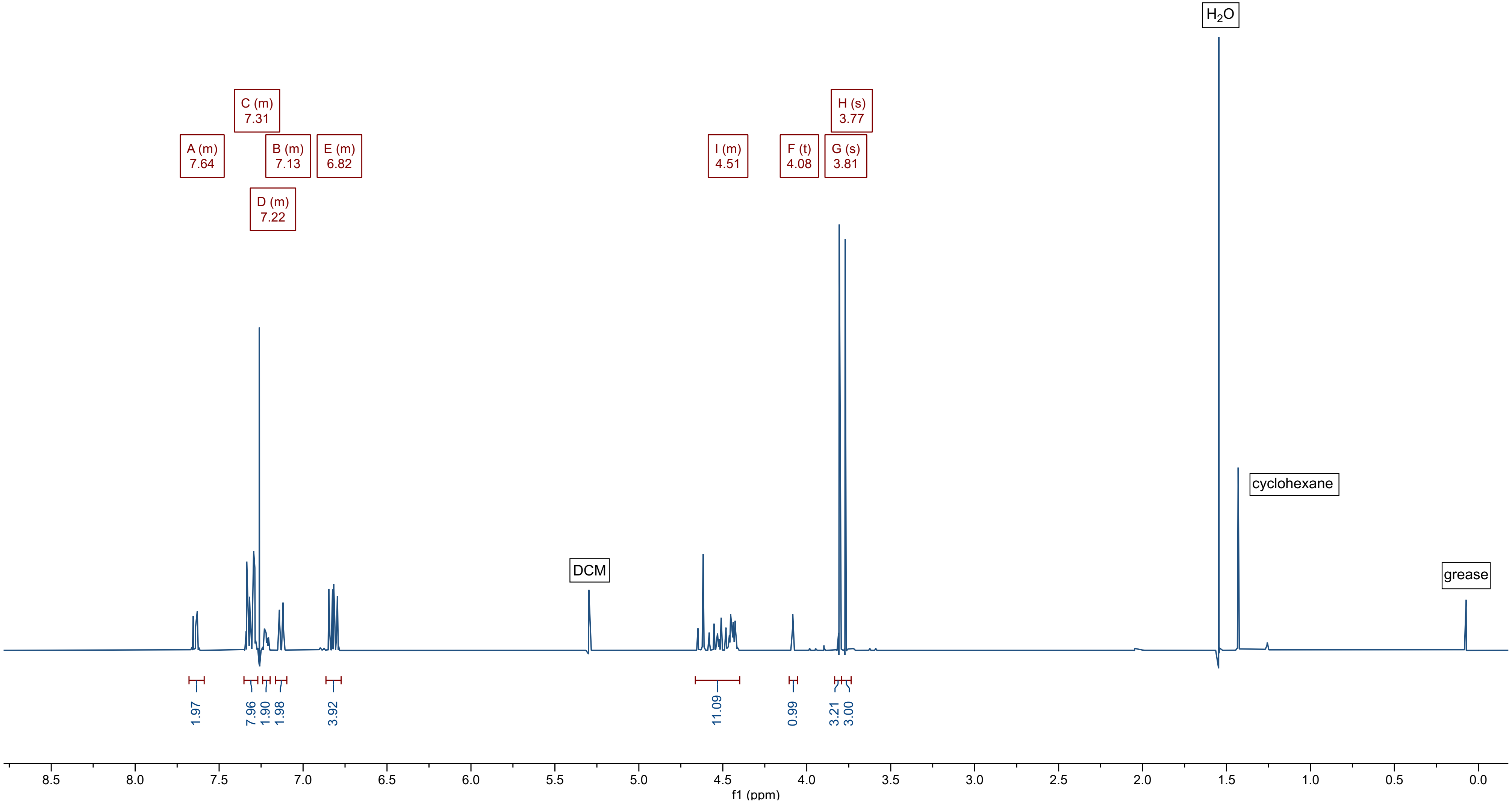

Compound ±16: $^{13}C\{^1H\}$ – NMR ($CDCl_3$, 101 MHz)

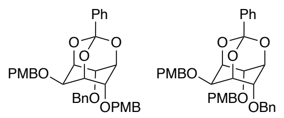

±16
$C_{36}H_{36}O_8$
MW: 596,6760

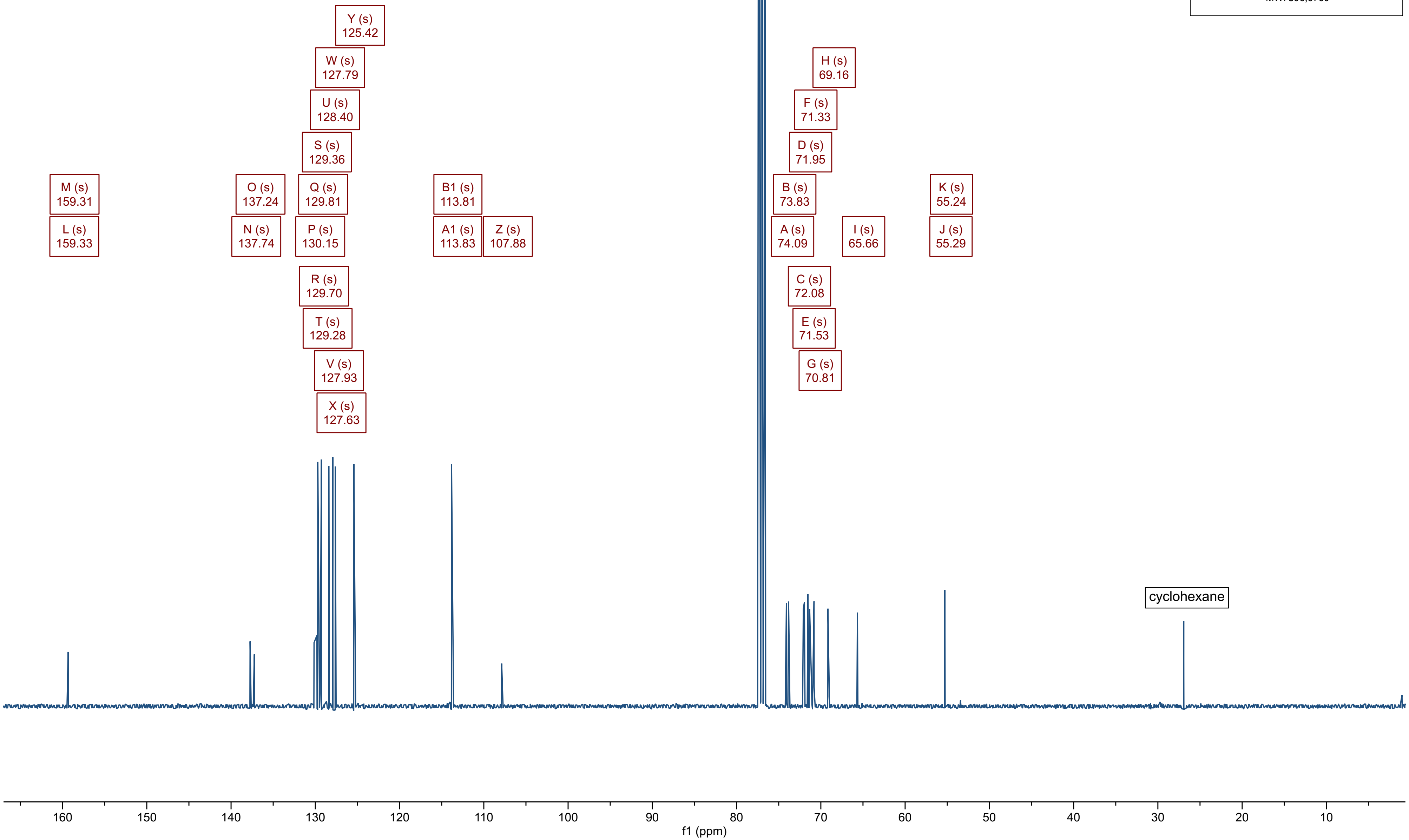

Compound ±17: ^{1}H – NMR ($CDCl_3$, 400 MHz)

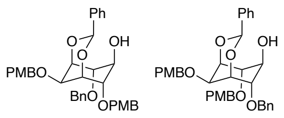

±17

$C_{36}H_{38}O_8$
MW: 598,6920

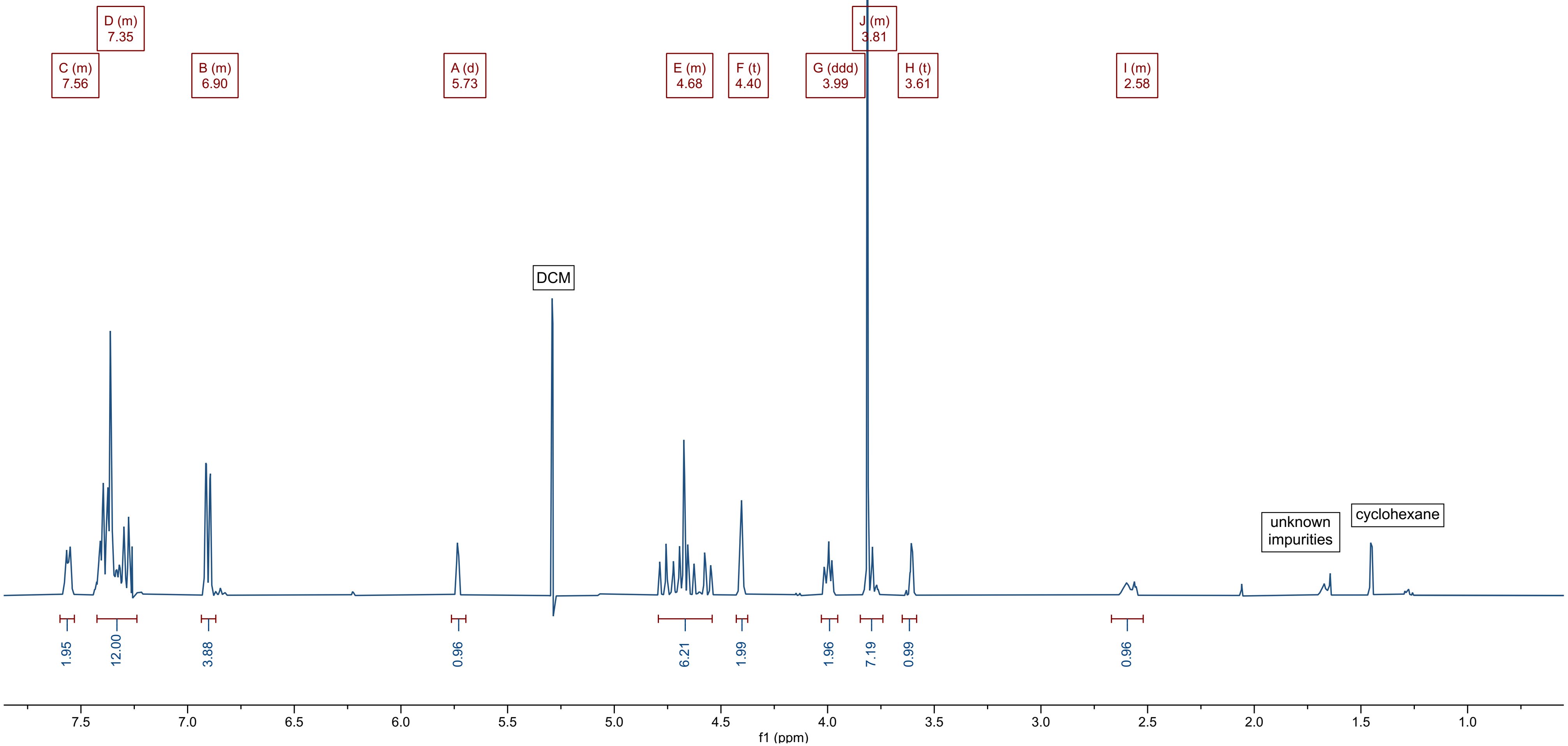

Compound ±17: $^{13}C\{^1H\}$ – NMR ($CDCl_3$, 101 MHz)

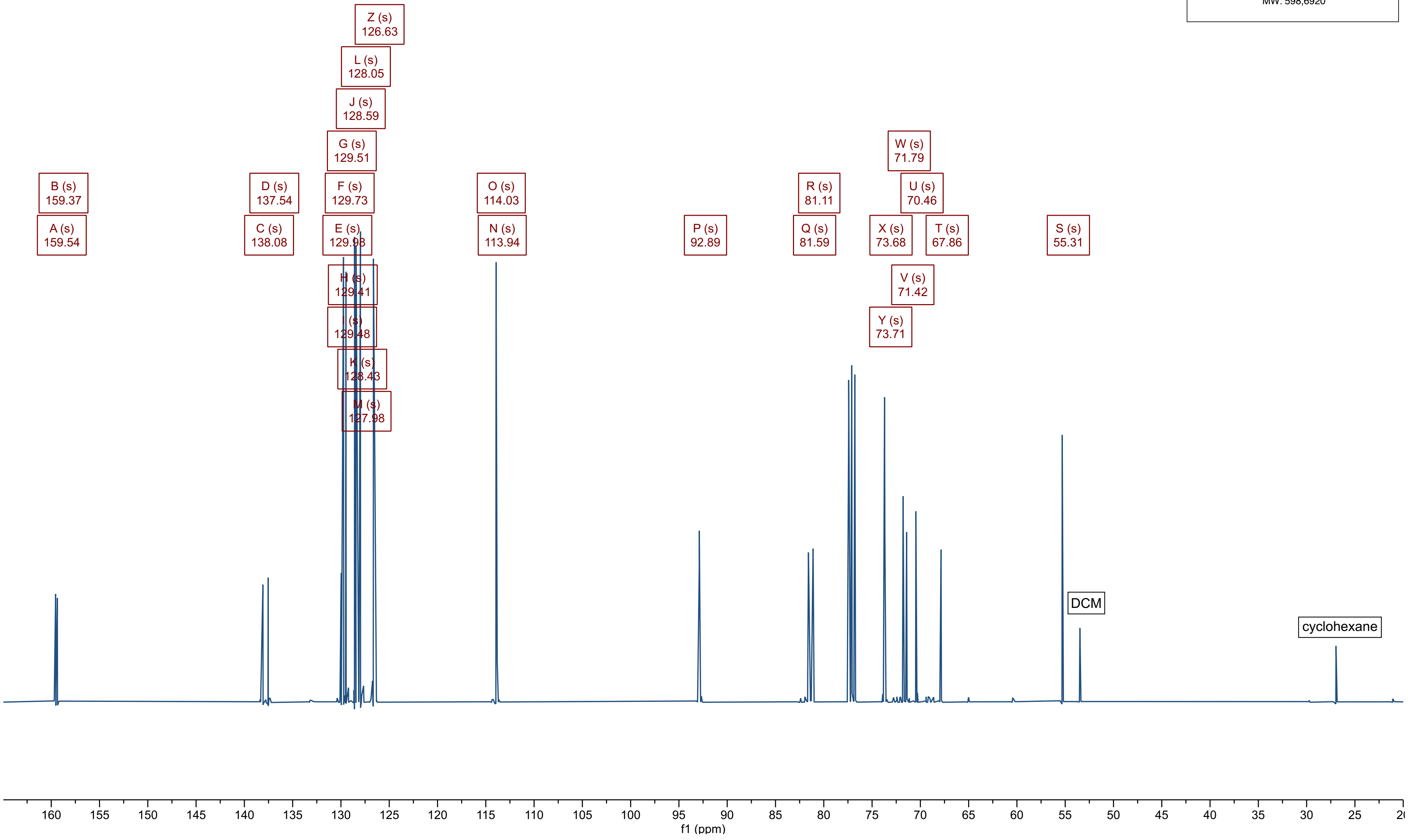

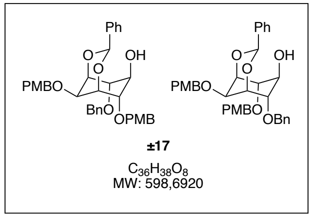

Compound ±19: ^{1}H – NMR ($CDCl_3$, 400 MHz)

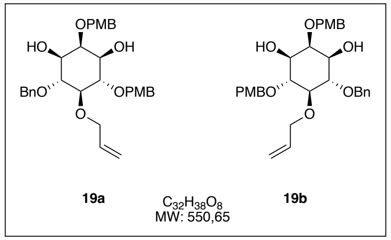

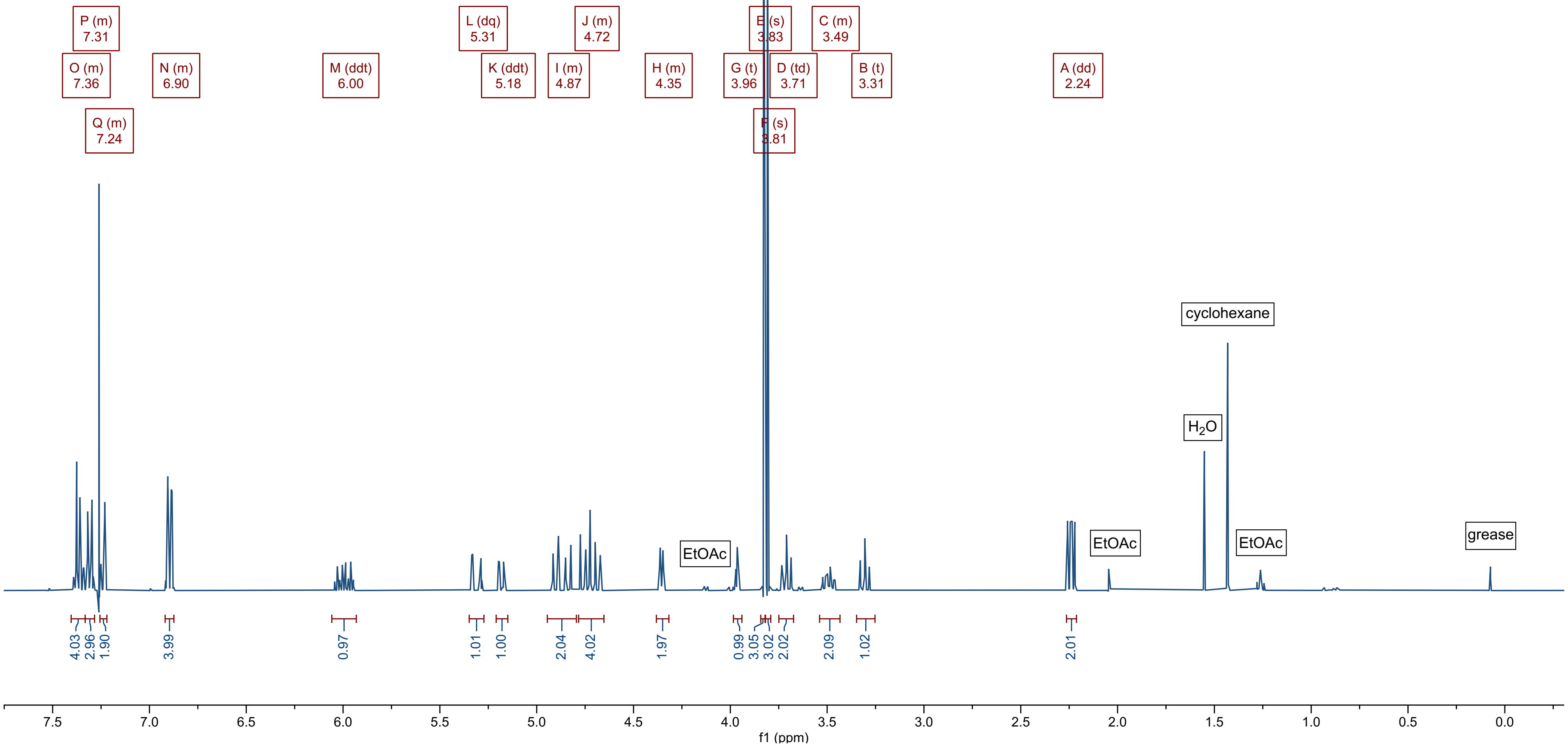

Compound ±19: $^{13}C\{^1H\}$ – NMR ($CDCl_3$, 101 MHz)

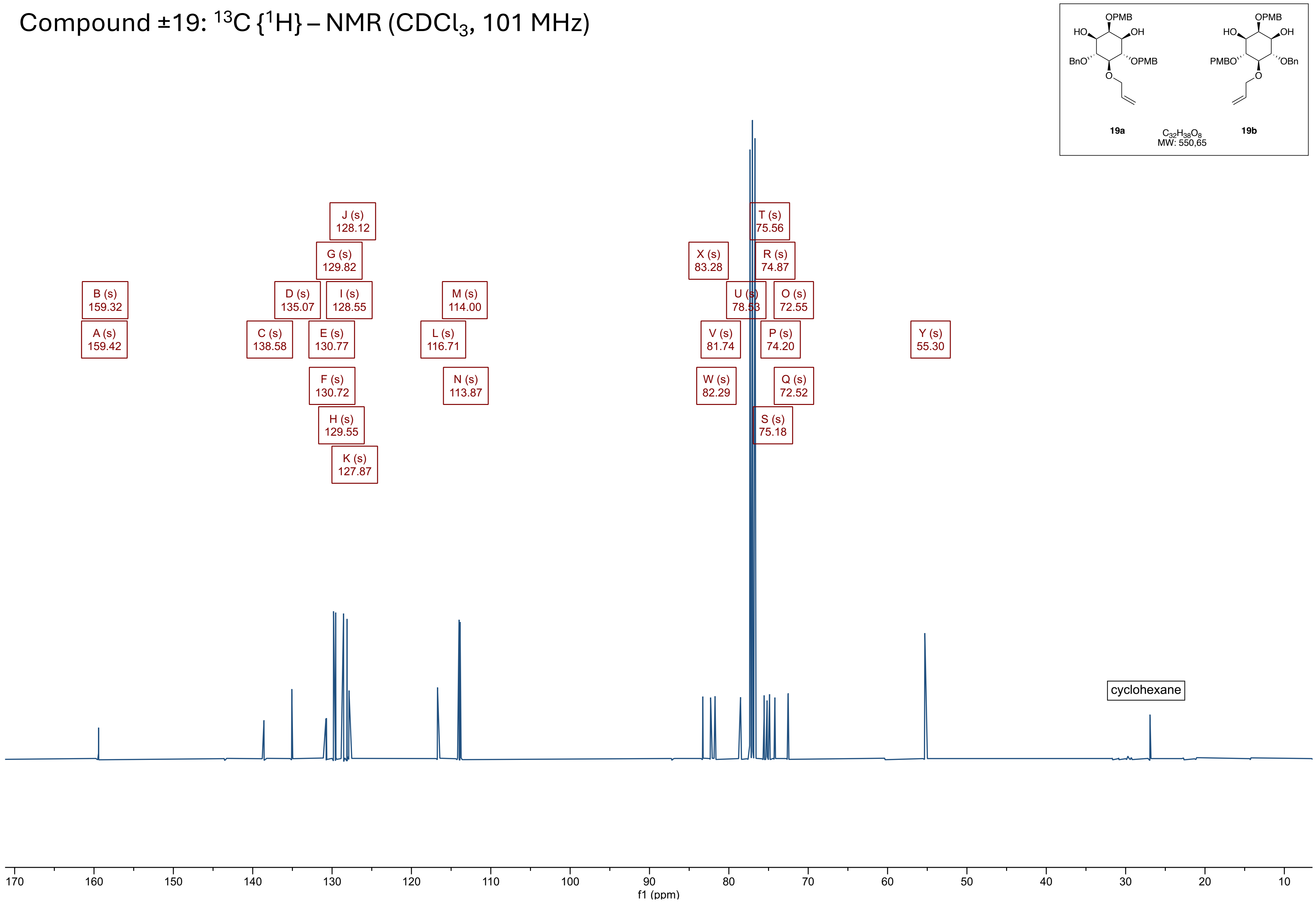

Compound ±21: ^{1}H – NMR ($CDCl_3$, 400 MHz)

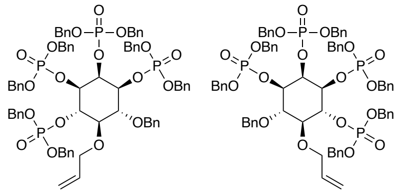

±21

$C_{72}H_{74}O_{18}P_4$
MW: 1351,26

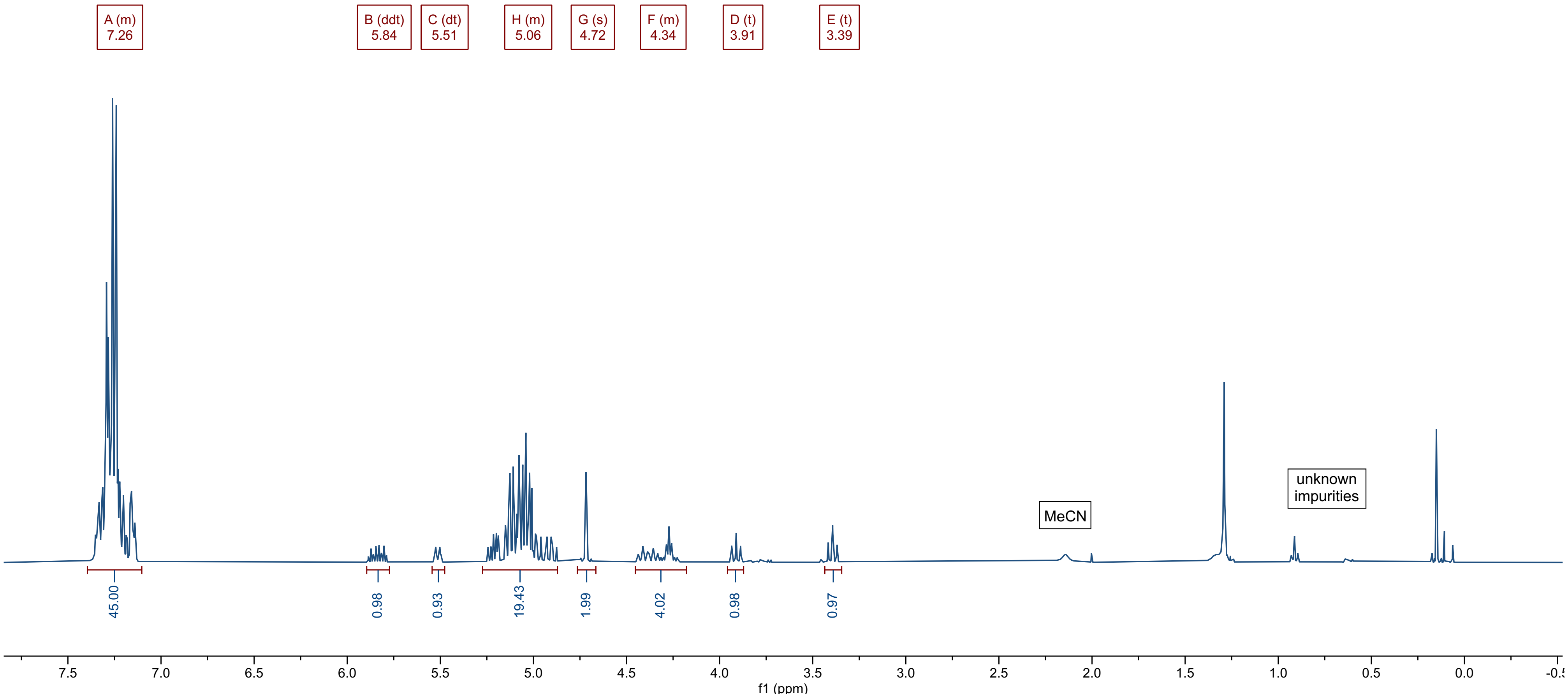

Compound ±21: $^{31}P\{^{1}H\}$ – NMR ($CDCl_3$, 162 MHz)

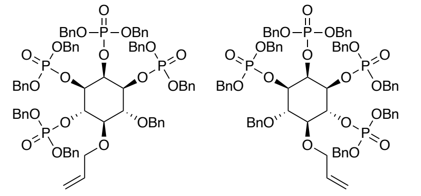

±21

$C_{72}H_{74}O_{18}P_4$
MW: 1351,26

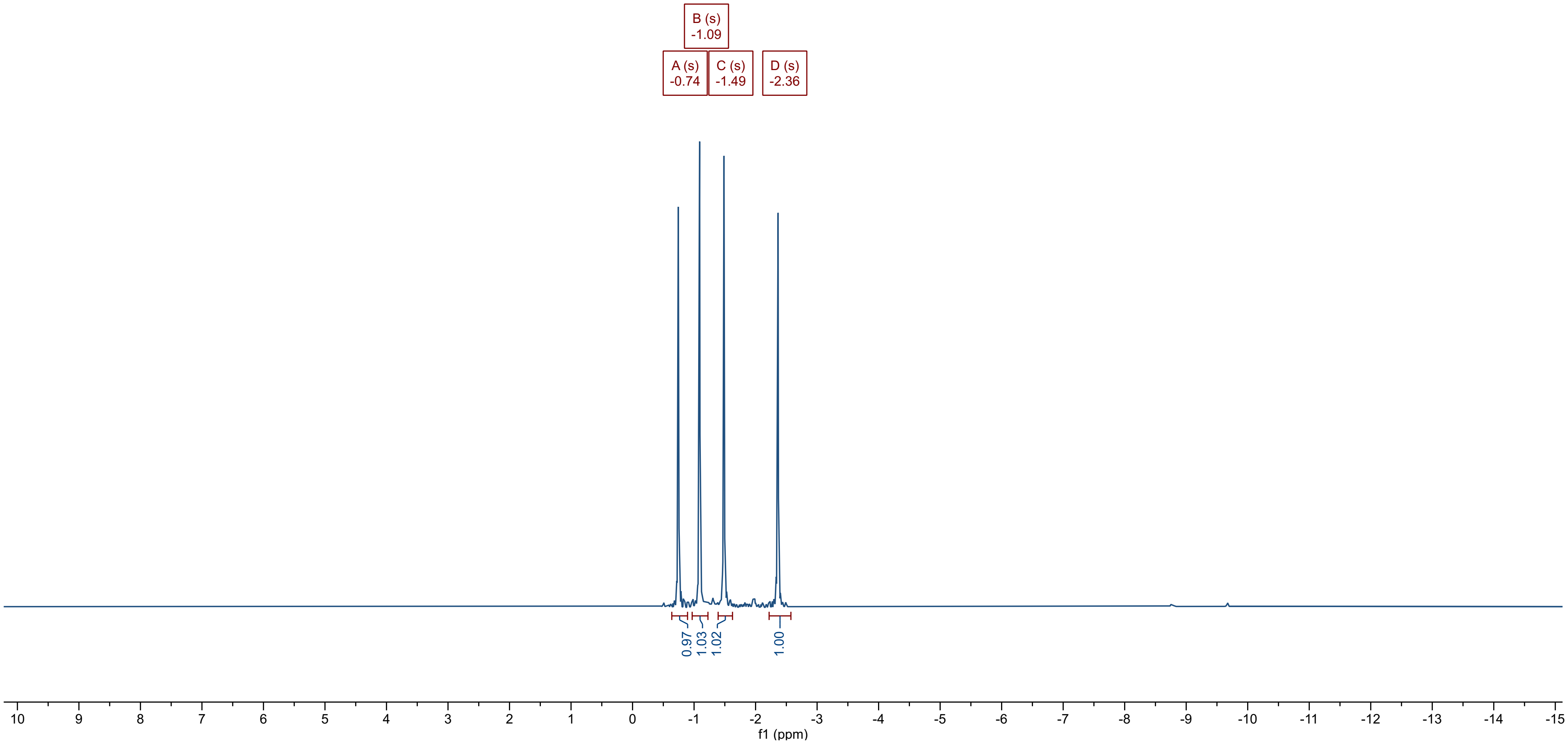

Compound ±21: $^{13}C\{^{1}H\}$ – NMR ($CDCl_3$, 101 MHz)

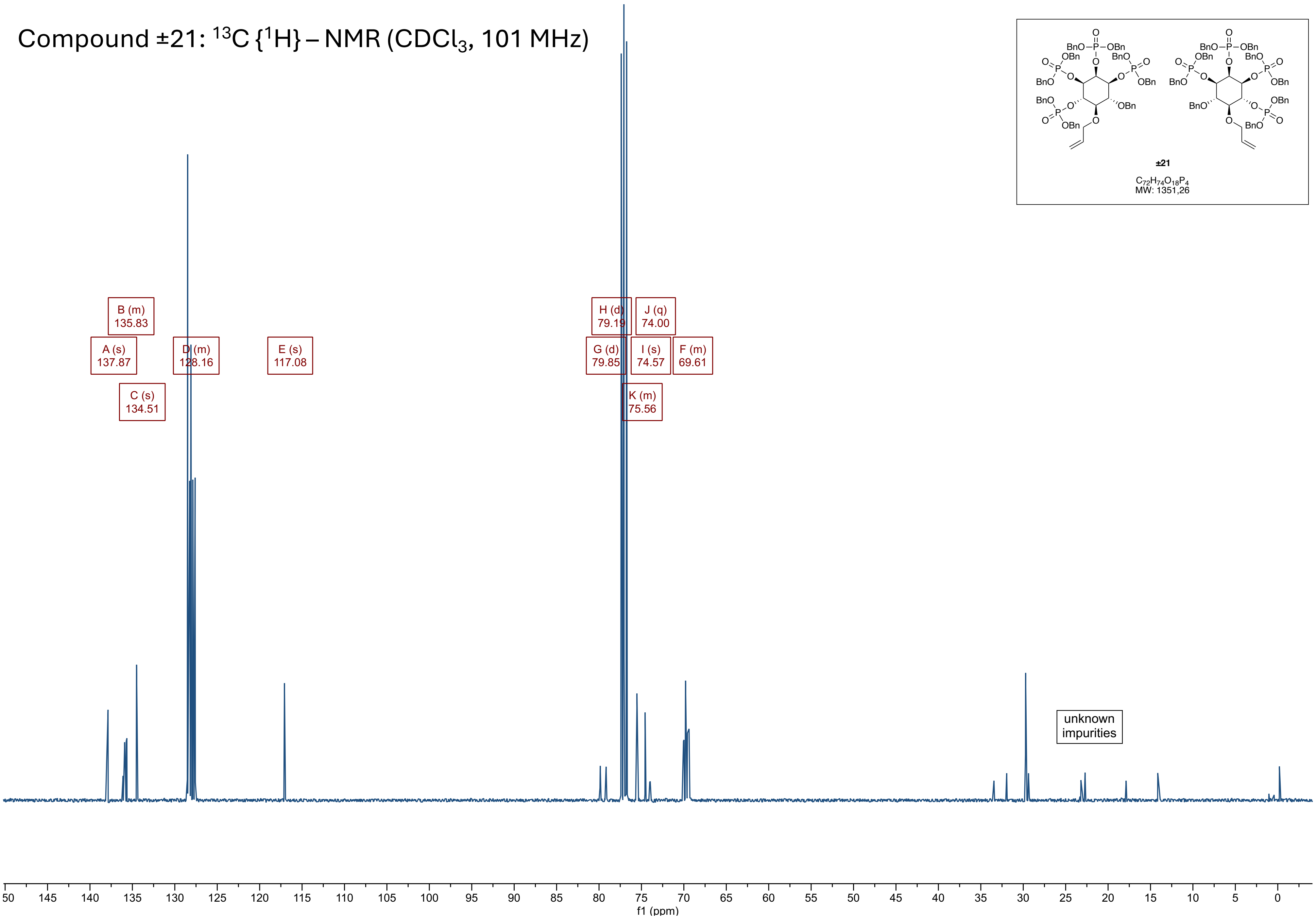

Compound ±23: ^{1}H – NMR ($CDCl_3$, 400 MHz)

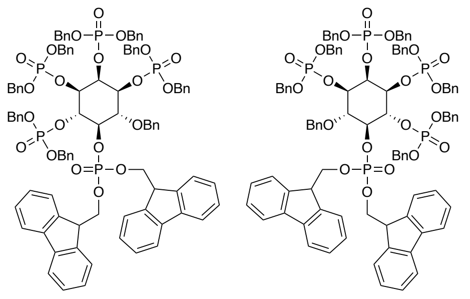

±23

$C_{97}H_{91}O_{21}P_5$
MW: 1747,64

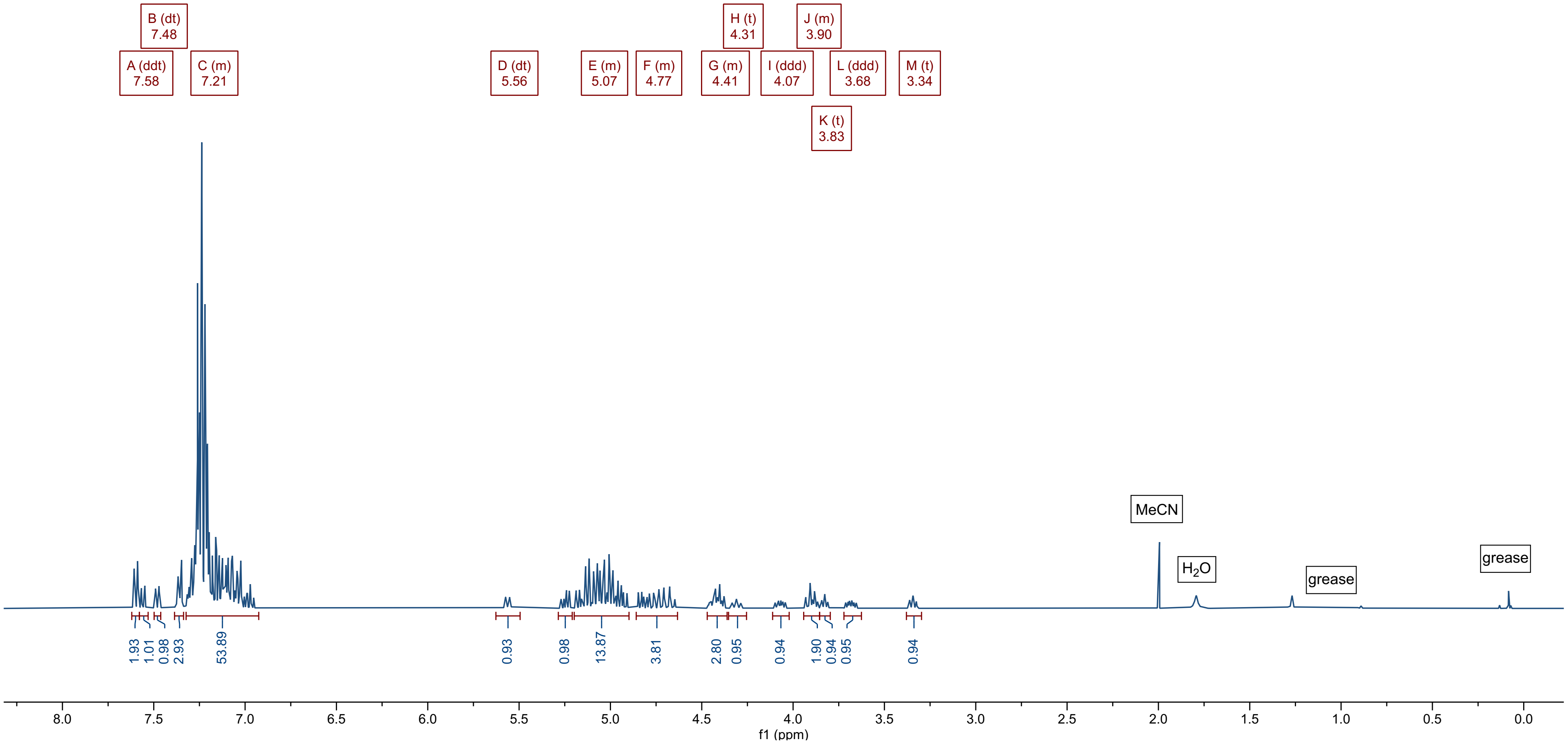

Compound ±23: $^{31}P\{^{1}H\}$ – NMR ($CDCl_3$, 162 MHz)

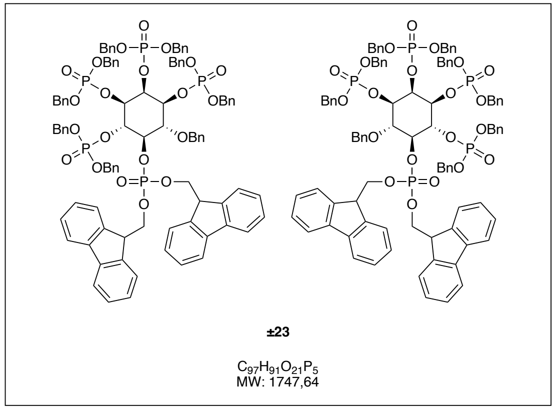

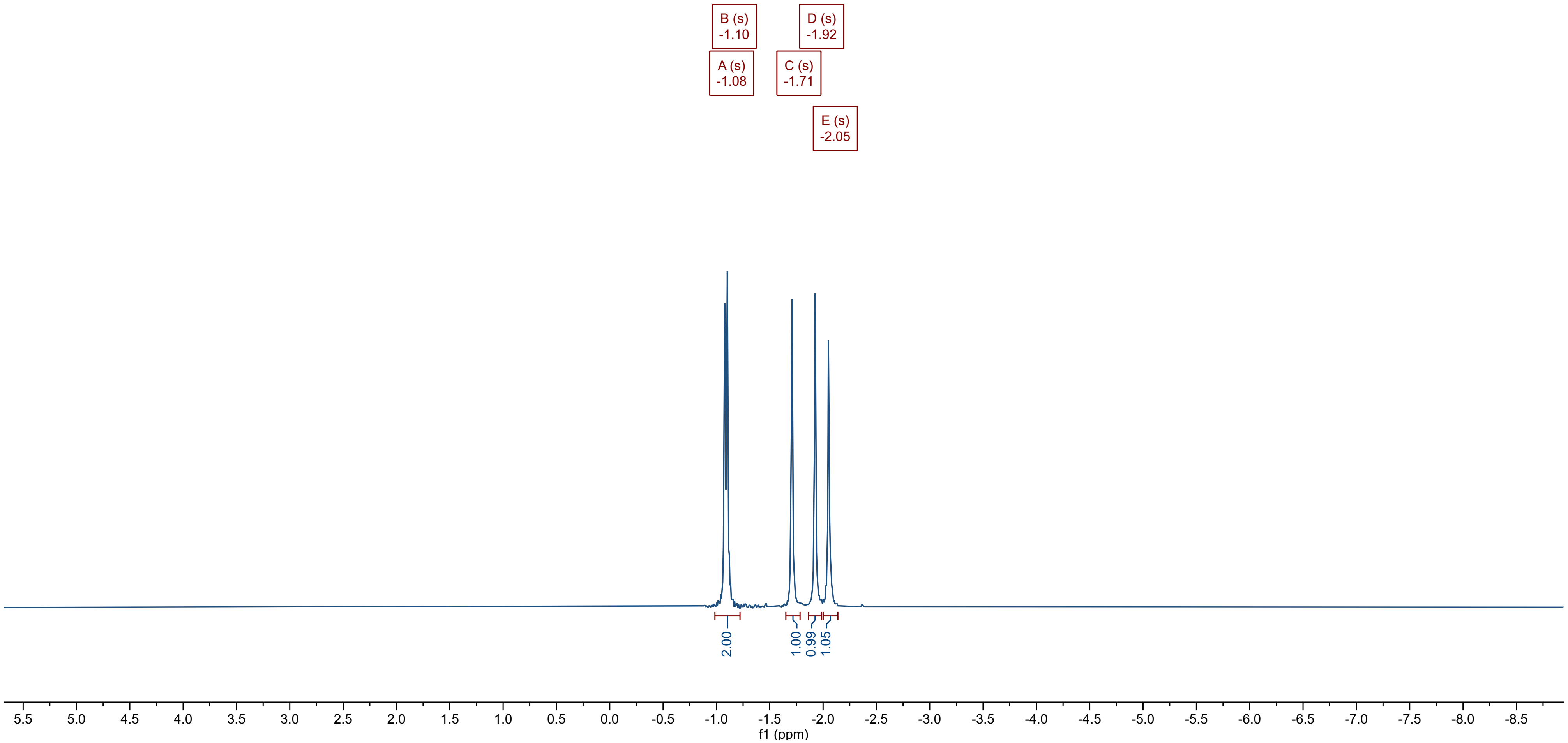

Compound ±23: $^{13}C\{^{1}H\}$ – NMR ($CDCl_3$, 101 MHz)

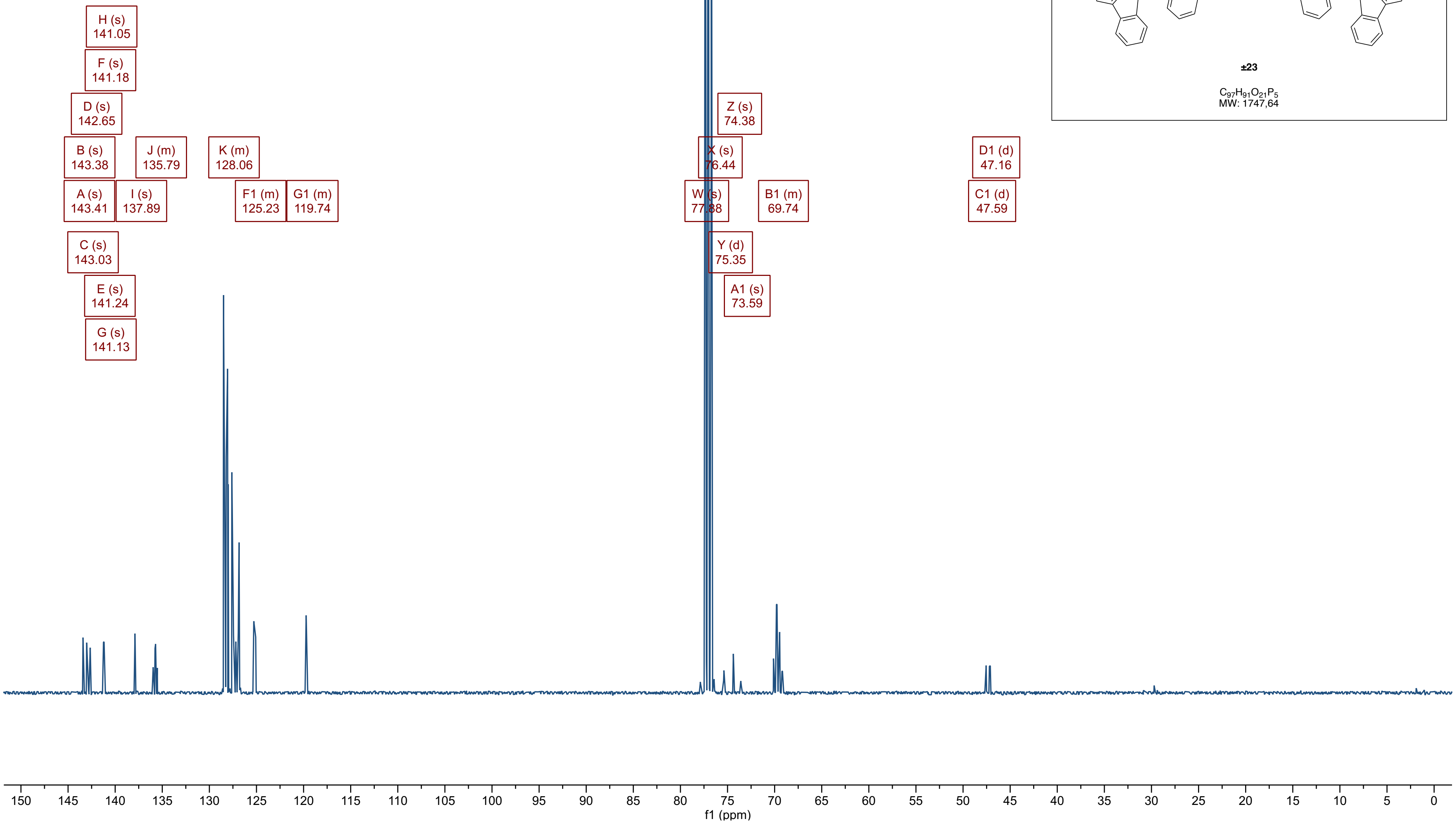

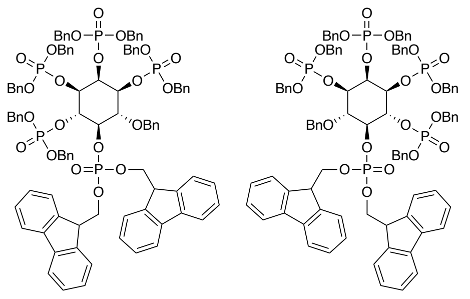

$C_{97}H_{91}O_{21}P_5$
MW: 1747,64

Compound ±24: ^{1}H – NMR ($CDCl_3$, 400 MHz)

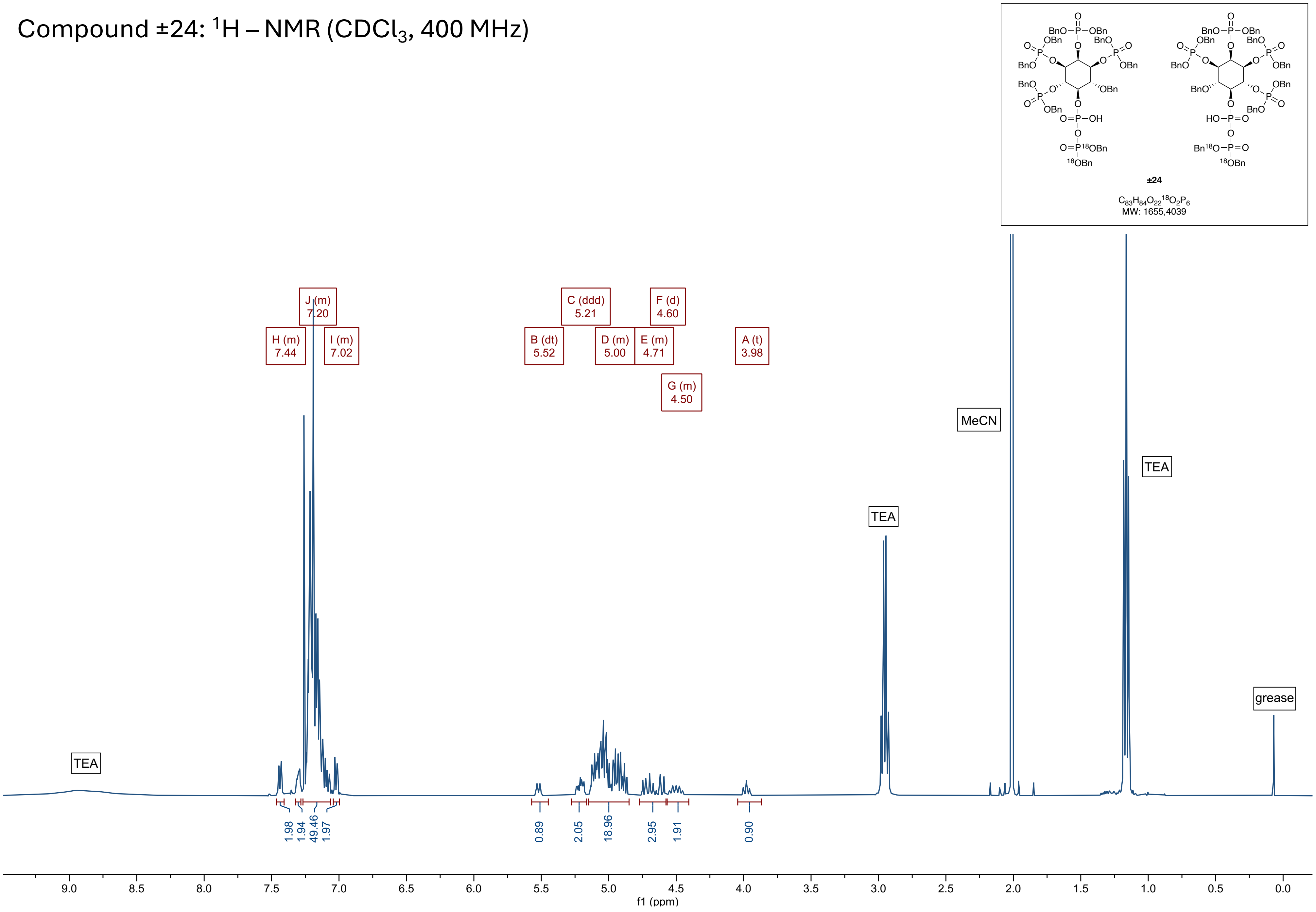

Compound ±24: $^{31}P\{^1H\}$ – NMR ($CDCl_3$, 162 MHz)

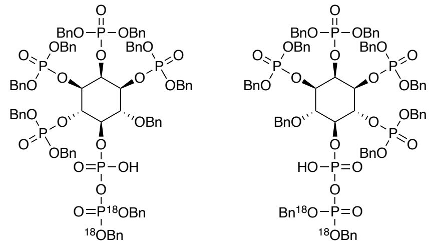

±24

$C_{83}H_{84}O_{22}{}^{18}O_2P_6$
MW: 1655,4039

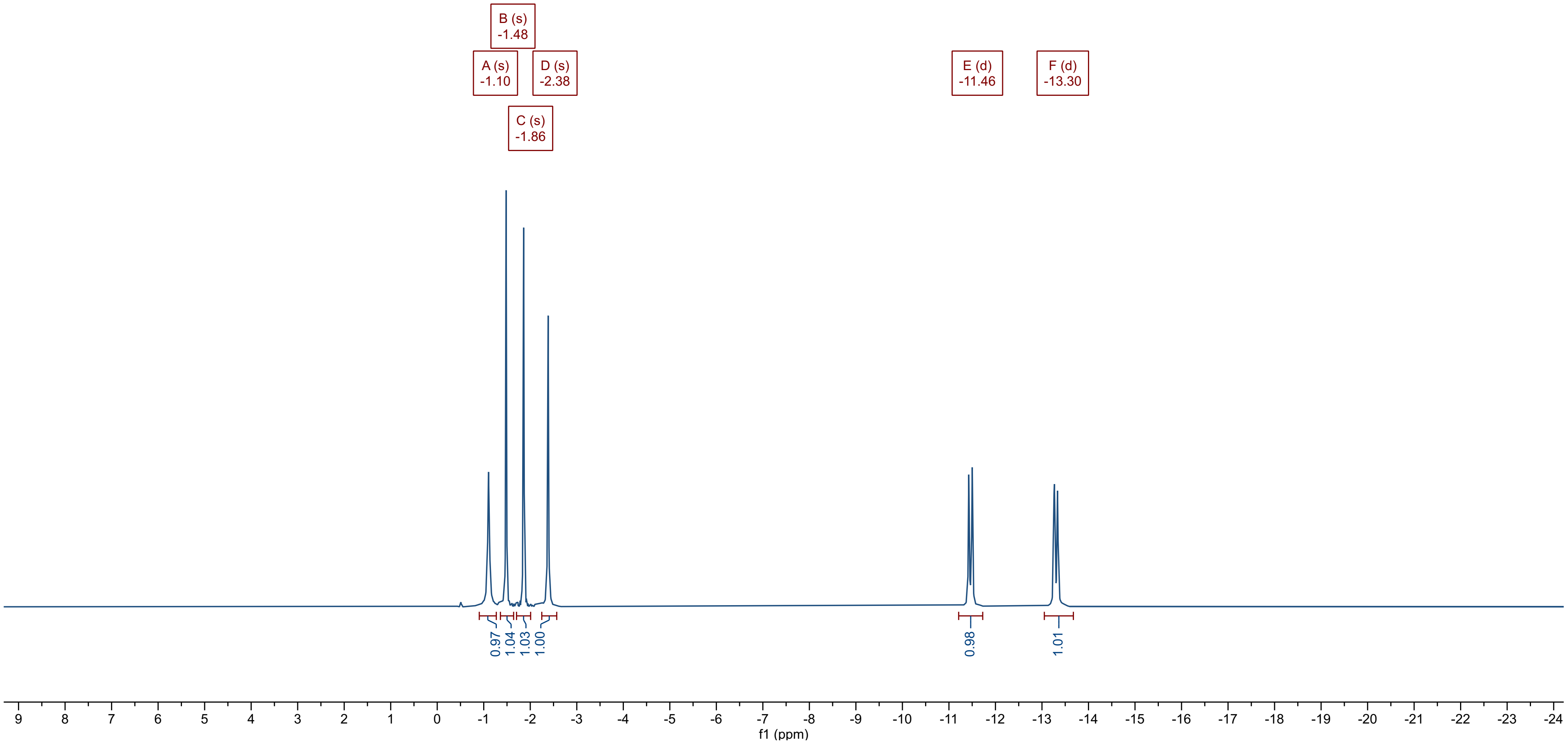

Compound ±24: $^{13}C\{^{1}H\}$ – NMR ($CDCl_3$, 101 MHz)

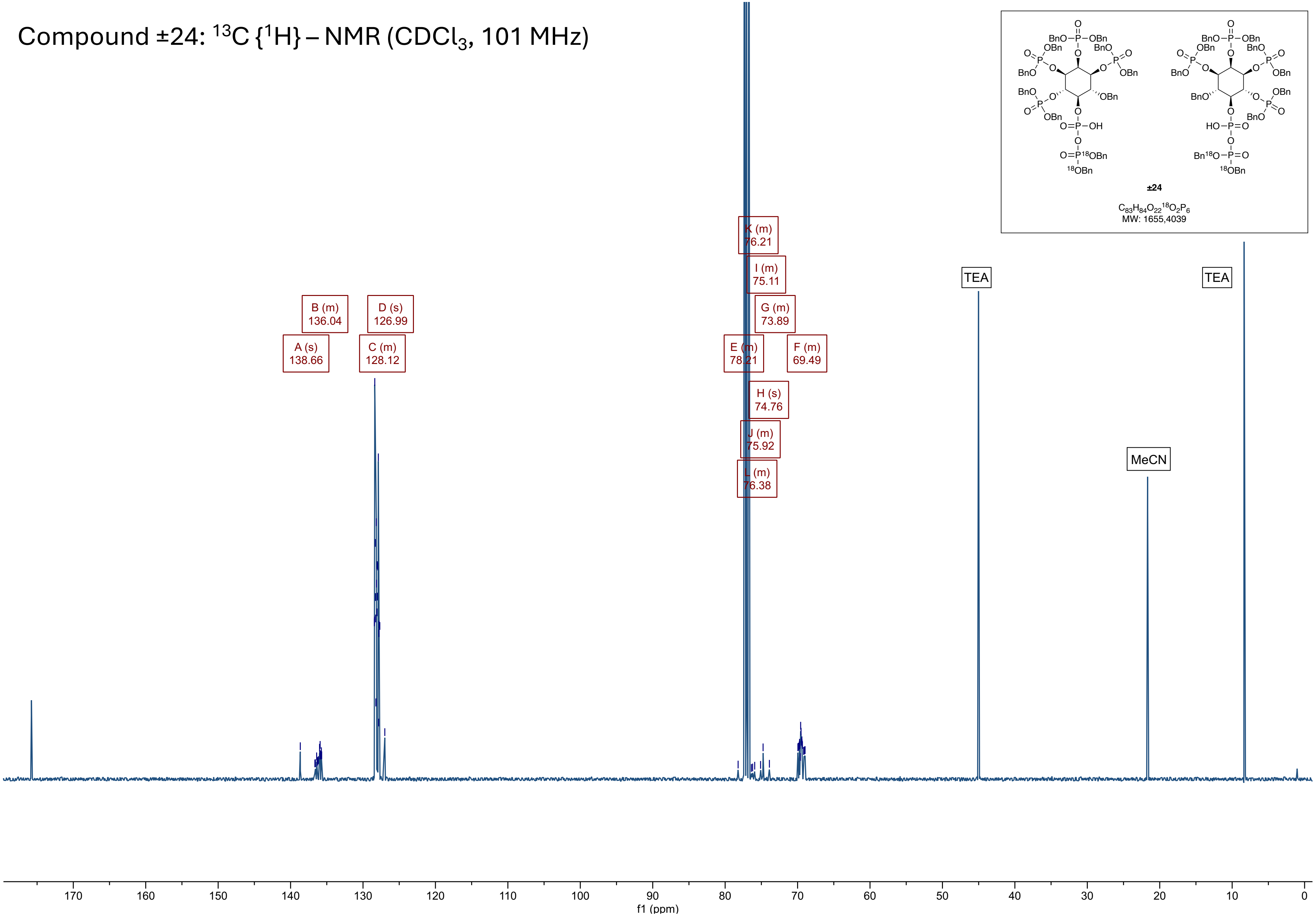

Compound ±25: $^{1}H – NMR (D_2O, 400 MHz)$

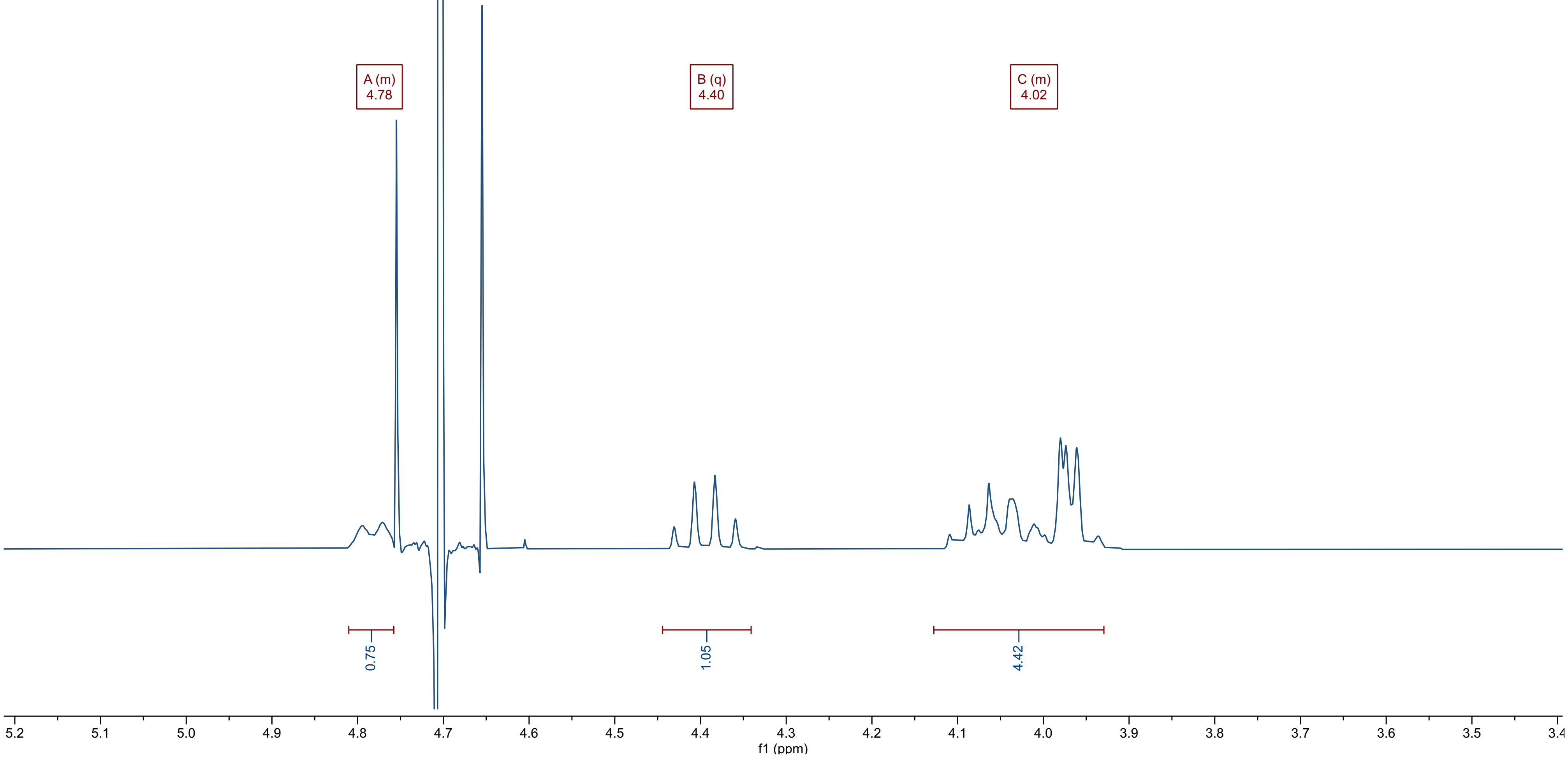

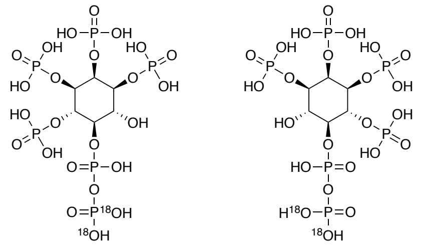

±25

$C_6H_{18}O_{22}{}^{18}O_2P_6$

MW: 664,0289

Compound ±25: $^{31}P\{^{1}H\}$ – NMR (D_2O, 162 MHz)

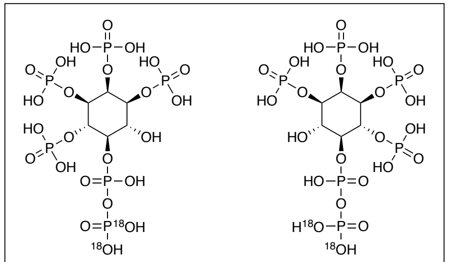

±25

$C_6H_{18}O_{22}{}^{18}O_2P_6$

MW: 664,0289

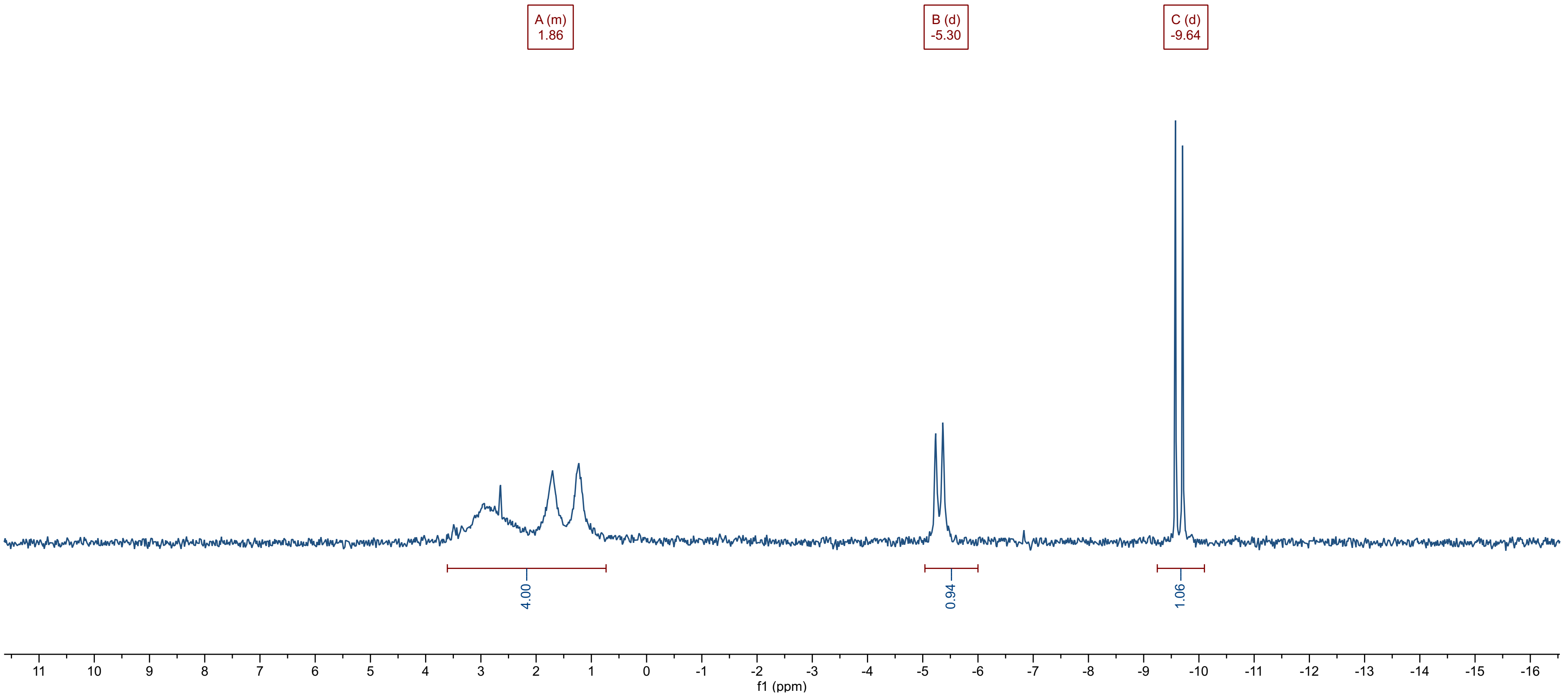

Compound ±25: ^{1}H – NMR (D_2O, 400 MHz)
Supplemented with EDTA

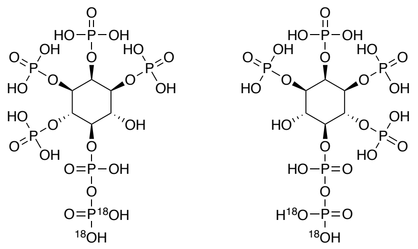

±25

$C_6H_{18}O_{22}{}^{18}O_2P_6$
MW: 664,0289

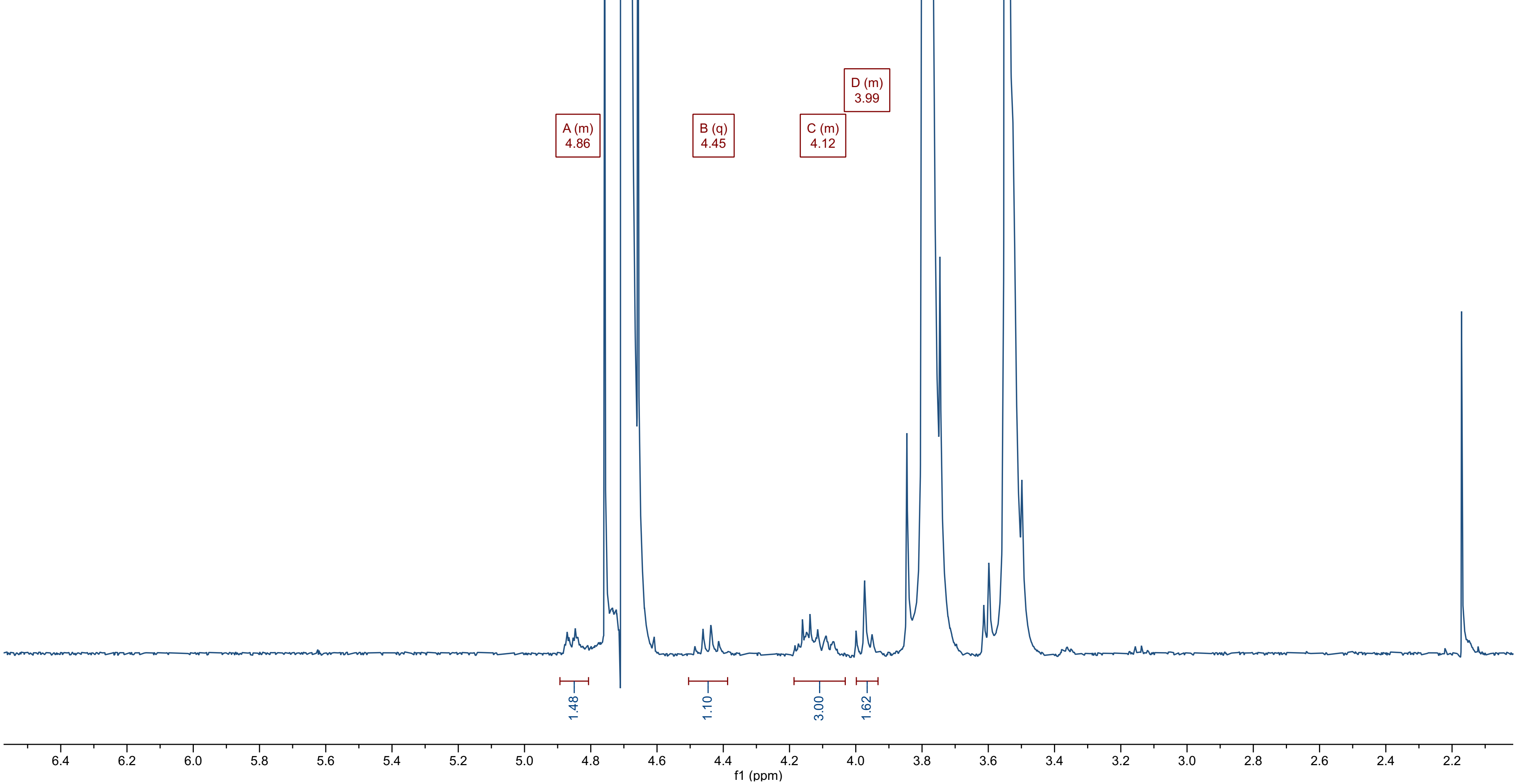

Compound ±25: $^{31}P\{^1H\}$ – NMR (D_2O, 162 MHz) Supplemented with ETDA

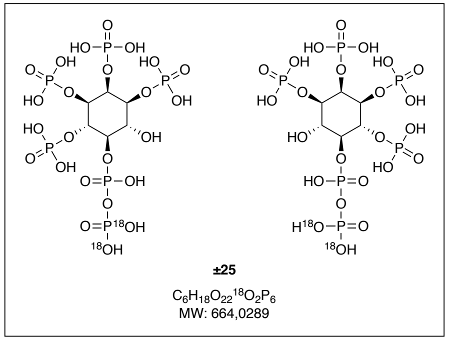

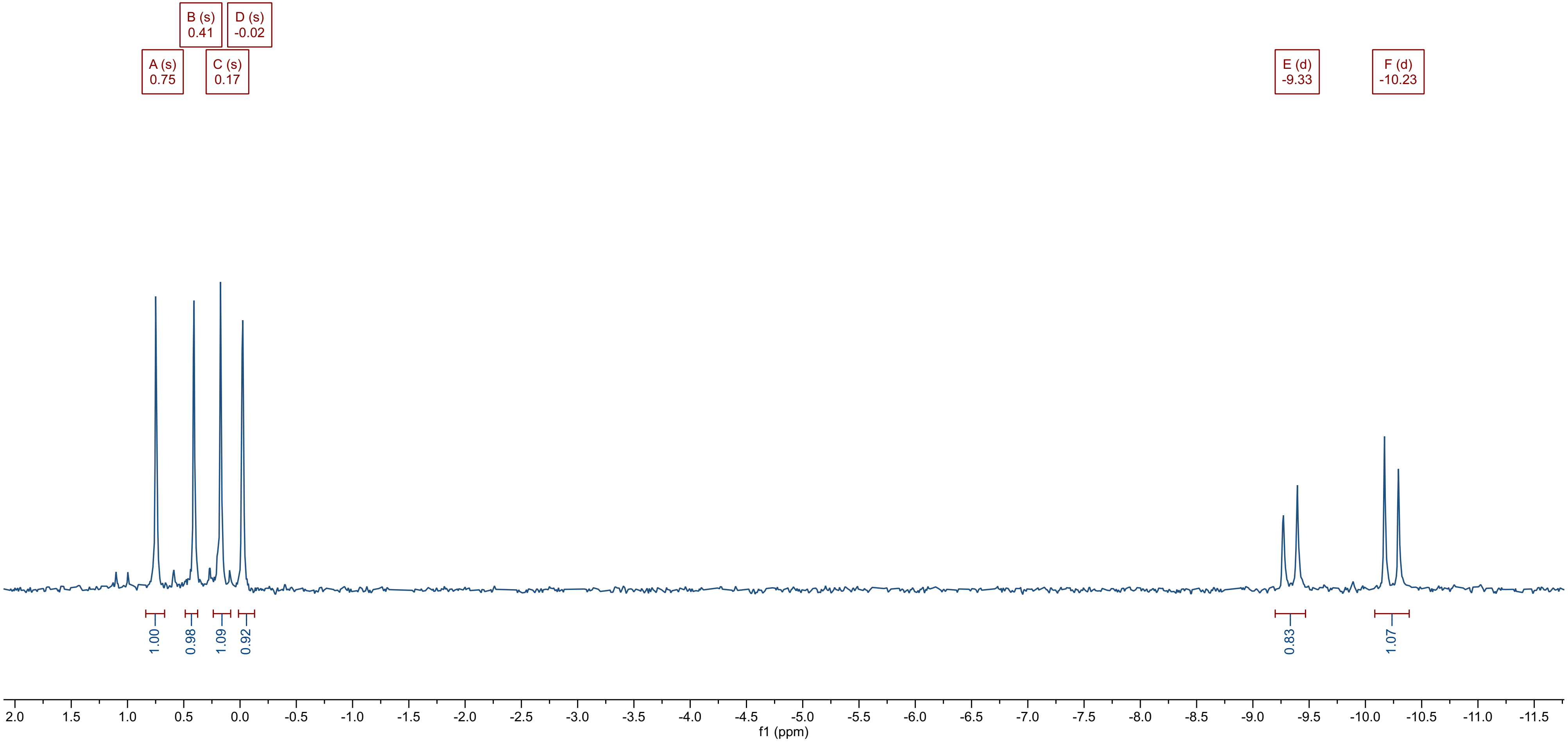

Compound ±25: edHSQC to identify ^{13}C peaks
Supplemented with EDTA

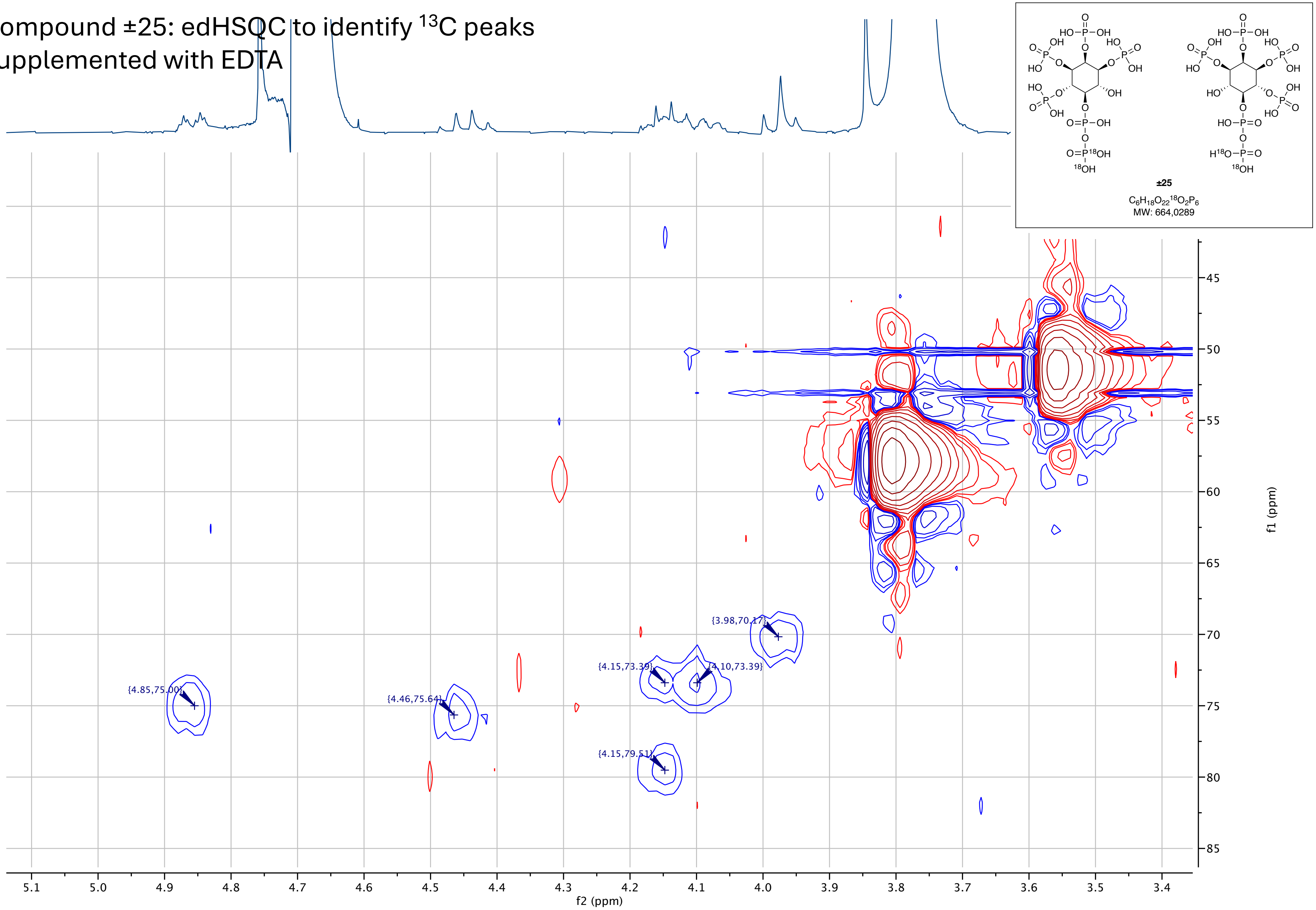

Compound 26a: ^{1}H – NMR (D_2O, 400 MHz) Supplemented with TFA

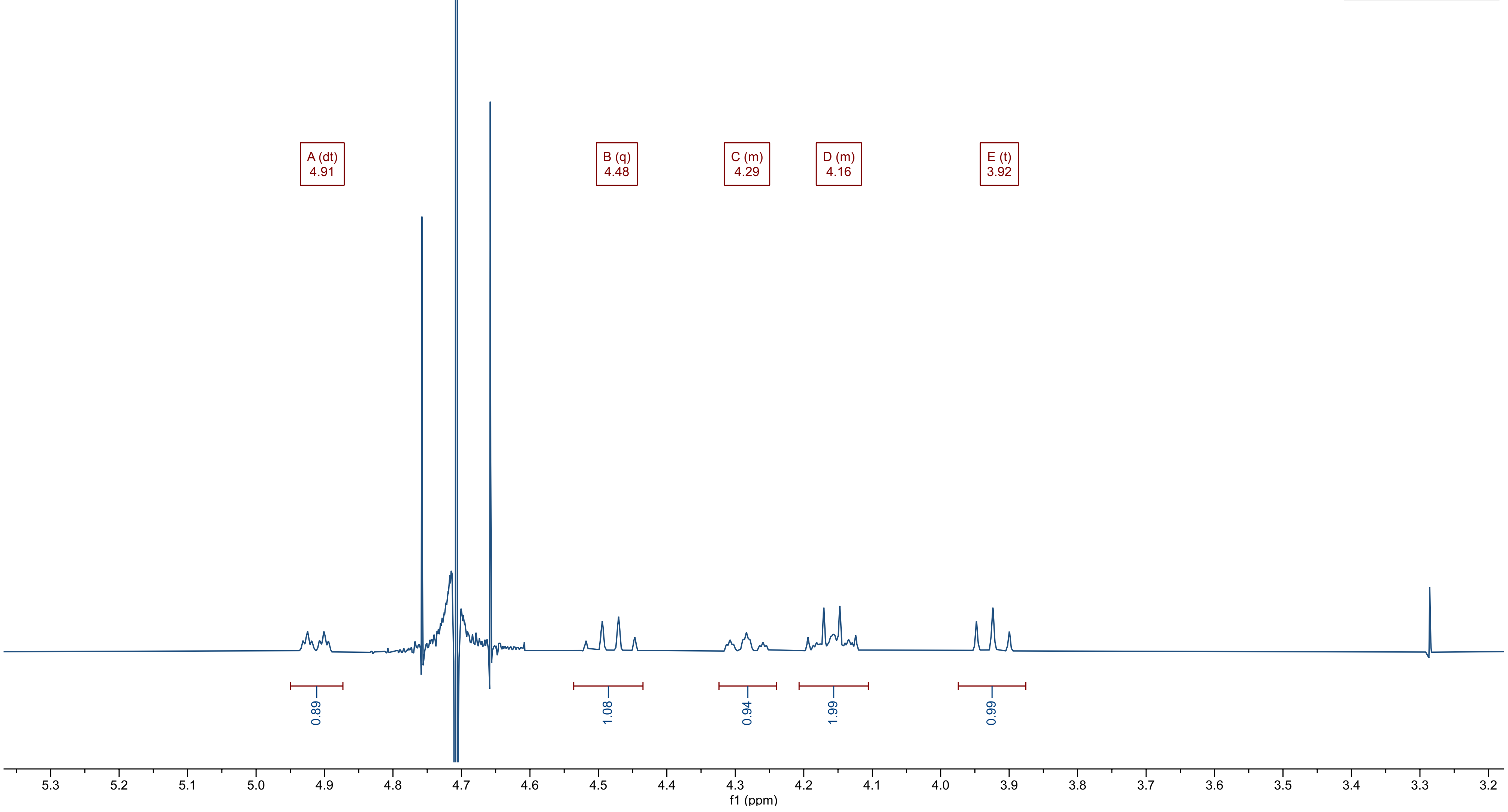

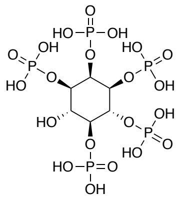

26a

$C_6H_{17}O_{21}P_5$
MW: 580,05

Compound 26a: $^{31}P\{^1H\}$ – NMR (D_2O, 162 MHz)
Supplemented with TFA

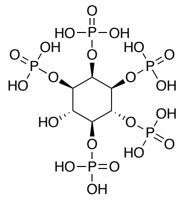

26a

$C_6H_{17}O_{21}P_5$
MW: 580,05

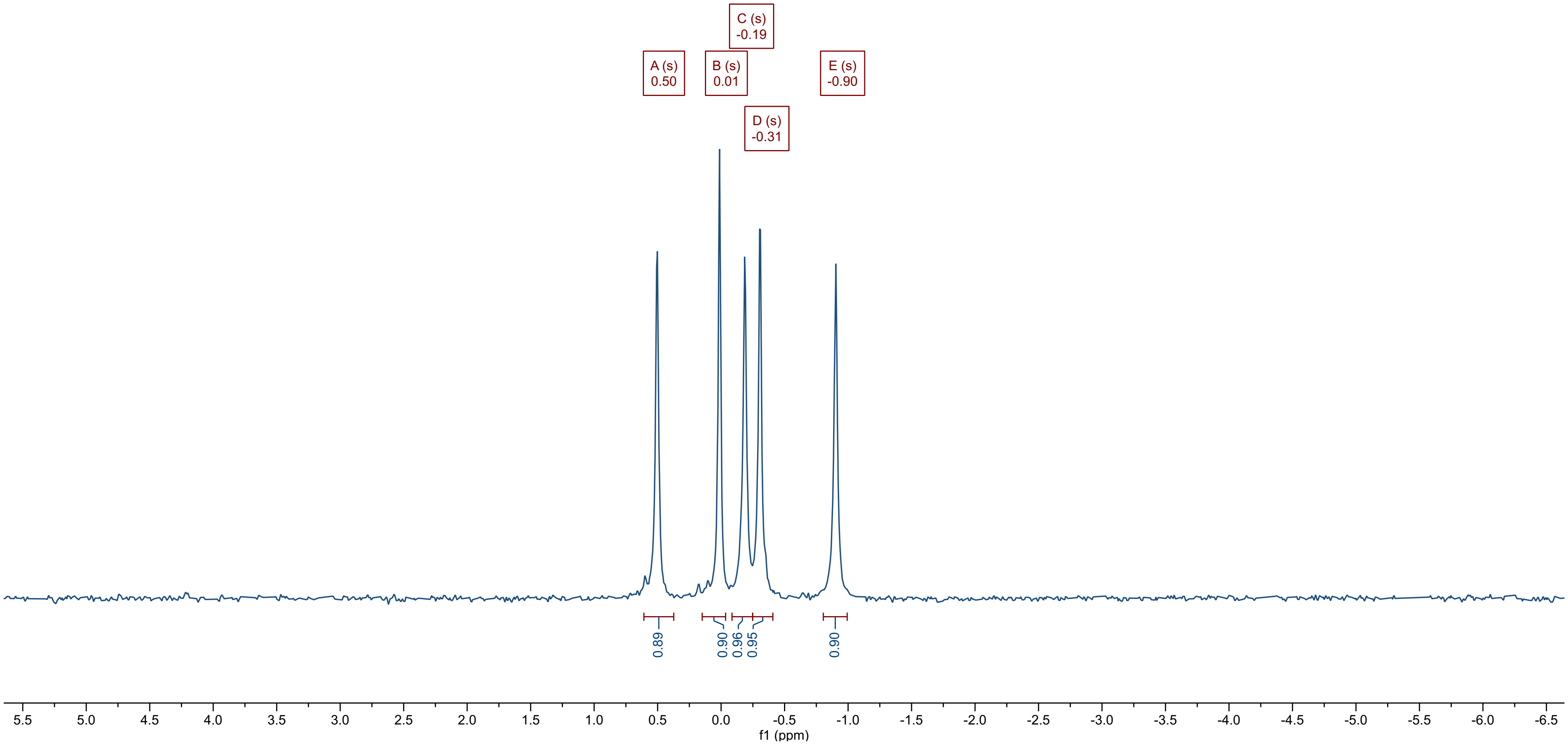

10 Mass Spectra, CE Electropherograms & Chiral HPLC

(Sorted according to molecule numbering)

Compound ±7: HRMS (ESI) Analysis

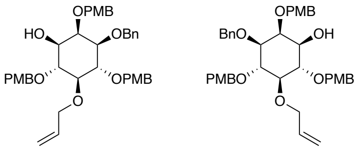

7a $C_{40}H_{46}O_9$ MW: 670,80 **7b**

Analysis Report

Agilent Trusted Answers

Sample Information

**Name**	kr-a509	**Data File Path**	D:\MassHunter\Data\2024\12\rijea66dis01.d
**Sample ID**		**Acq. Time (Local)**	12/10/2024 2:22:00 PM (UTC+01:00)
**Instrument**	QTOF-2	**Method Path (Acq)**	D:\MassHunter\Methods\Christoph\direkt0,2mlACN.m
**MS Type**	QTOF	**Version (Acq SW)**	6200 series TOF/6500 series Q-TOF 10.1 (48.0)
**Inj. Vol. (ul)**	3	**IRM Status**	Success
**Position**		**Method Path (DA)**	
**Plate Pos.**		**Target Source Path**	
**Operator**		**Result Summary**	

Sample Chromatograms

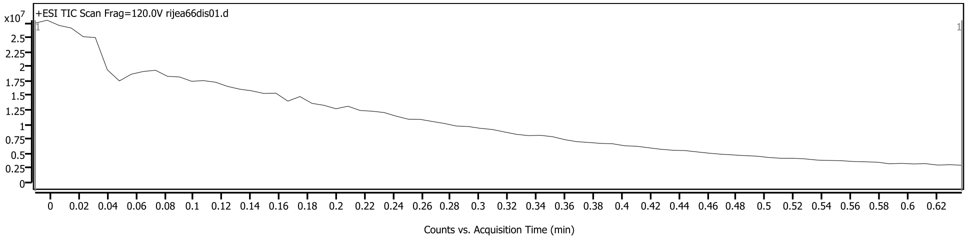

Sample Spectra

+ Scan (rt: 0.048-0.124 min) Sub

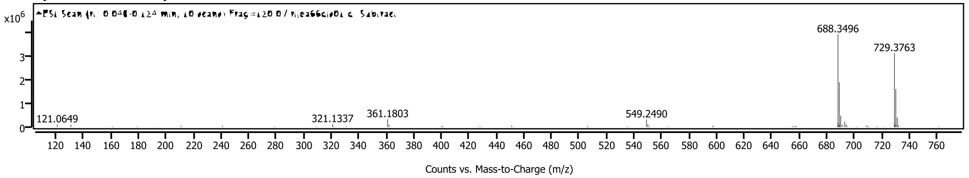

Spectrum Peaks

m/z	Z	Abund	Abund %	m/z (Calc)	Diff (ppm)	Ion Species	Formula	Ion Type
121.0649		110989	2.85	121.0648	0.61	(M+H)+	C8 H8 O	
131.0857		95727	2.46	131.0855	1.17	(M+H)+	C10 H10	
321.1337	1	123264	3.16	321.1333	1.22	(M+H)+	C17 H20 O6	
361.1803	1	331039	8.50	361.1798	1.40	(M+H)+	C24 H24 O3	
362.1837	1	93654	2.40	362.1832	1.22	(M+H)+	C24 H24 O3	
549.2490	1	318078	8.17	549.2483	1.27	(M+H)+	C32 H36 O8	
550.2523	1	120126	3.08	550.2517	1.13	(M+H)+	C32 H36 O8	
688.3496	1	3894640	100.00	688.3480	2.36	(M+NH4)+	C40 H46 O9	
689.3529	1	1883714	48.37	689.3514	2.26	(M+NH4)+	C40 H46 O9	
690.3555	1	477033	12.25	690.3543	1.67	(M+NH4)+	C40 H46 O9	
691.3580	1	100060	2.57	691.3571	1.29	(M+NH4)+	C40 H46 O9	
693.3043	1	231316	5.94	693.3034	1.29	(M+Na)+	C40 H46 O9	
694.3076	1	109812	2.82	694.3068	1.20	(M+Na)+	C40 H46 O9	
688.4045	1	173595	4.46					
689.3879		94578	2.43					
689.4088	1	93903	2.41					
729.3763	1	3103444	79.68					
729.4332		134704	3.46					
730.3795	1	1597821	41.03					
731.3820	1	402623	10.34					

Spectrum Identification Table

Best	ID Source	Name	Formula	Species	m/z	Diff (ppm)	CAS	Score	Score (Lib)	Score (DB)	Score (MFG)	Lib/DB
No	MFG		C32 H36 O8	(M+H)+	549.2490	1.19		97.34			97.34	
No	MFG		C40 H46 O9	(M+Na)+	693.3043	1.28		96.47			96.47	
No	MFG		C40 H46 O9	(M+NH4)+	688.3496	2.33		95.41			95.41	
No	MFG		C17 H20 O6	(M+H)+	321.1337	1.50		93.27			93.27	
No	MFG		C24 H24 O3	(M+H)+	361.1803	1.37		81.68			81.68	
No	MFG		C8 H8 O	(M+H)+	121.0649	0.62		47.58			47.58	
No	MFG		C10 H10	(M+H)+	131.0857	1.17		47.47			47.47	

MassHunter Qual 10.0
(End of Report)

Compound ±7: Chiral HPLC
(Daicel Chiralpak AD-H, isocratic heptane/ethanol 85:5)

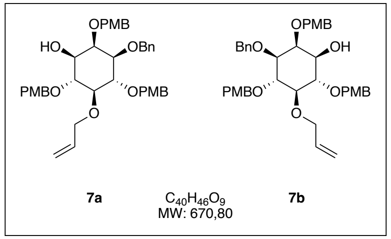

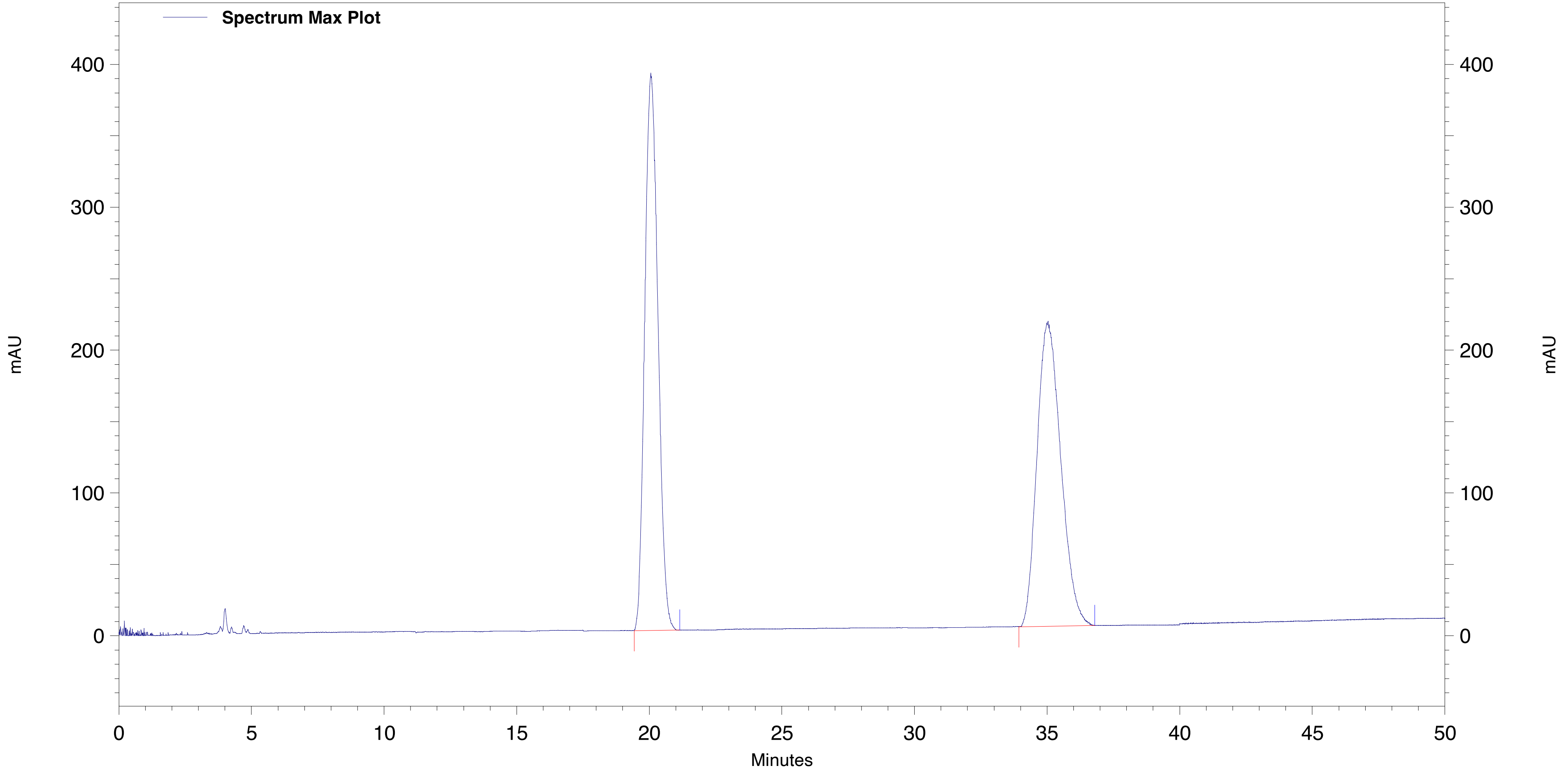

Compound 7a: Chiral HPLC
(Daicel Chiralpak AD-H, isocratic heptane/ethanol 85:5)

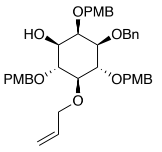

7a

$C_{40}H_{46}O_9$
MW: 670,80

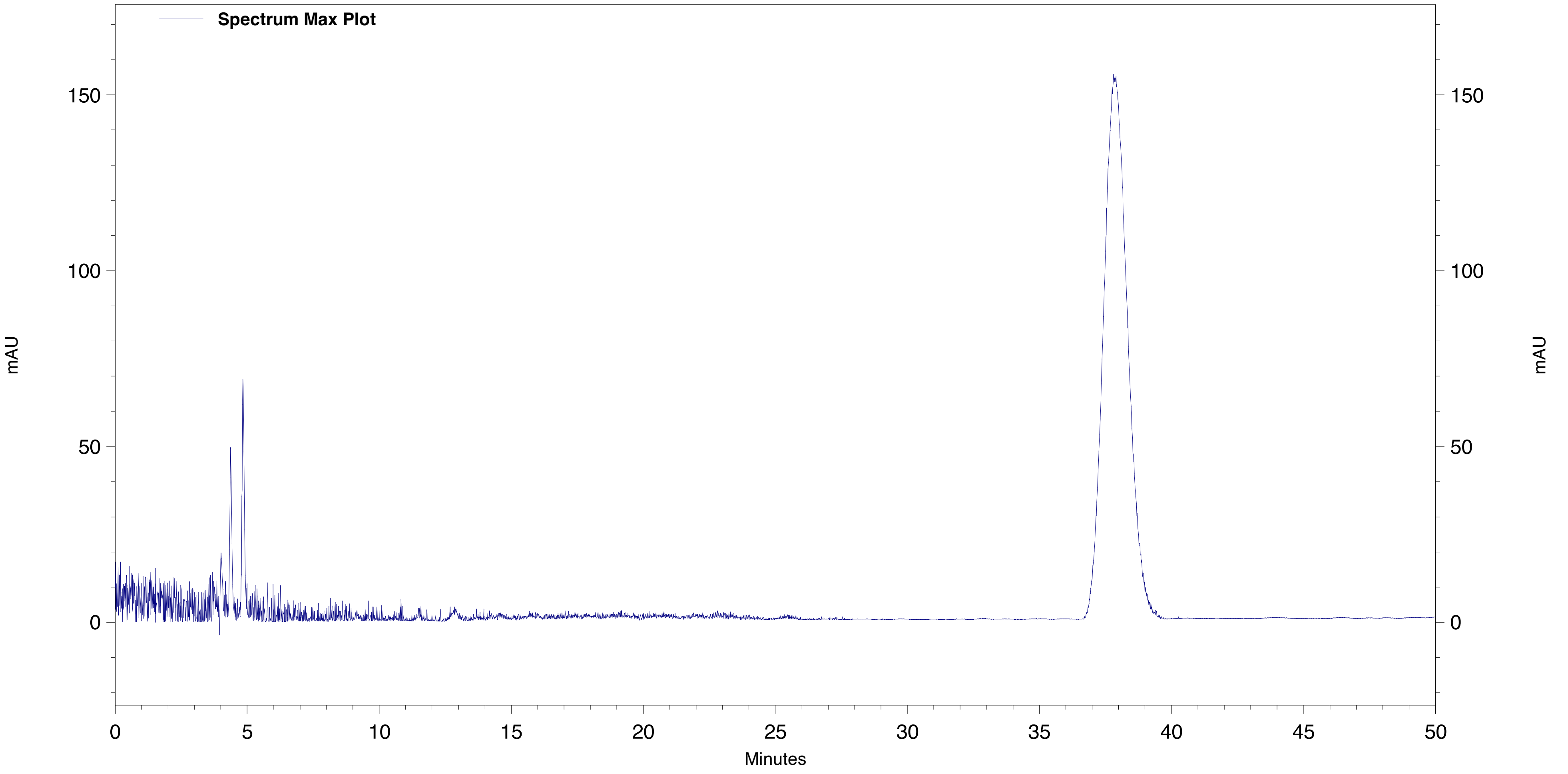

Compound 7b: Chiral HPLC
(Daicel Chiralpak AD-H, isocratic heptane/ethanol 85:5)

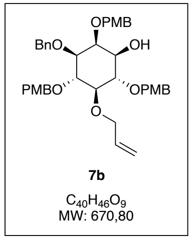

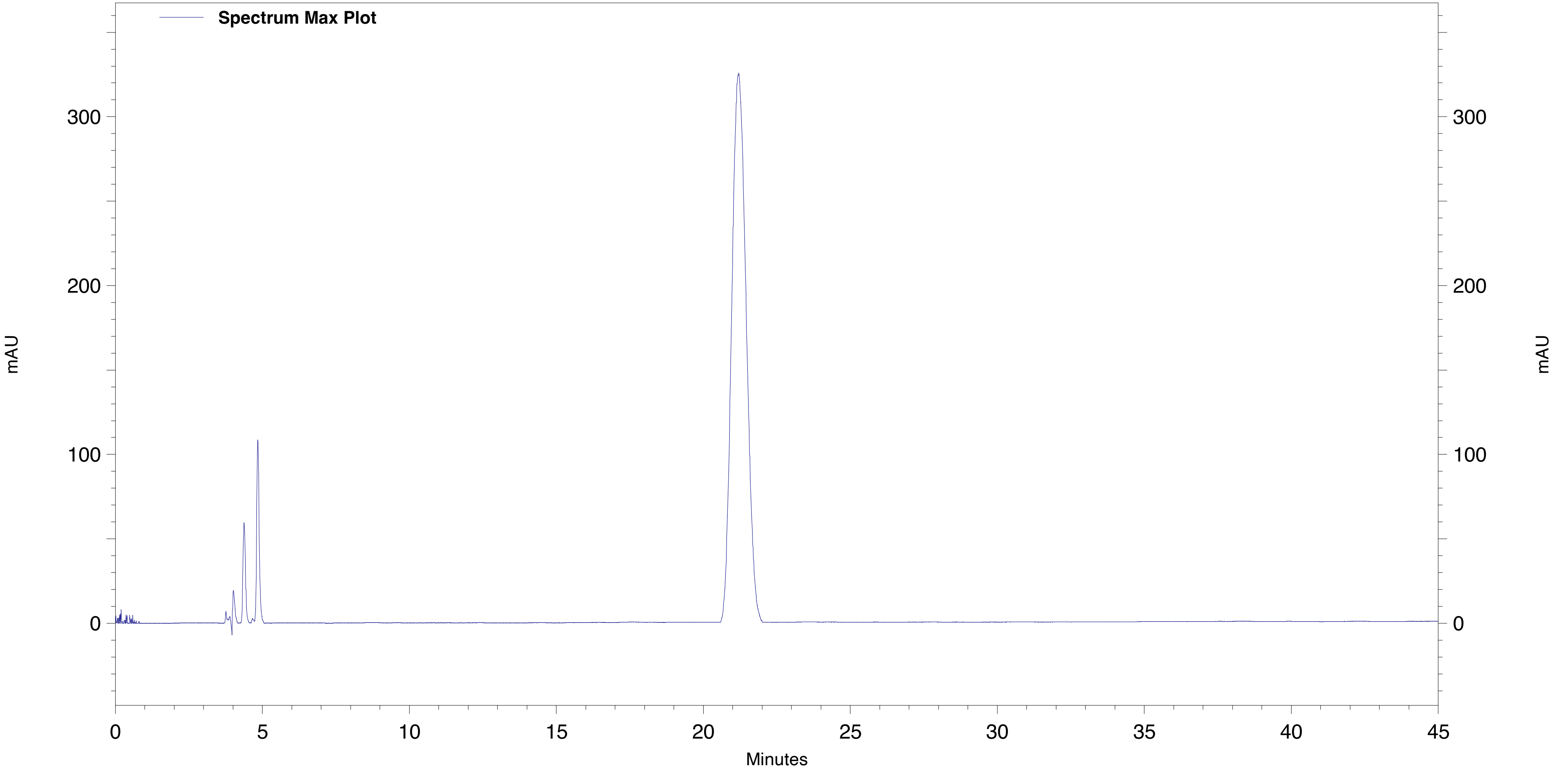

Compound ±9: HRMS (ESI) Analysis

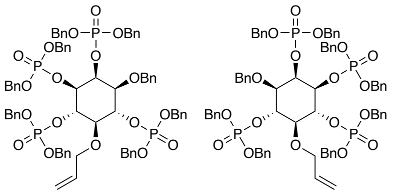

±9

$C_{72}H_{74}O_{18}P_4$
MW: 1351,26

Analysis Report

Agilent | Trusted Answers

Sample Information

**Name**	kr-a518	**Data File Path**	D:\MassHunter\Data\2024\12\rijea62dis02.d
**Sample ID**		**Acq. Time (Local)**	12/10/2024 9:18:41 AM (UTC+01:00)
**Instrument**	QTOF-2	**Method Path (Acq)**	D:\MassHunter\Methods\Christoph\direkt0,2mlACN.m
**MS Type**	QTOF	**Version (Acq SW)**	6200 series TOF/6500 series Q-TOF 10.1 (48.0)
**Inj. Vol. (ul)**	3	**IRM Status**	Success
**Position**		**Method Path (DA)**	
**Plate Pos.**		**Target Source Path**	
**Operator**		**Result Summary**	

Sample Chromatograms

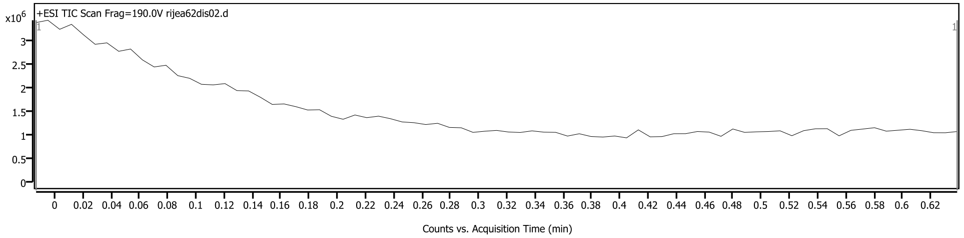

Sample Spectra

+ Scan (rt: -0.005-0.029 min) Sub

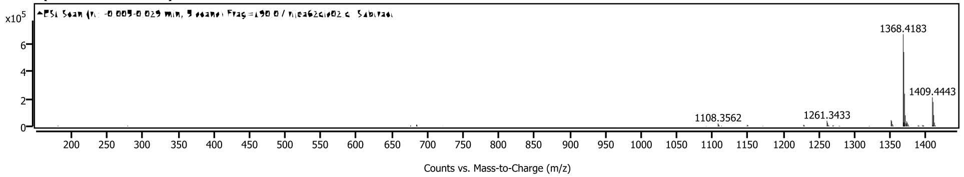

Spectrum Peaks

m/z	Z	Abund	Abund %	m/z (Calc)	Diff (ppm)	Ion Species	Formula	Ion Type
1108.3562	1	21262	3.18	1108.3562	0.02	(M+NH4)+	C58 H61 O15 P3	
1261.3433	1	41024	6.13	1261.3429	0.30	(M+NH4)+	C68 H63 O15 P4	
1262.3474	1	26482	3.96	1262.3463	0.85	(M+NH4)+	C68 H63 O15 P4	
1351.3912	1	45834	6.85	1351.3898	1.02	(M+Na)+	C70 H76 O18 P4	
1352.3936	1	38001	5.68	1352.3932	0.27	(M+Na)+	C70 H76 O18 P4	
1368.4183	1	668998	100.00	1368.4164	1.39	(M+NH4)+	C72 H74 O18 P4	
1369.4216	1	540072	80.73	1369.4198	1.36	(M+NH4)+	C72 H74 O18 P4	
1370.4242	1	237096	35.44	1370.4229	0.98	(M+NH4)+	C72 H74 O18 P4	
1371.4282	1	79951	11.95	1371.4258	1.71	(M+NH4)+	C72 H74 O18 P4	
1372.4293	1	21624	3.23	1372.4287	0.40	(M+NH4)+	C72 H74 O18 P4	
1373.3723	1	36223	5.41	1373.3718	0.37	(M+Na)+	C72 H74 O18 P4	
1374.3758	1	26625	3.98	1374.3752	0.44	(M+Na)+	C72 H74 O18 P4	
1368.3793	1	27047	4.04					
1368.4949	1	32554	4.87					
1369.3781	1	25977	3.88					
1369.4983	1	28441	4.25					
1409.4443	1	211558	31.62					
1410.4473	1	177861	26.59					
1411.4512	1	81937	12.25					
1412.4522	1	27476	4.11					

Compound ±11: HRMS (ESI) Analysis

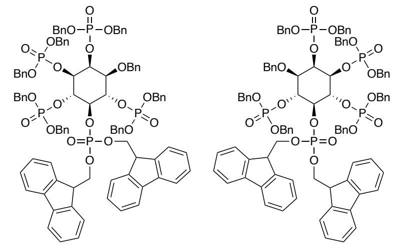

±11

$C_{97}H_{91}O_{21}P_5$
MW: 1747,64

Analysis Report

Sample Information

**Name**	kr-a523	**Data File Path**	D:\MassHunter\Data\2024\12\rijea63dis01.d
**Sample ID**		**Acq. Time (Local)**	12/10/2024 9:26:05 AM (UTC+01:00)
**Instrument**	QTOF-2	**Method Path (Acq)**	D:\MassHunter\Methods\Christoph\direkt0,2mlACN.m
**MS Type**	QTOF	**Version (Acq SW)**	6200 series TOF/6500 series Q-TOF 10.1 (48.0)
**Inj. Vol. (ul)**	3	**IRM Status**	Success
**Position**		**Method Path (DA)**	
**Plate Pos.**		**Target Source Path**	
**Operator**		**Result Summary**	

Sample Chromatograms

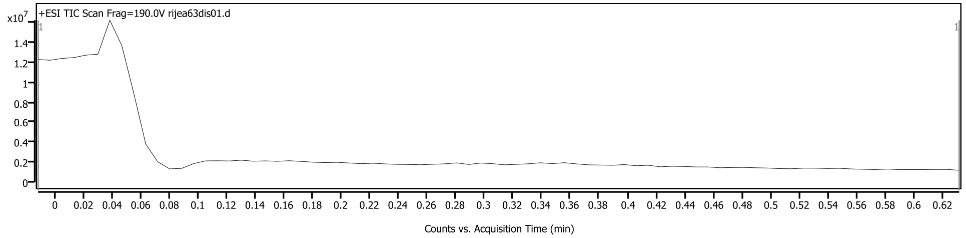

Sample Spectra

+ Scan (rt: 0.005-0.080 min) Sub

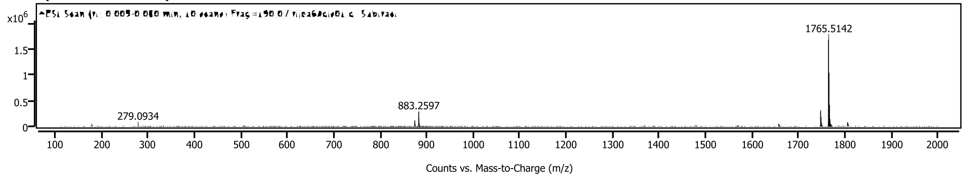

Spectrum Peaks

m/z	Z	Abund	Abund %	m/z (Calc)	Diff (ppm)	Ion Species	Formula	Ion Type
874.2445	2	111751	6.23	874.2443	0.18	(M+2H)+2	C97 H91 O21 P5	
874.7464	2	118240	6.59	874.7460	0.41	(M+2H)+2	C97 H91 O21 P5	
875.2478	2	72538	4.04	875.2476	0.24	(M+2H)+2	C97 H91 O21 P5	
1747.4832	1	291709	16.26	1747.4814	1.07	(M+Na)+	C95 H93 O21 P5	
1748.4865	1	320926	17.89	1748.4848	0.97	(M+Na)+	C95 H93 O21 P5	
1749.4893	1	183916	10.25	1749.4880	0.76	(M+Na)+	C95 H93 O21 P5	
1750.4916	1	77309	4.31	1750.4911	0.32	(M+Na)+	C95 H93 O21 P5	
1764.5107	1	1681421	93.74	1764.5079	1.60	(M+NH4)+	C97 H91 O21 P5	
1765.5142	1	1793661	100.00	1765.5113	1.62	(M+NH4)+	C97 H91 O21 P5	
1766.5172	1	1044140	58.21	1766.5145	1.55	(M+NH4)+	C97 H91 O21 P5	
1767.5195	1	417790	23.29	1767.5176	1.08	(M+NH4)+	C97 H91 O21 P5	
1768.5216	1	139490	7.78	1768.5205	0.62	(M+NH4)+	C97 H91 O21 P5	
279.0934	1	89337	4.98	279.0933	0.30			
882.7579	2	280124	15.62	882.7589	-1.15			
883.2597	2	293536	16.37	883.2606	-1.04			
883.7612	2	167726	9.35	883.7622	-1.12			
1764.5822	1	82137	4.58	1764.5807	0.85			
1765.5862	1	103186	5.75	1765.5841	1.19			
1805.5344	1	78127	4.36					
1806.5386	1	82892	4.62					

Spectrum Identification Table

Best	ID Source	Name	Formula	Species	m/z	Diff (ppm)	CAS	Score	Score (Lib)	Score (DB)	Score (MFG)	Lib/DB
No	MFG		C97 H91 O21 P5	(M+2H)+2	874.2445	0.24		98.56			98.56	
No	MFG		C97 H91 O21 P5	(M+H)+	1747.4832	0.88		98.43			98.43	
No	MFG		C97 H91 O21 P5	(M+NH4)+	1764.5107	1.53		97.58			97.58	
No	MFG		C95 H93 O21 P5	(M+Na)+	1747.4832	2.28		93.09			93.09	

MassHunter Qual 10.0
(End of Report)

Compound ±12: HRMS (ESI) Analysis

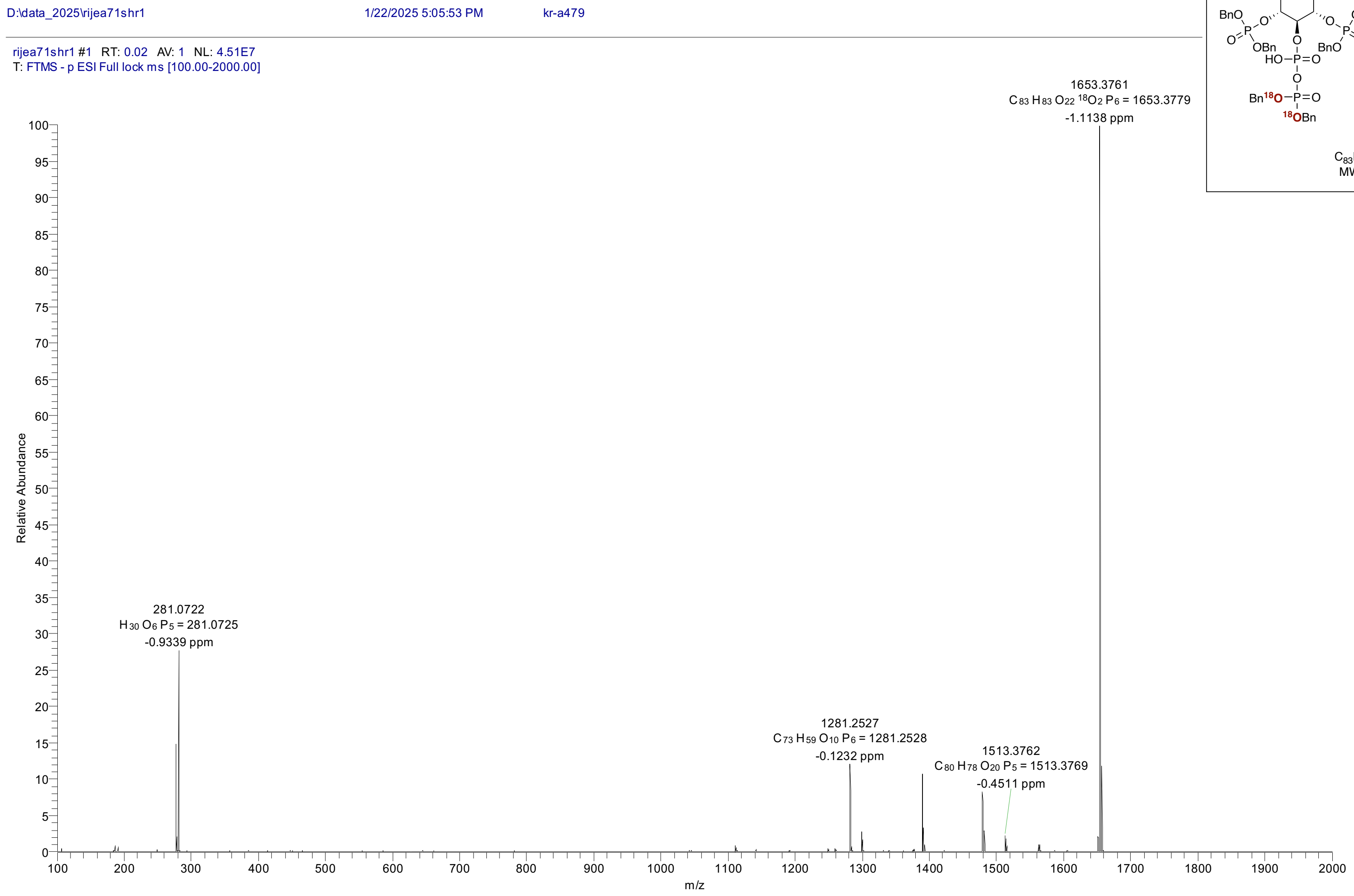

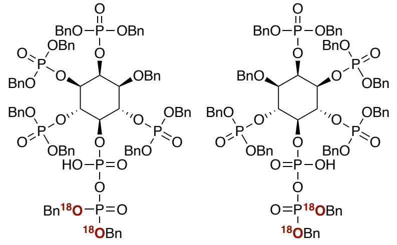

±12

$C_{83}H_{84}O_{22}{}^{18}O_2P_6$
MW: 1655,4039

Compound 12a: HRMS (ESI) Analysis

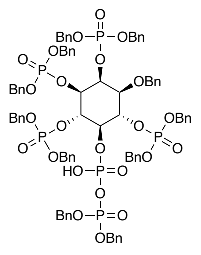

12a

$C_{83}H_{84}O_{24}P_6$
MW: 1651,40

Analysis Report

Agilent | Trusted Answers

Sample Information

**Name**	kr-a528	**Data File Path**	D:\MassHunter\Data\2024\12\rijea61dis01.d
**Sample ID**		**Acq. Time (Local)**	12/10/2024 9:05:28 AM (UTC+01:00)
**Instrument**	QTOF-2	**Method Path (Acq)**	D:\MassHunter\Methods\Christoph\direkt0,2mlACN.m
**MS Type**	QTOF	**Version (Acq SW)**	6200 series TOF/6500 series Q-TOF 10.1 (48.0)
**Inj. Vol. (ul)**	3	**IRM Status**	Some ions missed
**Position**		**Method Path (DA)**	
**Plate Pos.**		**Target Source Path**	
**Operator**		**Result Summary**	

Sample Chromatograms

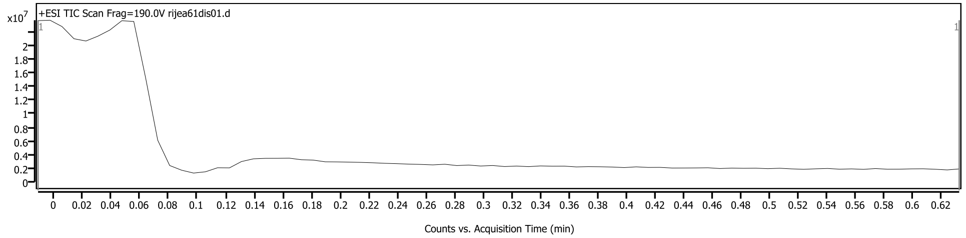

Sample Spectra

+ Scan (rt: 0.056-0.081 min) Sub

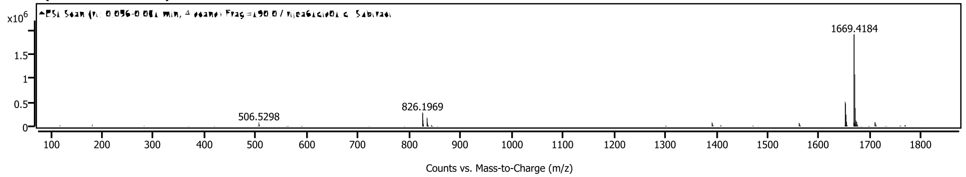

Spectrum Peaks

m/z	Z	Abund	Abund %	m/z (Calc)	Diff (ppm)	Ion Species	Formula	Ion Type
826.1969	2	290598	15.22	826.1962	0.87	(M+2(NH4))+2	C85 H73 O23 P5	
826.6987	2	268829	14.08	826.6979	0.93	(M+2(NH4))+2	C85 H73 O23 P5	
827.2001	2	132832	6.96	827.1994	0.81	(M+2(NH4))+2	C85 H73 O23 P5	
1651.3878	1	512704	26.85	1651.3851	1.63	(M+H)+	C83 H84 O24 P6	
1652.3912	1	478430	25.05	1652.3885	1.65	(M+H)+	C83 H84 O24 P6	
1653.3937	1	240983	12.62	1653.3916	1.23	(M+H)+	C83 H84 O24 P6	
1654.3964	1	93896	4.92	1654.3946	1.08	(M+H)+	C83 H84 O24 P6	
1668.4147	1	1905138	99.77	1668.4116	1.83	(M+NH4)+	C83 H84 O24 P6	
1669.4184	1	1909547	100.00	1669.4150	2.00	(M+NH4)+	C83 H84 O24 P6	
1670.4213	1	1077926	56.45	1670.4181	1.91	(M+NH4)+	C83 H84 O24 P6	
1671.4235	1	381442	19.98	1671.4211	1.45	(M+NH4)+	C83 H84 O24 P6	
1672.4257	1	123211	6.45	1672.4240	1.00	(M+NH4)+	C83 H84 O24 P6	
1673.3686	1	102472	5.37	1673.3694	-0.51	(M+Na)+	C83 H84 O24 P6	
1674.3717	1	92201	4.83	1674.3728	-0.67	(M+Na)+	C83 H84 O24 P6	
506.5298		84539	4.43					
834.7099	2	178872	9.37	834.7088	1.30			
835.2119	2	168879	8.84	835.2104	1.74			
835.7133	2	85093	4.46	835.7120	1.59			
1668.4843		85538	4.48					
1709.4390	1	88011	4.61	1709.4388	0.06			

Spectrum Identification Table

Best	ID Source	Name	Formula	Species	m/z	Diff (ppm)	CAS	Score	Score (Lib)	Score (DB)	Score (MFG)	Lib/DB
No	MFG		C83 H84 O24 P6	(M+2H)+2	826.1969	0.83		98.85			98.85	
No	MFG		C85 H82 O24 P6	(M+H)+	1673.3686	-0.65		97.56			97.56	
No	MFG		C85 H83 O24 P6	M+	1673.3686	-0.65		97.56			97.56	
No	MFG		C83 H84 O24 P6	(M+Na)+	1673.3686	0.80		97.46			97.46	
No	MFG		C83 H84 O24 P6	(M+H)+	1651.3878	1.51		97.44			97.44	
No	MFG		C83 H85 O24 P6	M+	1651.3878	1.50		97.44			97.44	
No	MFG		C85 H73 O23 P5	(M+2(NH4))+2	826.1969	1.89		96.79			96.79	
No	MFG		C83 H84 O24 P6	(M+NH4)+	1668.4147	1.87		94.40			94.40	

Compound 13a: CE-qTOF electropherogram

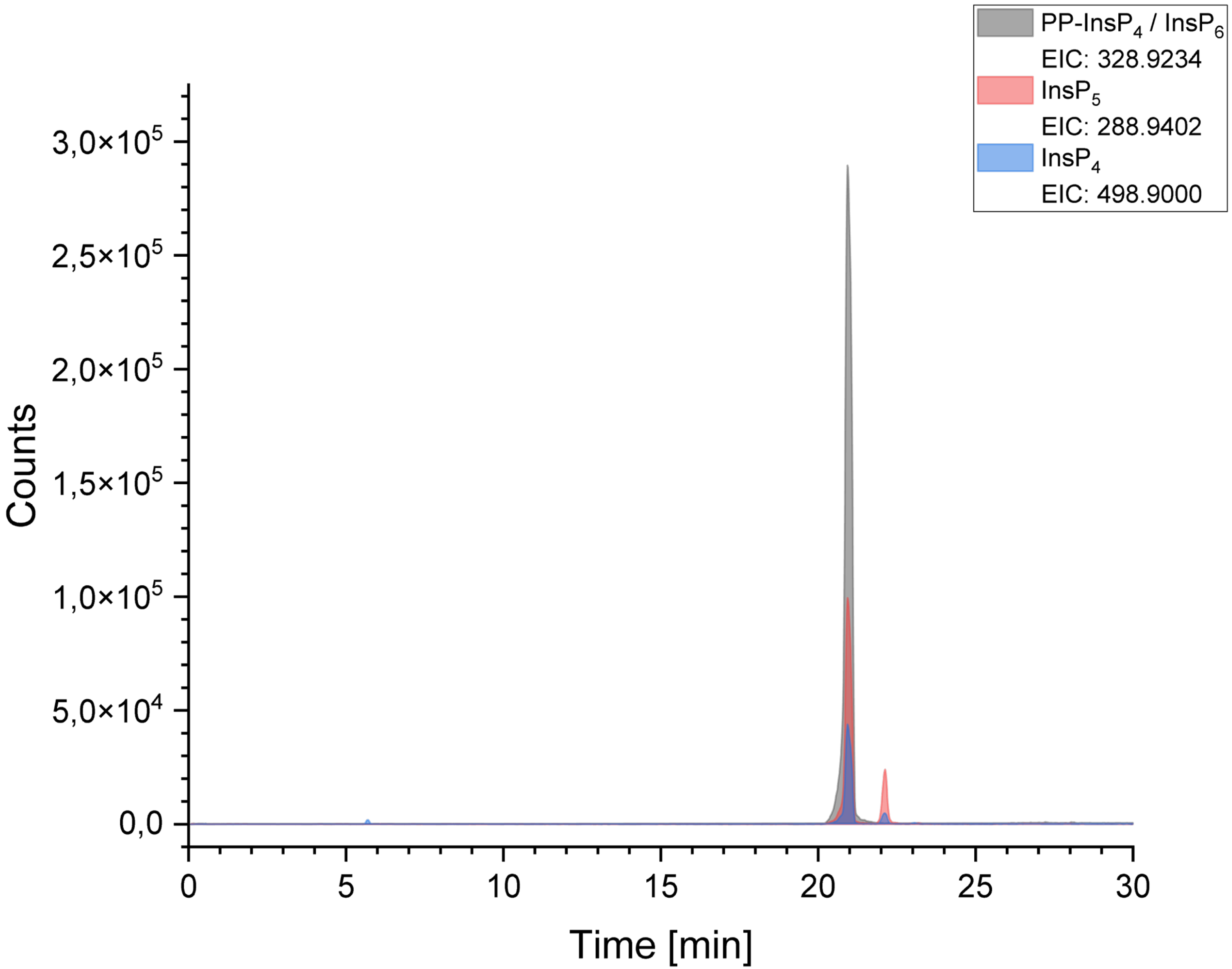

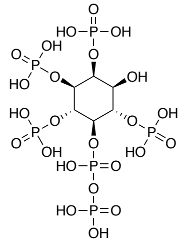

13a

$C_6H_{18}O_{24}P_6$
MW: 660,03

Compound 13a: HRMS (CE-qTOF) Analysis

**Sample Name**	KR-A547	**Position**	13	**Instrument Name**	QTOF-1
**User Name**		**Inj Vol**	Unknown / Injection Program	**InjPosition**	
**Sample Type**	Sample	**IRM Calibration Status**	Success	**Data Filename**	KR-A547.d
**ACQ Method**	Standard method.m	**Comment**		**Acquired Time**	13.12.2024 14:52:52 (UTC+01:00)

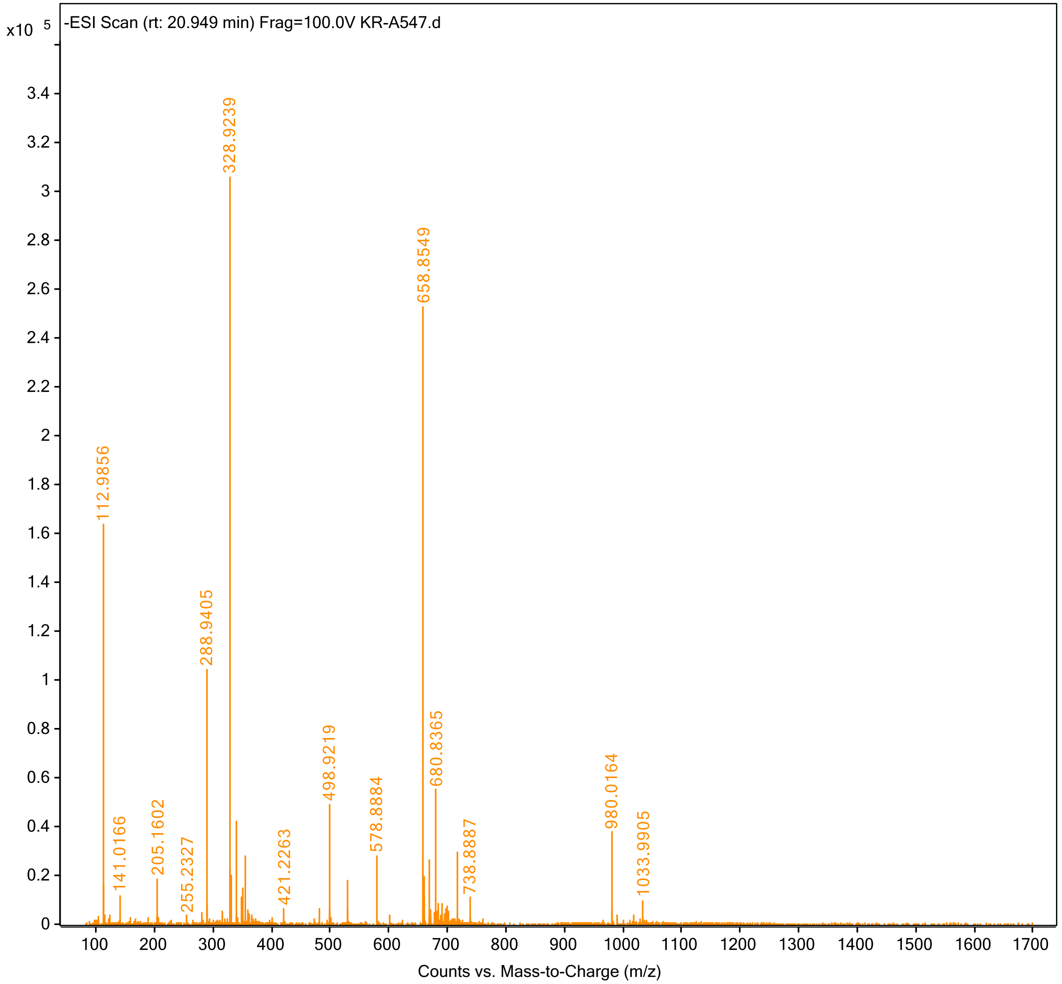

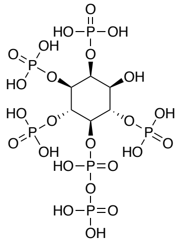

13a

$C_6H_{18}O_{24}P_6$
MW: 660,03

Compound 13b: CE-qTOF electropherogram

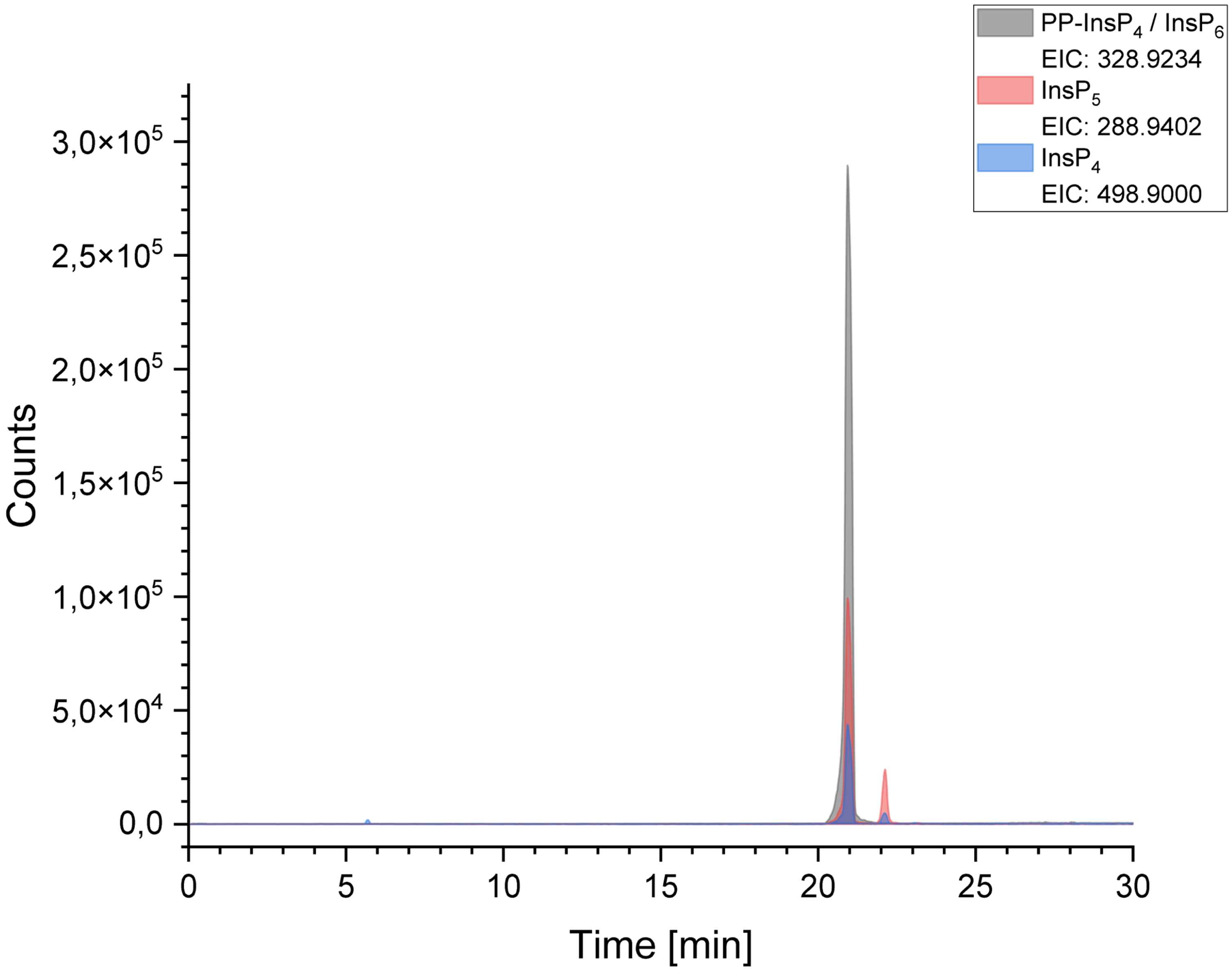

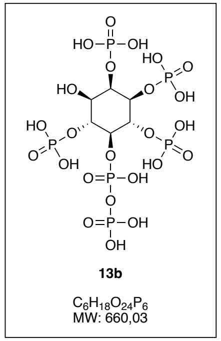

Compound 13b: HRMS (CE-qTOF) Analysis

**Sample Name**	KR-A545	**Position**	12	**Instrument Name**	QTOF-1
**User Name**		**Inj Vol**	Unknown / Injection Program	**InjPosition**	
**Sample Type**	Sample	**IRM Calibration Status**	Success	**Data Filename**	KR-A545.d
**ACQ Method**	Standard method.m	**Comment**		**Acquired Time**	13.12.2024 14:13:28 (UTC+01:00)

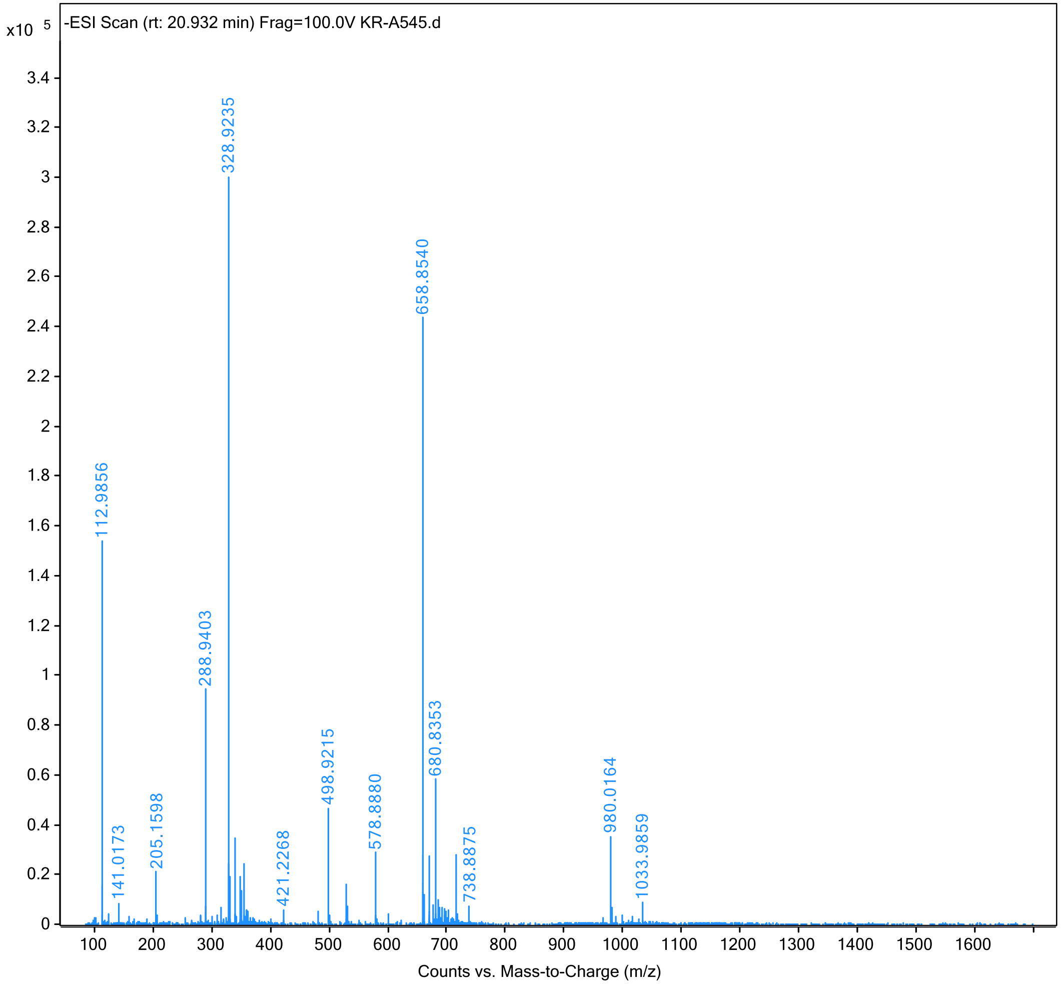

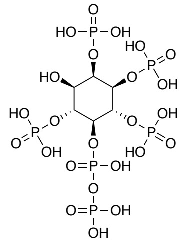

13b

$C_6H_{18}O_{24}P_6$
MW: 660,03

Compound 14a: CE-qTOF electropherogram

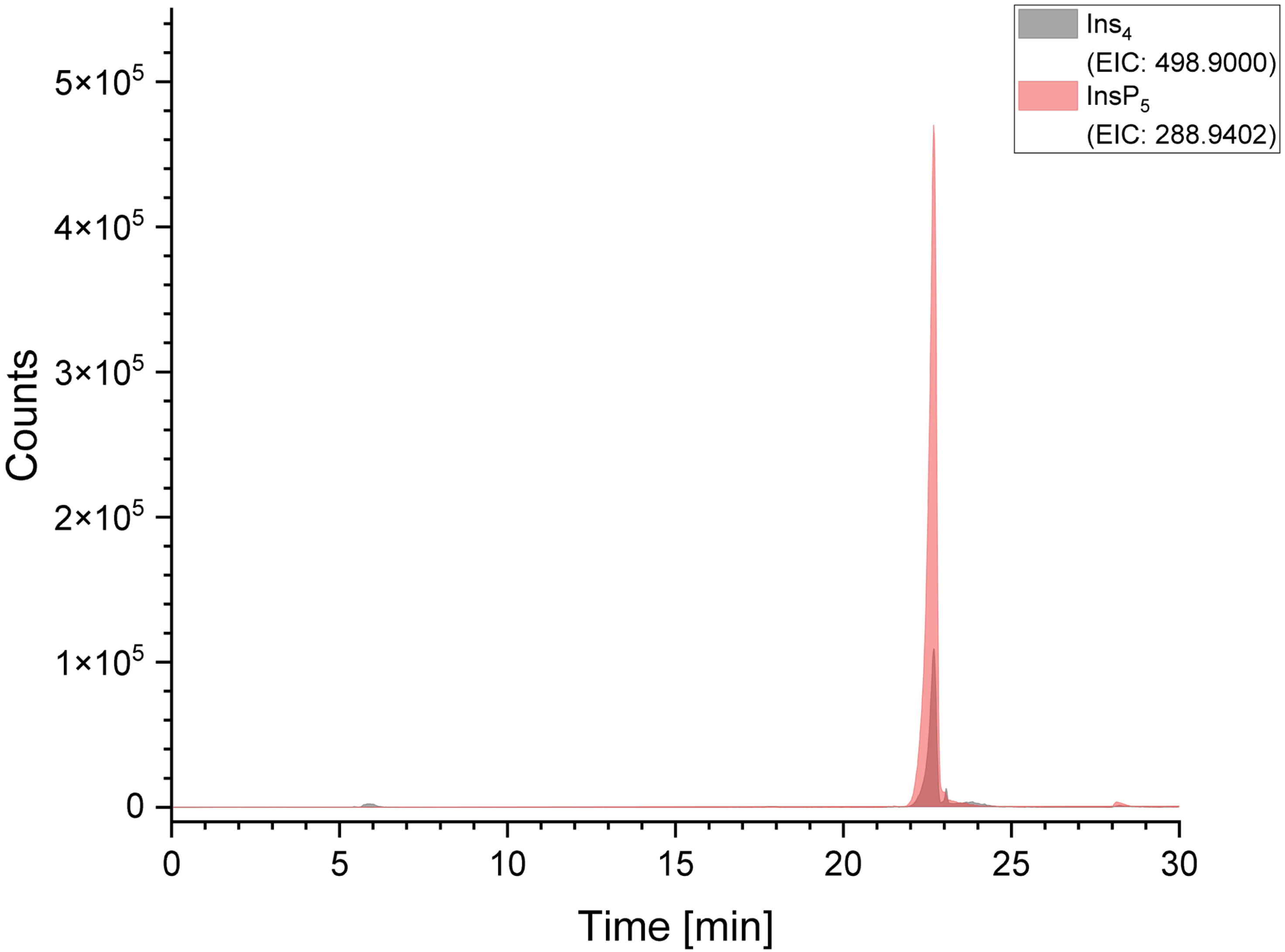

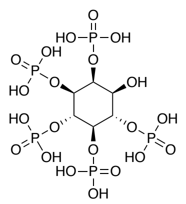

14a

$C_6H_{17}O_{21}P_5$
MW: 580,05

Compound 14a: HRMS (CE-qTOF) Analysis

**Sample Name**	KR-A534	**Position**	06	**Instrument Name**	QTOF-1
**User Name**		**Inj Vol**	Unknown / Injection Program	**InjPosition**	
**Sample Type**	Sample	**IRM Calibration Status**	Success	**Data Filename**	KR-A534.d
**ACQ Method**	Standard method.m	**Comment**		**Acquired Time**	13.12.2024 10:17:01 (UTC+01:00)

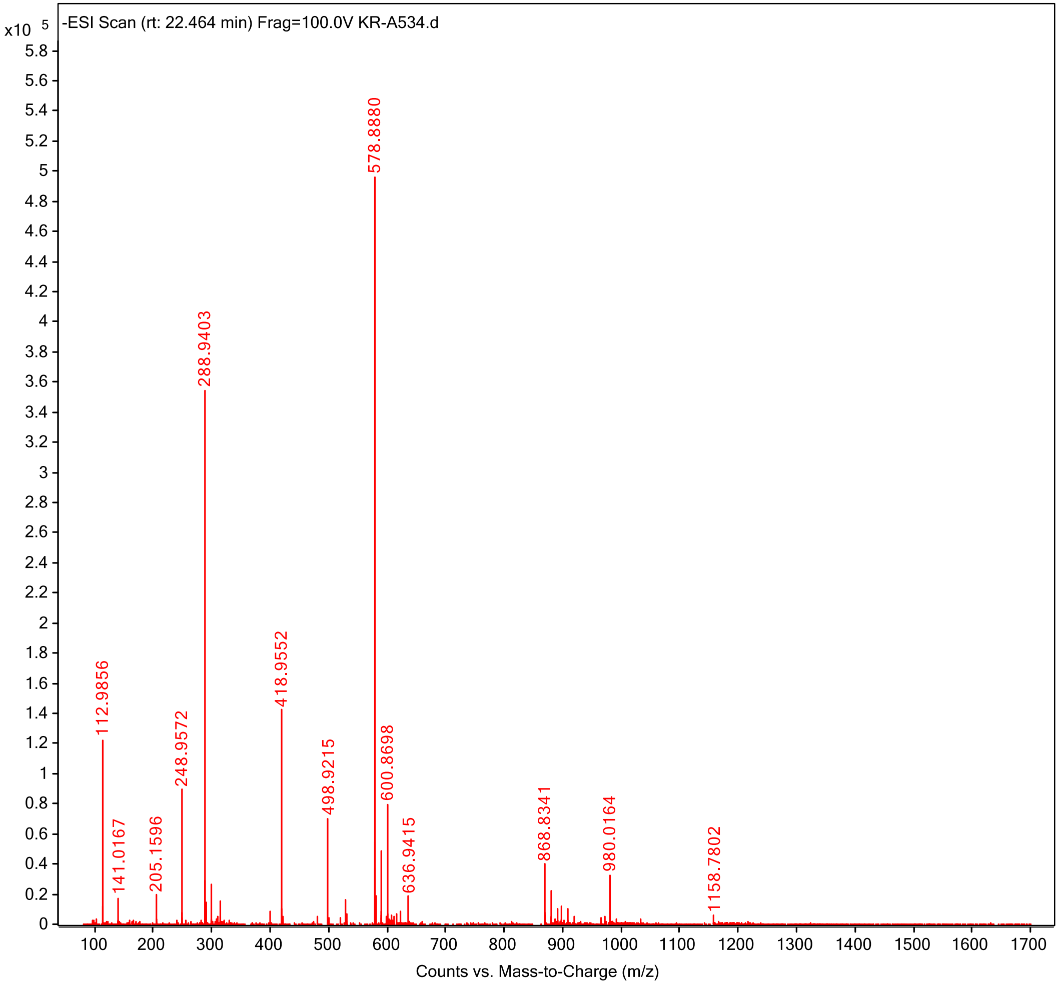

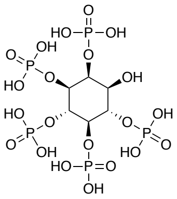

14a

$C_6H_{17}O_{21}P_5$
MW: 580,05

Compound 14b: CE-qTOF electropherogram

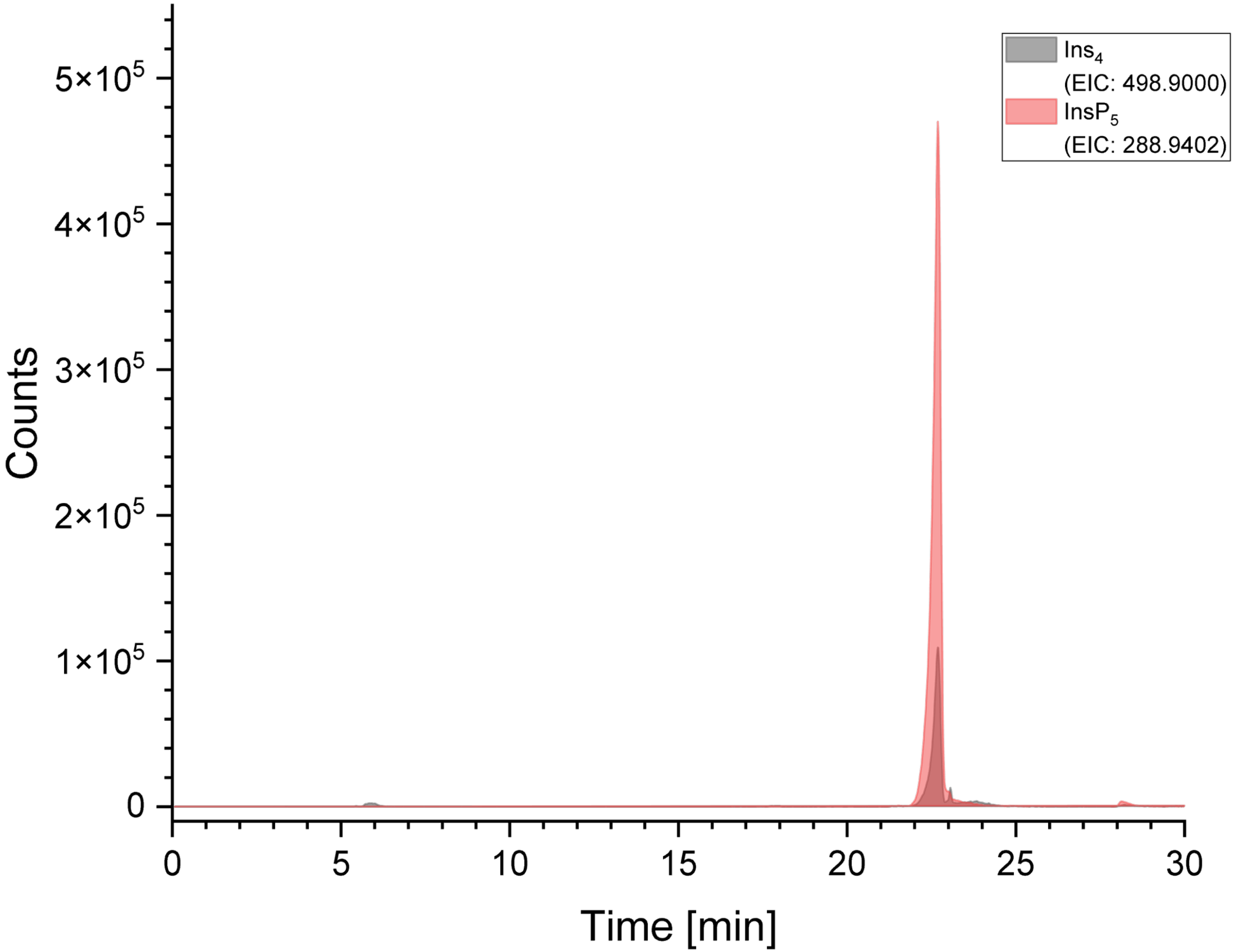

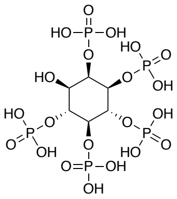

14b

$C_6H_{17}O_{21}P_5$
MW: 580,05

Compound 14b: HRMS (CE-qTOF) Analysis

**Sample Name**	KR-A533	**Position**	05	**Instrument Name**	QTOF-1
**User Name**		**Inj Vol**	Unknown / Injection Program	**InjPosition**	
**Sample Type**	Sample	**IRM Calibration Status**	Success	**Data Filename**	KR-A533.d
**ACQ Method**	Standard method.m	**Comment**		**Acquired Time**	13.12.2024 09:37:33 (UTC+01:00)

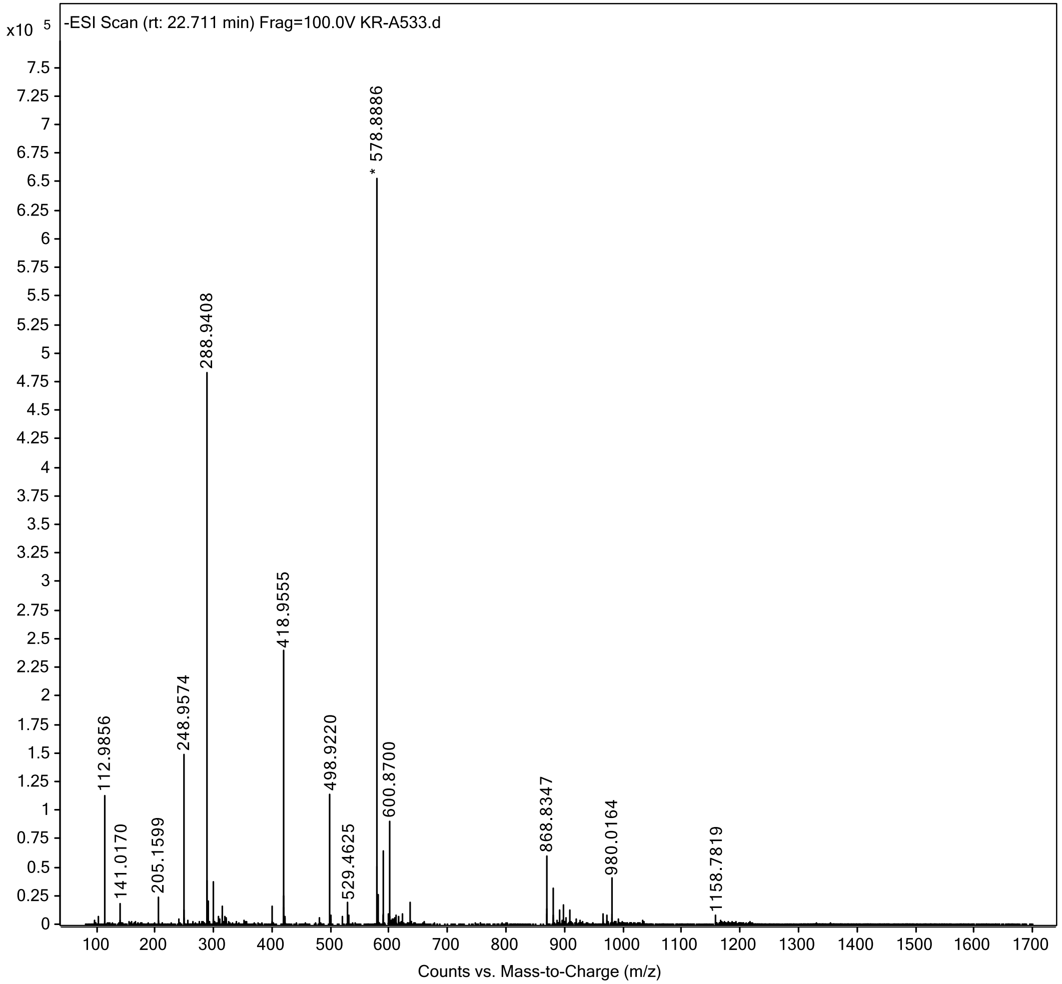

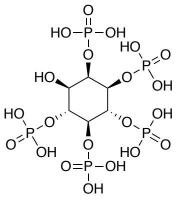

14b

$C_6H_{17}O_{21}P_5$
MW: 580,05

Compound ±16: HRMS (ESI) Analysis

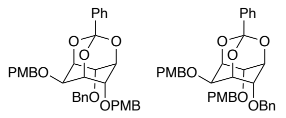

±16

$C_{36}H_{36}O_8$
MW: 596,6760

Analysis Report

Agilent | Trusted Answers

Sample Information

Name	kr-a499	Data File Path	D:\MassHunter\Data\2024\12\rijea64dis01.d
Sample ID		Acq. Time (Local)	12/10/2024 1:55:15 PM (UTC+01:00)
Instrument	QTOF-2	Method Path (Acq)	D:\MassHunter\Methods\Christoph\direkt0,2mlACN.m
MS Type	QTOF	Version (Acq SW)	6200 series TOF/6500 series Q-TOF 10.1 (48.0)
Inj. Vol. (ul)	3	IRM Status	Some ions missed
Position		Method Path (DA)	
Plate Pos.		Target Source Path	
Operator		Result Summary	

Sample Chromatograms

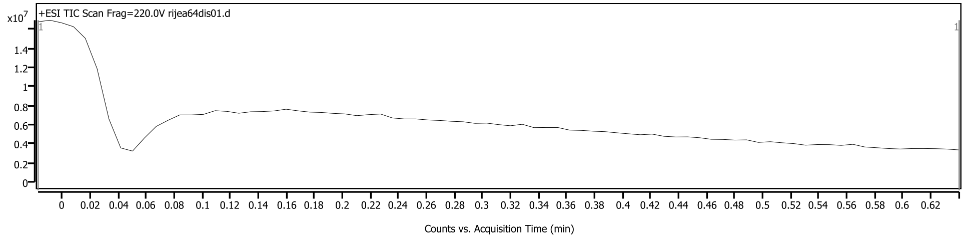

Sample Spectra

+ Scan (rt: 0.017-0.042 min) Sub

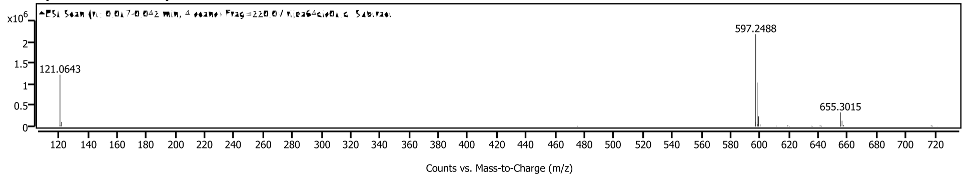

Spectrum Peaks

m/z	Z	Abund	Abund %	m/z (Calc)	Diff (ppm)	Ion Species	Formula	Ion Type
597.2488	1	2182729	100.00	597.2483	0.84	(M+NH4)+	C34 H31 N2 O7	
598.2519	1	1038277	47.57	598.2517	0.36	(M+NH4)+	C34 H31 N2 O7	
599.2545	1	237679	10.89	599.2546	-0.20	(M+NH4)+	C34 H31 N2 O7	
600.2571	1	48304	2.21	600.2574	-0.59	(M+NH4)+	C34 H31 N2 O7	
121.0643	1	1223442	56.05					
122.0675	1	105596	4.84					
597.3006	1	106413	4.88					
598.2848		51361	2.35					
598.3046	1	55988	2.57					
655.3015	1	331550	15.19					
656.3047	1	135410	6.20					

Spectrum Identification Table

Best	ID Source	Name	Formula	Species	m/z	Diff (ppm)	CAS	Score	Score (Lib)	Score (DB)	Score (MFG)	Lib/DB
No	MFG		C36 H36 O8	(M+H)+	597.2488	0.61		94.37			94.37	
No	MFG		C34 H31 N2 O7	(M+NH4)+	597.2488	3.08		88.30			88.30	

MassHunter Qual 10.0
(End of Report)

Compound ±17: HRMS (ESI) Analysis

D:\data_2024\rijea65shr2 12/17/2024 3:40:48 PM kr-a500

rijea65shr2 #1 RT: 0.02 AV: 1 NL: 1.06E6
T: FTMS + p ESI Full lock ms [70.00-1000.00]

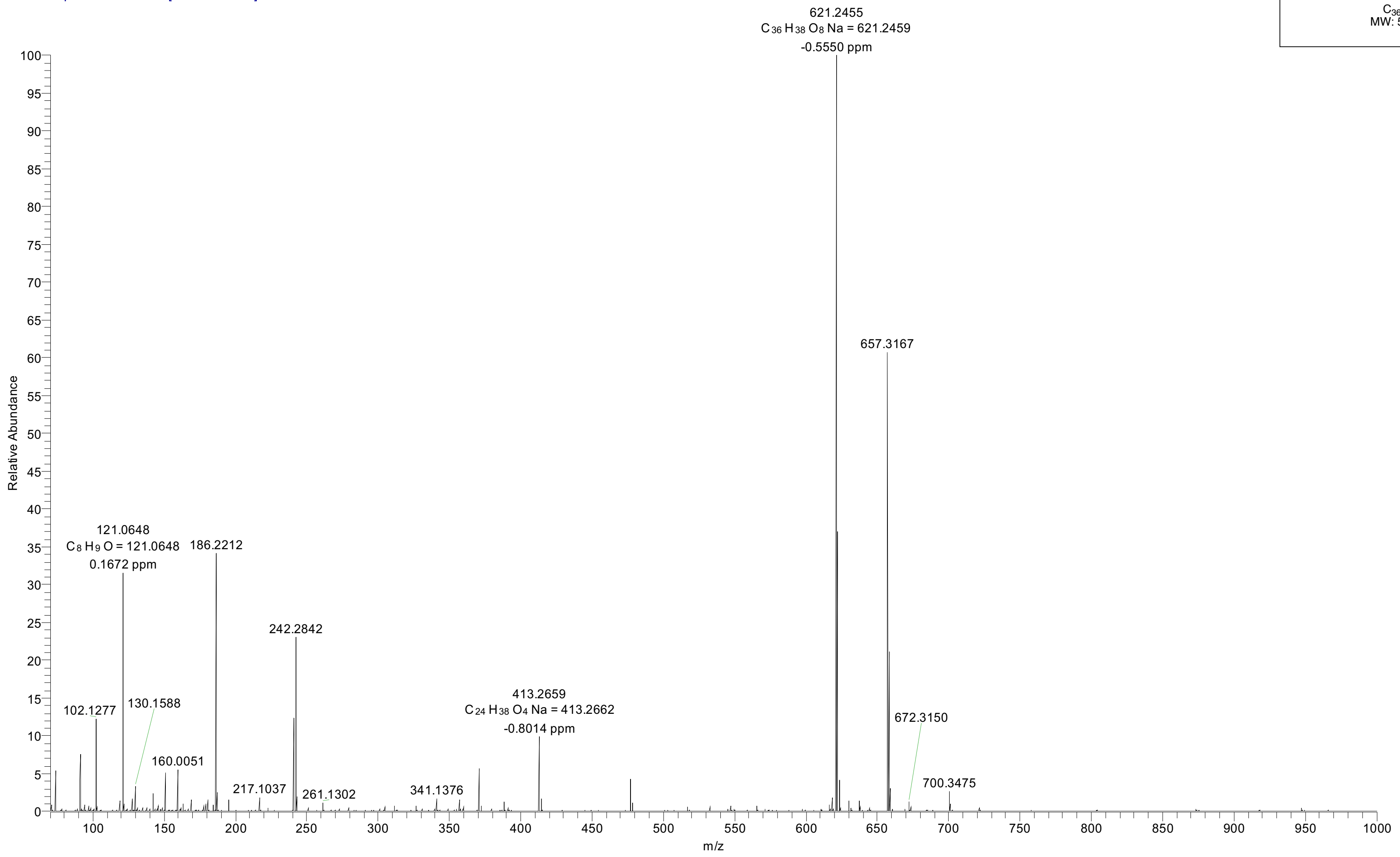

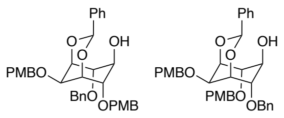

±17

$C_{36}H_{38}O_8$
MW: 598,6920

Compound ±19: HRMS (ESI) Analysis

D:\data_2025\rijea72shr2 1/22/2025 5:12:16 PM kr-a487

rijea72shr2 #1 RT: 0.02 AV: 1 NL: 5.47E7
T: FTMS + p ESI Full lock ms [100.00-1000.00]

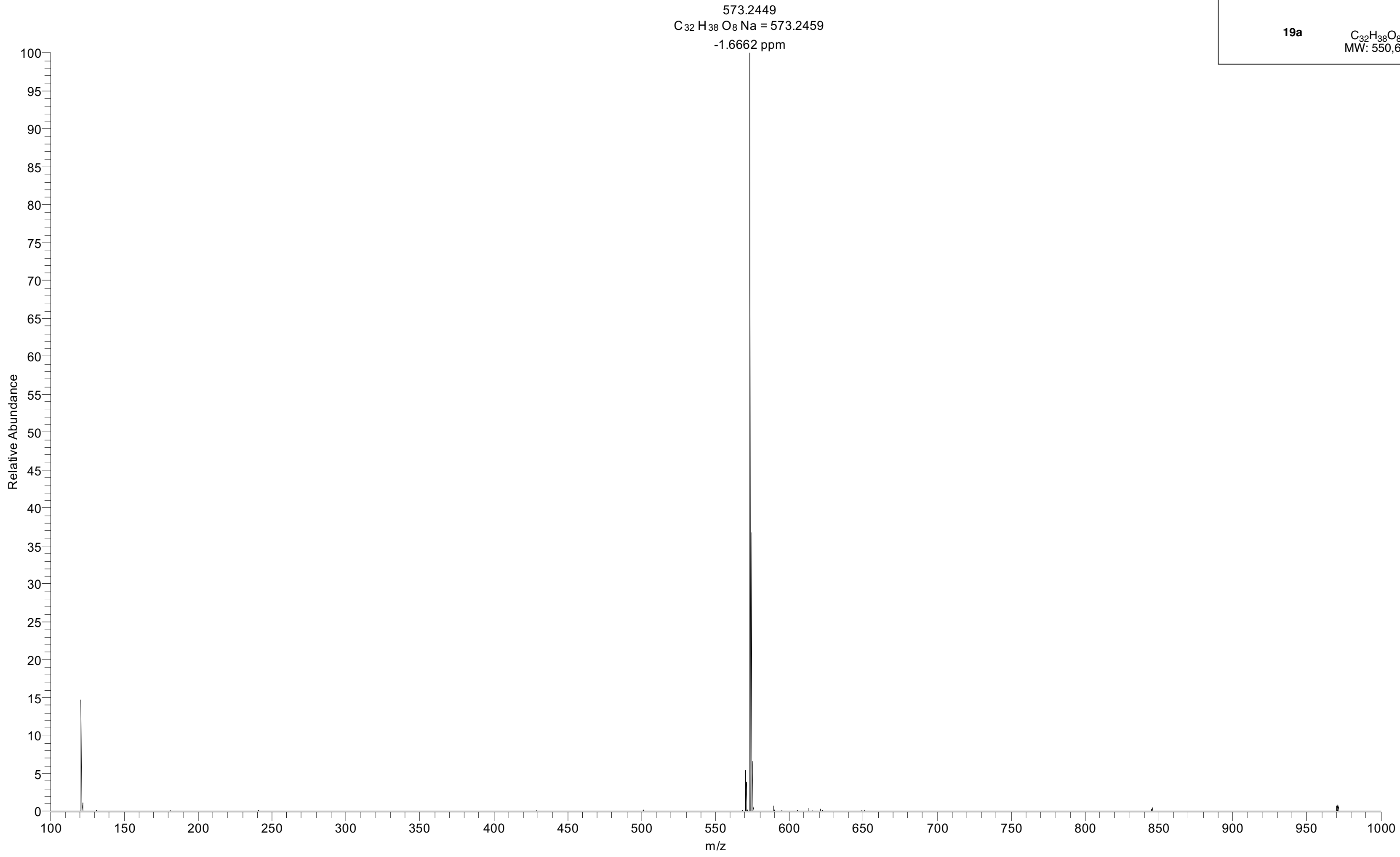

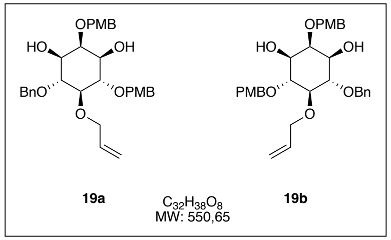

Compound ±19: Chiral HPLC
(Daicel Chiralpak AD-H, isocratic heptane/ethanol 85:5)

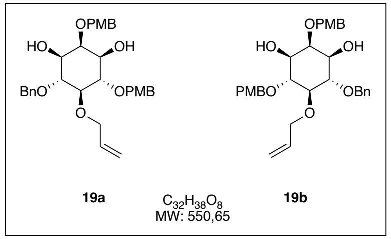

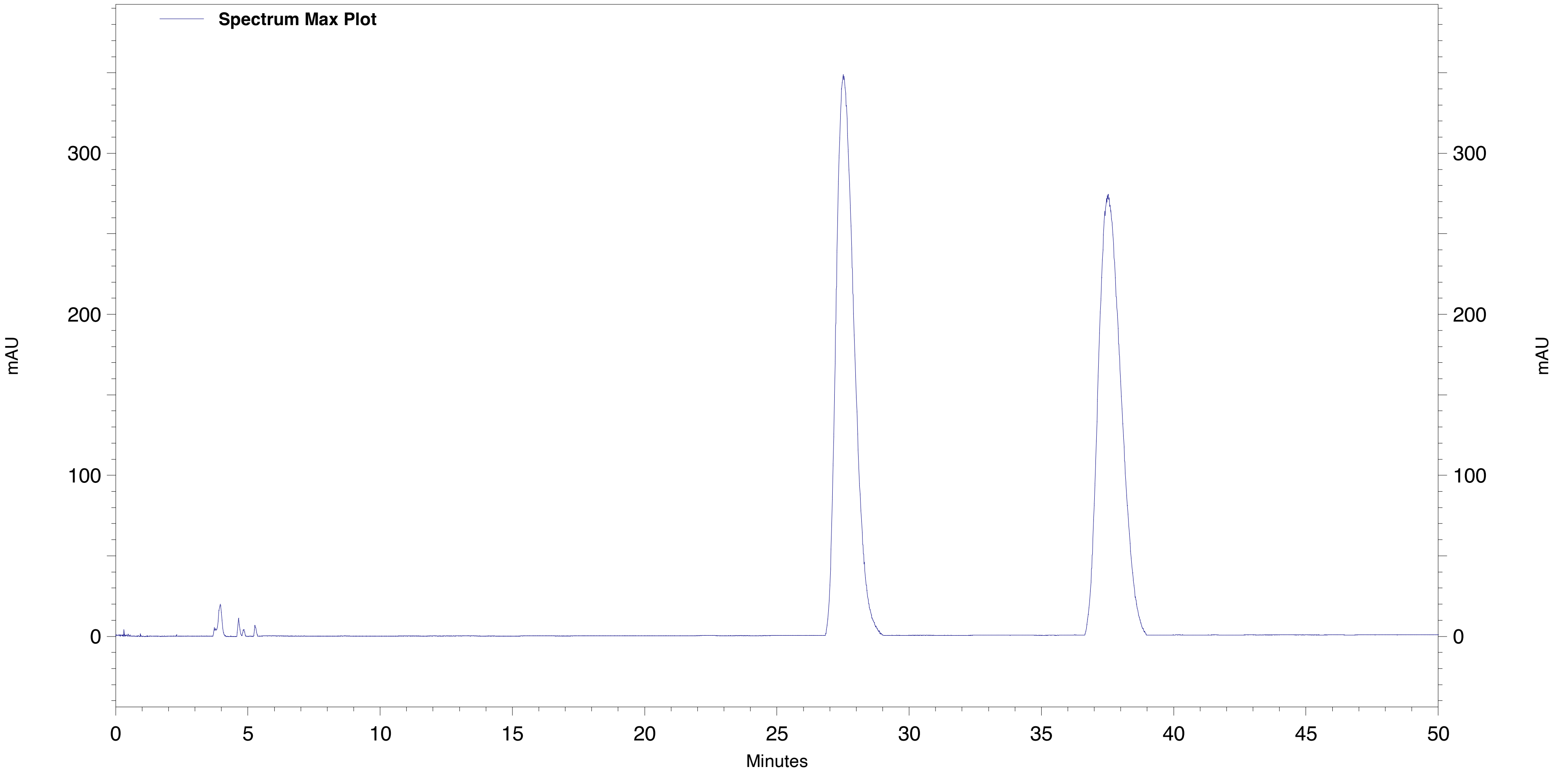

Compound 19a: Chiral HPLC
(Daicel Chiralpak AD-H, isocratic heptane/ethanol 85:5)

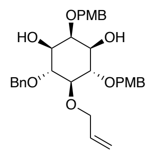

19a

$C_{32}H_{38}O_8$
MW: 550,65

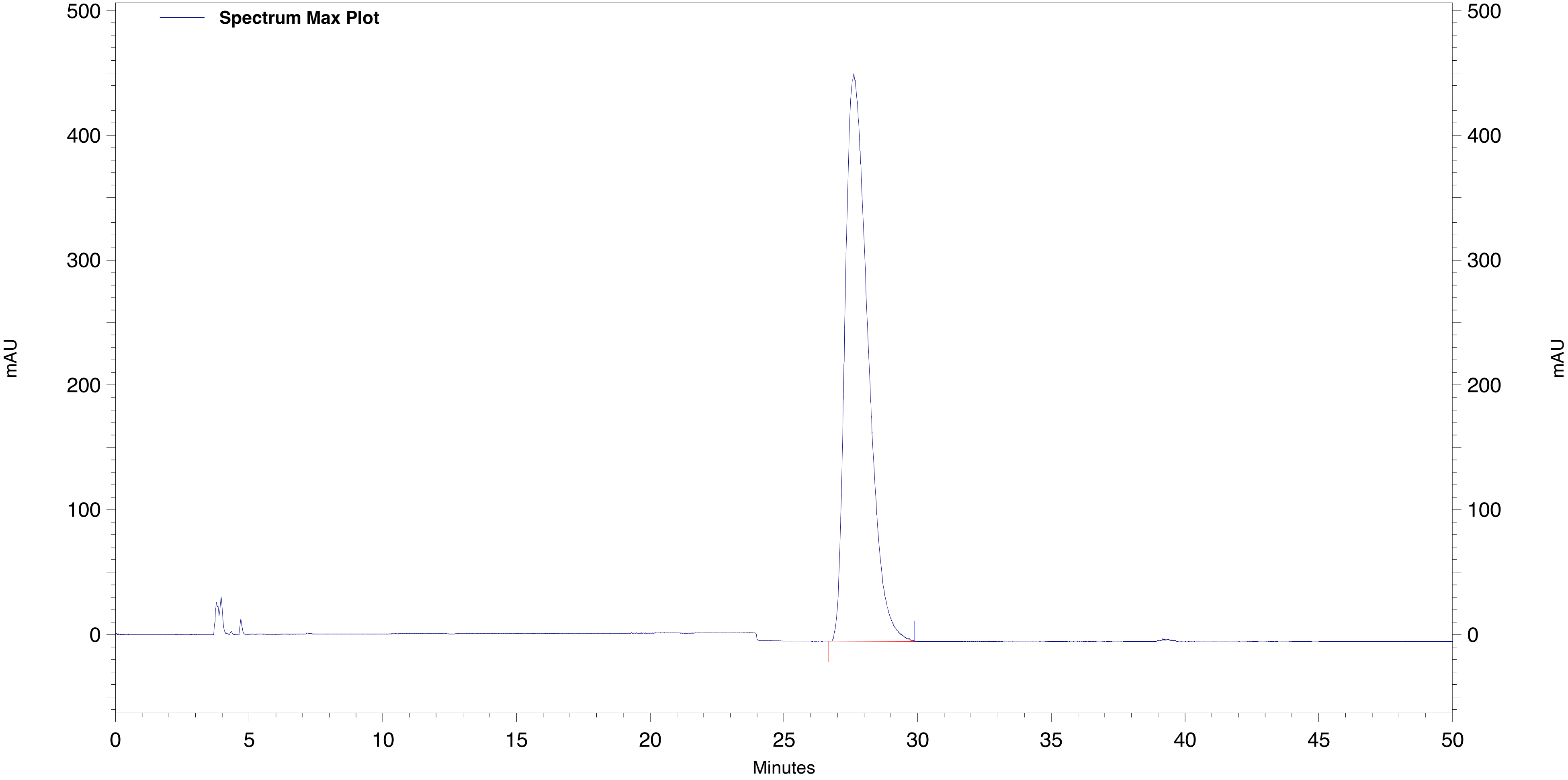

Compound 19b: Chiral HPLC
(Daicel Chiralpak AD-H, isocratic heptane/ethanol 85:5)

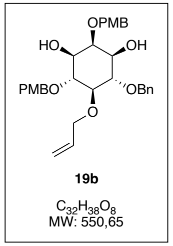

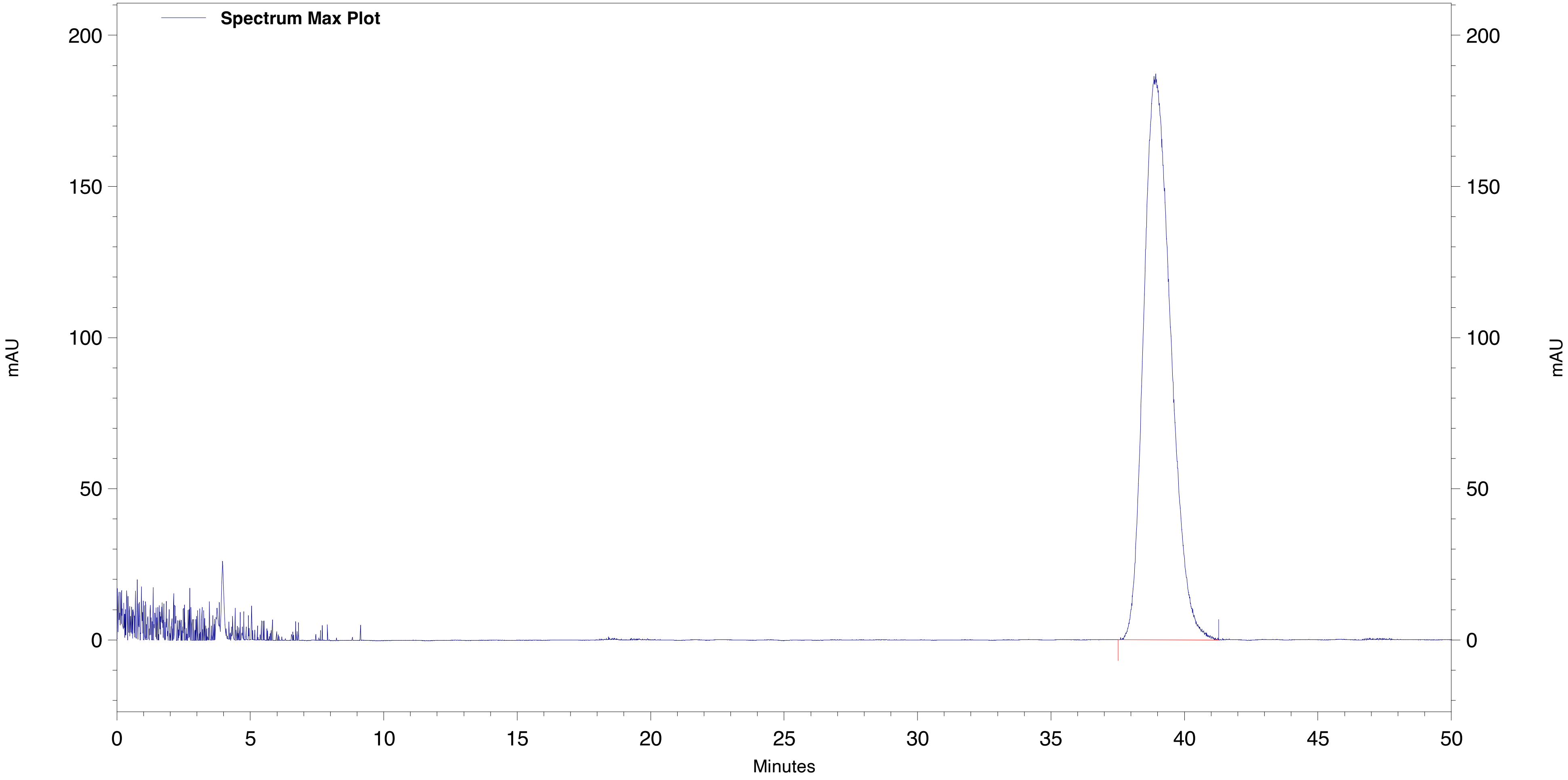

Compound ±21: HRMS (ESI) Analysis

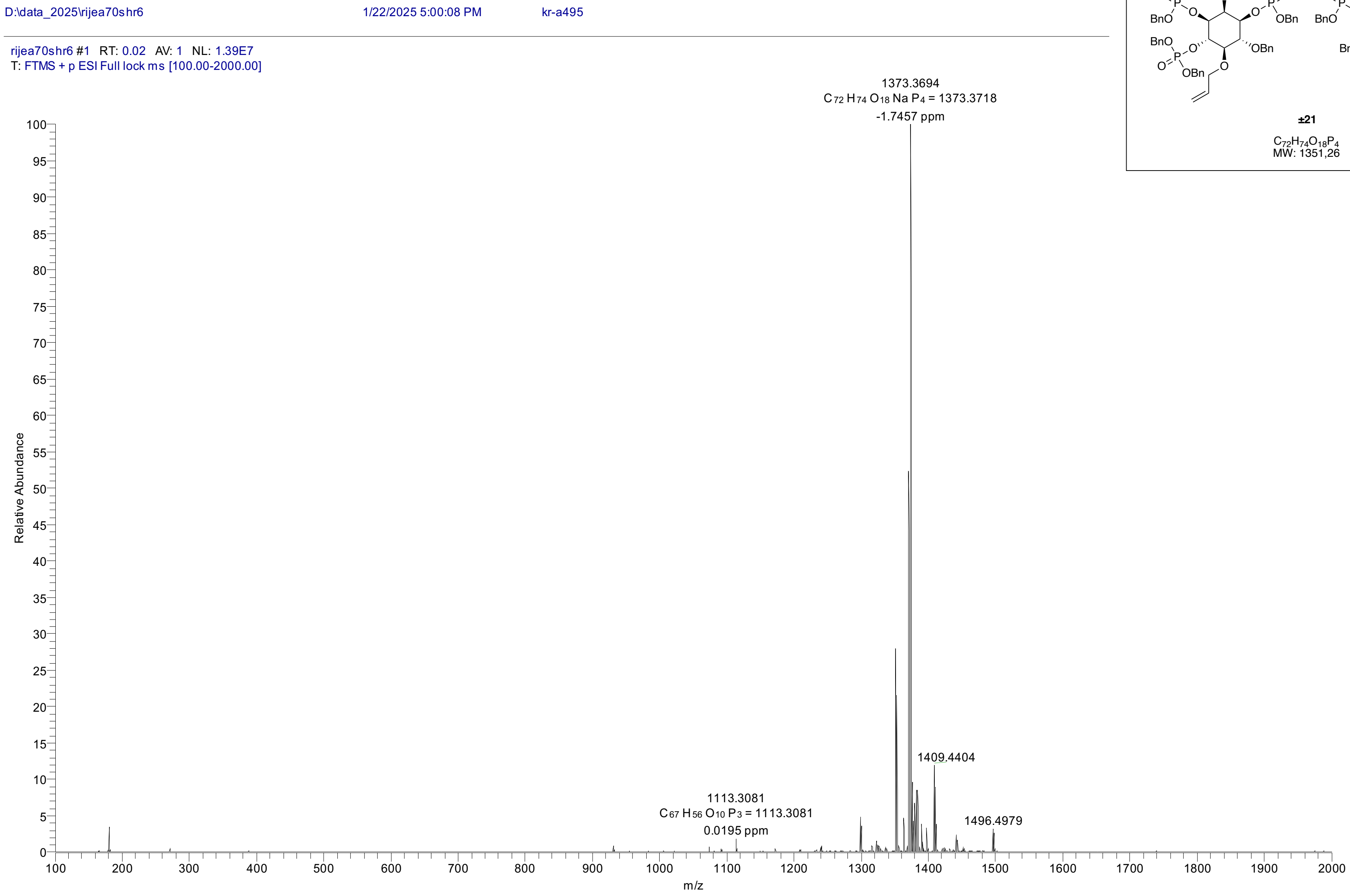

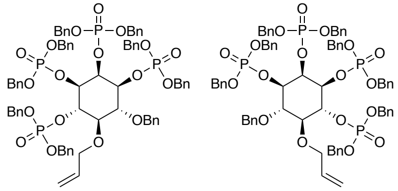

±21

$C_{72}H_{74}O_{18}P_4$
MW: 1351,26

Compound ±23: HRMS (ESI) Analysis

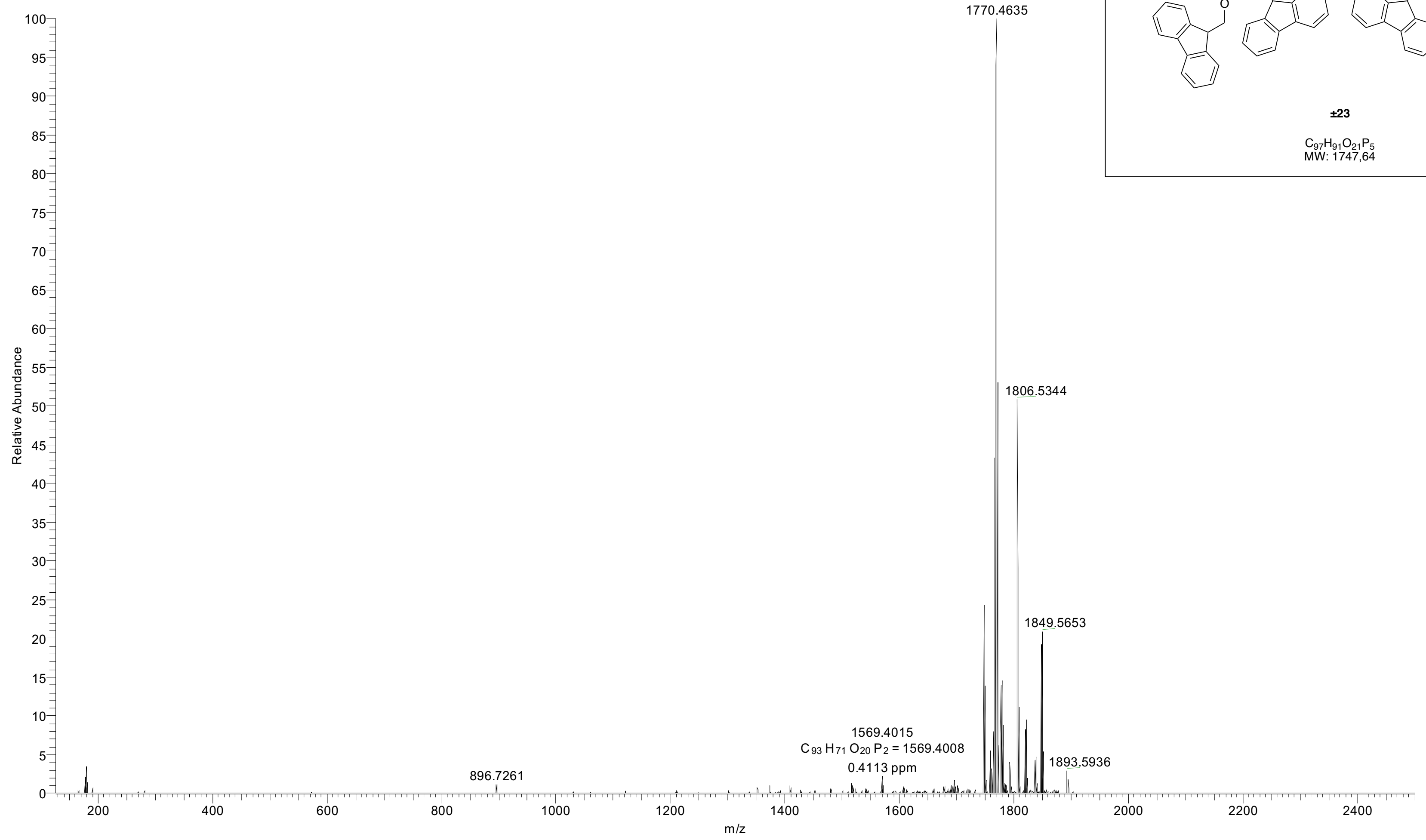

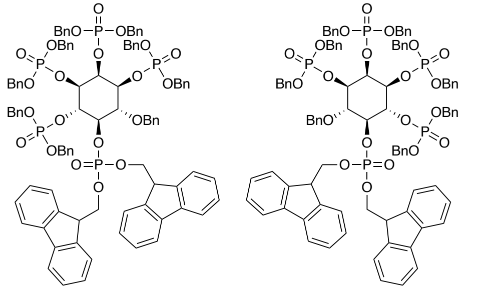

±23

$C_{97}H_{91}O_{21}P_5$
MW: 1747,64

Compound ±23: HRMS (ESI) Analysis

D:\data_2025\01\2201\rijea68shr1 1/22/2025 5:19:52 PM kr-a526

rijea68shr1 #1 RT: 0.03 AV: 1 NL: 1.26E7
T: FTMS + p ESI Full lock ms [125.00-2500.00]

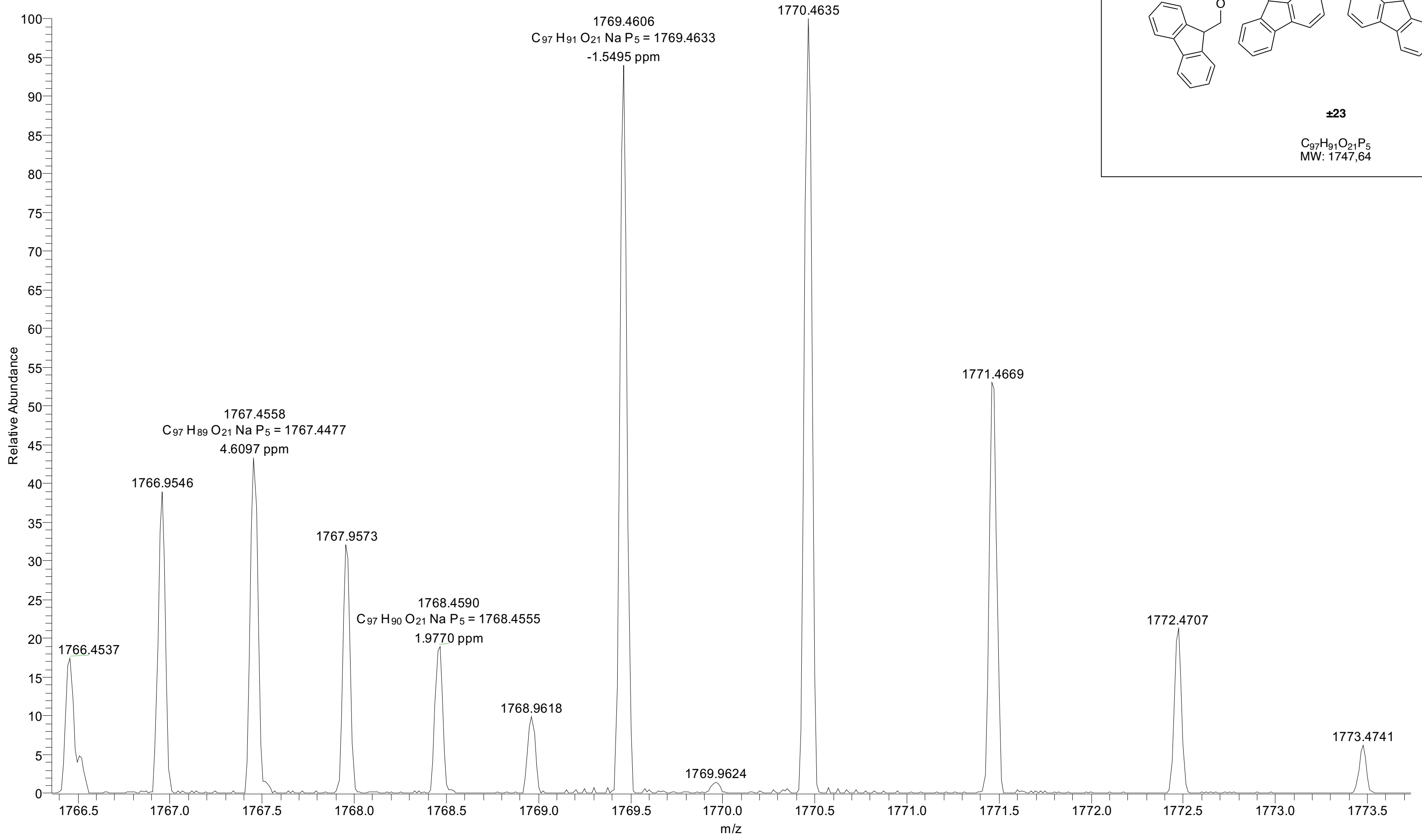

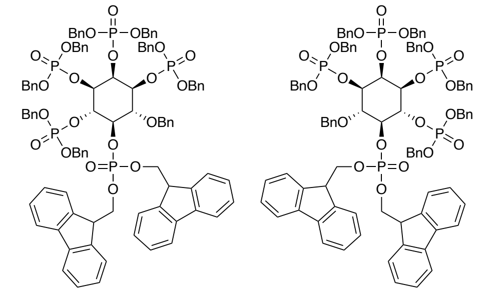

±23

$C_{97}H_{91}O_{21}P_5$
MW: 1747,64

Compound ±24: HRMS (ESI) Analysis

D:\data_2025\rijea67shr1 1/22/2025 3:30:54 PM kr-a532

rijea67shr1 #1 RT: 0.02 AV: 1 NL: 3.83E7
T: FTMS - p ESI Full lock ms [100.00-2000.00]

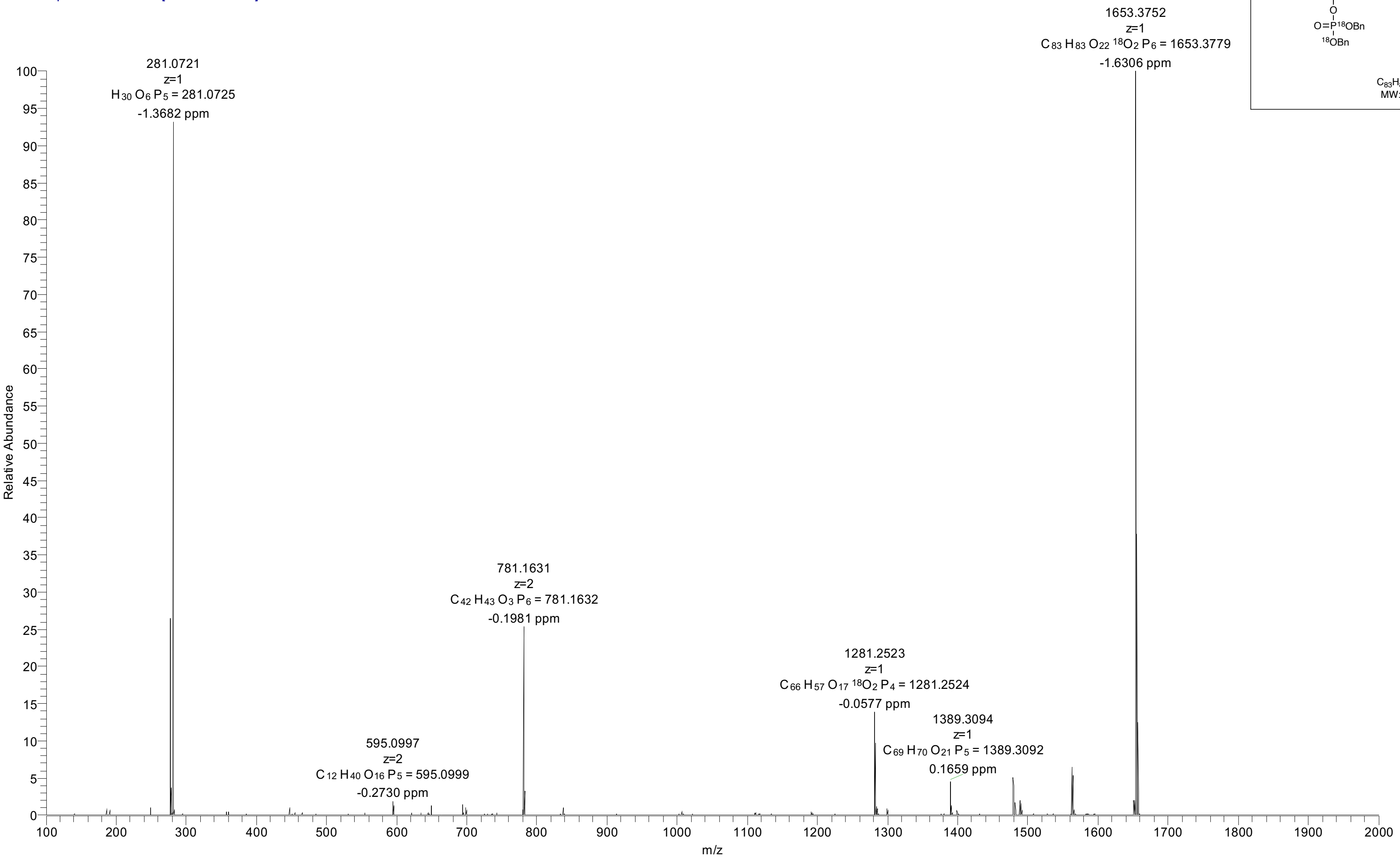

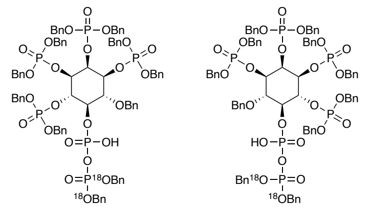

±24

$C_{83}H_{84}O_{22}{}^{18}O_2P_6$
MW: 1655,4039

Compound 24a: HRMS (ESI) Analysis

D:\data_2025\rijea69shr4 1/22/2025 3:40:28 PM kr-a530

rijea69shr4 #1 RT: 0.02 AV: 1 NL: 3.80E7
T: FTMS - p ESI Full lock ms [100.00-2000.00]

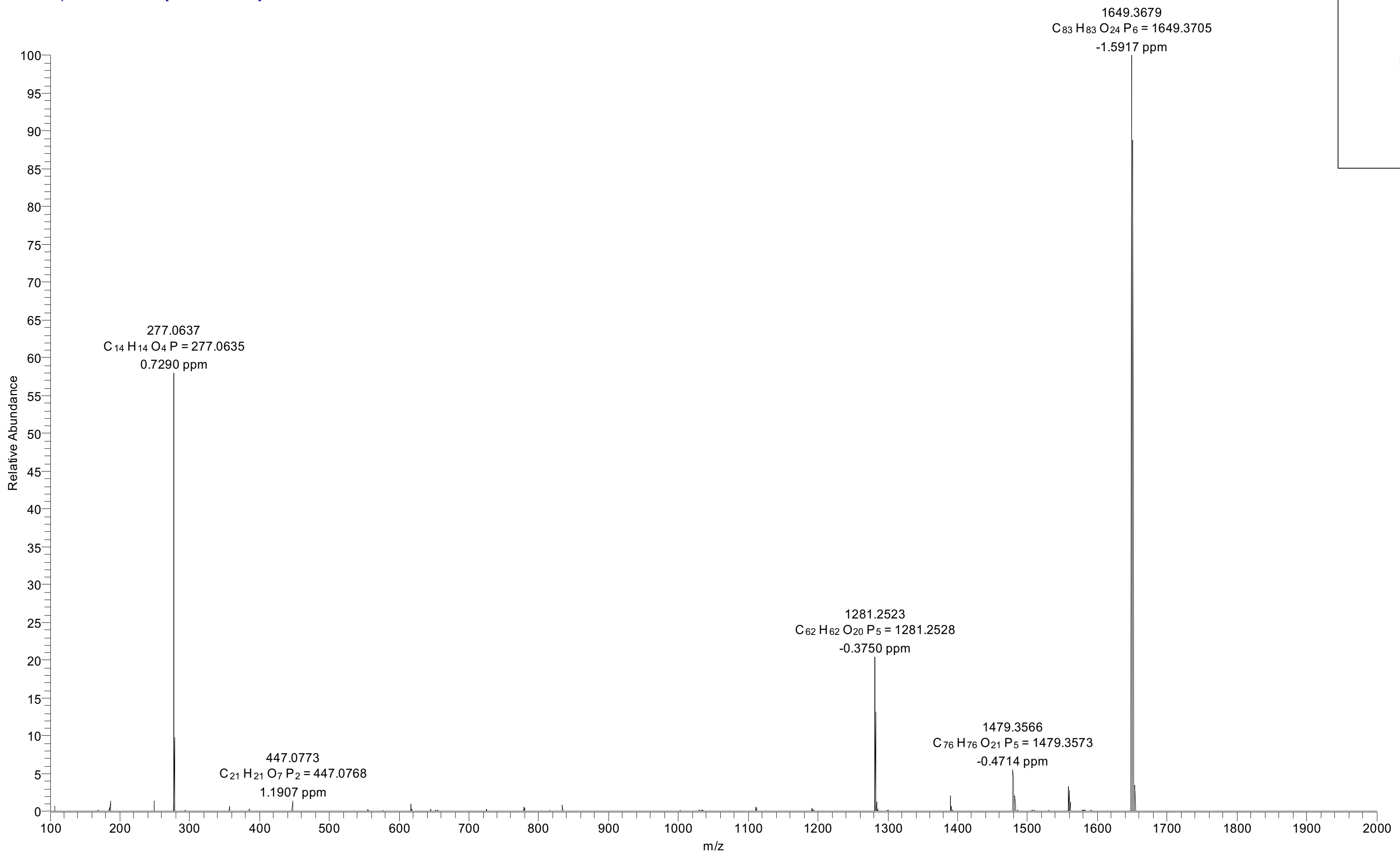

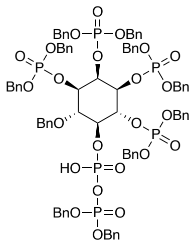

24a

$C_{83}H_{84}O_{24}P_6$
MW: 1651,40

Compound ±25: CE-qTOF electropherogram

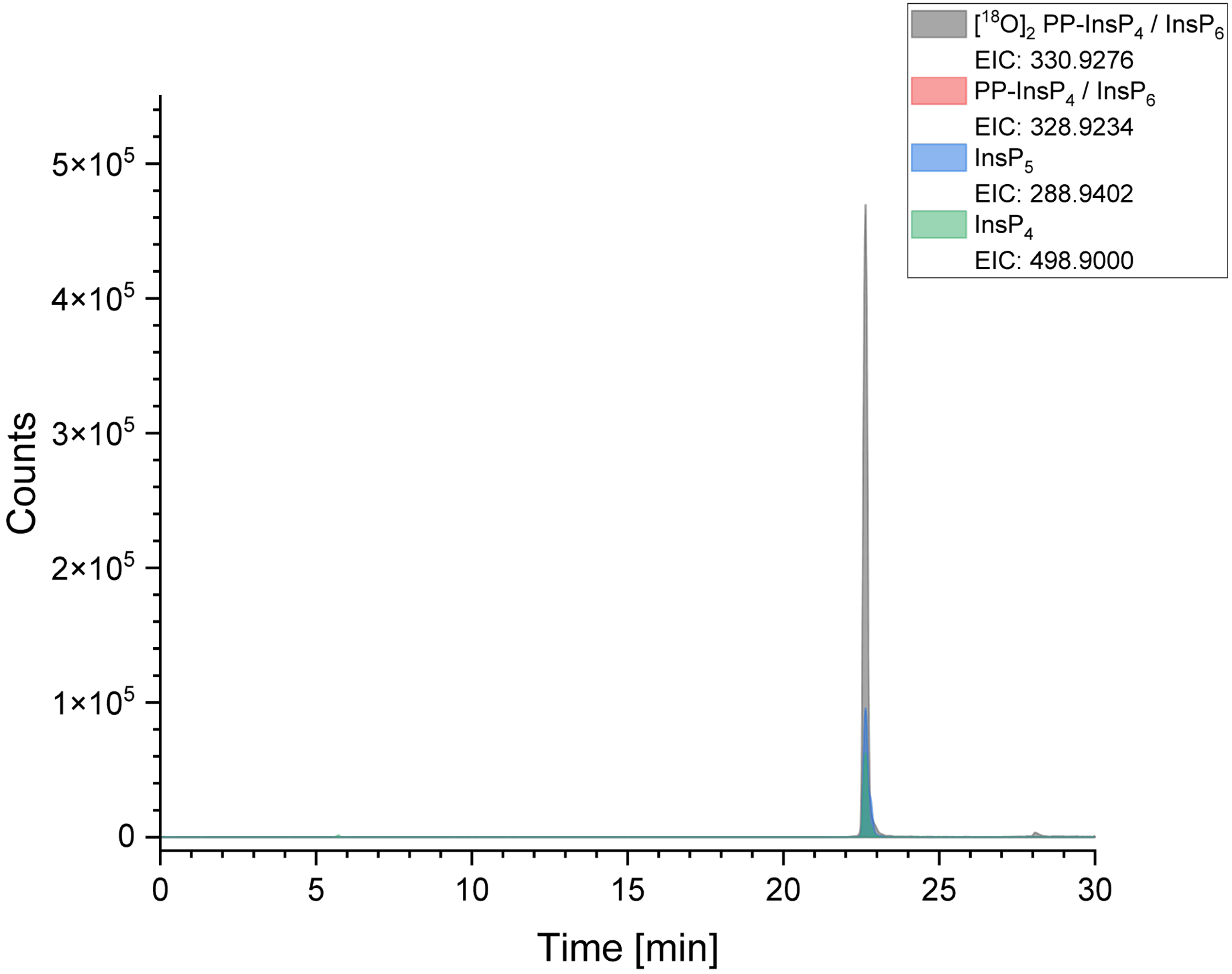

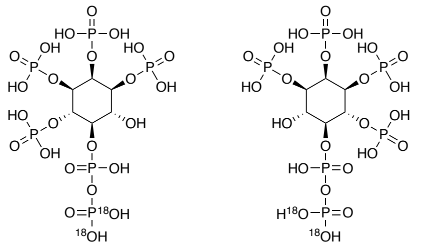

±25

$C_6H_{18}O_{22}{}^{18}O_2P_6$

MW: 664,0289

Compound ±25: HRMS (CE-qTOF) Analysis

**Sample Name**	KR-A542	**Position**	09	**Instrument Name**	QTOF-1
**User Name**		**Inj Vol**	Unknown / Injection Program	**InjPosition**	
**Sample Type**	Sample	**IRM Calibration Status**	Success	**Data Filename**	KR-A542.d
**ACQ Method**	Standard method.m	**Comment**		**Acquired Time**	13.12.2024 1

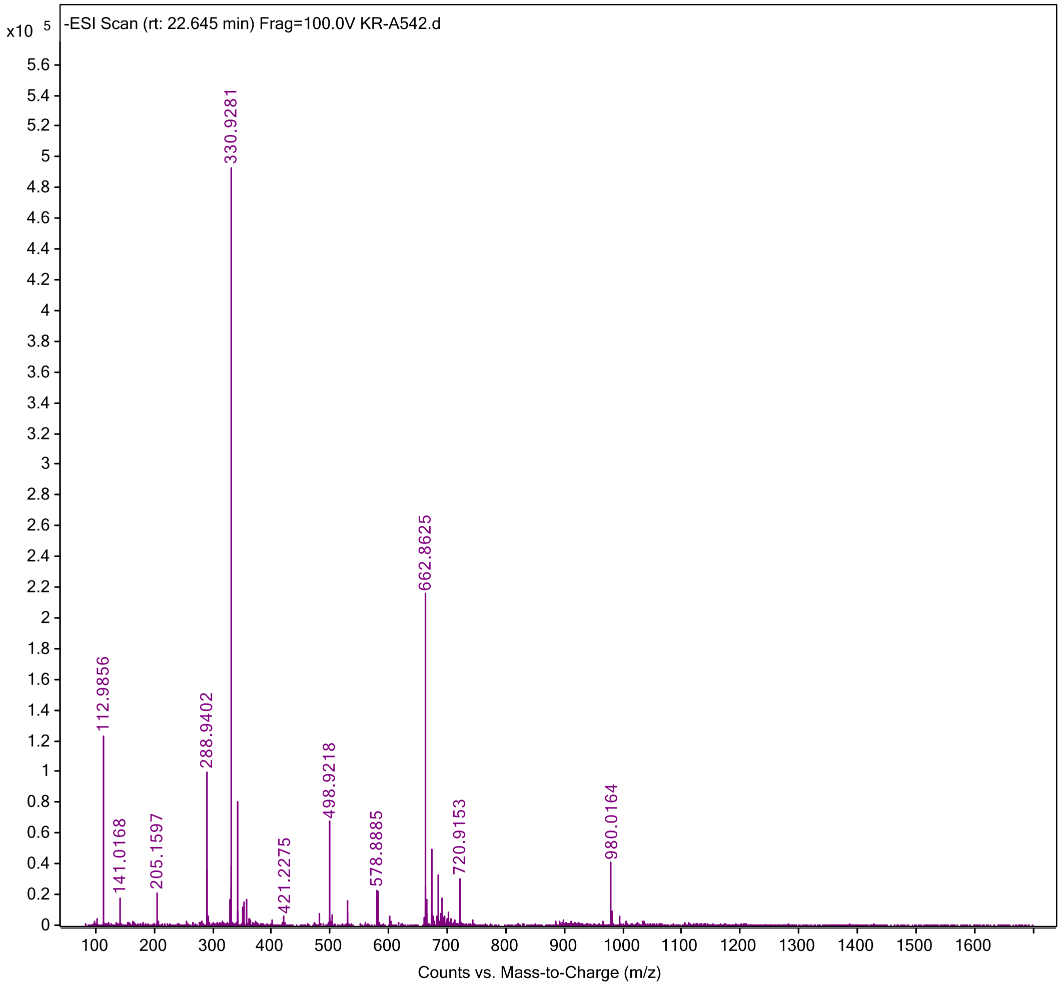

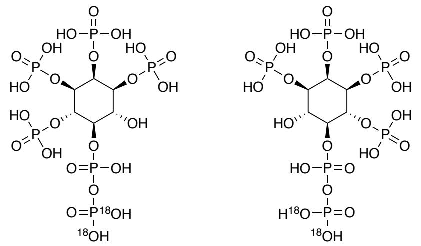

±25

$C_6H_{18}O_{22}{}^{18}O_2P_6$

MW: 664,0289

Compound 25a: CE-qTOF electropherogram

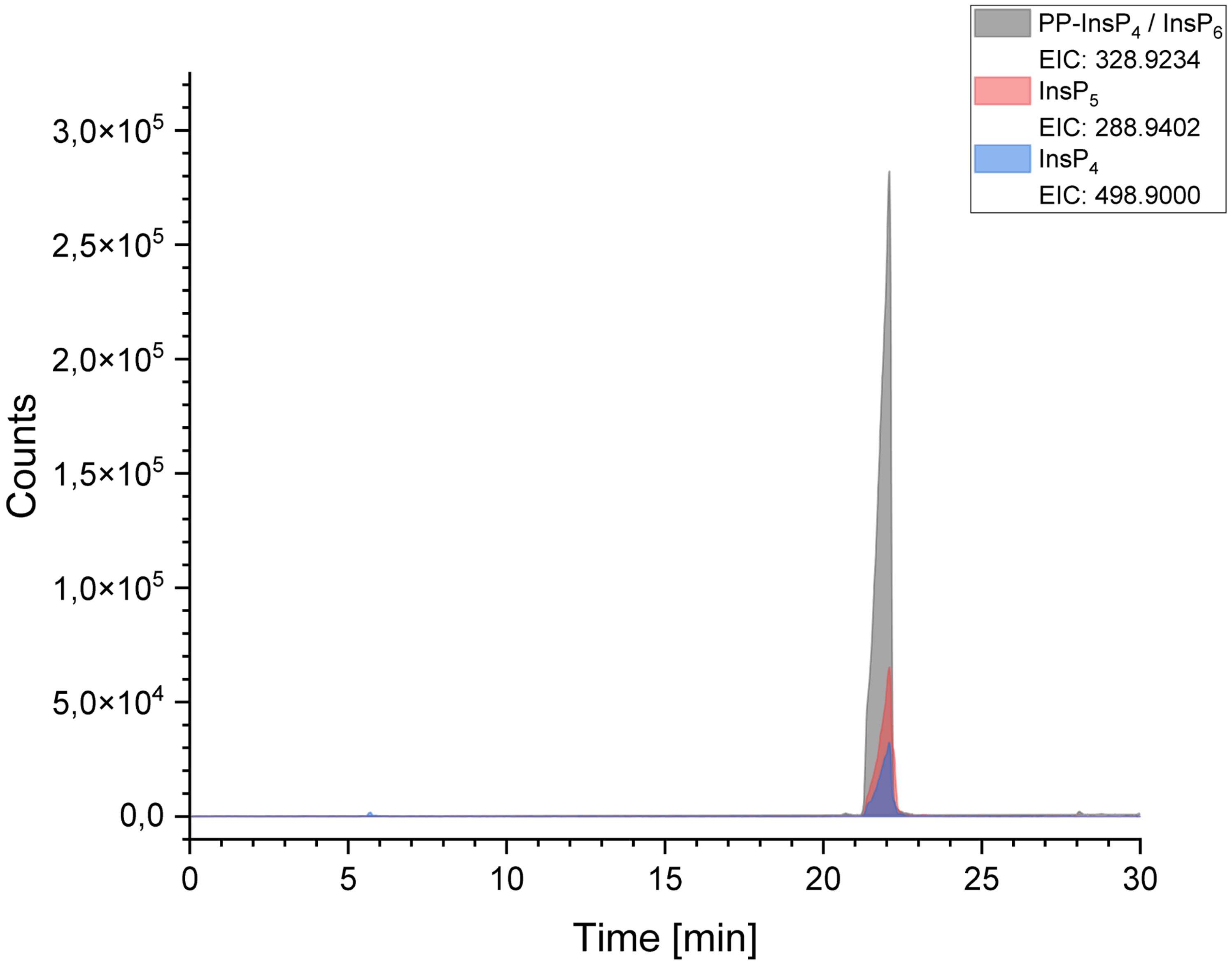

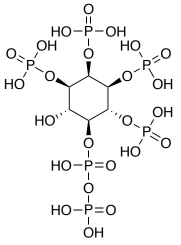

25a

$C_6H_{18}O_{24}P_6$
MW: 660,0286

Compound 25b: CE-qTOF electropherogram

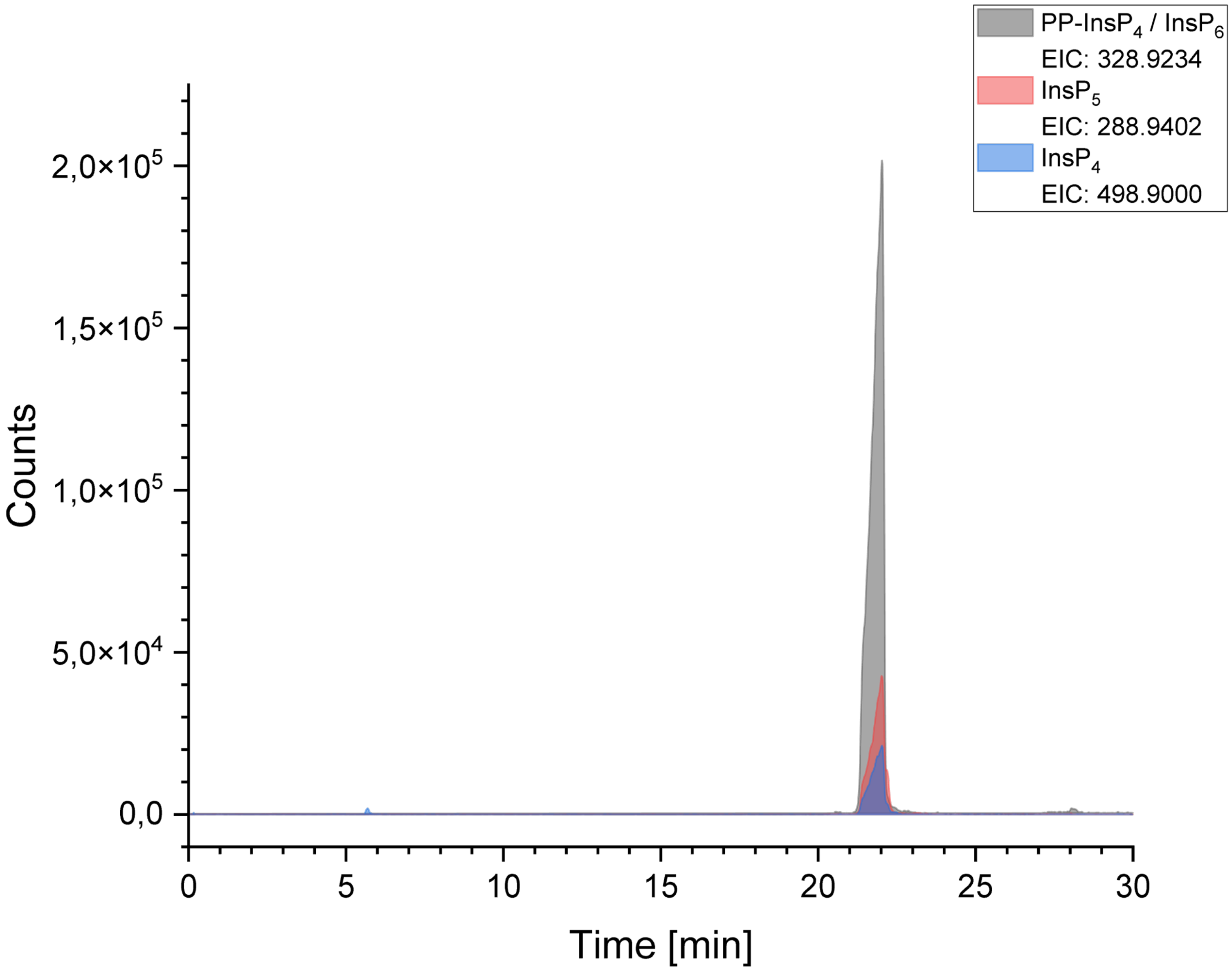

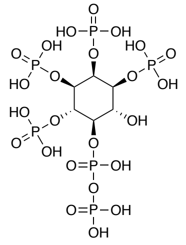

25b

$C_6H_{18}O_{24}P_6$
MW: 660,0286

Compound 26a: CE-qTOF electropherogram

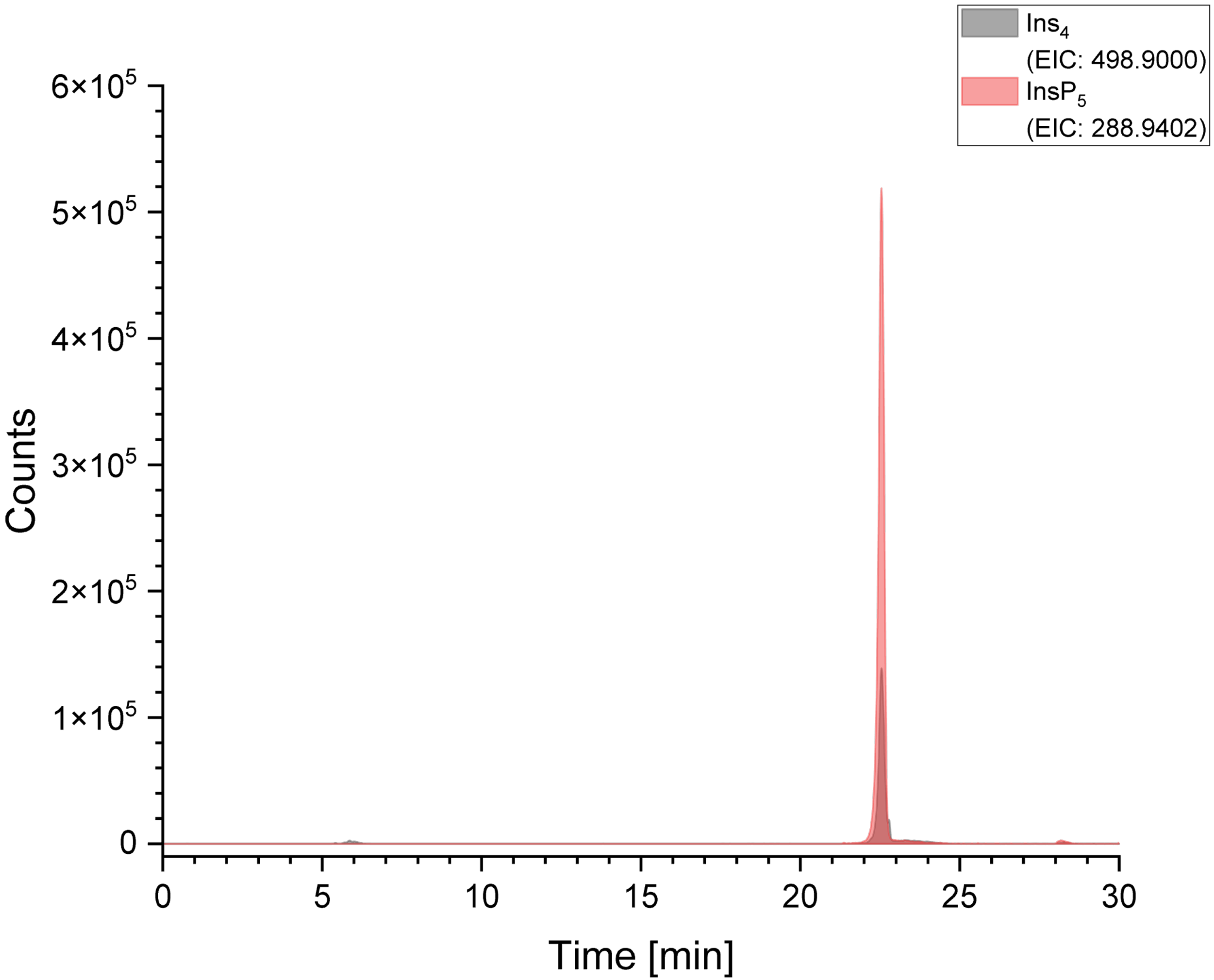

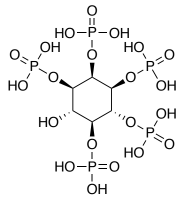

26a

$C_6H_{17}O_{21}P_5$
MW: 580,05

Compound 26b: CE-qTOF electropherogram

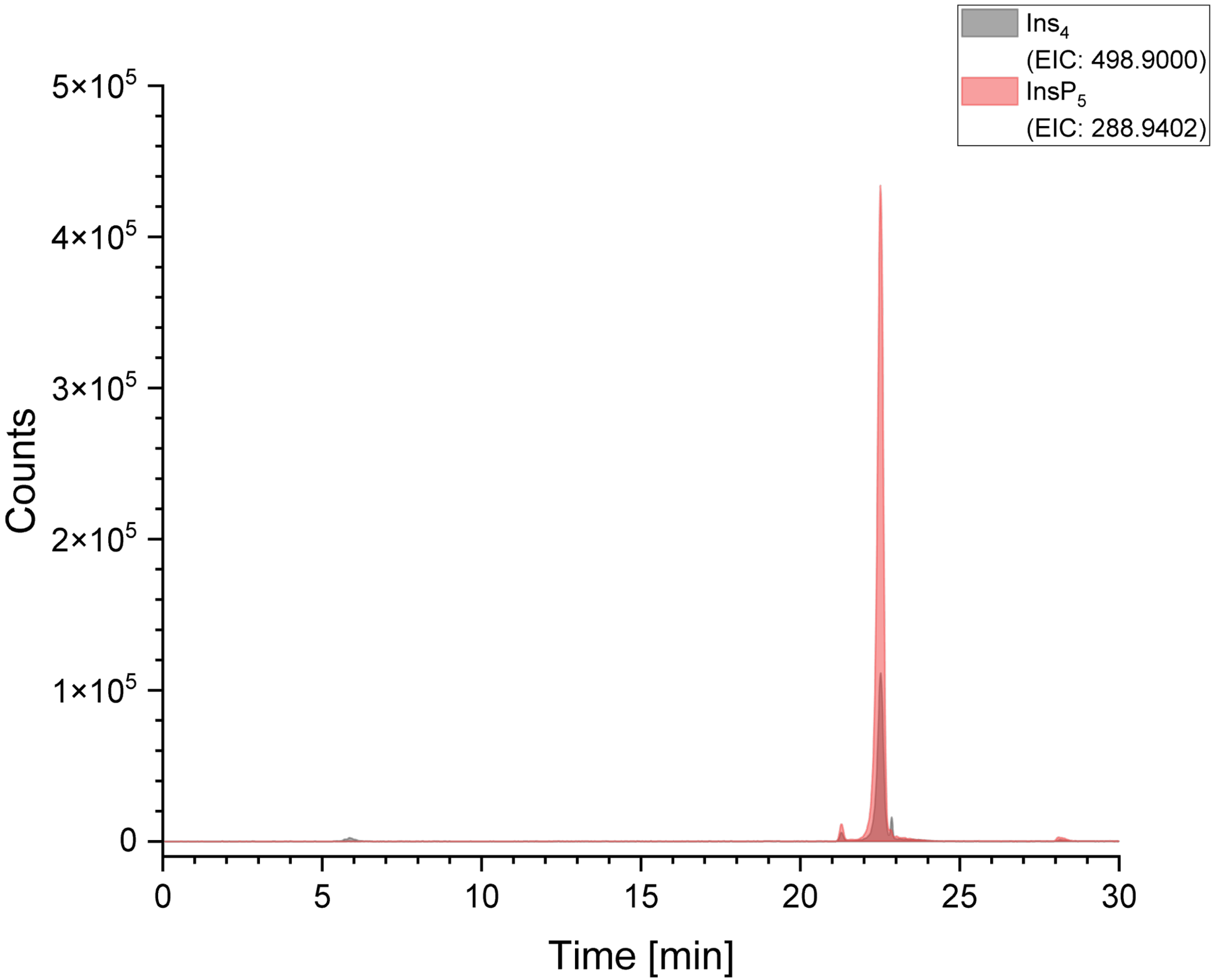

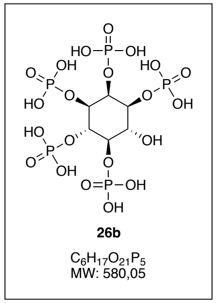

Supplement: Supplementary file 1 — Supporting Information [file ANIE-64-e202507058-s001.pdf]
